# Supplementary figures and images for: Cytosolic and endoplasmic reticulum chaperones inhibit wt-p53 to increase cancer cells' survival by refluxing ER-proteins to the cytosol (part 1 of 3)
Source: eLife. 2025 Apr 9;14:e102658. doi: 10.7554/eLife.102658 (PMC11981610; doi:10.7554/eLife.102658)

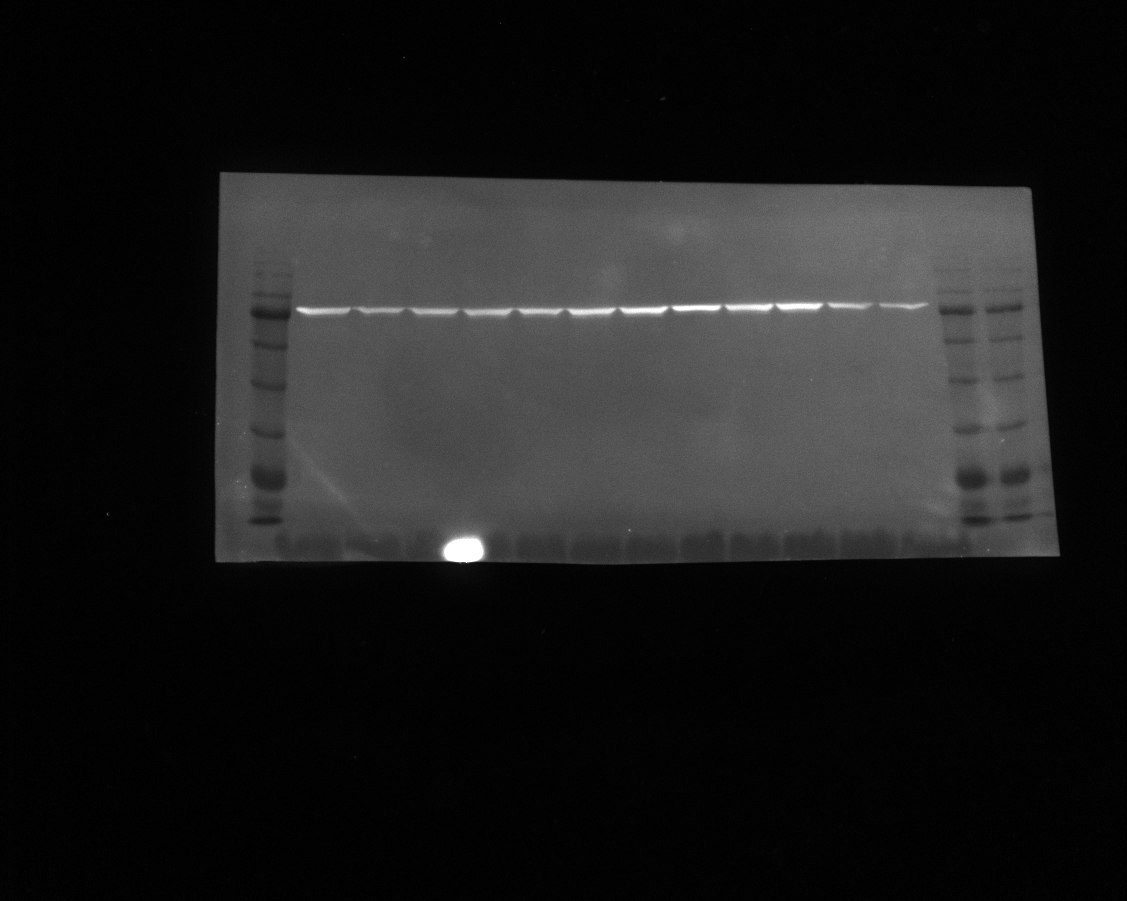

Supplement: Figure 2—source data 1. [file elife-102658-fig2-data1.zip › Figure 2-source data1 copy/Figure 2D-4-source data1.tif]

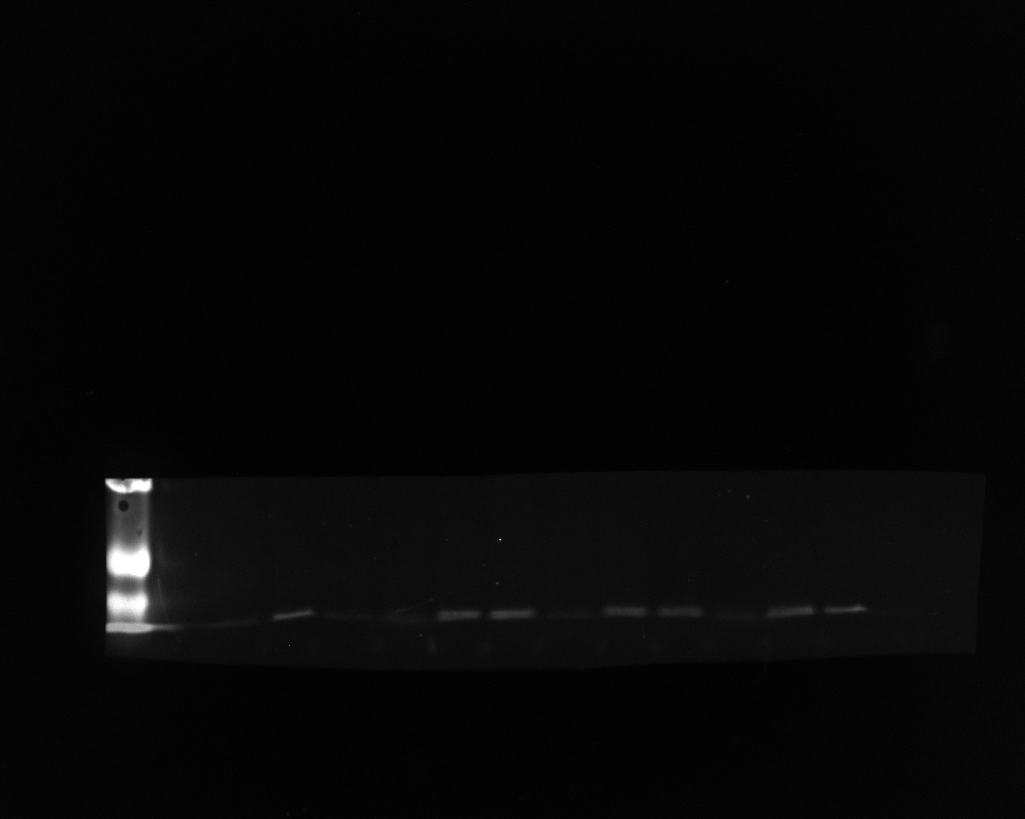

Supplement: Figure 2—source data 1. [file elife-102658-fig2-data1.zip › Figure 2-source data1 copy/Figure 2D-1-source data1.tif]

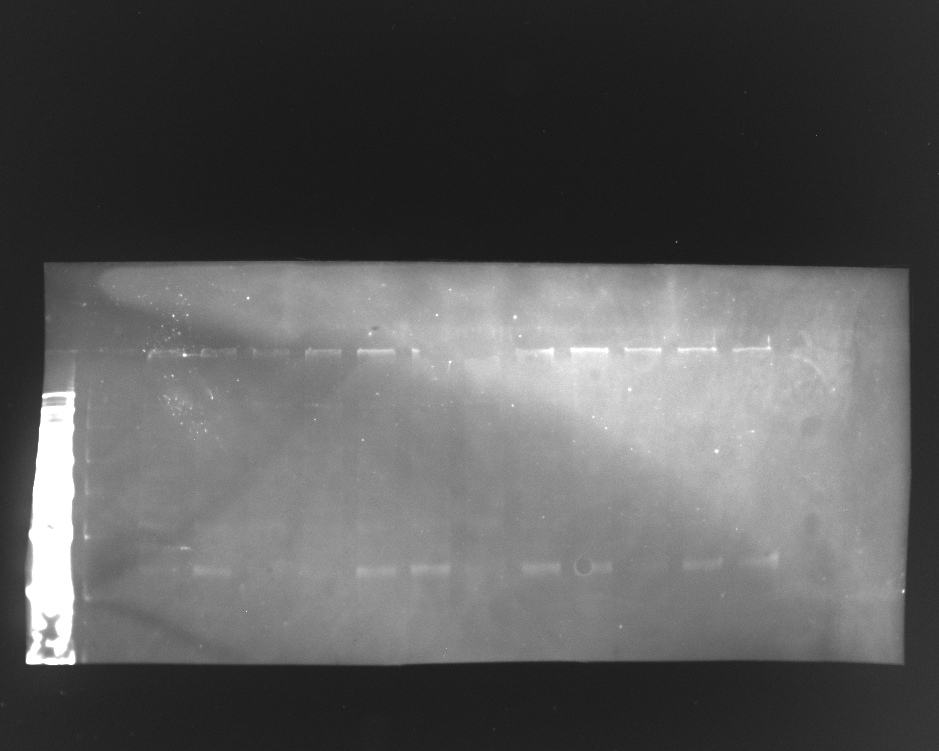

Supplement: Figure 2—source data 1. [file elife-102658-fig2-data1.zip › Figure 2-source data1 copy/Figure 2D-2-source data1.tif]

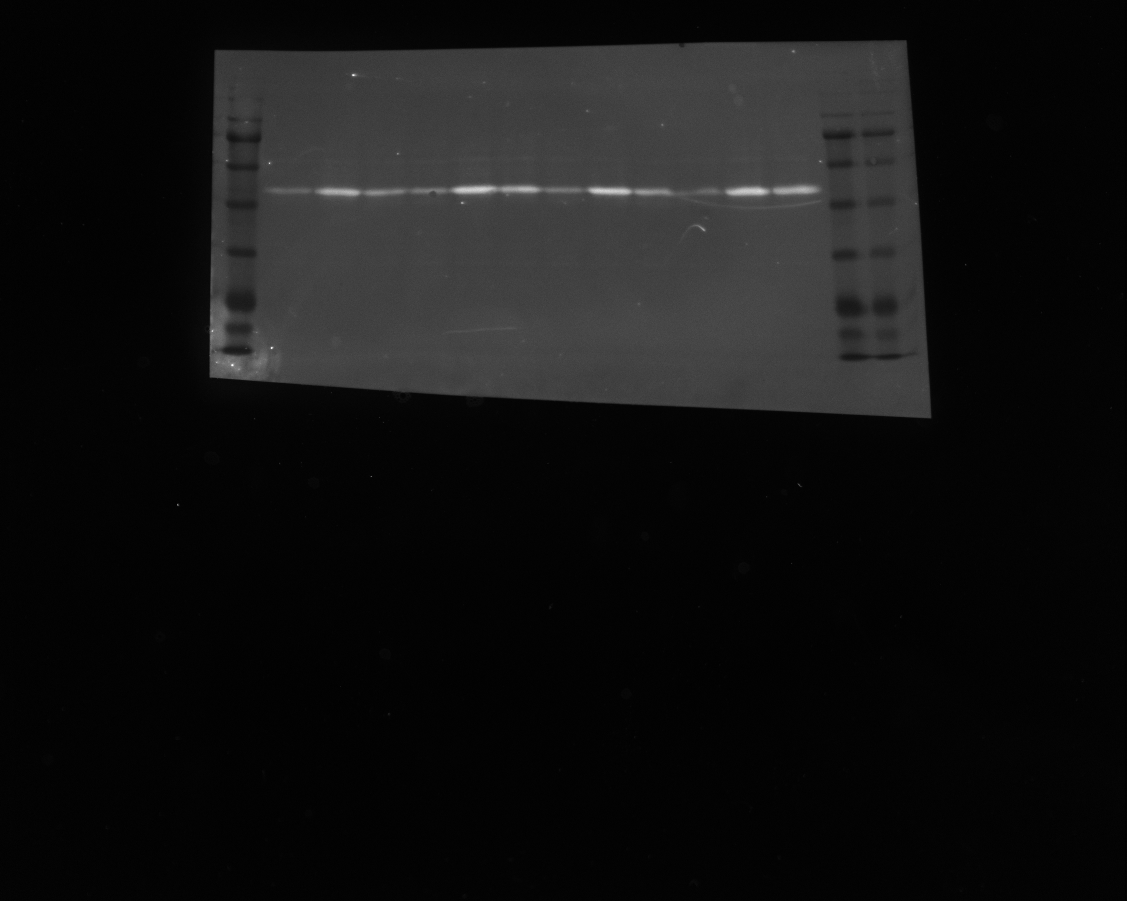

Supplement: Figure 2—source data 1. [file elife-102658-fig2-data1.zip › Figure 2-source data1 copy/Figure 2D-3-source data1.tif]

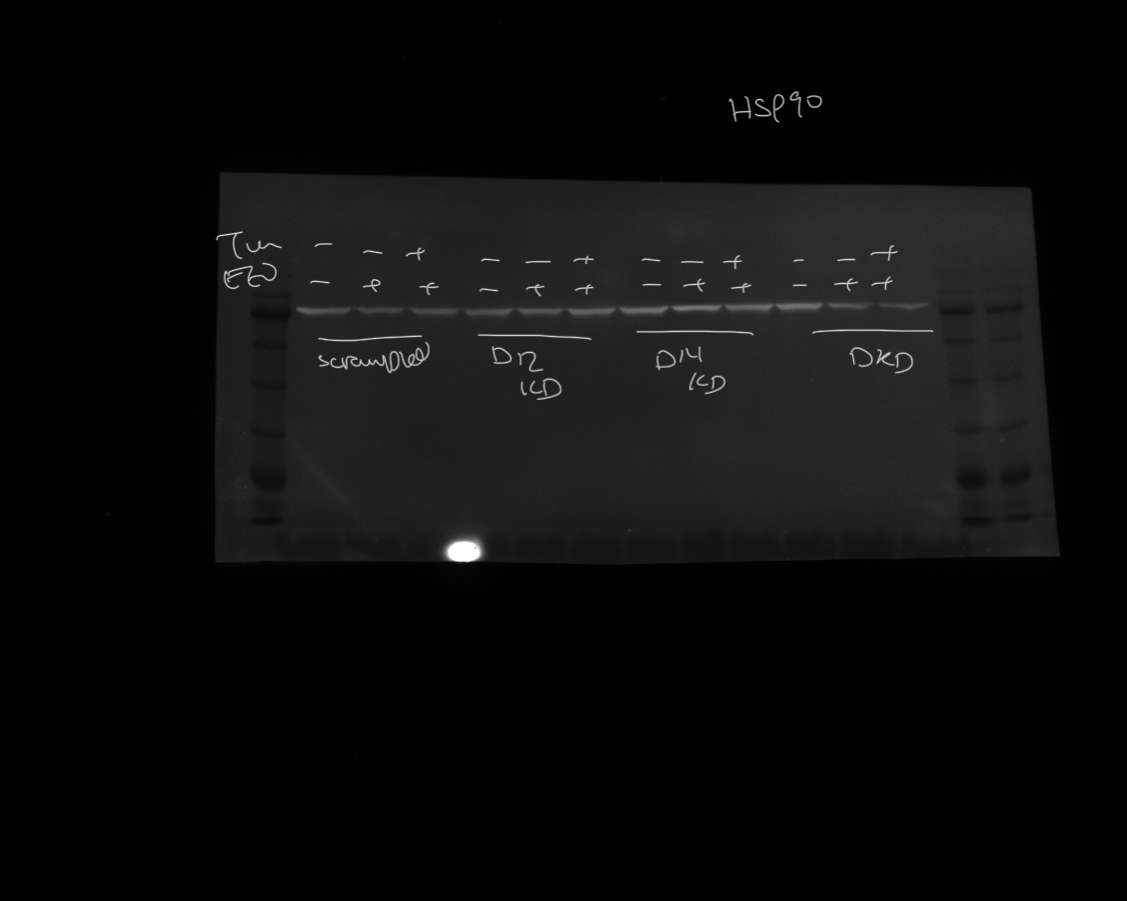

Supplement: Figure 2—source data 2. [file elife-102658-fig2-data2.zip › Figure 2-source data1 copy 2/Figure 2D-4-source data1.tif]

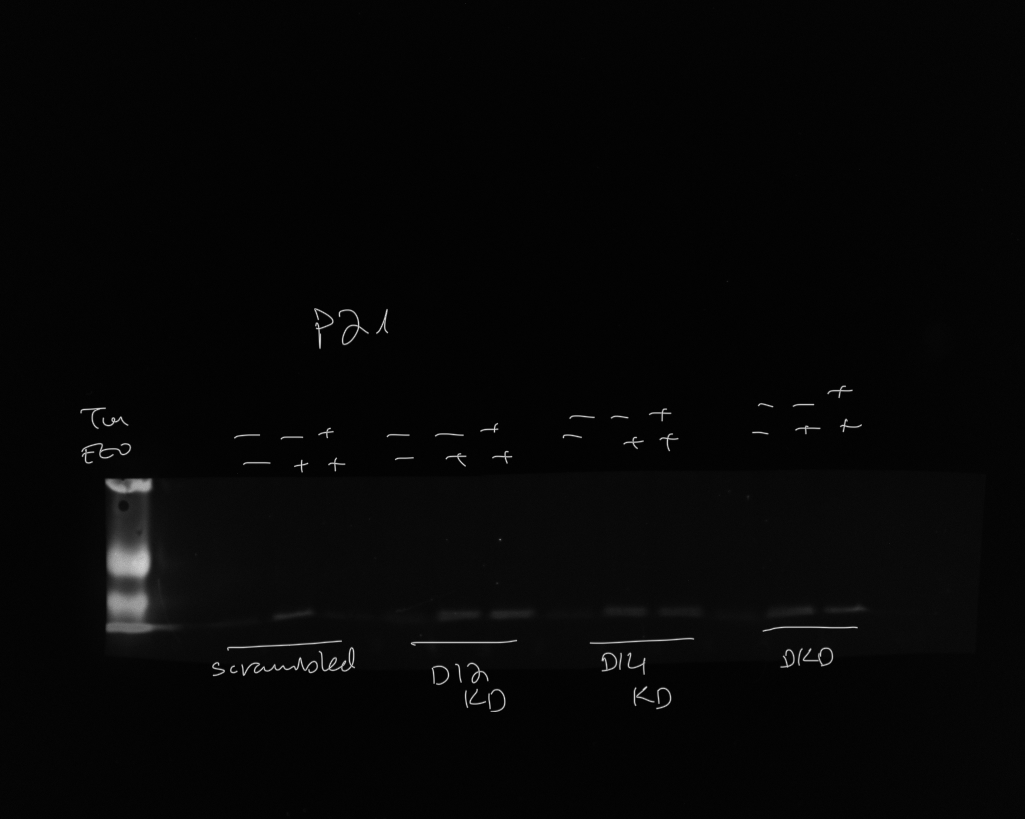

Supplement: Figure 2—source data 2. [file elife-102658-fig2-data2.zip › Figure 2-source data1 copy 2/Figure 2D-1-source data1.tif]

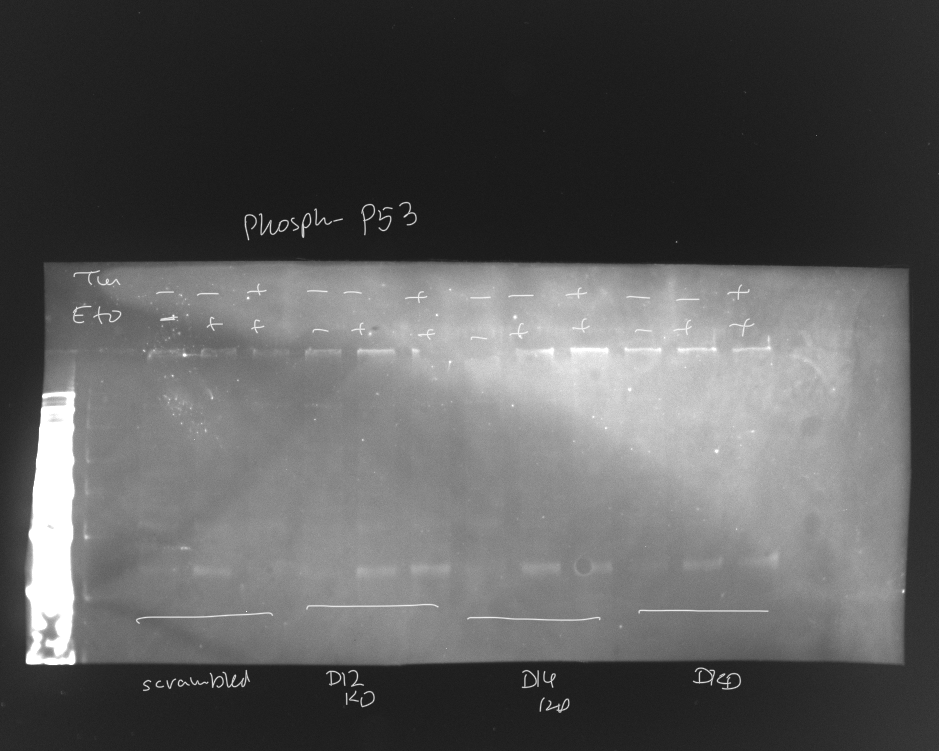

Supplement: Figure 2—source data 2. [file elife-102658-fig2-data2.zip › Figure 2-source data1 copy 2/Figure 2D-2-source data1.tif]

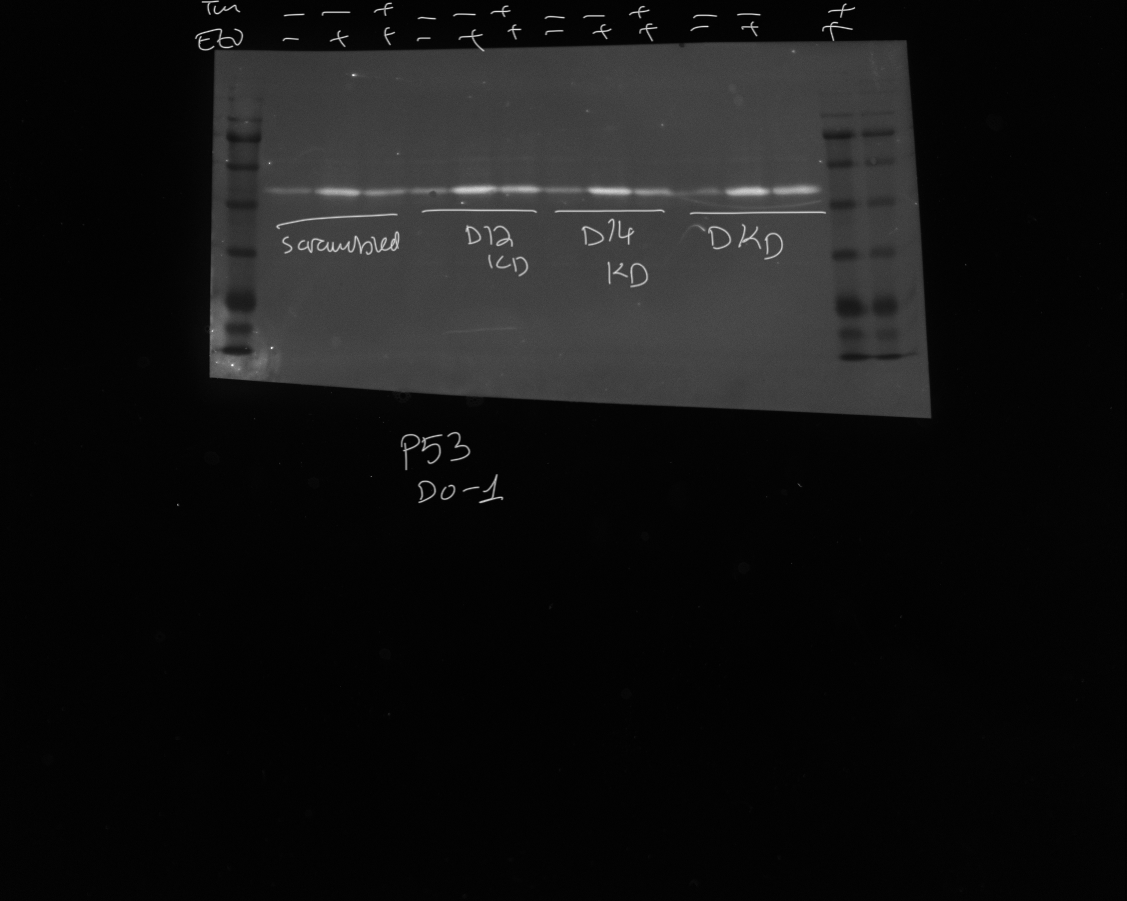

Supplement: Figure 2—source data 2. [file elife-102658-fig2-data2.zip › Figure 2-source data1 copy 2/Figure 2D-3-source data1.tif]

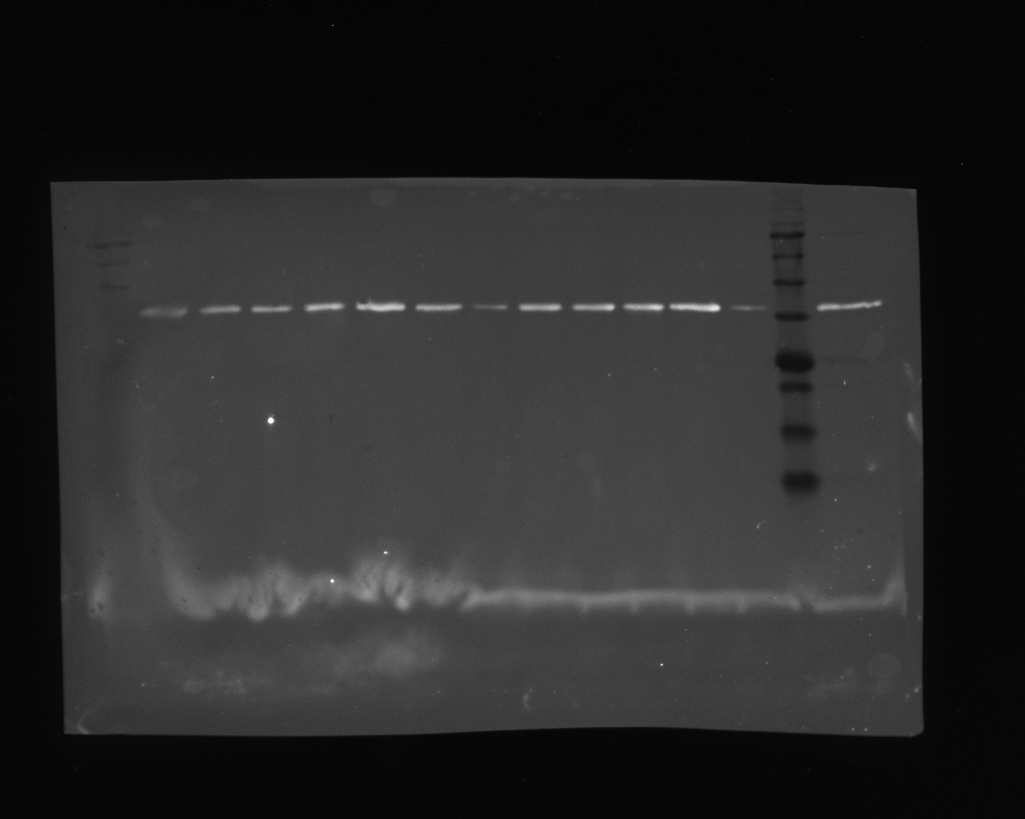

Supplement: Figure 2—figure supplement 1—source data 1. [file elife-102658-fig2-figsupp1-data1.zip › Figure 2-figure suplement 1-source data1 copy/Figure 2-figure suplemment 1-A-B-3-source data1.tif]

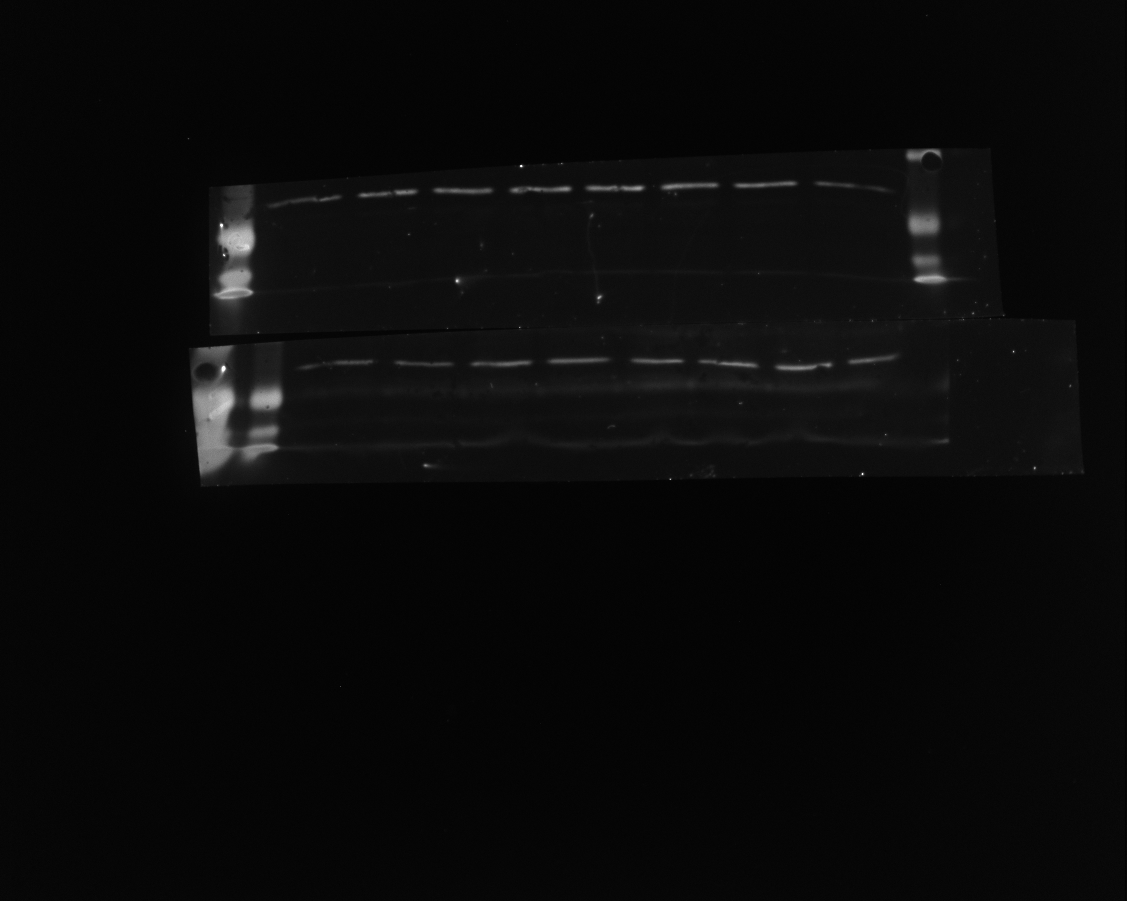

Supplement: Figure 2—figure supplement 1—source data 1. [file elife-102658-fig2-figsupp1-data1.zip › Figure 2-figure suplement 1-source data1 copy/Figure 2-figure suplemment 1-C-10-source data1.tif]

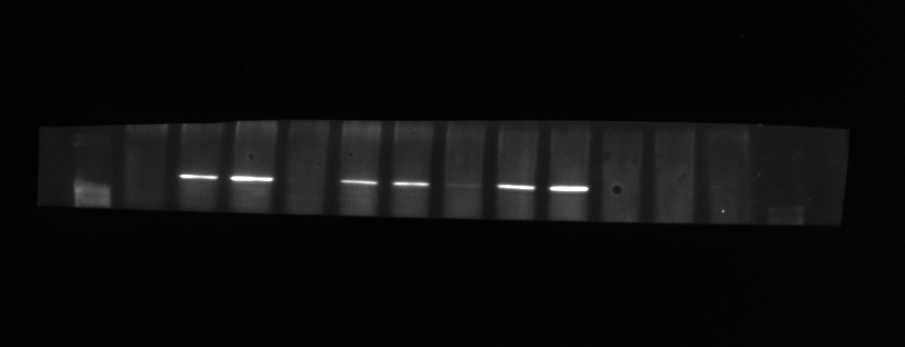

Supplement: Figure 2—figure supplement 1—source data 1. [file elife-102658-fig2-figsupp1-data1.zip › Figure 2-figure suplement 1-source data1 copy/Figure 2-figure suplemment 1-E-4-source data1.tif]

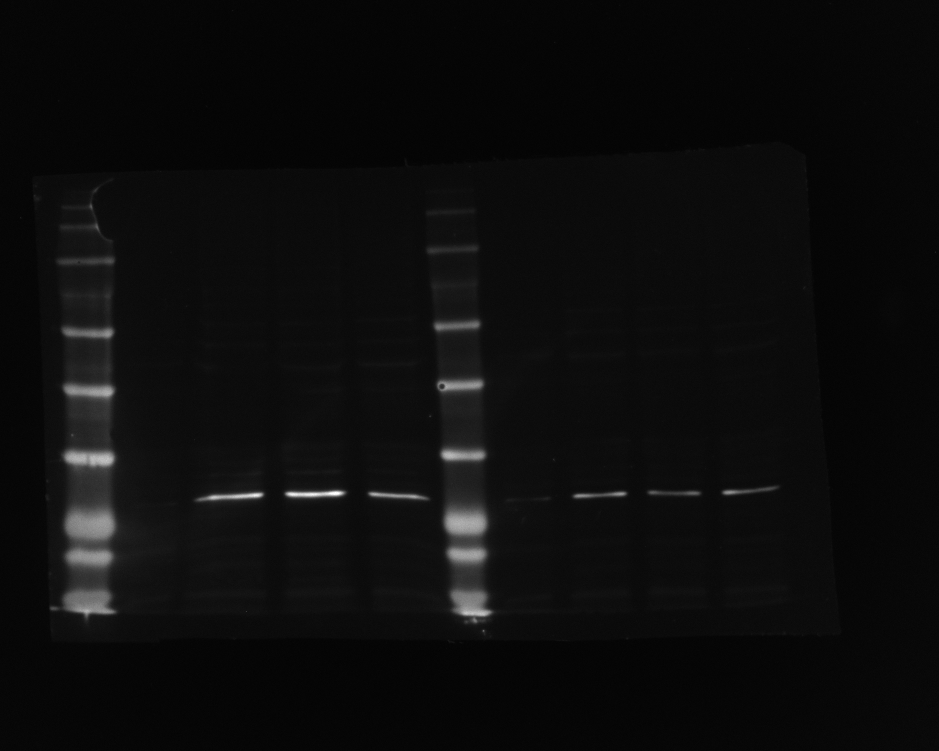

Supplement: Figure 2—figure supplement 1—source data 1. [file elife-102658-fig2-figsupp1-data1.zip › Figure 2-figure suplement 1-source data1 copy/Figure 2-figure suplemment 1-C-1-source data1.tif]

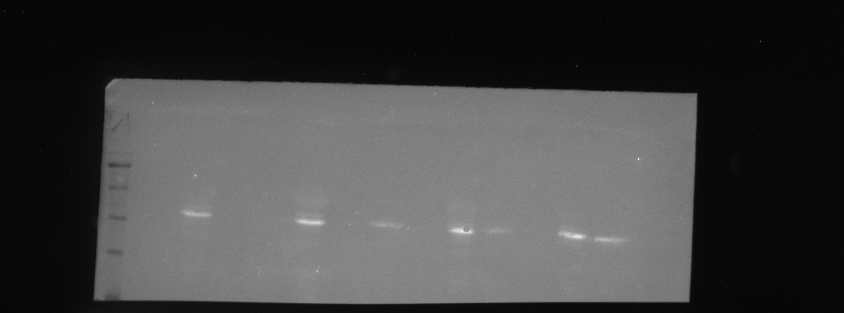

Supplement: Figure 2—figure supplement 1—source data 1. [file elife-102658-fig2-figsupp1-data1.zip › Figure 2-figure suplement 1-source data1 copy/Figure 2-figure suplemment 1-O-2-source data1.tif]

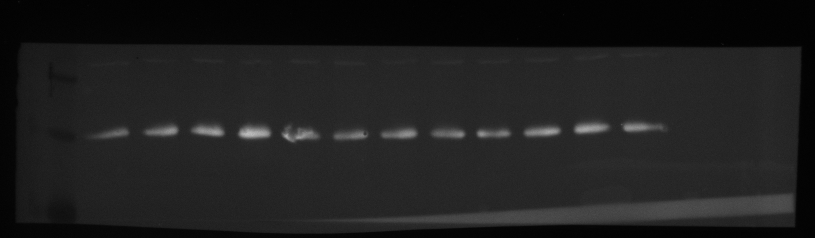

Supplement: Figure 2—figure supplement 1—source data 1. [file elife-102658-fig2-figsupp1-data1.zip › Figure 2-figure suplement 1-source data1 copy/Figure 2-figure suplemment 1-F-4-source data1.tif]

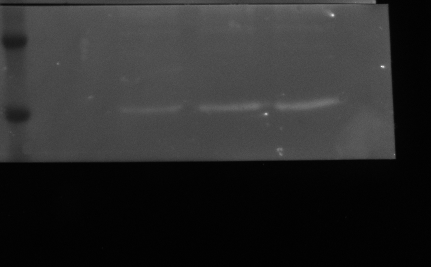

Supplement: Figure 2—figure supplement 1—source data 1. [file elife-102658-fig2-figsupp1-data1.zip › Figure 2-figure suplement 1-source data1 copy/Figure 2-figure suplemment 1-C-5-source data1.tif]

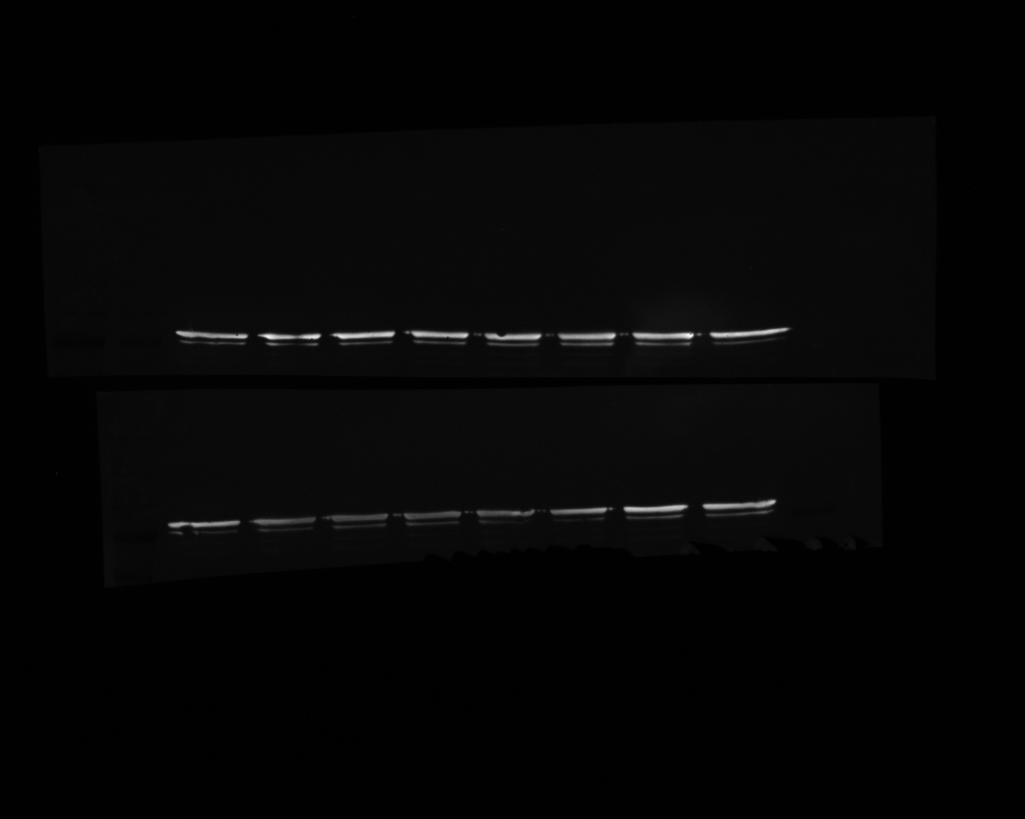

Supplement: Figure 2—figure supplement 1—source data 1. [file elife-102658-fig2-figsupp1-data1.zip › Figure 2-figure suplement 1-source data1 copy/Figure 2-figure suplemment 1-C-14-source data1.tif]

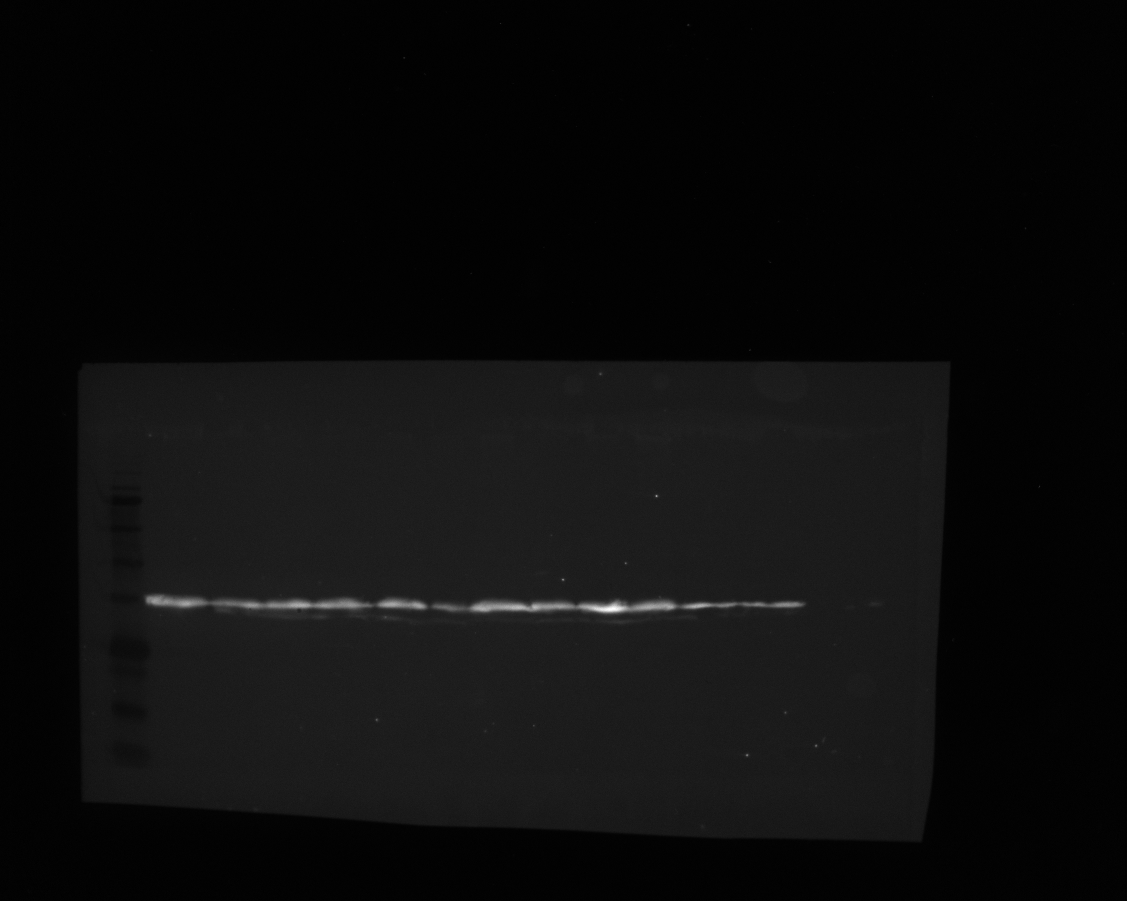

Supplement: Figure 2—figure supplement 1—source data 1. [file elife-102658-fig2-figsupp1-data1.zip › Figure 2-figure suplement 1-source data1 copy/Figure 2-figure suplemment 1-E-5-source data1.tif]

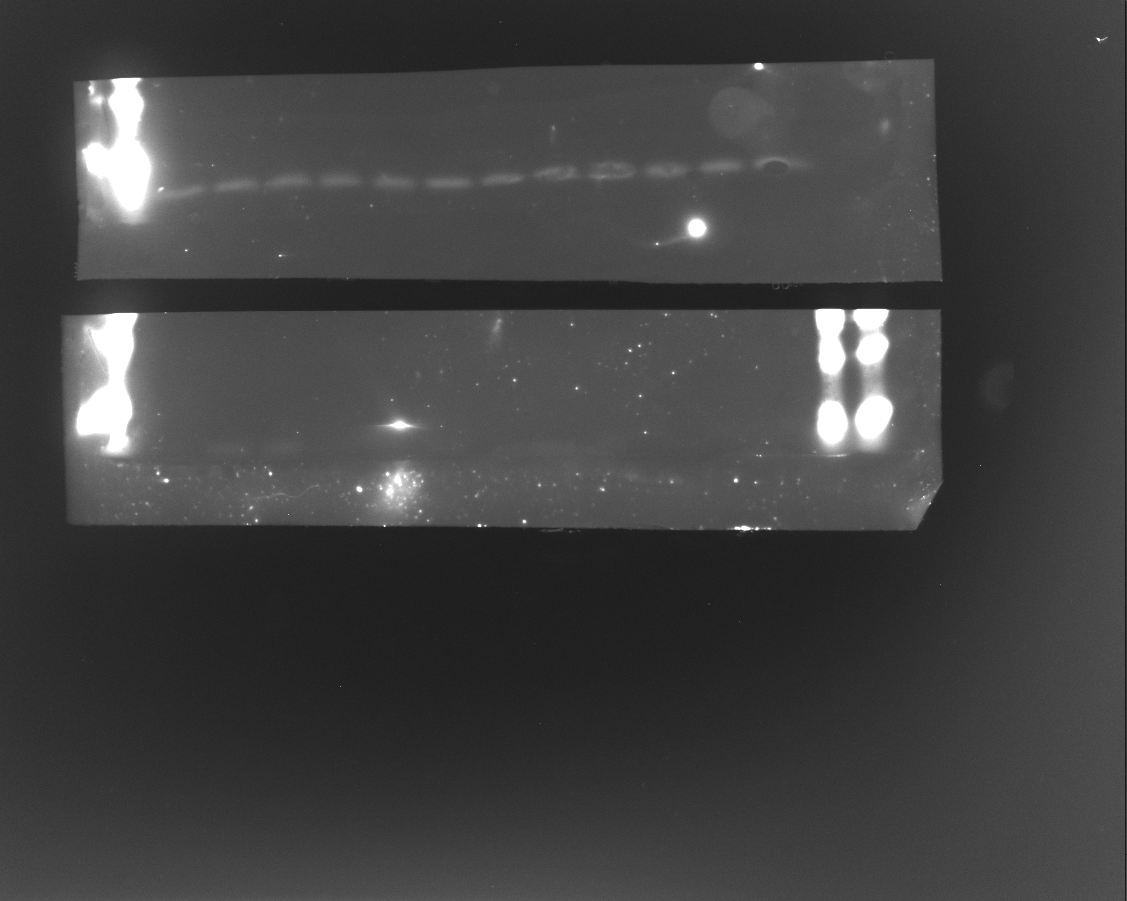

Supplement: Figure 2—figure supplement 1—source data 1. [file elife-102658-fig2-figsupp1-data1.zip › Figure 2-figure suplement 1-source data1 copy/Figure 2-figure suplemment 1-F-1-source data1.tif]

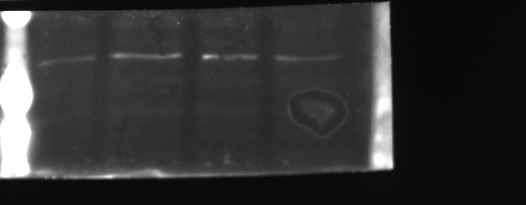

Supplement: Figure 2—figure supplement 1—source data 1. [file elife-102658-fig2-figsupp1-data1.zip › Figure 2-figure suplement 1-source data1 copy/Figure 2-figure suplemment 1-C-11-source data1.tif]

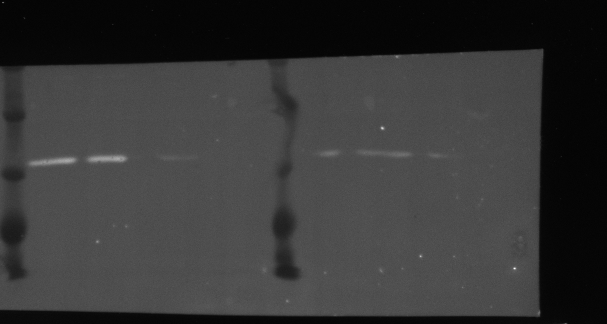

Supplement: Figure 2—figure supplement 1—source data 1. [file elife-102658-fig2-figsupp1-data1.zip › Figure 2-figure suplement 1-source data1 copy/Figure 2-figure suplemment 1-A-B-2-source data1.tif]

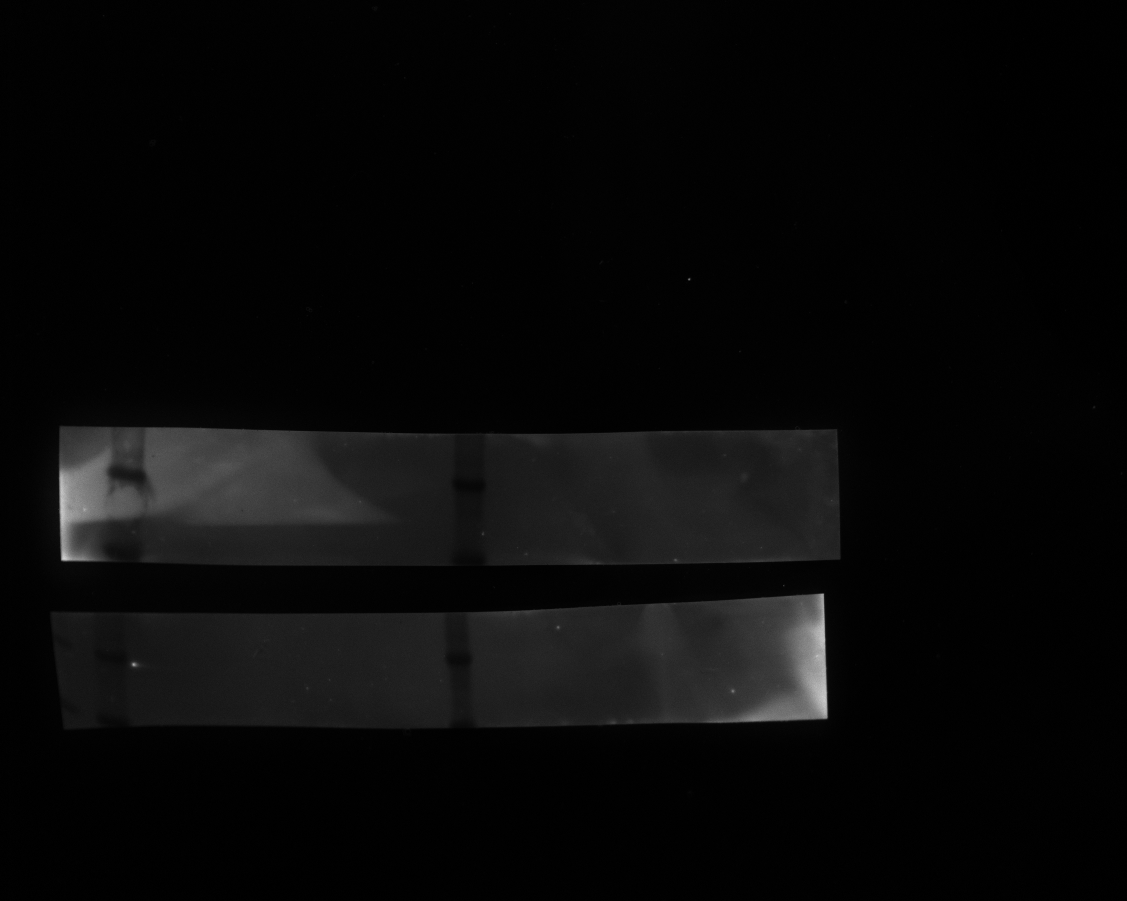

Supplement: Figure 2—figure supplement 1—source data 1. [file elife-102658-fig2-figsupp1-data1.zip › Figure 2-figure suplement 1-source data1 copy/Figure 2-figure suplemment 1-C-15-source data1.tif]

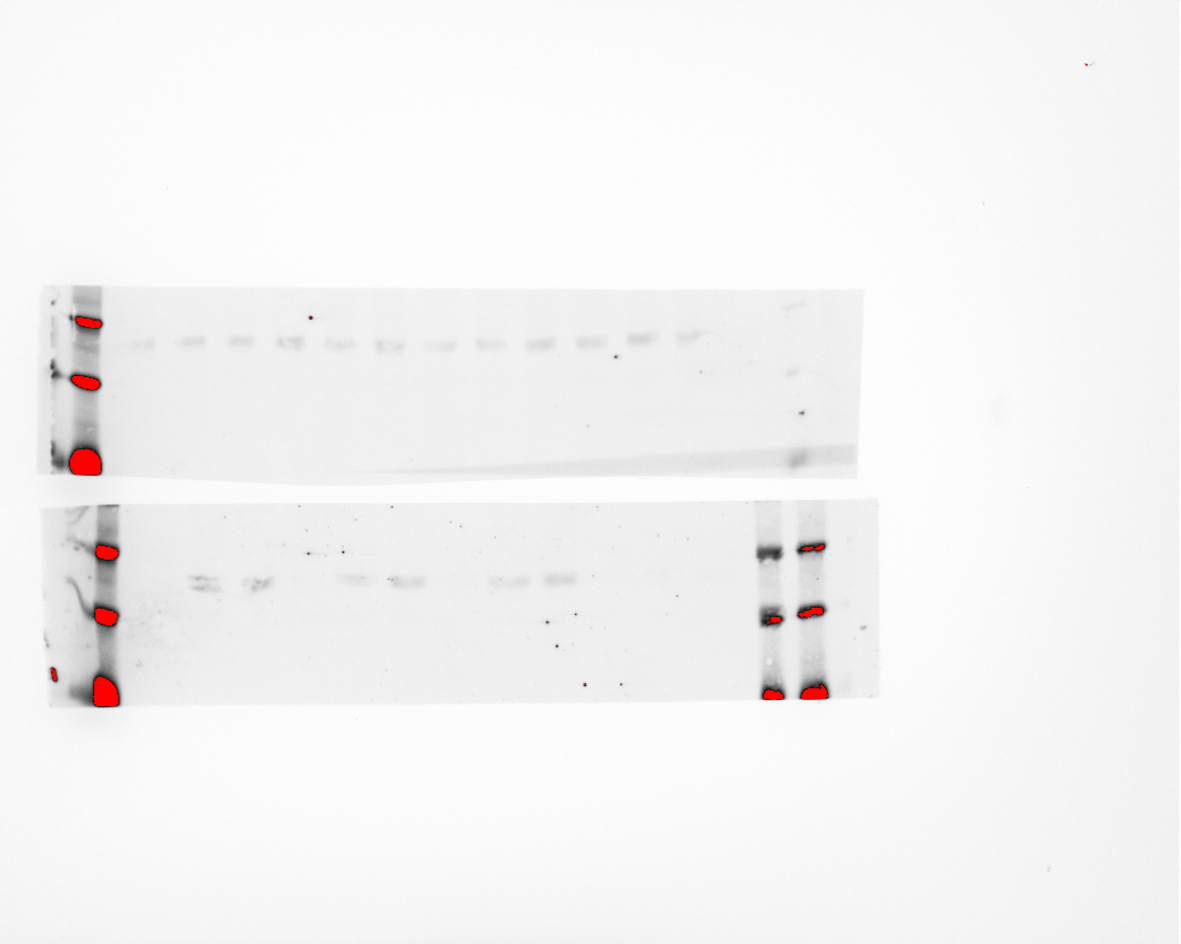

Supplement: Figure 2—figure supplement 1—source data 1. [file elife-102658-fig2-figsupp1-data1.zip › Figure 2-figure suplement 1-source data1 copy/Figure 2-figure suplemment 1-F-5-source data1.tif]

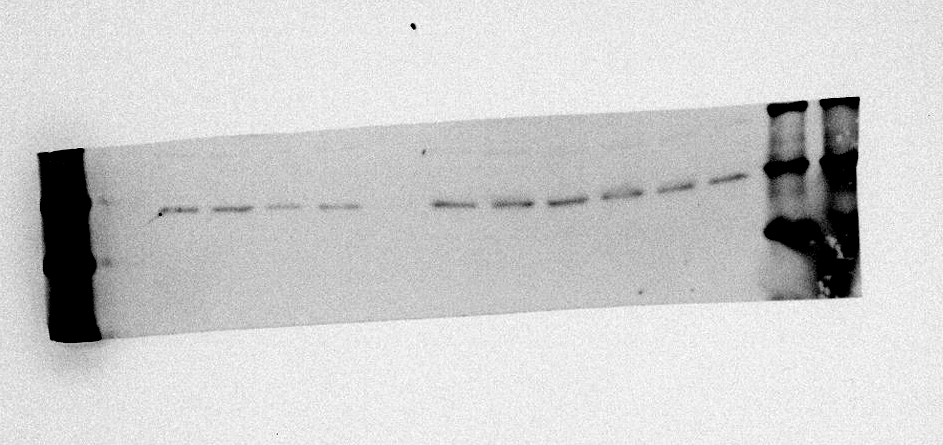

Supplement: Figure 2—figure supplement 1—source data 1. [file elife-102658-fig2-figsupp1-data1.zip › Figure 2-figure suplement 1-source data1 copy/Figure 2-figure suplemment 1-C-4-source data1.tif]

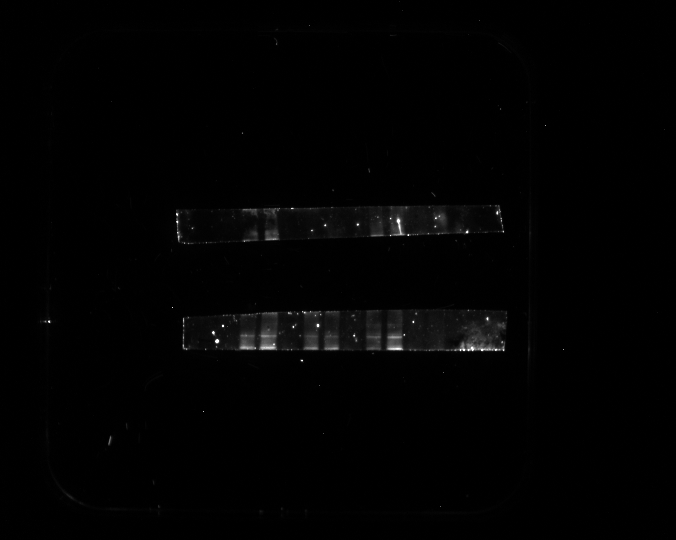

Supplement: Figure 2—figure supplement 1—source data 1. [file elife-102658-fig2-figsupp1-data1.zip › Figure 2-figure suplement 1-source data1 copy/Figure 2-figure suplemment 1-E-1-source data1.tif]

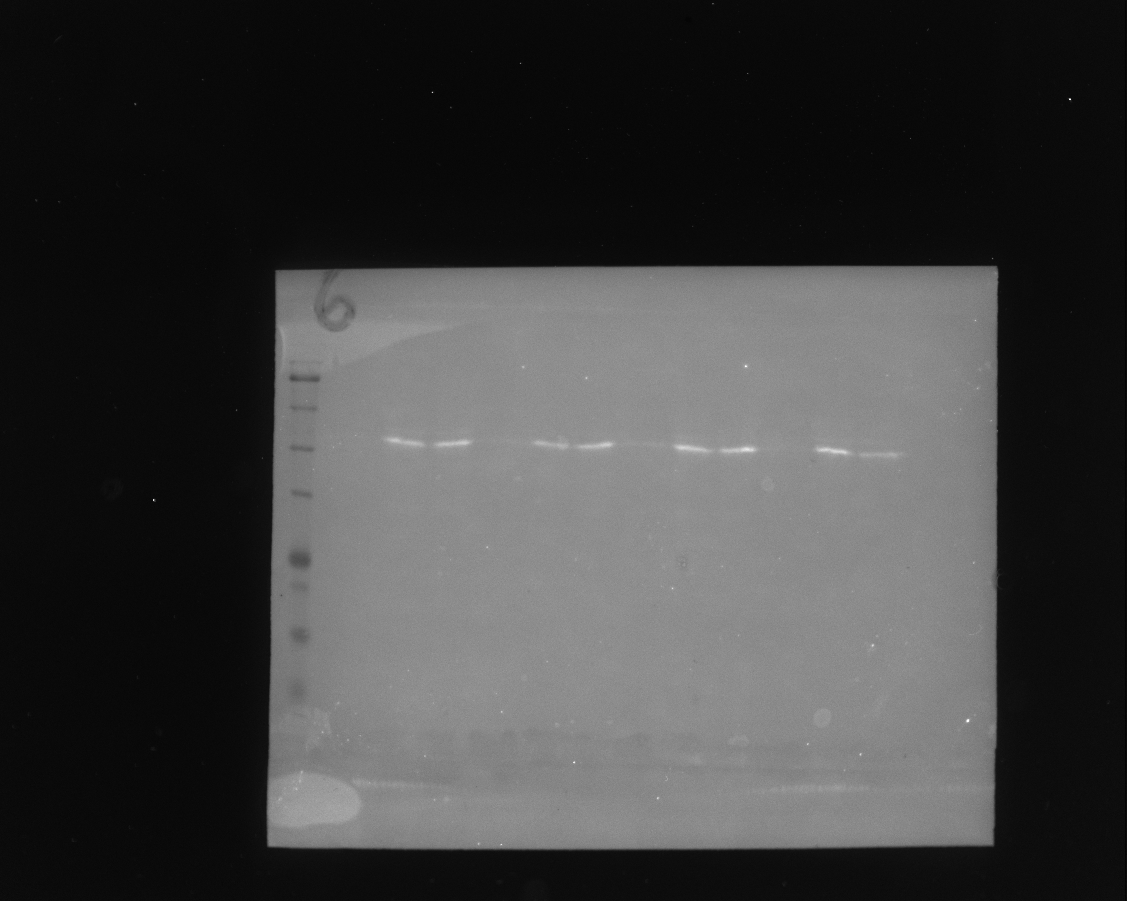

Supplement: Figure 2—figure supplement 1—source data 1. [file elife-102658-fig2-figsupp1-data1.zip › Figure 2-figure suplement 1-source data1 copy/Figure 2-figure suplemment 1-O-3-source data1.tif]

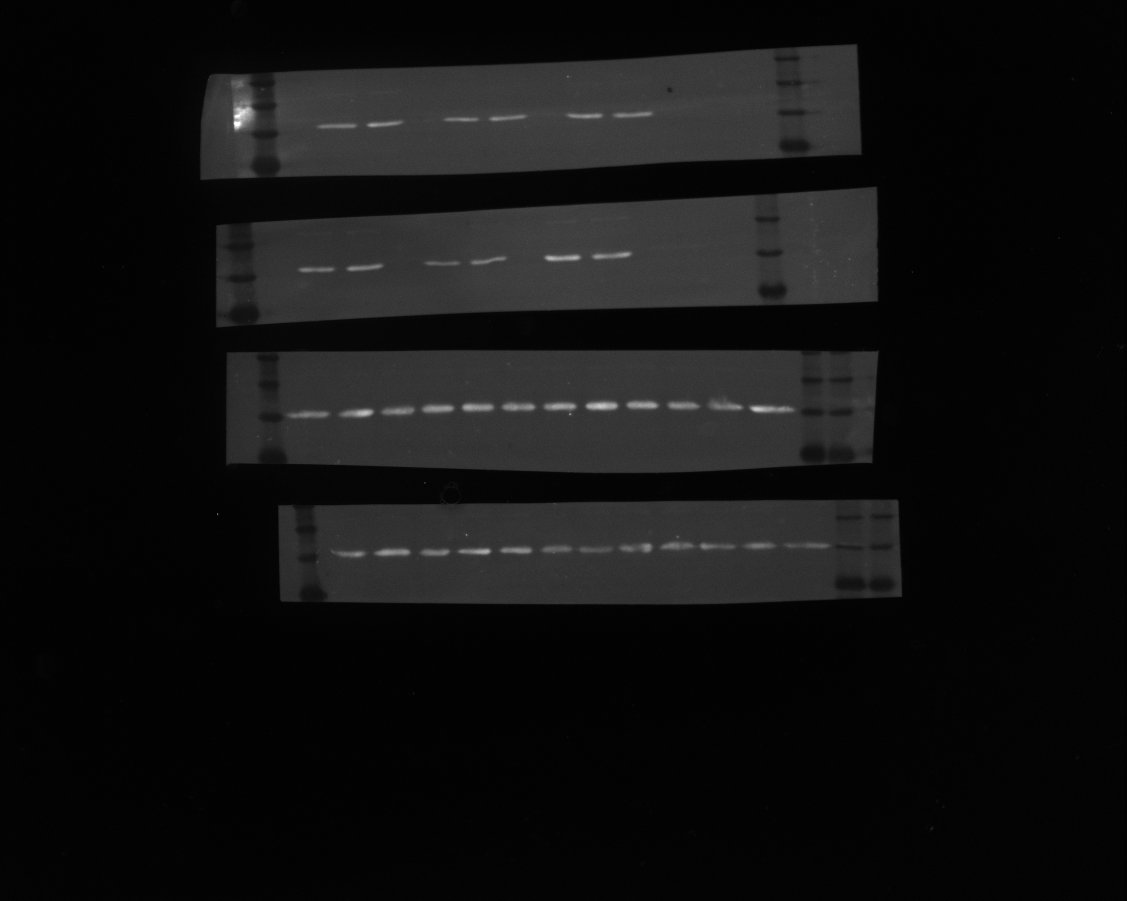

Supplement: Figure 2—figure supplement 1—source data 1. [file elife-102658-fig2-figsupp1-data1.zip › Figure 2-figure suplement 1-source data1 copy/Figure 2-figure suplemment 1-D-1-source data1.tif]

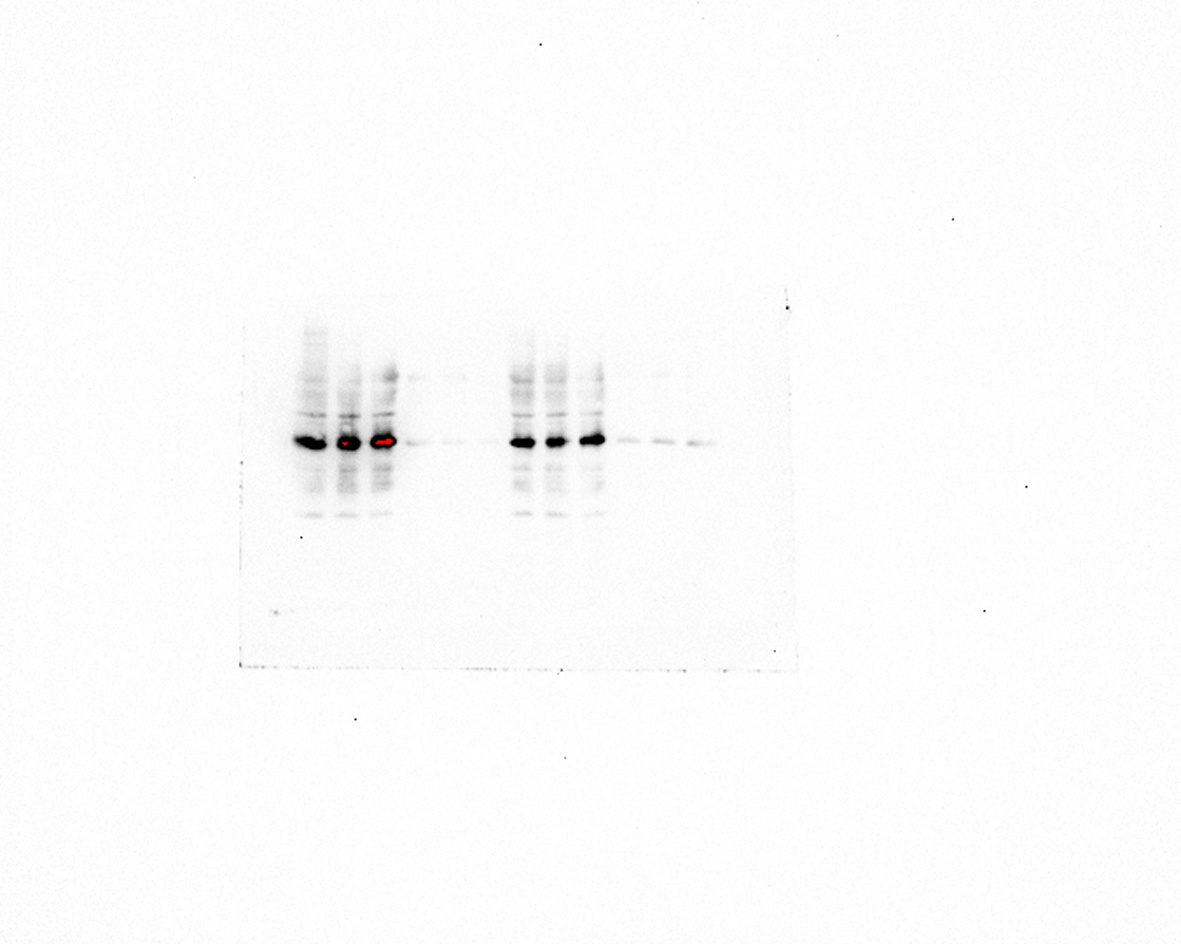

Supplement: Figure 2—figure supplement 1—source data 1. [file elife-102658-fig2-figsupp1-data1.zip › Figure 2-figure suplement 1-source data1 copy/Figure 2-figure suplemment 1-F-2-source data1.tif]

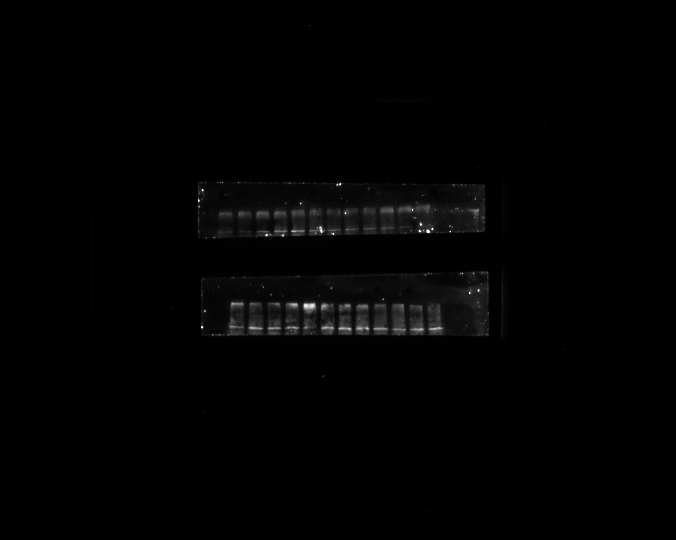

Supplement: Figure 2—figure supplement 1—source data 1. [file elife-102658-fig2-figsupp1-data1.zip › Figure 2-figure suplement 1-source data1 copy/Figure 2-figure suplemment 1-E-6-source data1.tif]

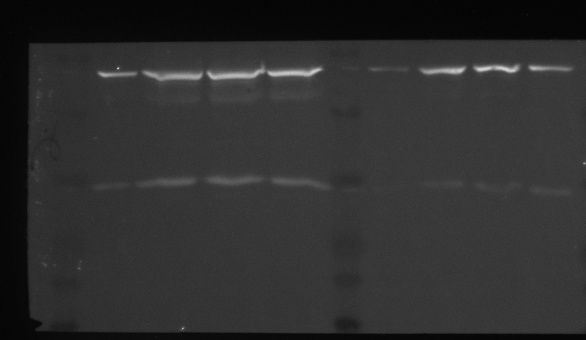

Supplement: Figure 2—figure supplement 1—source data 1. [file elife-102658-fig2-figsupp1-data1.zip › Figure 2-figure suplement 1-source data1 copy/Figure 2-figure suplemment 1-C-3-source data1.tif]

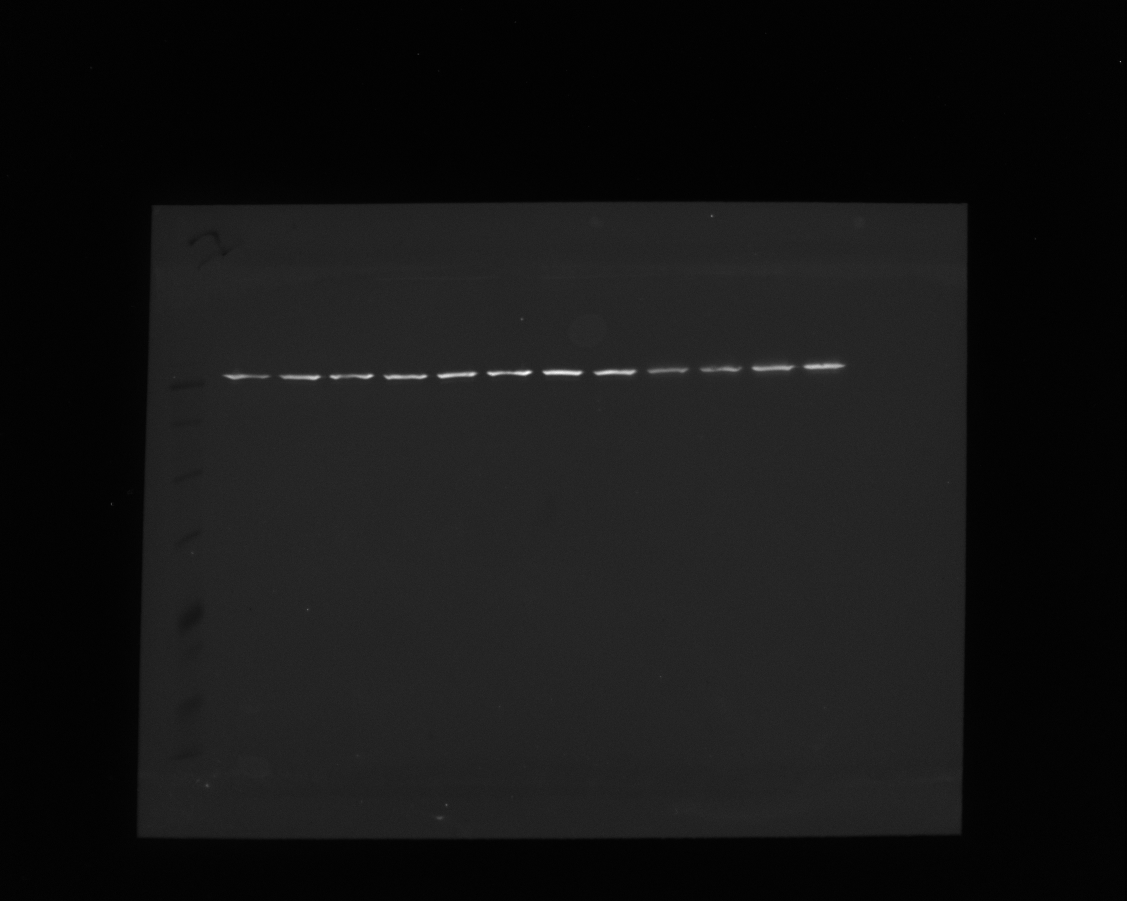

Supplement: Figure 2—figure supplement 1—source data 1. [file elife-102658-fig2-figsupp1-data1.zip › Figure 2-figure suplement 1-source data1 copy/Figure 2-figure suplemment 1-O-4-source data1.tif]

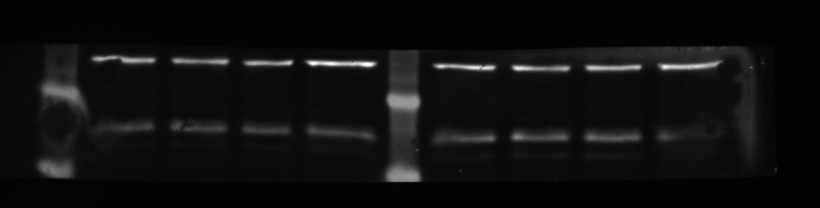

Supplement: Figure 2—figure supplement 1—source data 1. [file elife-102658-fig2-figsupp1-data1.zip › Figure 2-figure suplement 1-source data1 copy/Figure 2-figure suplemment 1-C-12-source data1.tif]

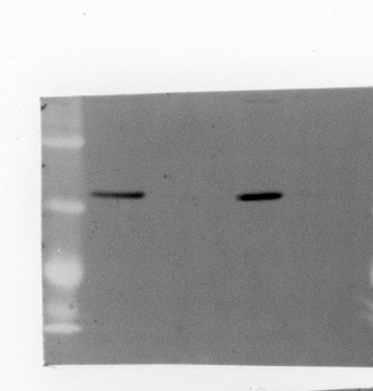

Supplement: Figure 2—figure supplement 1—source data 1. [file elife-102658-fig2-figsupp1-data1.zip › Figure 2-figure suplement 1-source data1 copy/Figure 2-figure suplemment 1-A-B-1-source data1.tif]

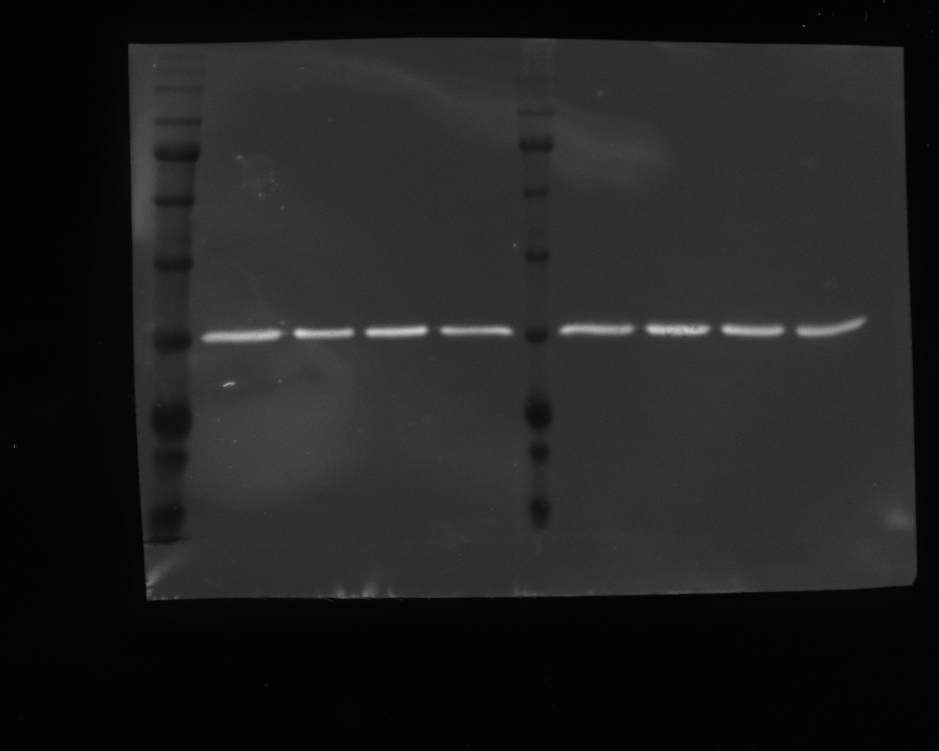

Supplement: Figure 2—figure supplement 1—source data 1. [file elife-102658-fig2-figsupp1-data1.zip › Figure 2-figure suplement 1-source data1 copy/Figure 2-figure suplemment 1-C-8-source data1.tif]

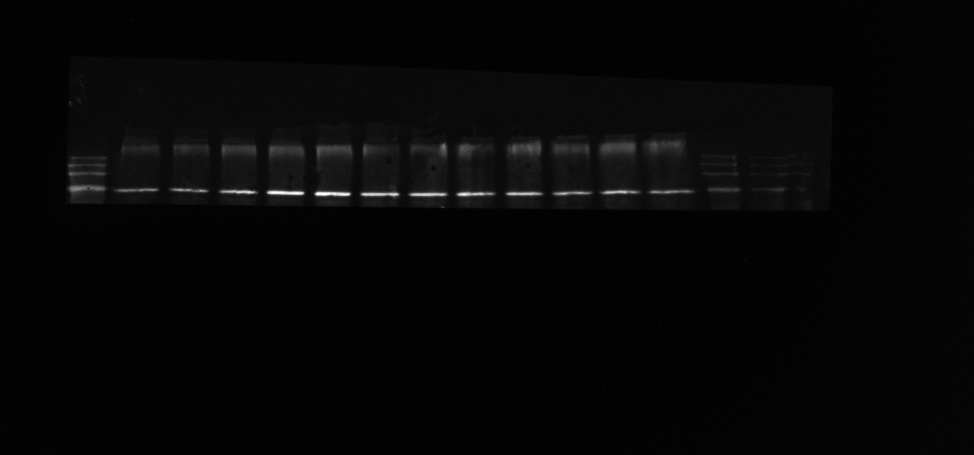

Supplement: Figure 2—figure supplement 1—source data 1. [file elife-102658-fig2-figsupp1-data1.zip › Figure 2-figure suplement 1-source data1 copy/Figure 2-figure suplemment 1-E-9-source data1.tif]

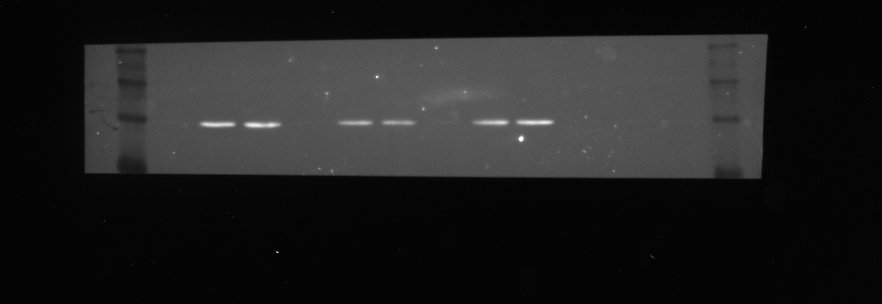

Supplement: Figure 2—figure supplement 1—source data 1. [file elife-102658-fig2-figsupp1-data1.zip › Figure 2-figure suplement 1-source data1 copy/Figure 2-figure suplemment 1-E-2-source data1.tif]

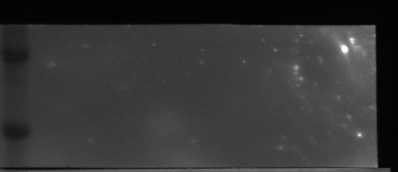

Supplement: Figure 2—figure supplement 1—source data 1. [file elife-102658-fig2-figsupp1-data1.zip › Figure 2-figure suplement 1-source data1 copy/Figure 2-figure suplemment 1-C-7-source data1.tif]

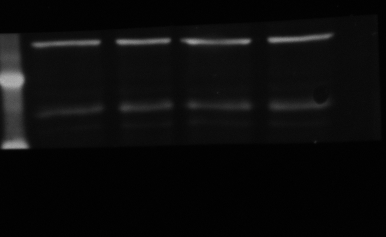

Supplement: Figure 2—figure supplement 1—source data 1. [file elife-102658-fig2-figsupp1-data1.zip › Figure 2-figure suplement 1-source data1 copy/Figure 2-figure suplemment 1-C-13-source data1.tif]

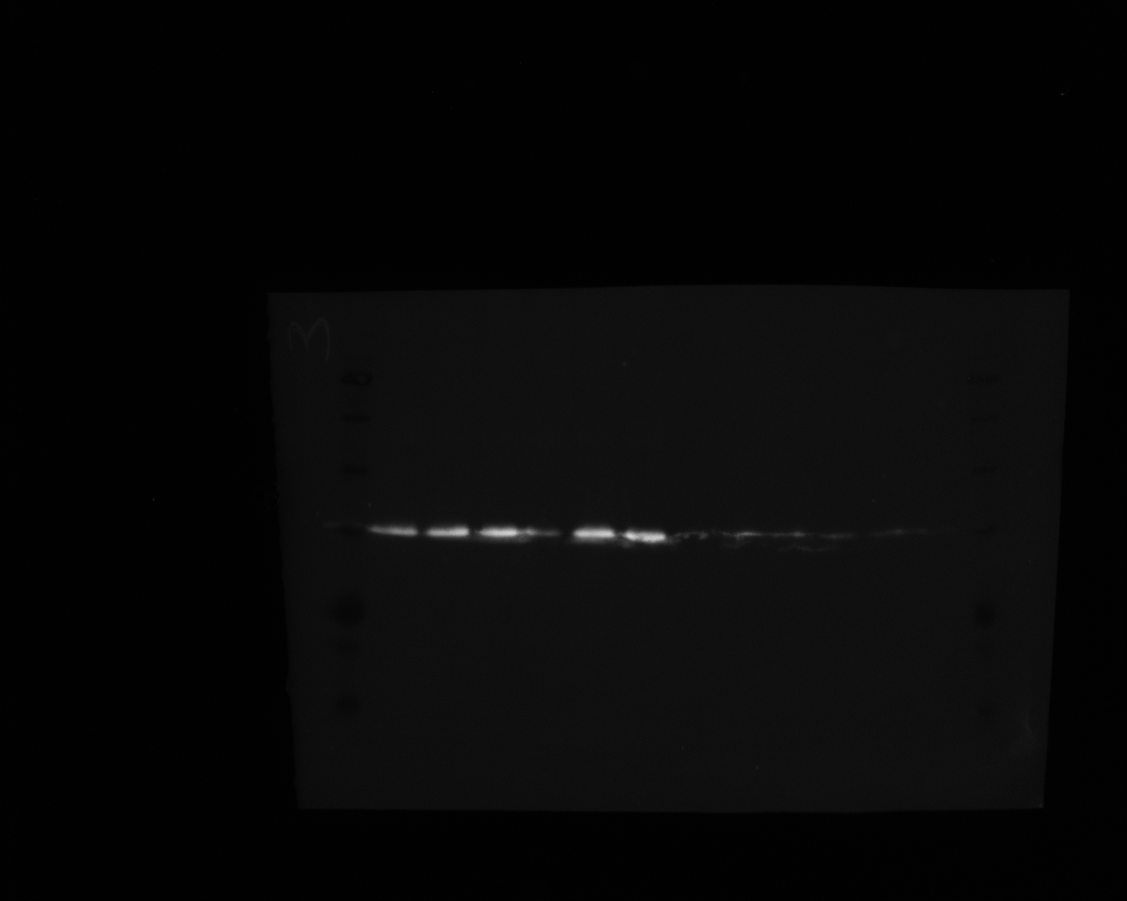

Supplement: Figure 2—figure supplement 1—source data 1. [file elife-102658-fig2-figsupp1-data1.zip › Figure 2-figure suplement 1-source data1 copy/Figure 2-figure suplemment 1-F-3-source data1.tif]

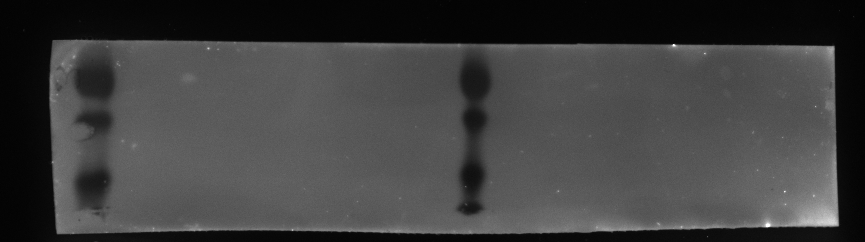

Supplement: Figure 2—figure supplement 1—source data 1. [file elife-102658-fig2-figsupp1-data1.zip › Figure 2-figure suplement 1-source data1 copy/Figure 2-figure suplemment 1-C-2-source data1.tif]

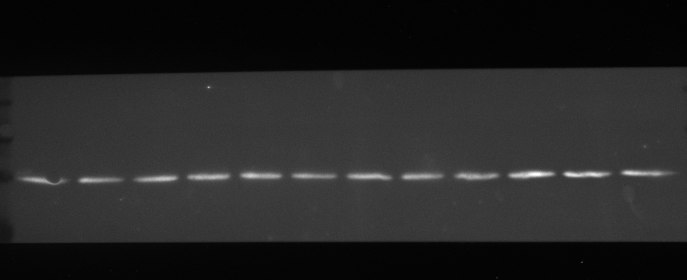

Supplement: Figure 2—figure supplement 1—source data 1. [file elife-102658-fig2-figsupp1-data1.zip › Figure 2-figure suplement 1-source data1 copy/Figure 2-figure suplemment 1-E-7-source data1.tif]

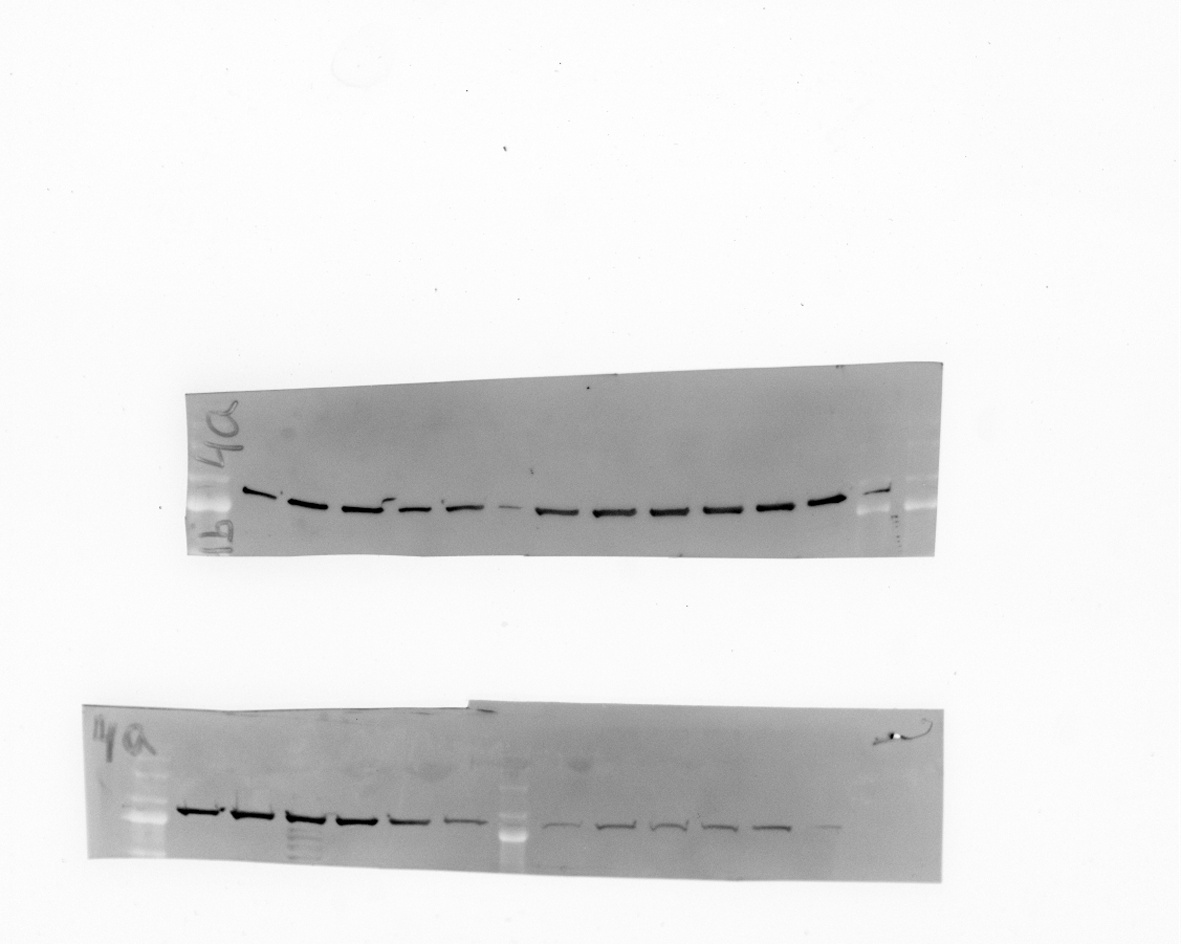

Supplement: Figure 2—figure supplement 1—source data 1. [file elife-102658-fig2-figsupp1-data1.zip › Figure 2-figure suplement 1-source data1 copy/Figure 2-figure suplemment 1-C-9-source data1.tif]

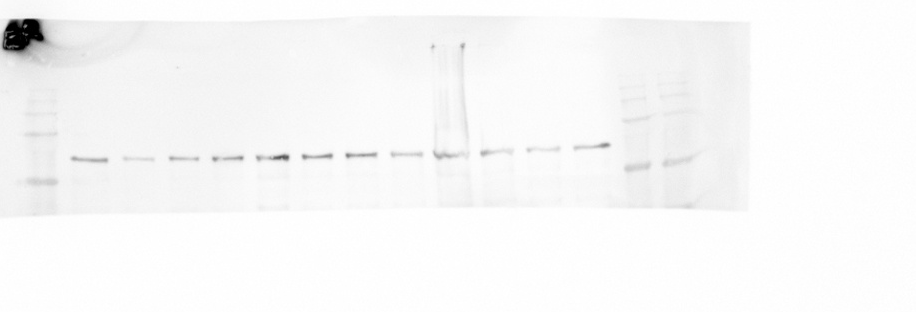

Supplement: Figure 2—figure supplement 1—source data 1. [file elife-102658-fig2-figsupp1-data1.zip › Figure 2-figure suplement 1-source data1 copy/Figure 2-figure suplemment 1-E-8-source data1.tif]

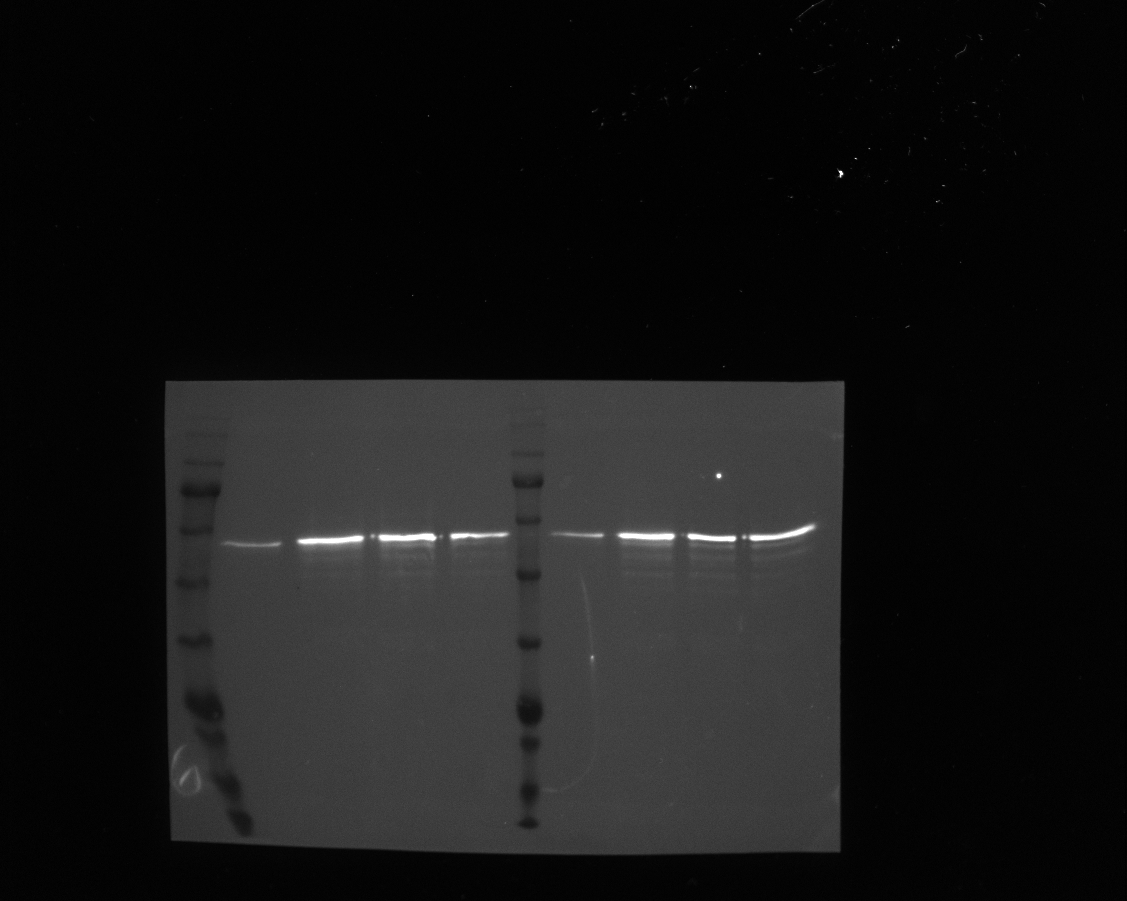

Supplement: Figure 2—figure supplement 1—source data 1. [file elife-102658-fig2-figsupp1-data1.zip › Figure 2-figure suplement 1-source data1 copy/Figure 2-figure suplemment 1-C-6-source data1.tif]

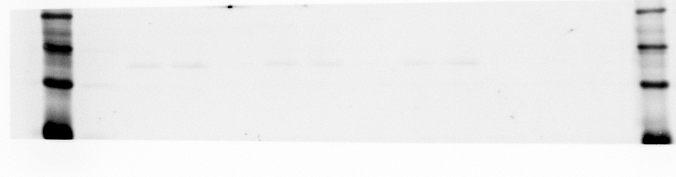

Supplement: Figure 2—figure supplement 1—source data 1. [file elife-102658-fig2-figsupp1-data1.zip › Figure 2-figure suplement 1-source data1 copy/Figure 2-figure suplemment 1-E-3-source data1.tif]

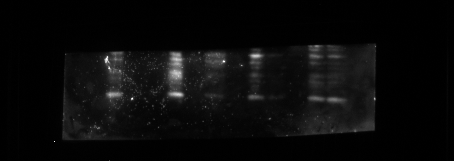

Supplement: Figure 2—figure supplement 1—source data 1. [file elife-102658-fig2-figsupp1-data1.zip › Figure 2-figure suplement 1-source data1 copy/Figure 2-figure suplemment 1-O-1-source data1.tif]

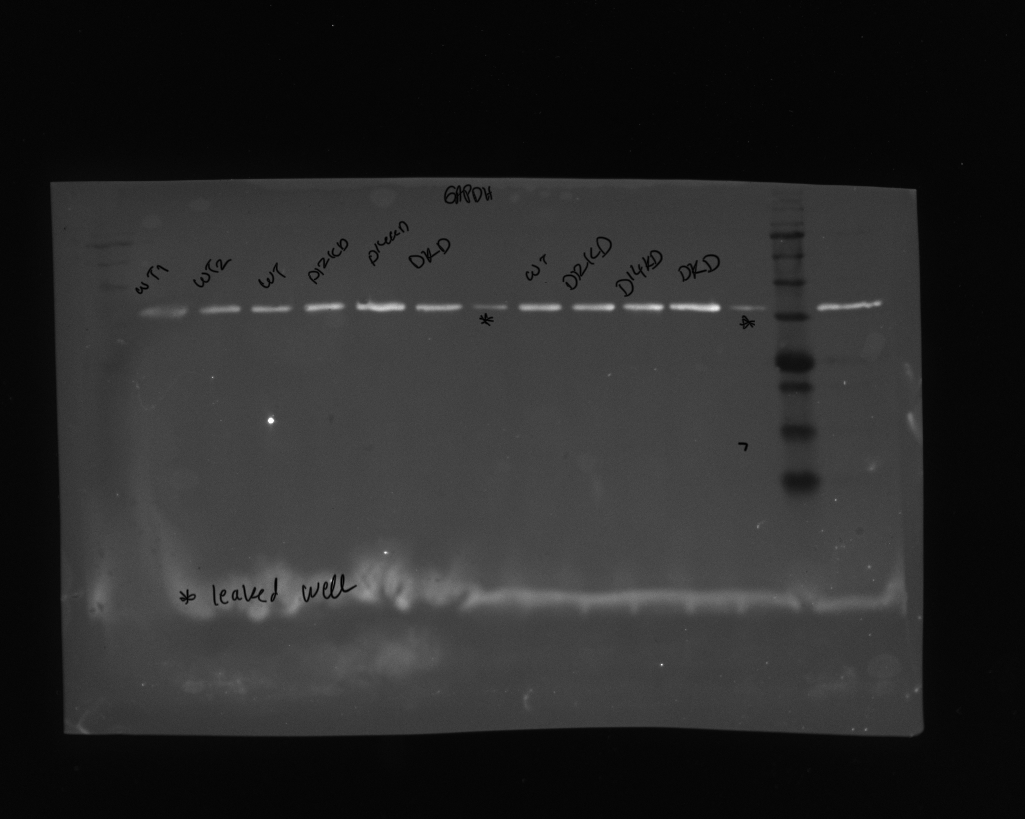

Supplement: Figure 2—figure supplement 1—source data 2. [file elife-102658-fig2-figsupp1-data2.zip › Figure 2-figure suplement 1-source data1/Figure 2-figure suplemment 1-A-B-3-source data1.tif]

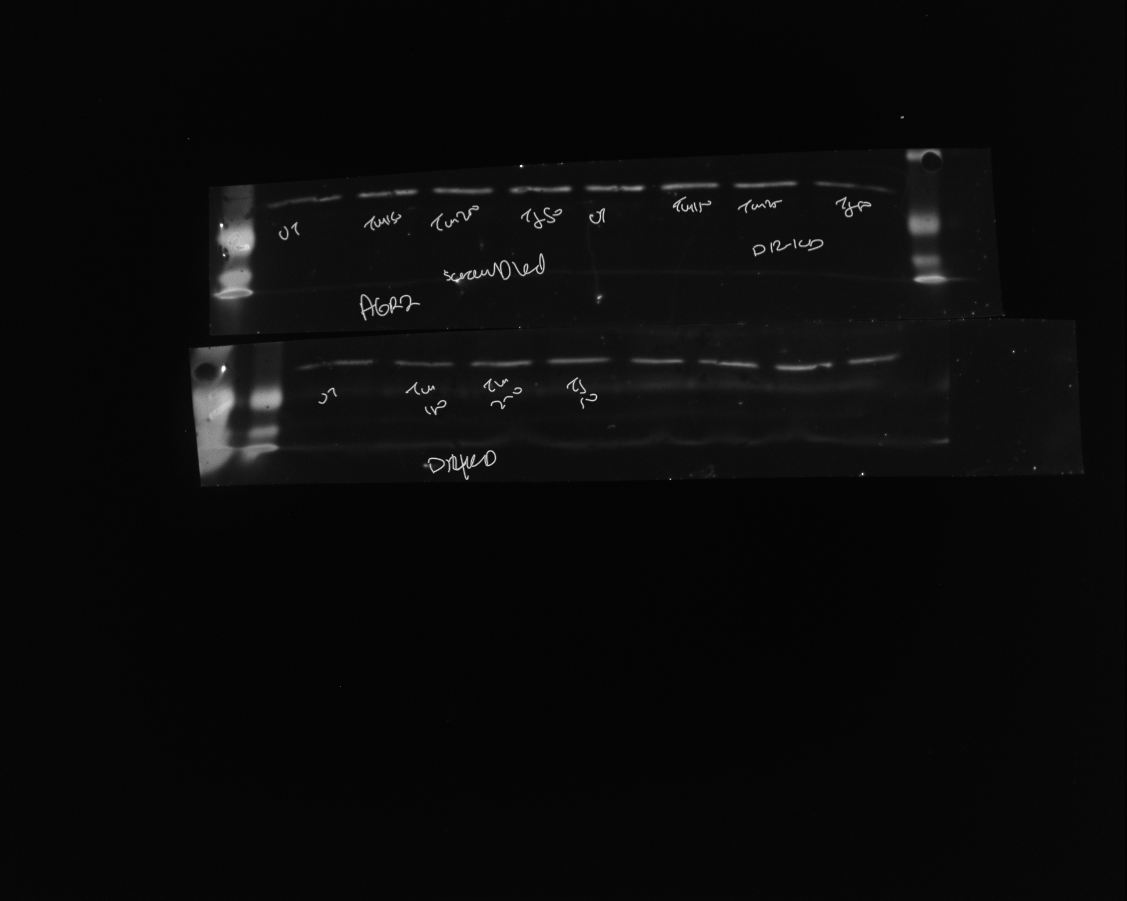

Supplement: Figure 2—figure supplement 1—source data 2. [file elife-102658-fig2-figsupp1-data2.zip › Figure 2-figure suplement 1-source data1/Figure 2-figure suplemment 1-C-10-source data1.tif]

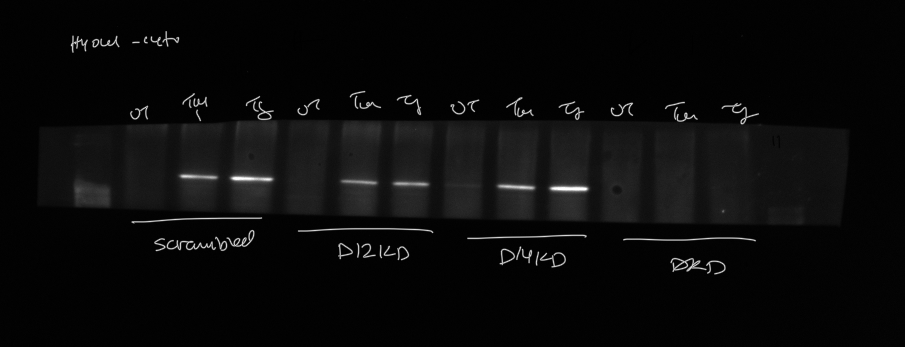

Supplement: Figure 2—figure supplement 1—source data 2. [file elife-102658-fig2-figsupp1-data2.zip › Figure 2-figure suplement 1-source data1/Figure 2-figure suplemment 1-E-4-source data1.tif]

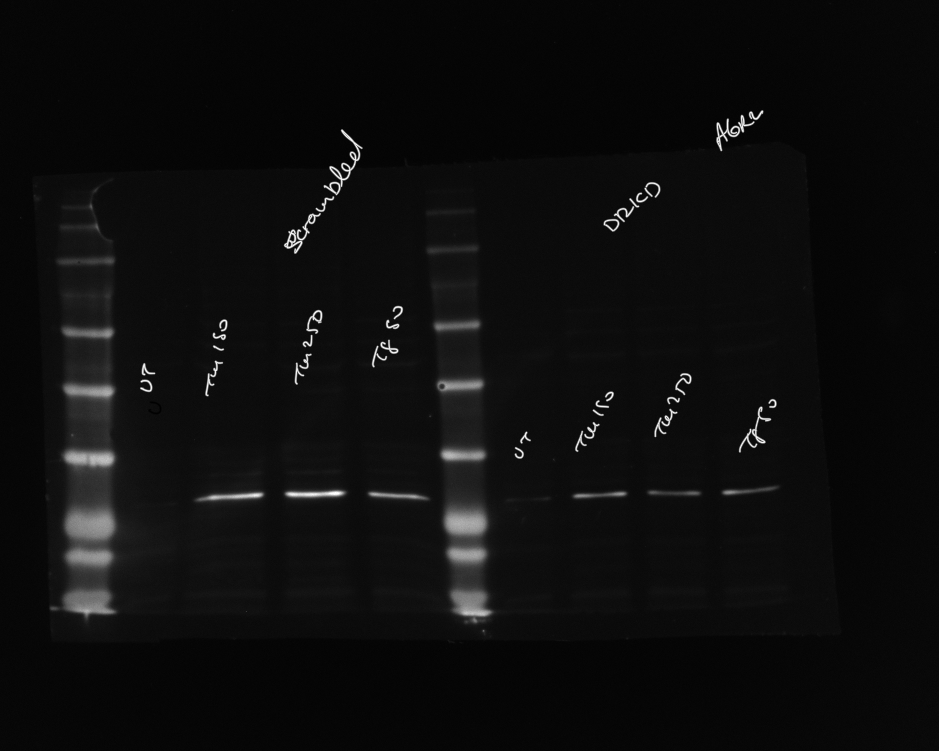

Supplement: Figure 2—figure supplement 1—source data 2. [file elife-102658-fig2-figsupp1-data2.zip › Figure 2-figure suplement 1-source data1/Figure 2-figure suplemment 1-C-1-source data1.tif]

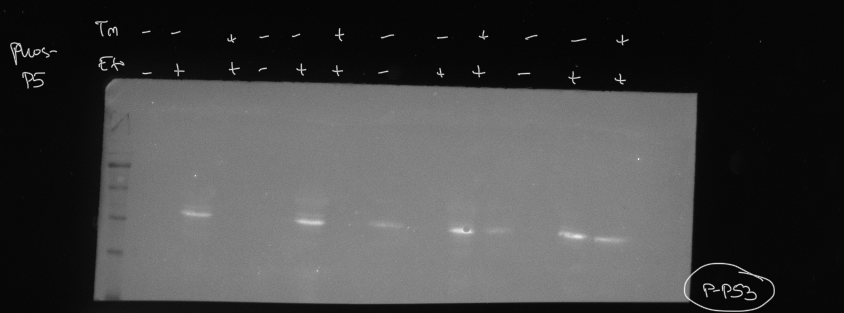

Supplement: Figure 2—figure supplement 1—source data 2. [file elife-102658-fig2-figsupp1-data2.zip › Figure 2-figure suplement 1-source data1/Figure 2-figure suplemment 1-O-2-source data1.tif]

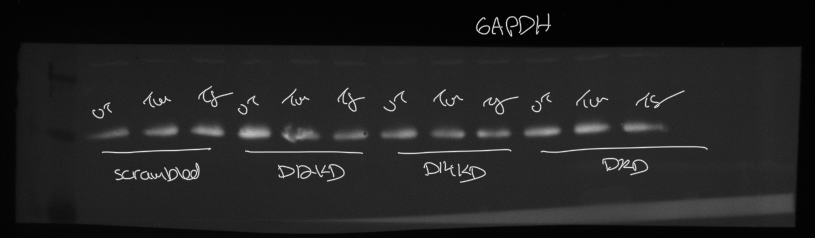

Supplement: Figure 2—figure supplement 1—source data 2. [file elife-102658-fig2-figsupp1-data2.zip › Figure 2-figure suplement 1-source data1/Figure 2-figure suplemment 1-F-4-source data1.tif]

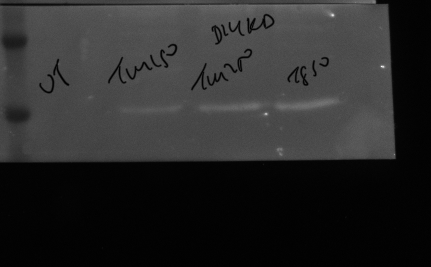

Supplement: Figure 2—figure supplement 1—source data 2. [file elife-102658-fig2-figsupp1-data2.zip › Figure 2-figure suplement 1-source data1/Figure 2-figure suplemment 1-C-5-source data1.tif]

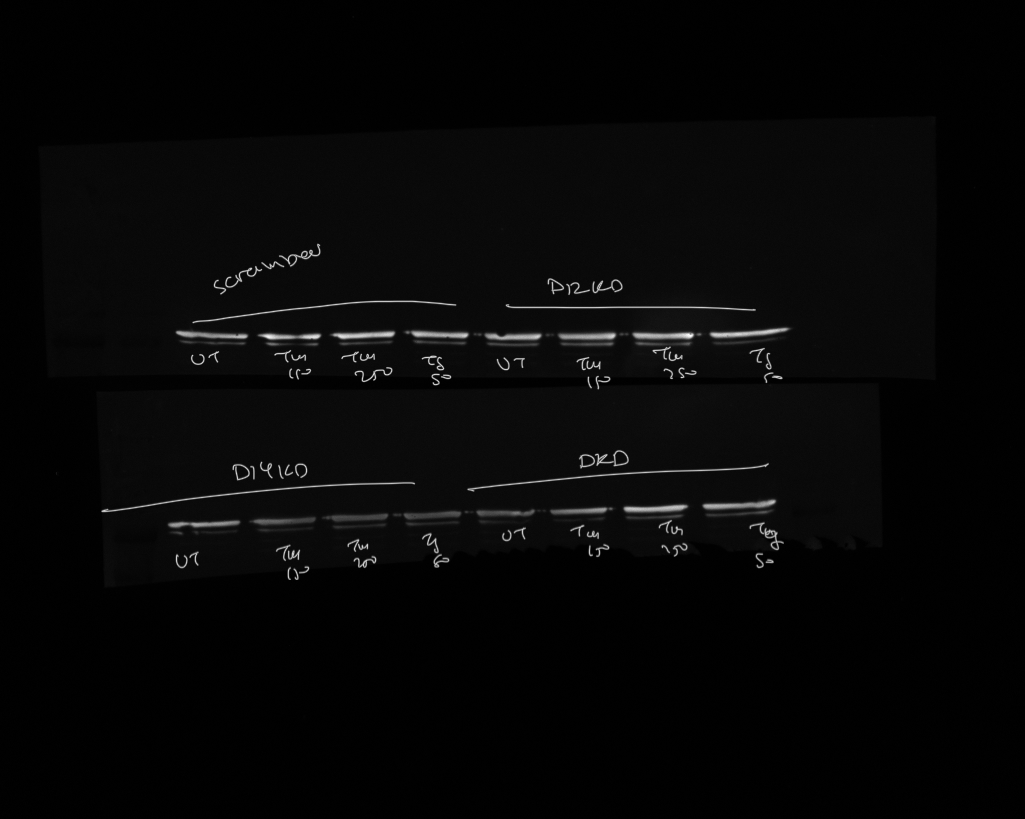

Supplement: Figure 2—figure supplement 1—source data 2. [file elife-102658-fig2-figsupp1-data2.zip › Figure 2-figure suplement 1-source data1/Figure 2-figure suplemment 1-C-14-source data1.tif]

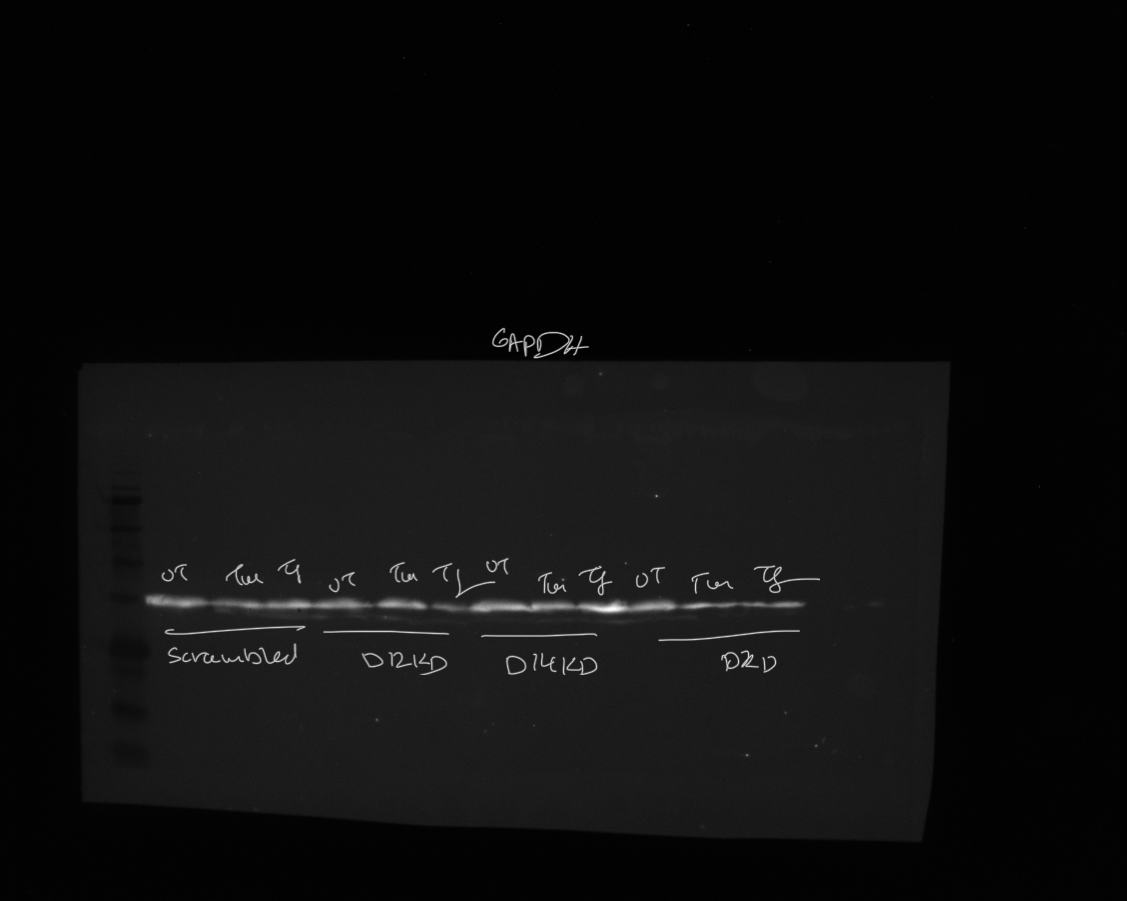

Supplement: Figure 2—figure supplement 1—source data 2. [file elife-102658-fig2-figsupp1-data2.zip › Figure 2-figure suplement 1-source data1/Figure 2-figure suplemment 1-E-5-source data1.tif]

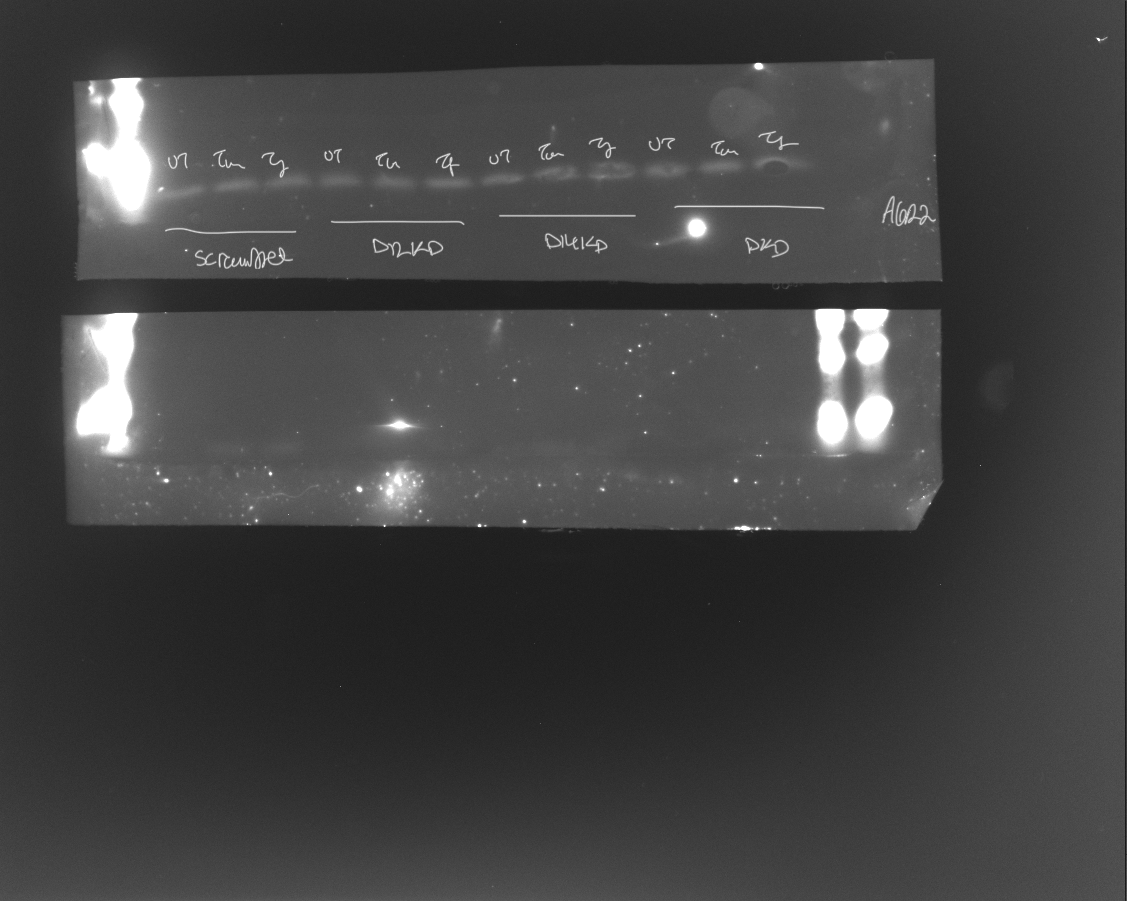

Supplement: Figure 2—figure supplement 1—source data 2. [file elife-102658-fig2-figsupp1-data2.zip › Figure 2-figure suplement 1-source data1/Figure 2-figure suplemment 1-F-1-source data1.tif]

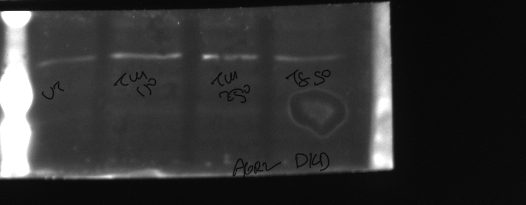

Supplement: Figure 2—figure supplement 1—source data 2. [file elife-102658-fig2-figsupp1-data2.zip › Figure 2-figure suplement 1-source data1/Figure 2-figure suplemment 1-C-11-source data1.tif]

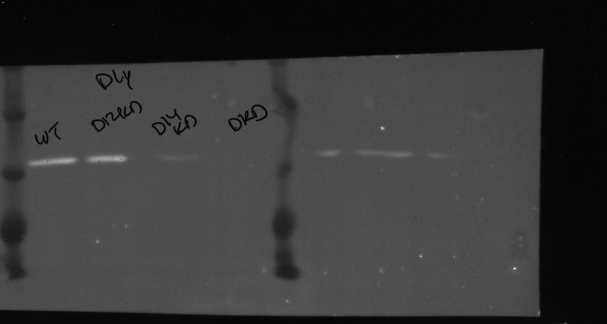

Supplement: Figure 2—figure supplement 1—source data 2. [file elife-102658-fig2-figsupp1-data2.zip › Figure 2-figure suplement 1-source data1/Figure 2-figure suplemment 1-A-B-2-source data1.tif]

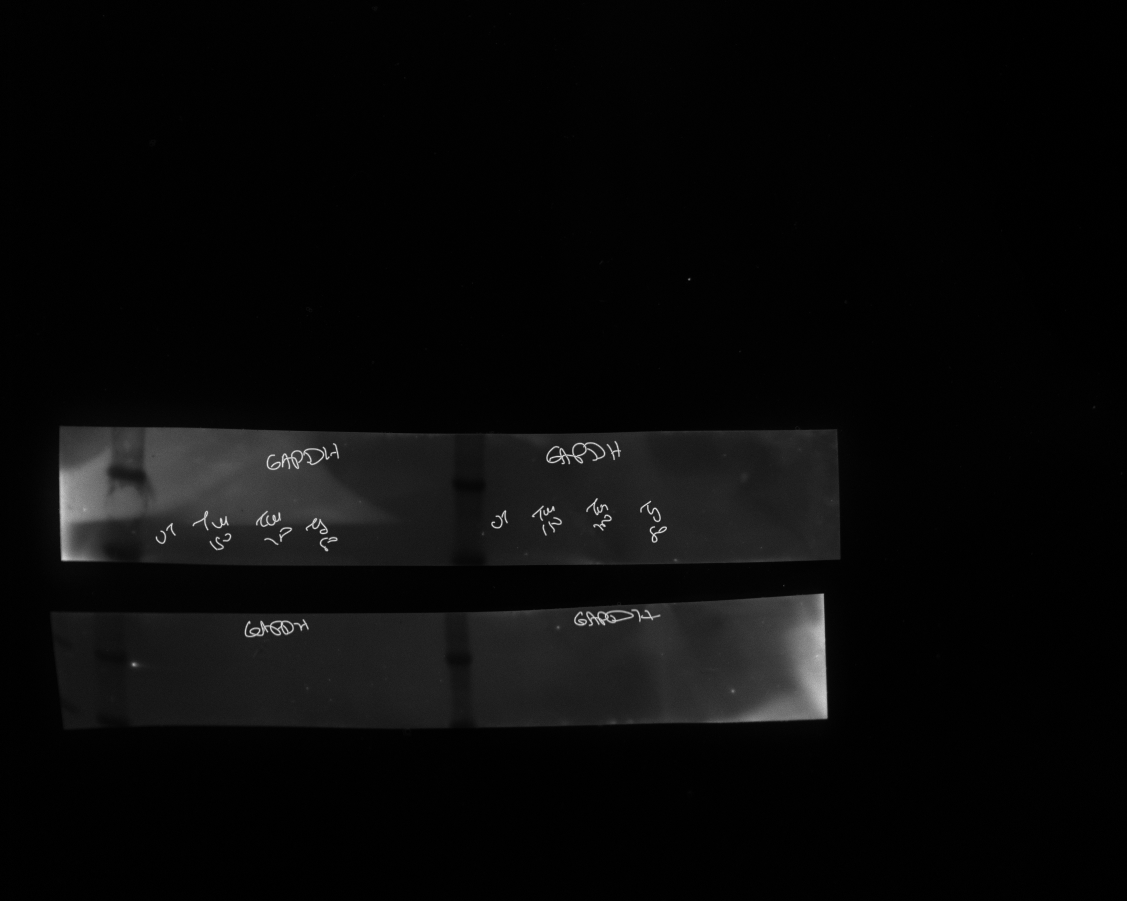

Supplement: Figure 2—figure supplement 1—source data 2. [file elife-102658-fig2-figsupp1-data2.zip › Figure 2-figure suplement 1-source data1/Figure 2-figure suplemment 1-C-15-source data1.tif]

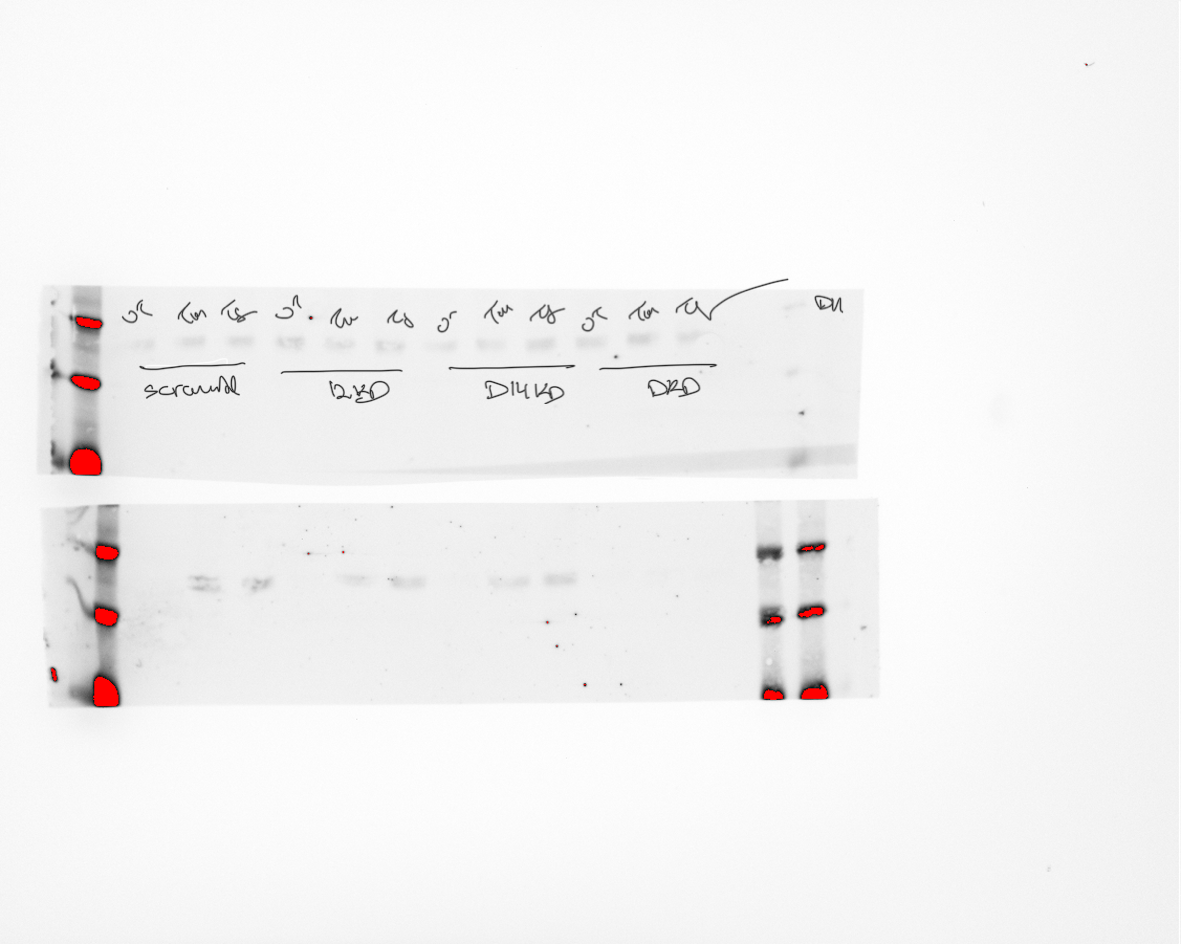

Supplement: Figure 2—figure supplement 1—source data 2. [file elife-102658-fig2-figsupp1-data2.zip › Figure 2-figure suplement 1-source data1/Figure 2-figure suplemment 1-F-5-source data1.tif]

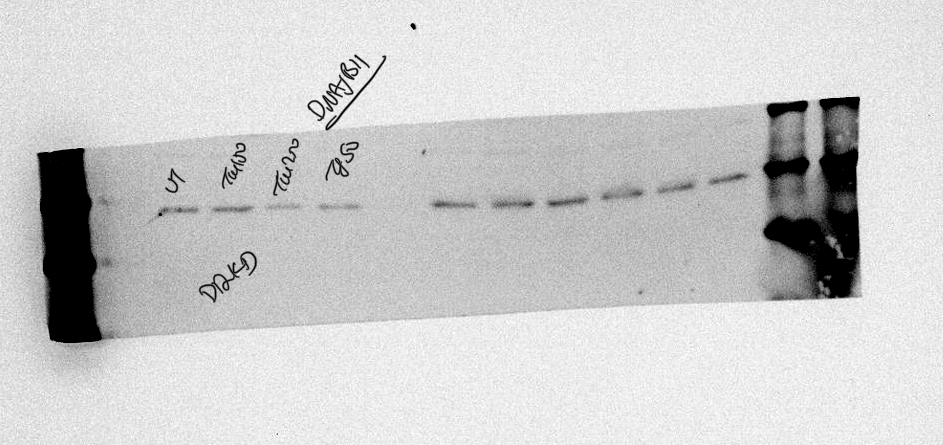

Supplement: Figure 2—figure supplement 1—source data 2. [file elife-102658-fig2-figsupp1-data2.zip › Figure 2-figure suplement 1-source data1/Figure 2-figure suplemment 1-C-4-source data1.tif]

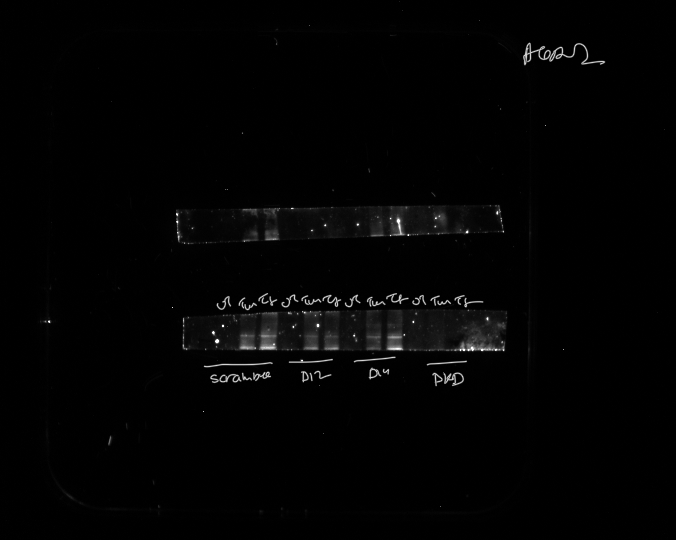

Supplement: Figure 2—figure supplement 1—source data 2. [file elife-102658-fig2-figsupp1-data2.zip › Figure 2-figure suplement 1-source data1/Figure 2-figure suplemment 1-E-1-source data1.tif]

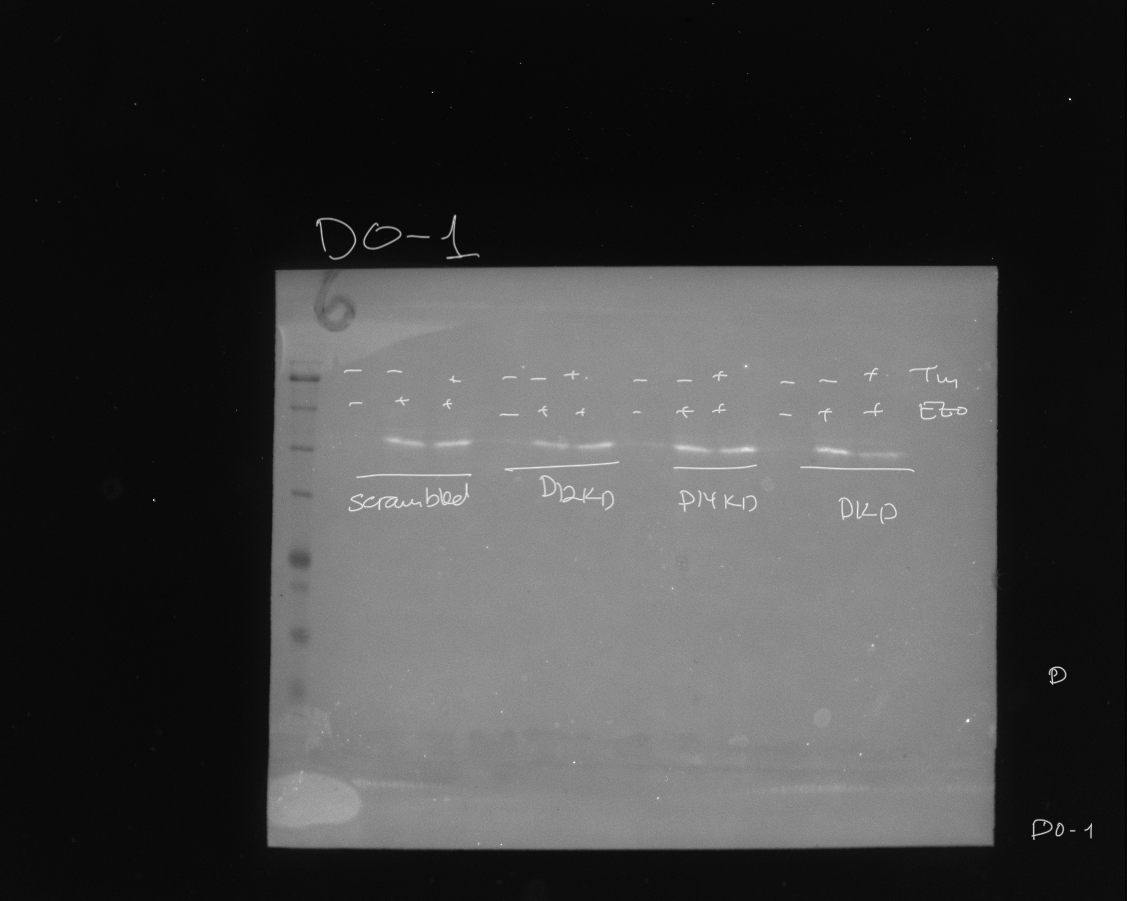

Supplement: Figure 2—figure supplement 1—source data 2. [file elife-102658-fig2-figsupp1-data2.zip › Figure 2-figure suplement 1-source data1/Figure 2-figure suplemment 1-O-3-source data1.tif]

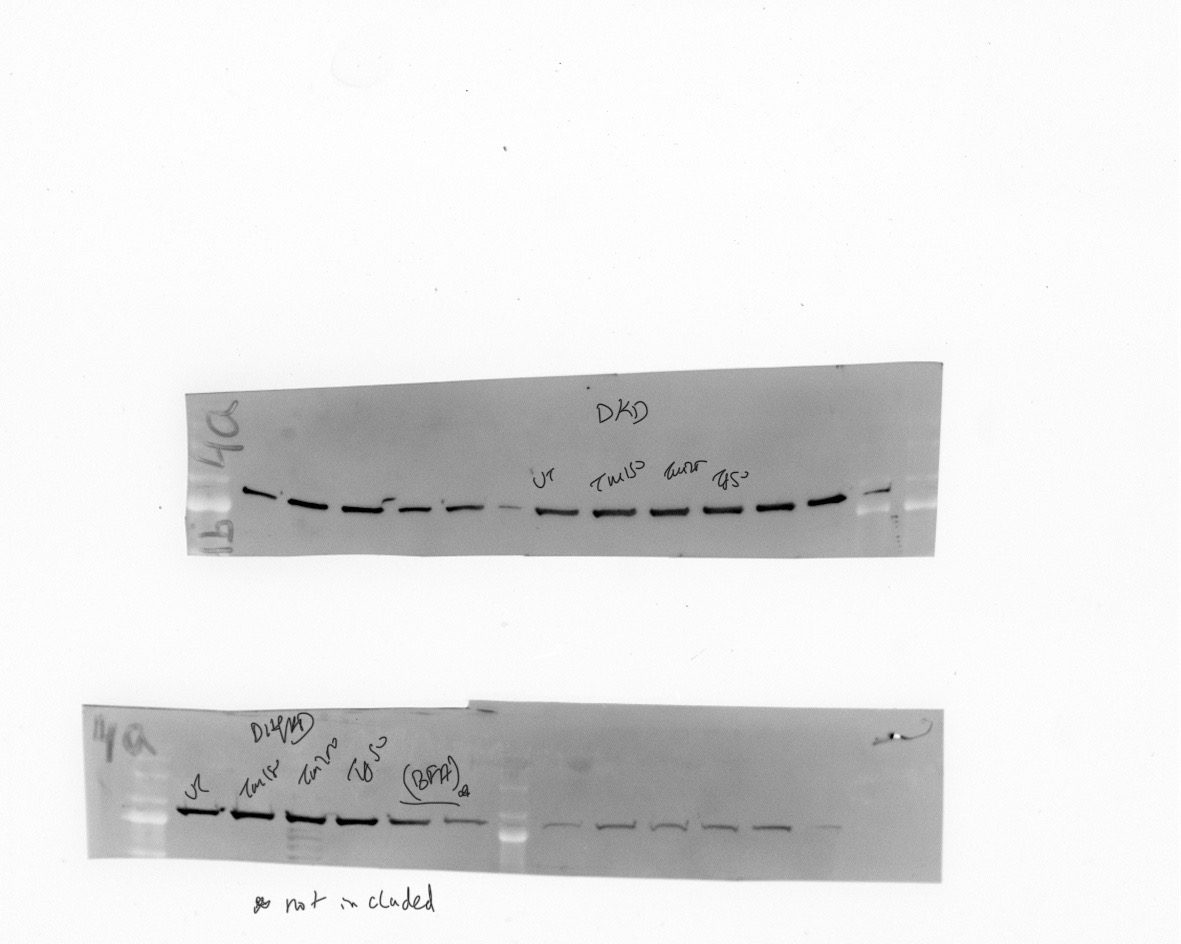

Supplement: Figure 2—figure supplement 1—source data 2. [file elife-102658-fig2-figsupp1-data2.zip › Figure 2-figure suplement 1-source data1/Figure 2-figure suplemment 1-C-9-source data1 copy.jpeg]

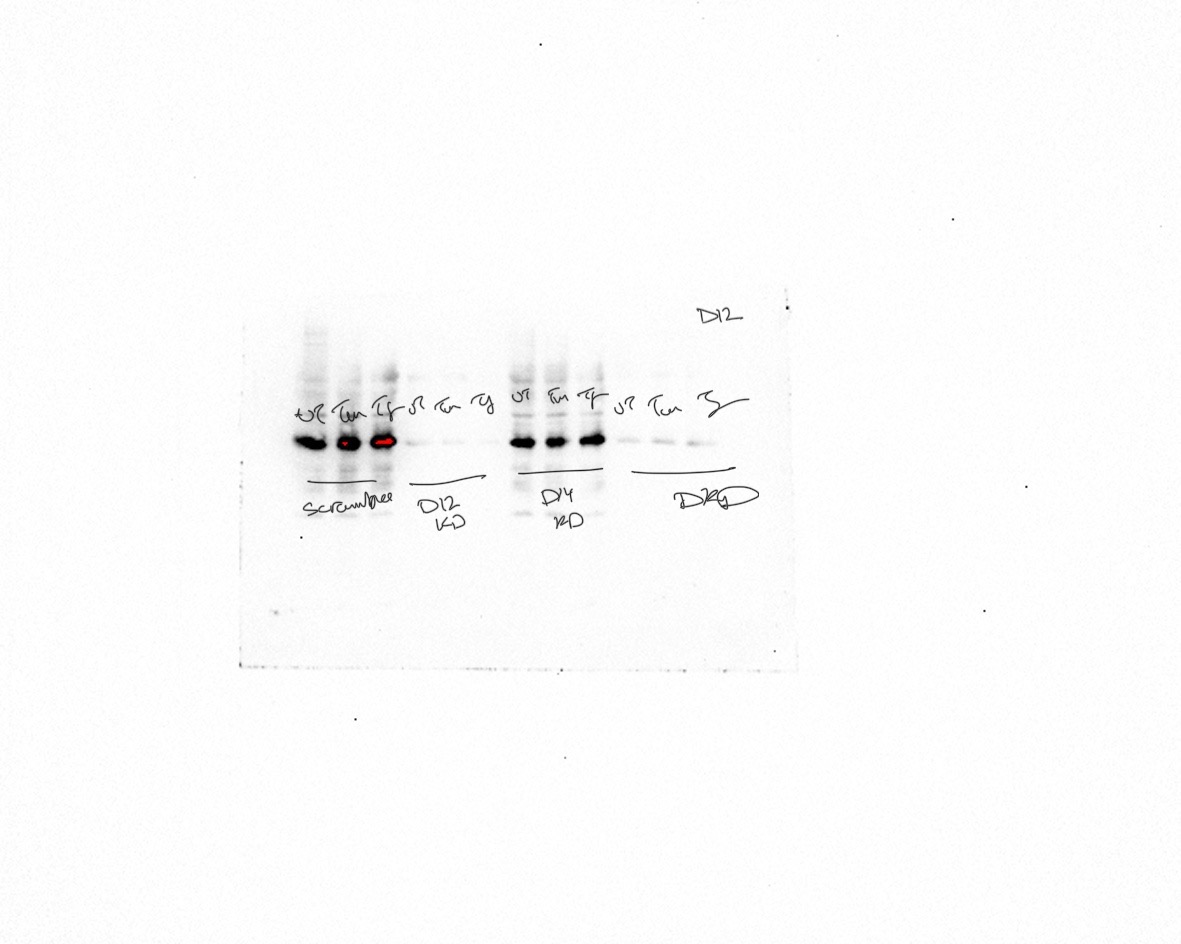

Supplement: Figure 2—figure supplement 1—source data 2. [file elife-102658-fig2-figsupp1-data2.zip › Figure 2-figure suplement 1-source data1/Figure 2-figure suplemment 1-F-2-source data1 copy.jpeg]

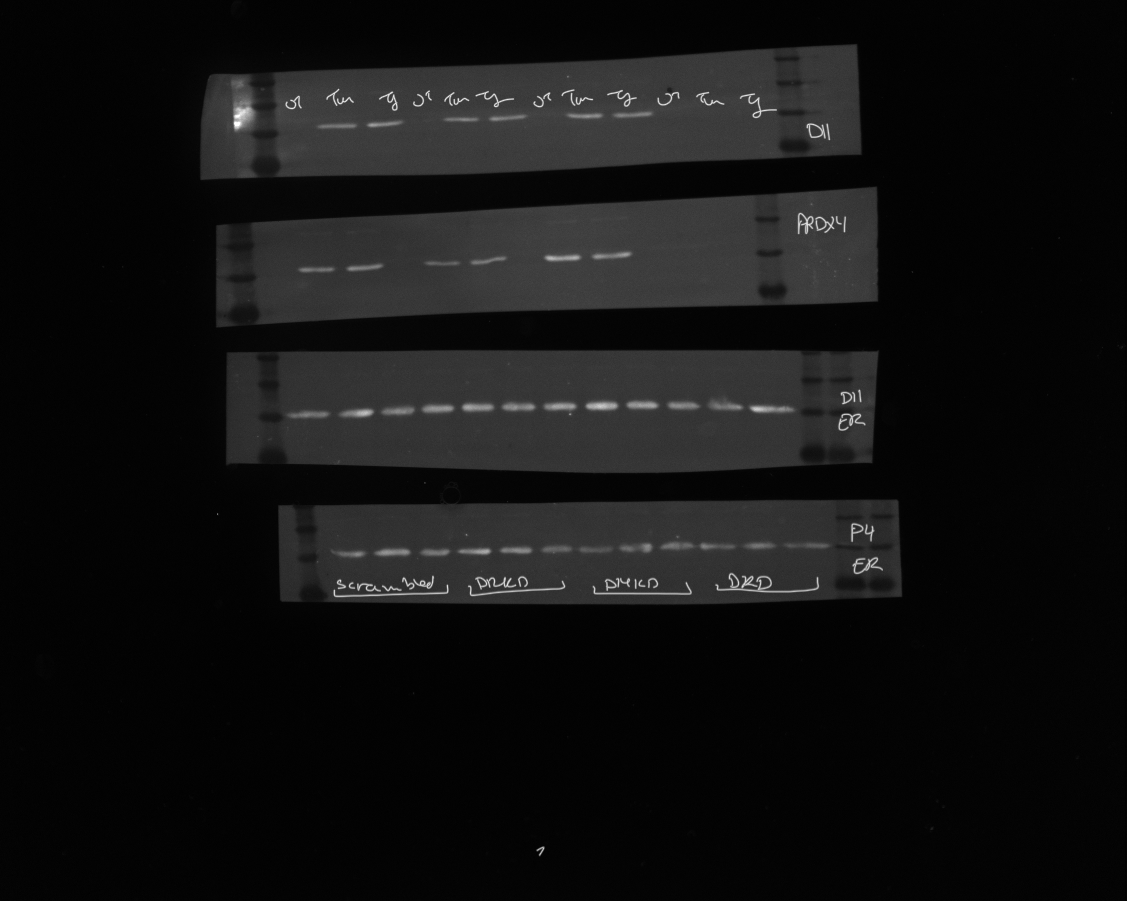

Supplement: Figure 2—figure supplement 1—source data 2. [file elife-102658-fig2-figsupp1-data2.zip › Figure 2-figure suplement 1-source data1/Figure 2-figure suplemment 1-D-1-source data1.tif]

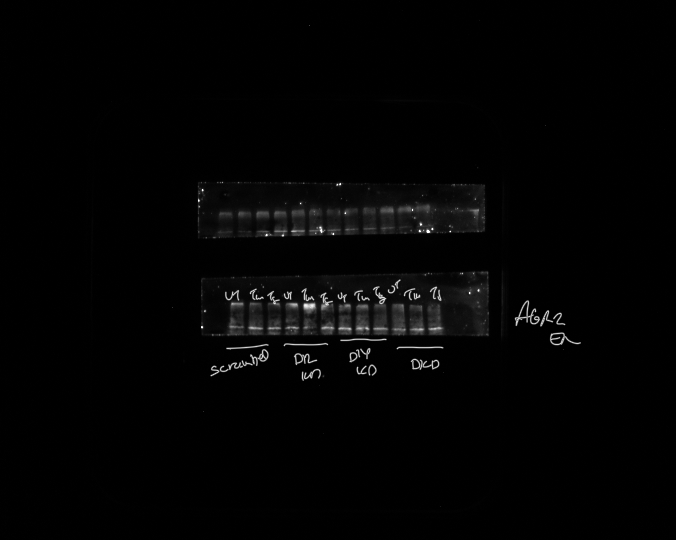

Supplement: Figure 2—figure supplement 1—source data 2. [file elife-102658-fig2-figsupp1-data2.zip › Figure 2-figure suplement 1-source data1/Figure 2-figure suplemment 1-E-6-source data1.tif]

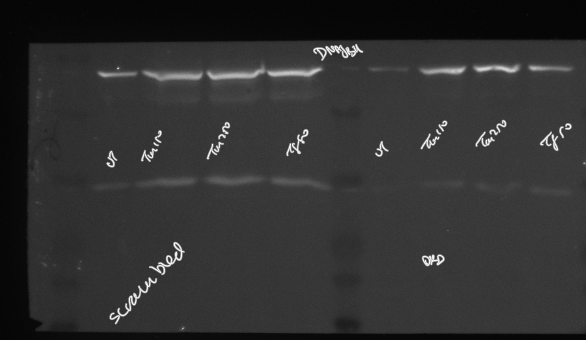

Supplement: Figure 2—figure supplement 1—source data 2. [file elife-102658-fig2-figsupp1-data2.zip › Figure 2-figure suplement 1-source data1/Figure 2-figure suplemment 1-C-3-source data1.tif]

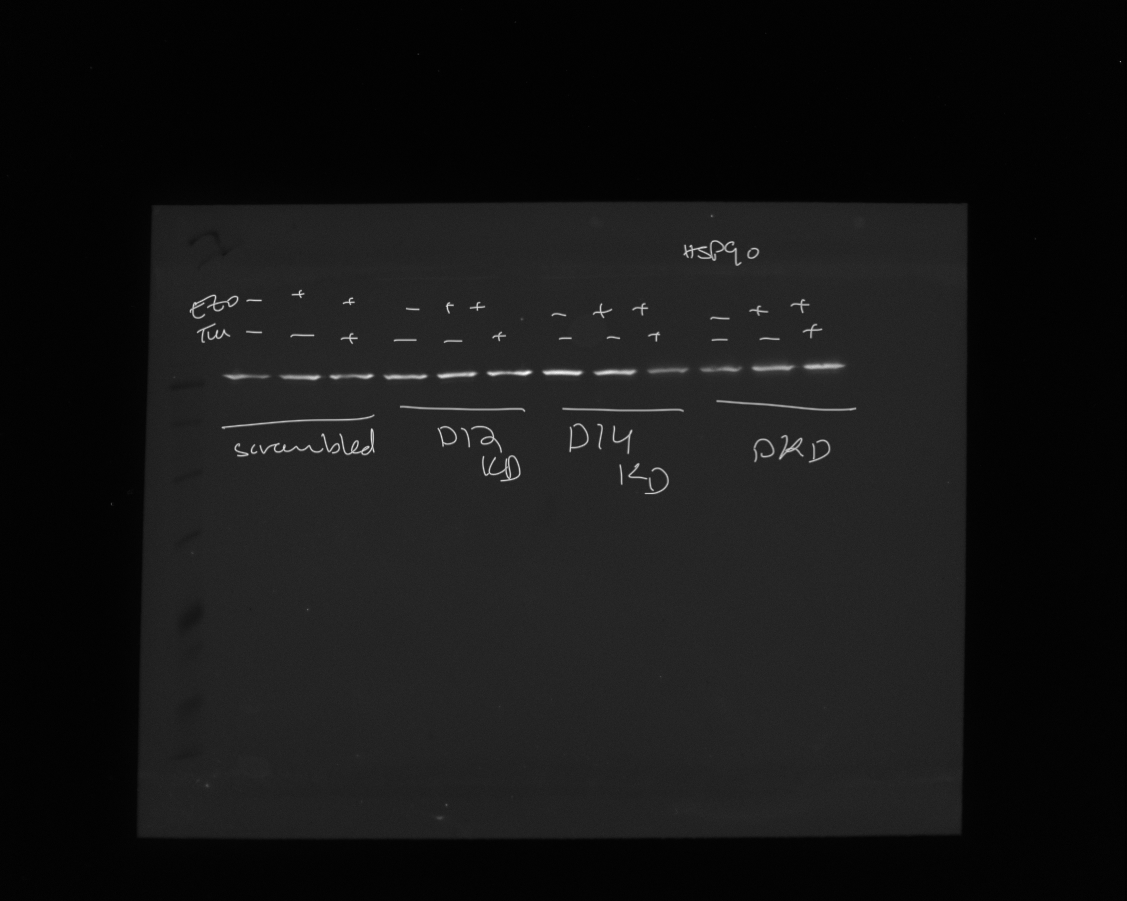

Supplement: Figure 2—figure supplement 1—source data 2. [file elife-102658-fig2-figsupp1-data2.zip › Figure 2-figure suplement 1-source data1/Figure 2-figure suplemment 1-O-4-source data1.tif]

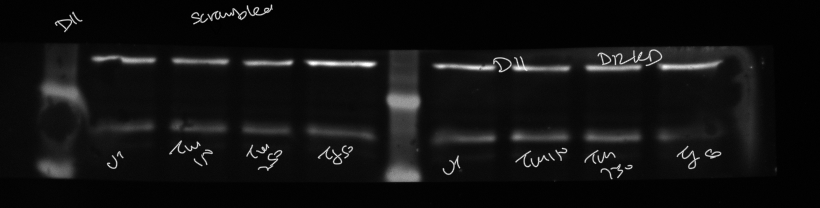

Supplement: Figure 2—figure supplement 1—source data 2. [file elife-102658-fig2-figsupp1-data2.zip › Figure 2-figure suplement 1-source data1/Figure 2-figure suplemment 1-C-12-source data1.tif]

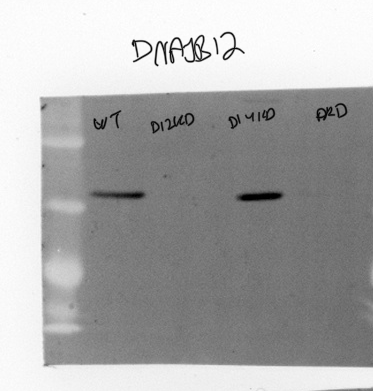

Supplement: Figure 2—figure supplement 1—source data 2. [file elife-102658-fig2-figsupp1-data2.zip › Figure 2-figure suplement 1-source data1/Figure 2-figure suplemment 1-A-B-1-source data1.tif]

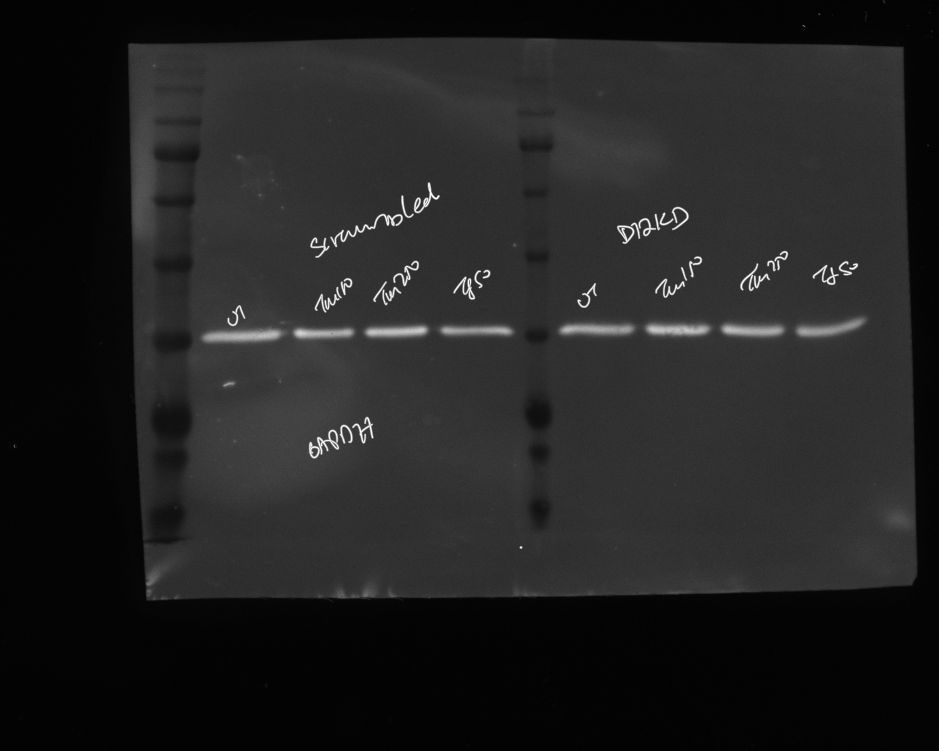

Supplement: Figure 2—figure supplement 1—source data 2. [file elife-102658-fig2-figsupp1-data2.zip › Figure 2-figure suplement 1-source data1/Figure 2-figure suplemment 1-C-8-source data1.tif]

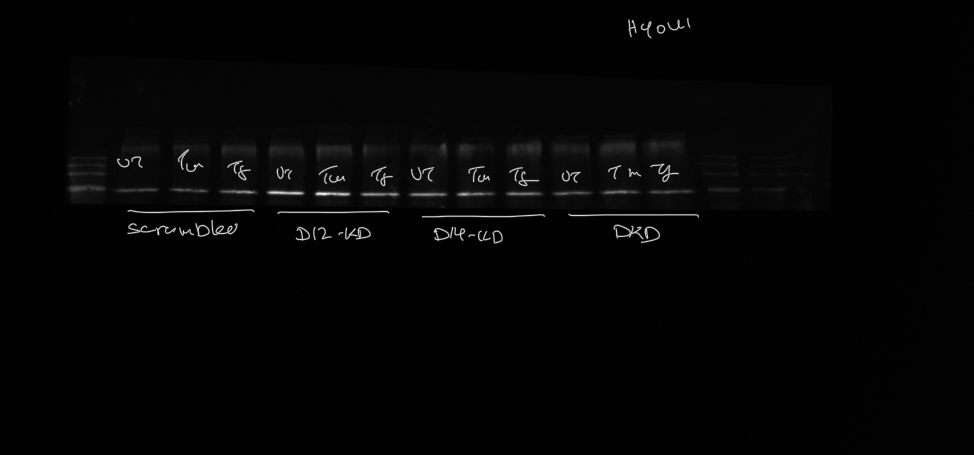

Supplement: Figure 2—figure supplement 1—source data 2. [file elife-102658-fig2-figsupp1-data2.zip › Figure 2-figure suplement 1-source data1/Figure 2-figure suplemment 1-E-9-source data1.tif]

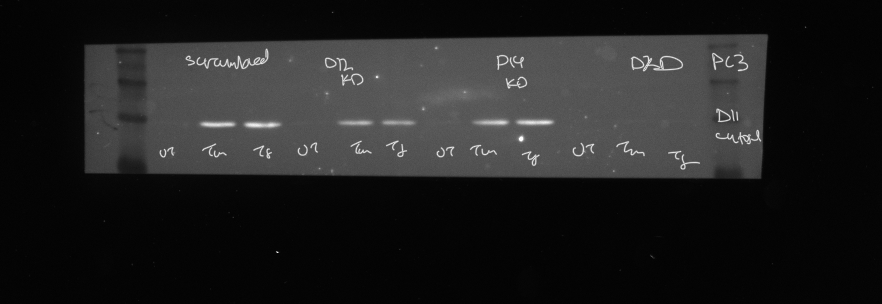

Supplement: Figure 2—figure supplement 1—source data 2. [file elife-102658-fig2-figsupp1-data2.zip › Figure 2-figure suplement 1-source data1/Figure 2-figure suplemment 1-E-2-source data1.tif]

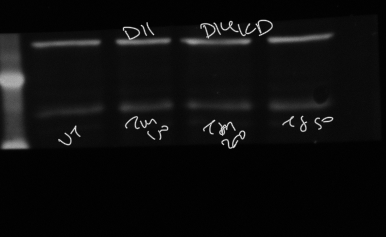

Supplement: Figure 2—figure supplement 1—source data 2. [file elife-102658-fig2-figsupp1-data2.zip › Figure 2-figure suplement 1-source data1/Figure 2-figure suplemment 1-C-13-source data1.tif]

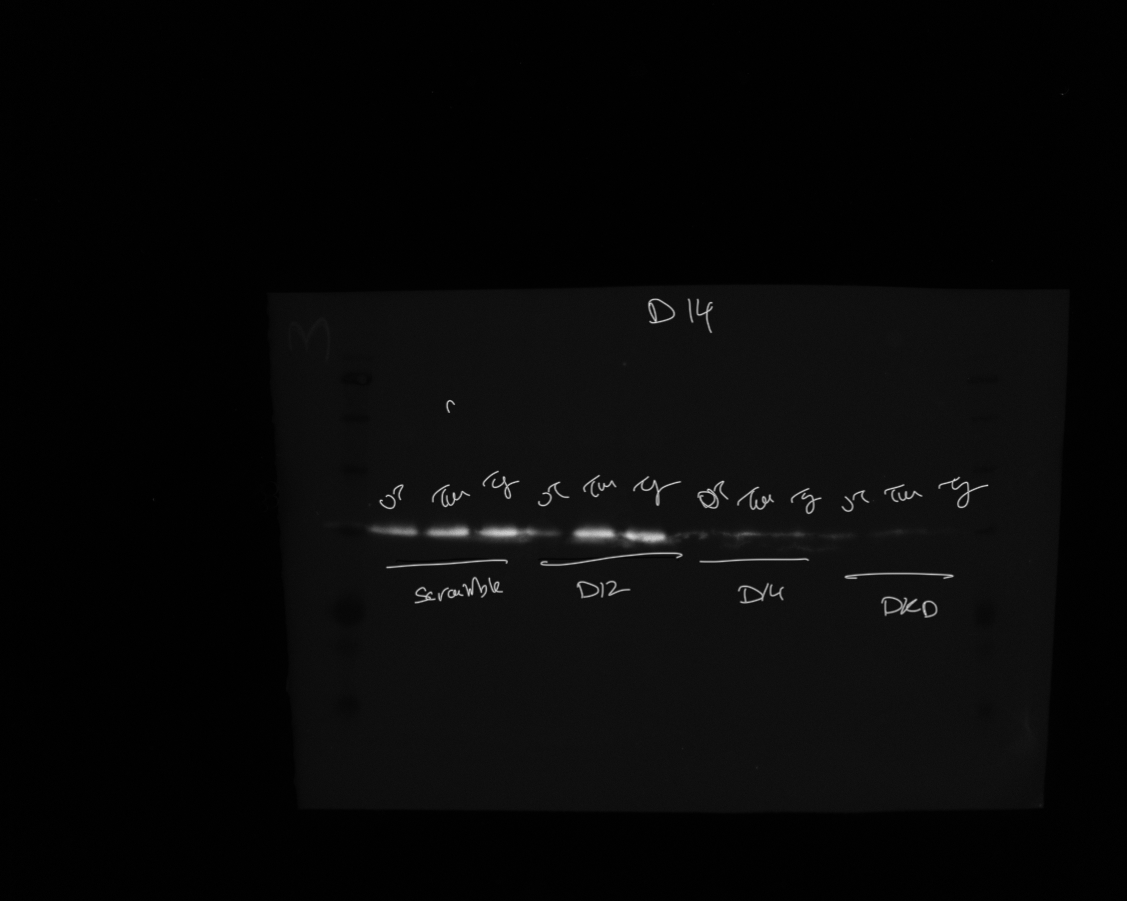

Supplement: Figure 2—figure supplement 1—source data 2. [file elife-102658-fig2-figsupp1-data2.zip › Figure 2-figure suplement 1-source data1/Figure 2-figure suplemment 1-F-3-source data1.tif]

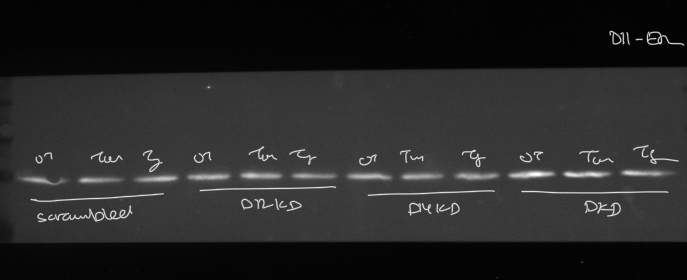

Supplement: Figure 2—figure supplement 1—source data 2. [file elife-102658-fig2-figsupp1-data2.zip › Figure 2-figure suplement 1-source data1/Figure 2-figure suplemment 1-E-7-source data1.tif]

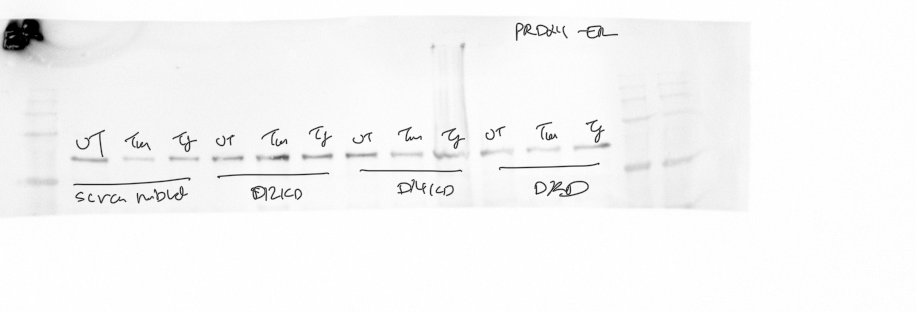

Supplement: Figure 2—figure supplement 1—source data 2. [file elife-102658-fig2-figsupp1-data2.zip › Figure 2-figure suplement 1-source data1/Figure 2-figure suplemment 1-E-8-source data1.tif]

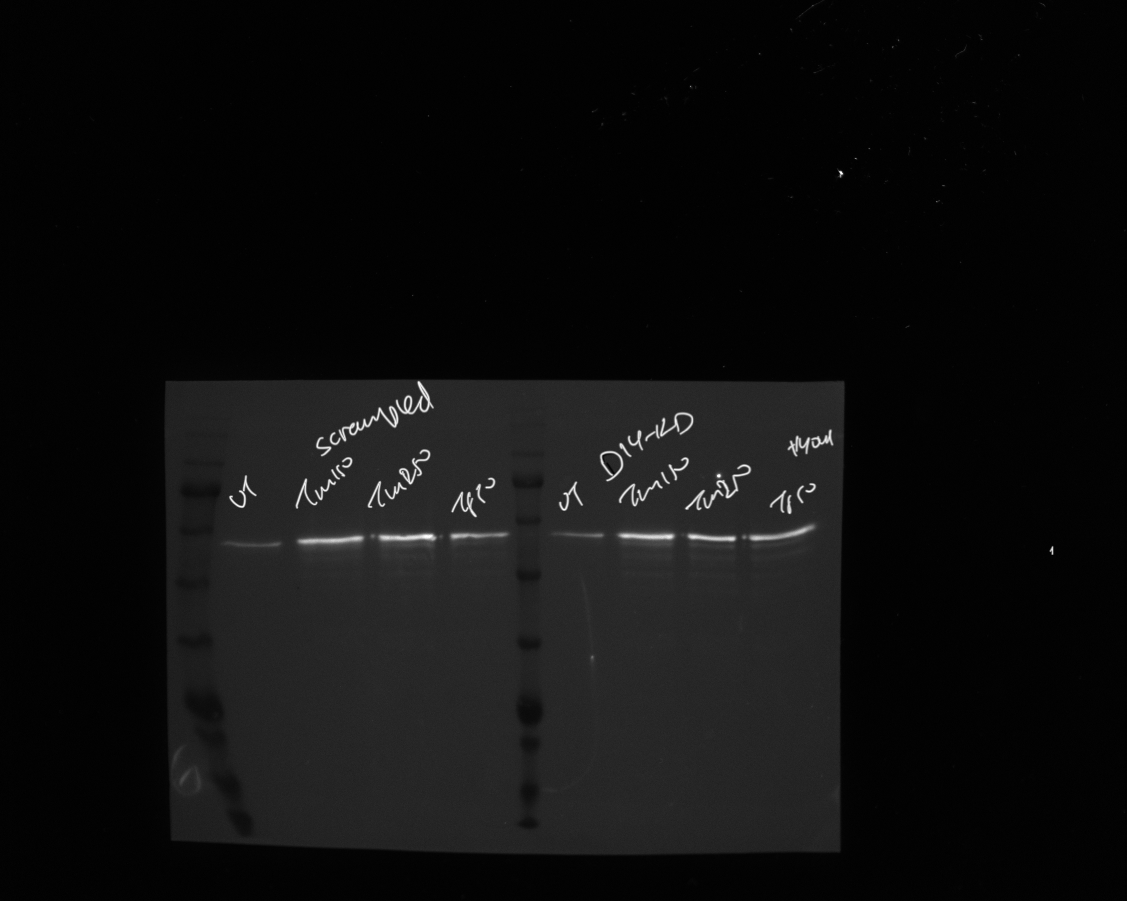

Supplement: Figure 2—figure supplement 1—source data 2. [file elife-102658-fig2-figsupp1-data2.zip › Figure 2-figure suplement 1-source data1/Figure 2-figure suplemment 1-C-6-source data1.tif]

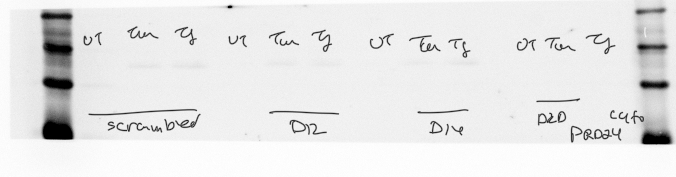

Supplement: Figure 2—figure supplement 1—source data 2. [file elife-102658-fig2-figsupp1-data2.zip › Figure 2-figure suplement 1-source data1/Figure 2-figure suplemment 1-E-3-source data1.tif]

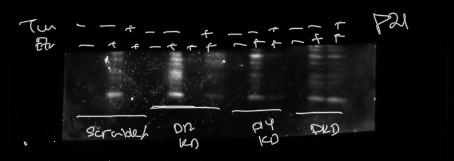

Supplement: Figure 2—figure supplement 1—source data 2. [file elife-102658-fig2-figsupp1-data2.zip › Figure 2-figure suplement 1-source data1/Figure 2-figure suplemment 1-O-1-source data1.tif]

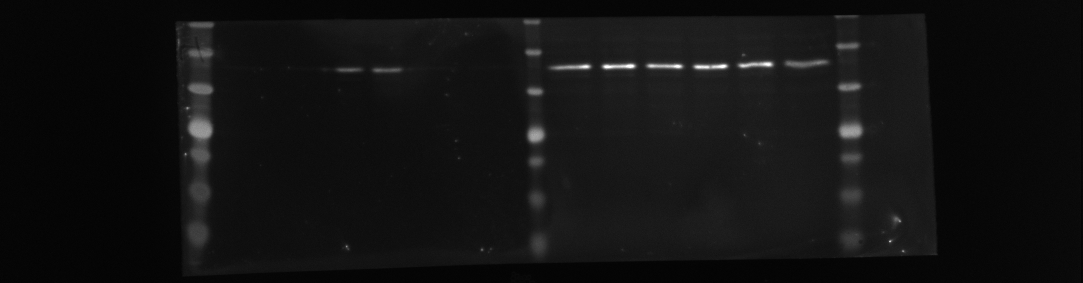

Supplement: Figure 3—source data 1. [file elife-102658-fig3-data1.zip › Figure 3-source data1/Figure 3C-2-source data1.tif]

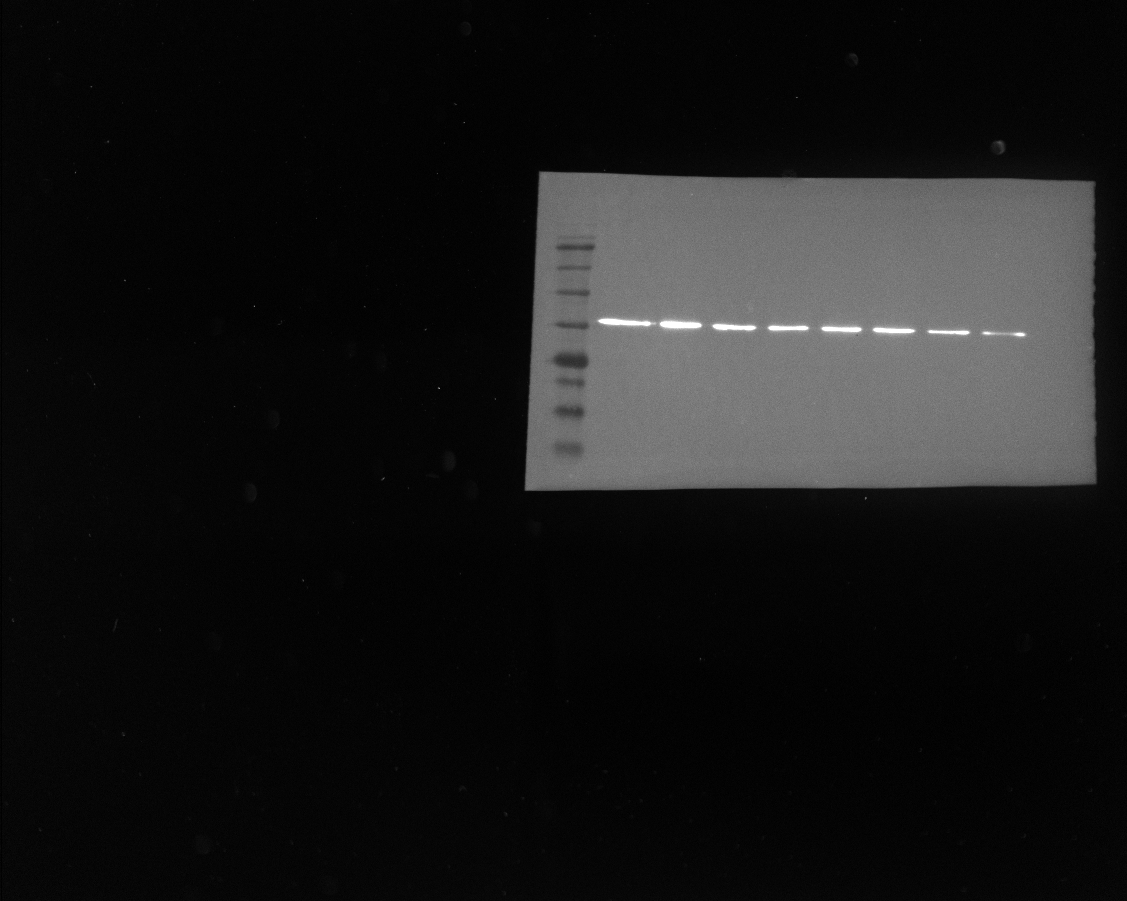

Supplement: Figure 3—source data 1. [file elife-102658-fig3-data1.zip › Figure 3-source data1/Figure 3D-4-source data1.tif]

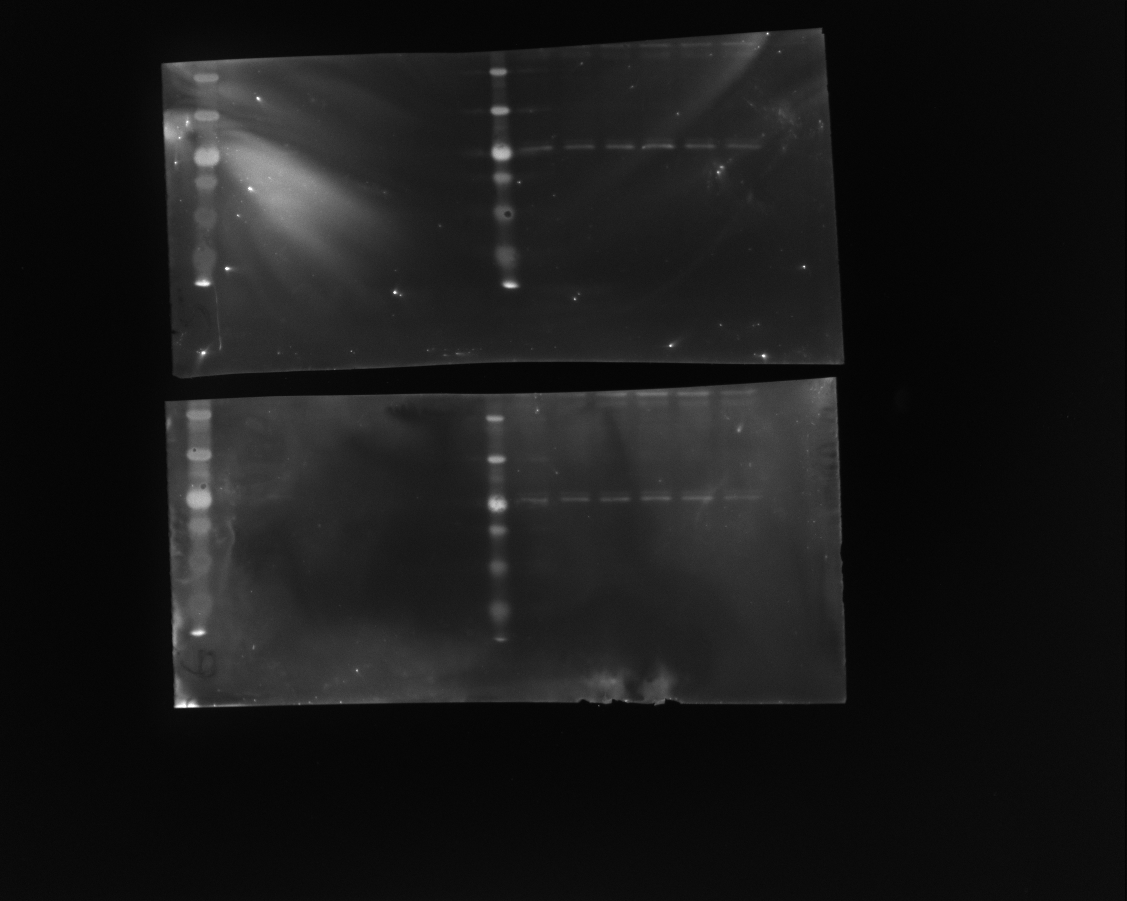

Supplement: Figure 3—source data 1. [file elife-102658-fig3-data1.zip › Figure 3-source data1/Figure 3D-1-source data1.tif]

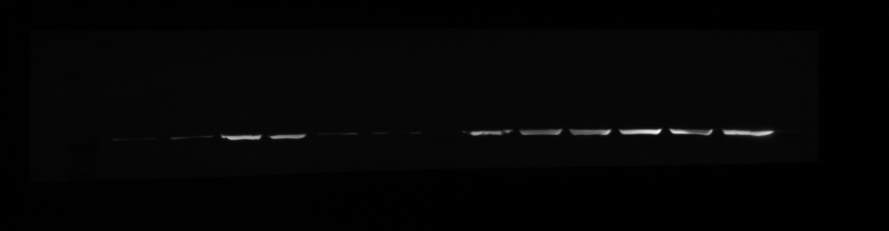

Supplement: Figure 3—source data 1. [file elife-102658-fig3-data1.zip › Figure 3-source data1/Figure 3C-3-source data1.tif]

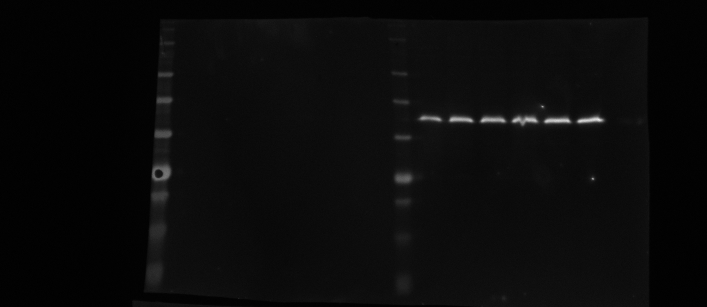

Supplement: Figure 3—source data 1. [file elife-102658-fig3-data1.zip › Figure 3-source data1/Figure 3D-2-source data1.tif]

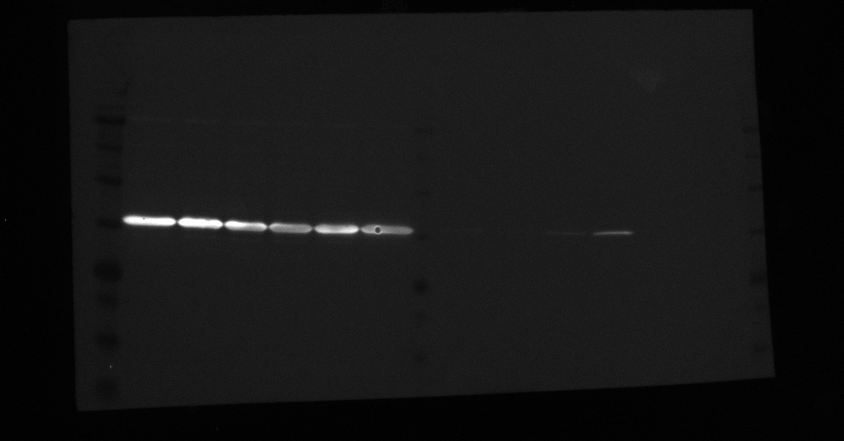

Supplement: Figure 3—source data 1. [file elife-102658-fig3-data1.zip › Figure 3-source data1/Figure 3C-4-source data1.tif]

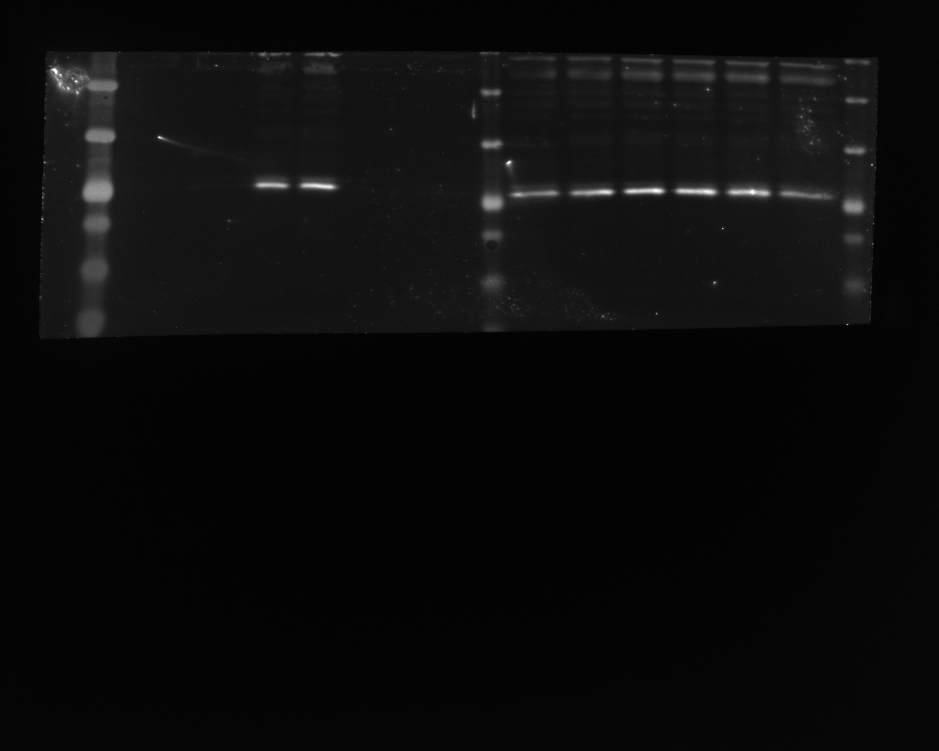

Supplement: Figure 3—source data 1. [file elife-102658-fig3-data1.zip › Figure 3-source data1/Figure 3C-1-Source data 1.tif]

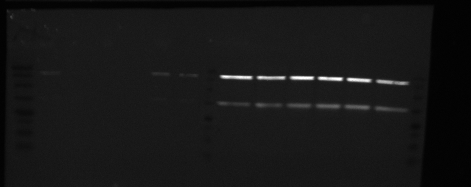

Supplement: Figure 3—source data 1. [file elife-102658-fig3-data1.zip › Figure 3-source data1/Figure 3D-3-source data1.tif]

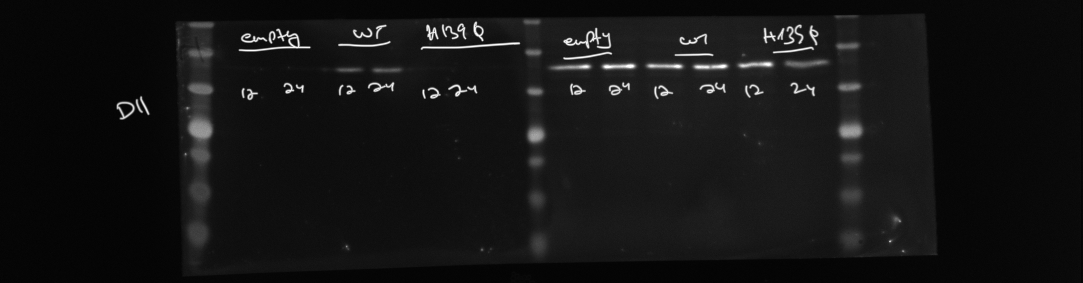

Supplement: Figure 3—source data 2. [file elife-102658-fig3-data2.zip › Figure 3-source data1 2/Figure 3C-2-source data1.tif]

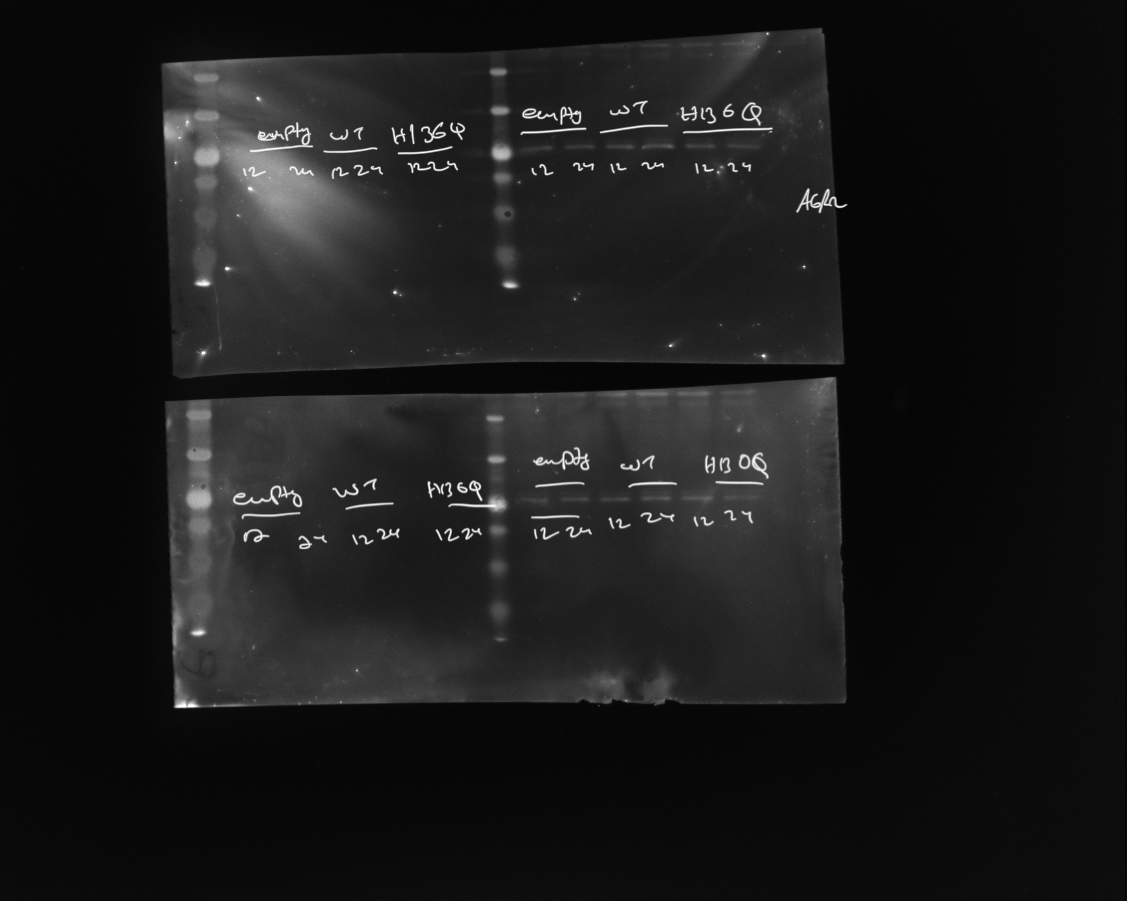

Supplement: Figure 3—source data 2. [file elife-102658-fig3-data2.zip › Figure 3-source data1 2/Figure 3D-1-source data1.tif]

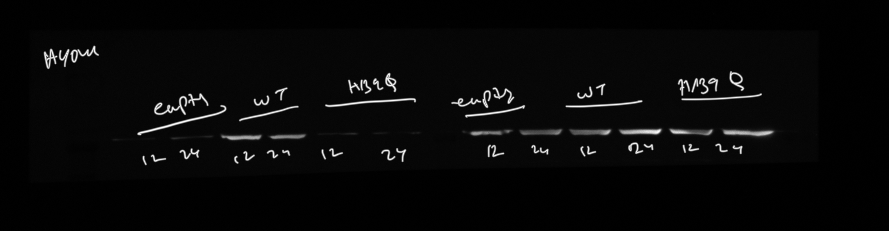

Supplement: Figure 3—source data 2. [file elife-102658-fig3-data2.zip › Figure 3-source data1 2/Figure 3C-3-source data1.tif]

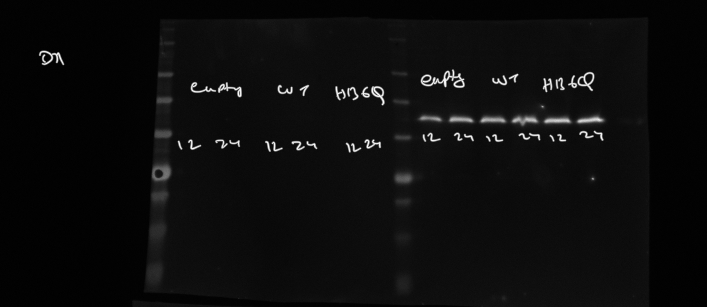

Supplement: Figure 3—source data 2. [file elife-102658-fig3-data2.zip › Figure 3-source data1 2/Figure 3D-2-source data1.tif]

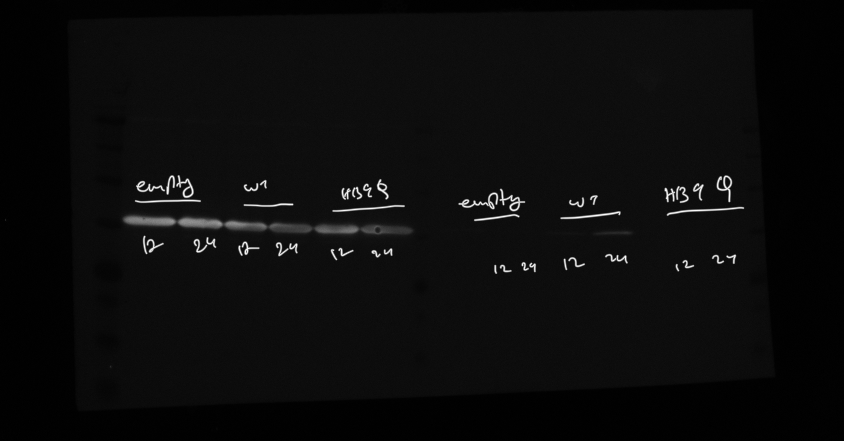

Supplement: Figure 3—source data 2. [file elife-102658-fig3-data2.zip › Figure 3-source data1 2/Figure 3C-4-source data1.tif]

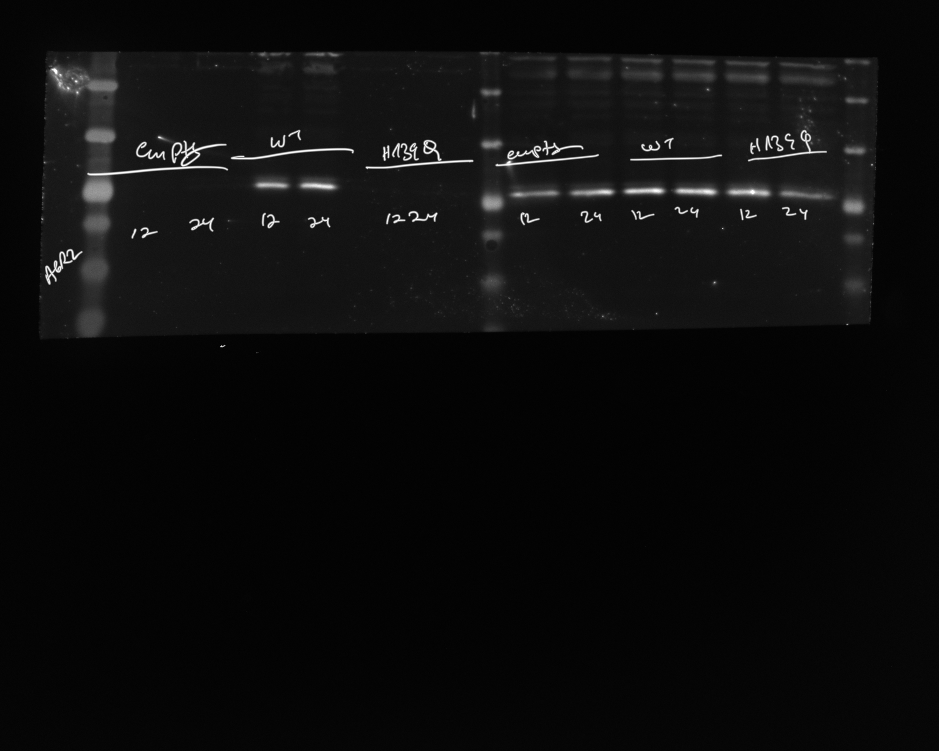

Supplement: Figure 3—source data 2. [file elife-102658-fig3-data2.zip › Figure 3-source data1 2/Figure 3C-1-Source data 1.tif]

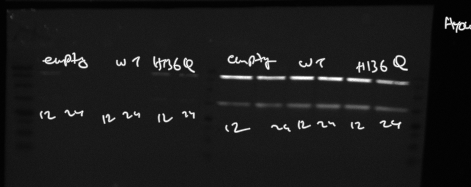

Supplement: Figure 3—source data 2. [file elife-102658-fig3-data2.zip › Figure 3-source data1 2/Figure 3D-3-source data1.tif]

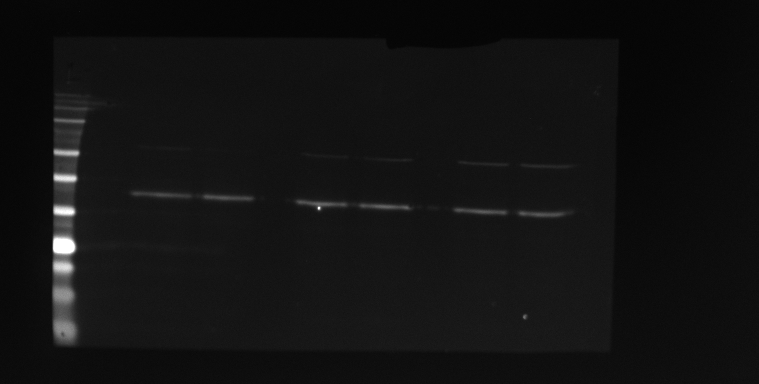

Supplement: Figure 3—figure supplement 1—source data 1. [file elife-102658-fig3-figsupp1-data1.zip › Figure 3-figure suplement 1-source data1/Figure 3-figure suplemment 1-A-B-2-source data1.tif]

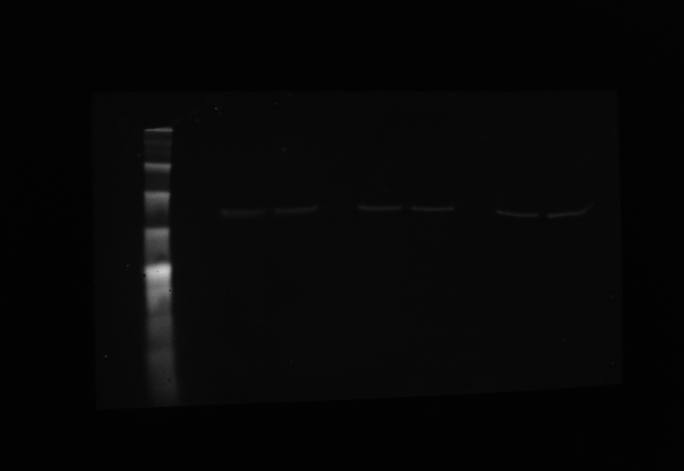

Supplement: Figure 3—figure supplement 1—source data 1. [file elife-102658-fig3-figsupp1-data1.zip › Figure 3-figure suplement 1-source data1/Figure 3-figure suplemment 1-A-B-1-source data1.tif]

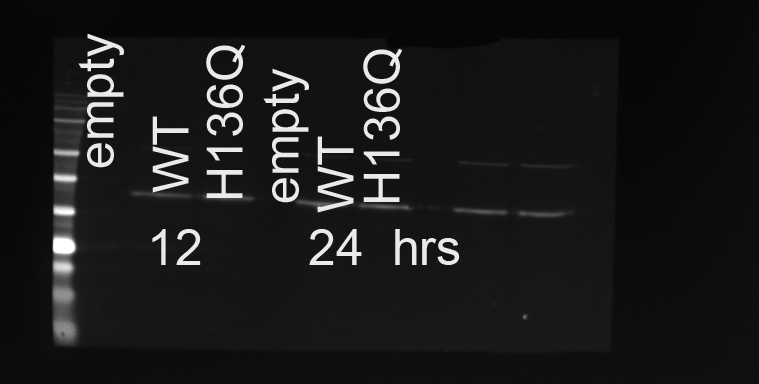

Supplement: Figure 3—figure supplement 1—source data 2. [file elife-102658-fig3-figsupp1-data2.zip › Figure 3-figure suplement 1-source data2/Figure 3-figure suplemment 1-A-B-2-source data1.tif]

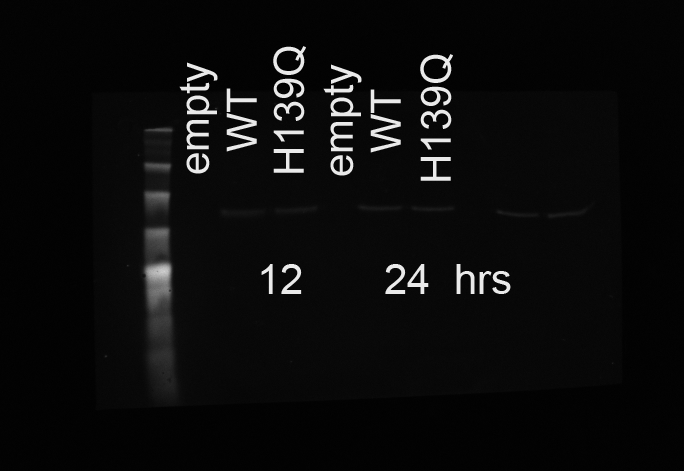

Supplement: Figure 3—figure supplement 1—source data 2. [file elife-102658-fig3-figsupp1-data2.zip › Figure 3-figure suplement 1-source data2/Figure 3-figure suplemment 1-A-B-1-source data1.tif]

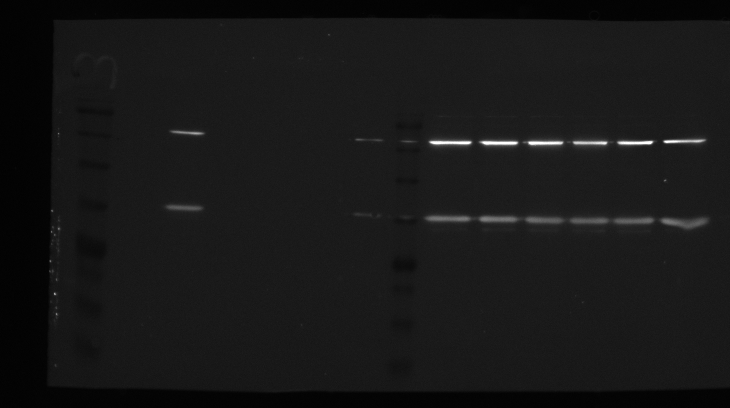

Supplement: Figure 4—source data 1. [file elife-102658-fig4-data1.zip › Figure 4-source data1/Figure 4F-3-source data1.tif]
