# Supplementary figures and images for: Cytosolic and endoplasmic reticulum chaperones inhibit wt-p53 to increase cancer cells' survival by refluxing ER-proteins to the cytosol (part 2 of 3)
Source: eLife. 2025 Apr 9;14:e102658. doi: 10.7554/eLife.102658 (PMC11981610; doi:10.7554/eLife.102658)

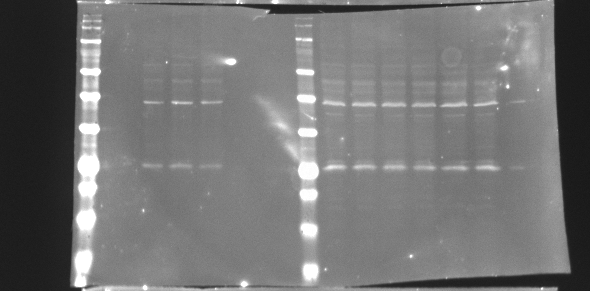

Supplement: Figure 4—source data 1. [file elife-102658-fig4-data1.zip › Figure 4-source data1/Figure 4A-1-source data1.tif]

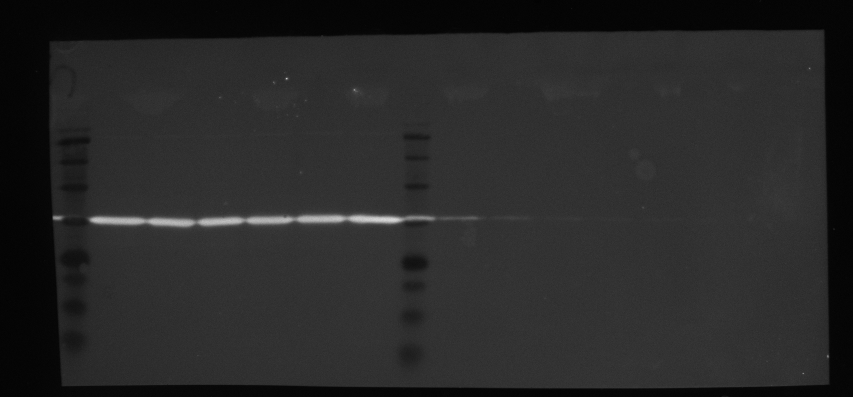

Supplement: Figure 4—source data 1. [file elife-102658-fig4-data1.zip › Figure 4-source data1/Figure 4A-4-source data1.tif]

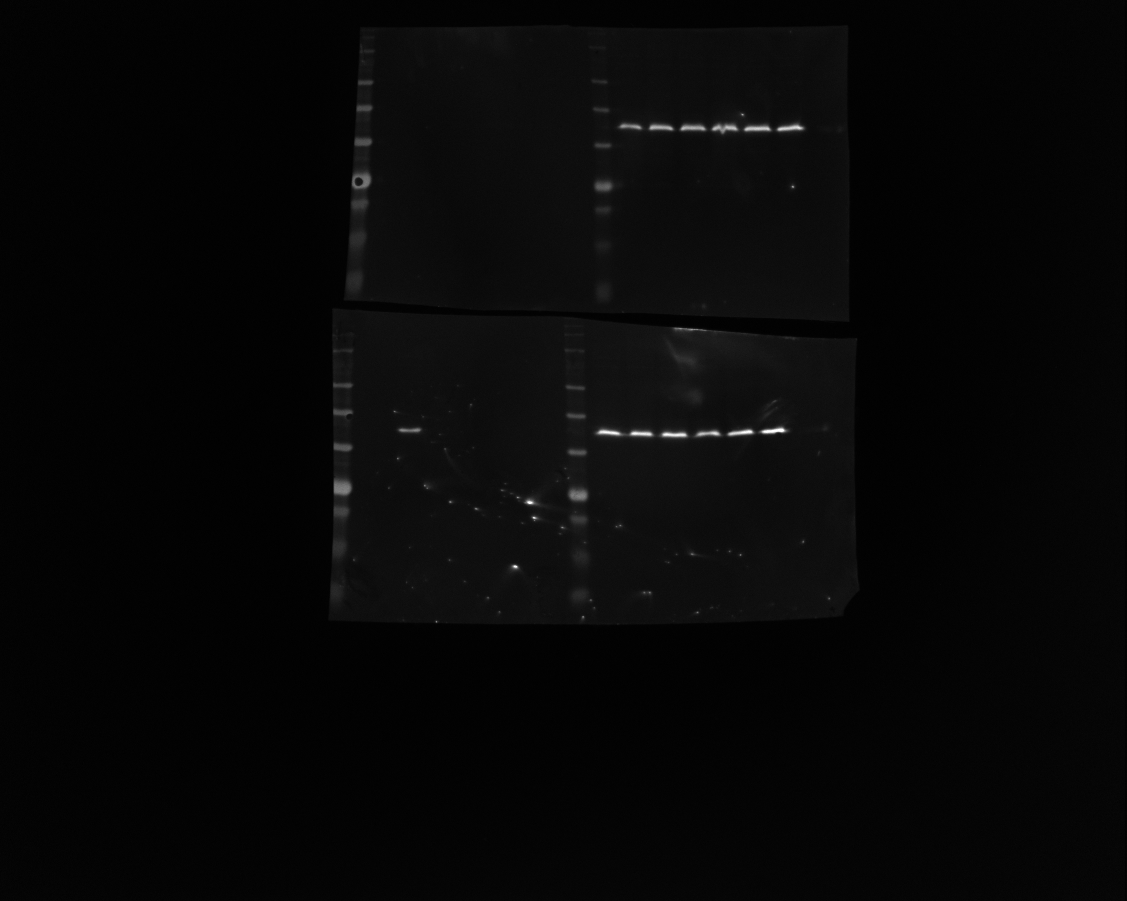

Supplement: Figure 4—source data 1. [file elife-102658-fig4-data1.zip › Figure 4-source data1/Figure 4F-2-source data1.tif]

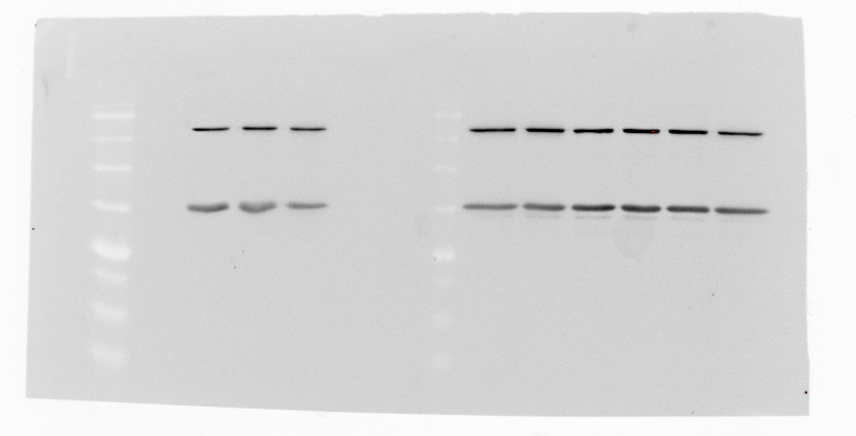

Supplement: Figure 4—source data 1. [file elife-102658-fig4-data1.zip › Figure 4-source data1/Figure 4A-3-source data1.tif]

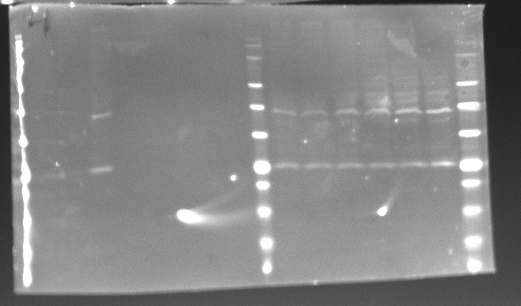

Supplement: Figure 4—source data 1. [file elife-102658-fig4-data1.zip › Figure 4-source data1/Figure 4F-1-source data1.tif]

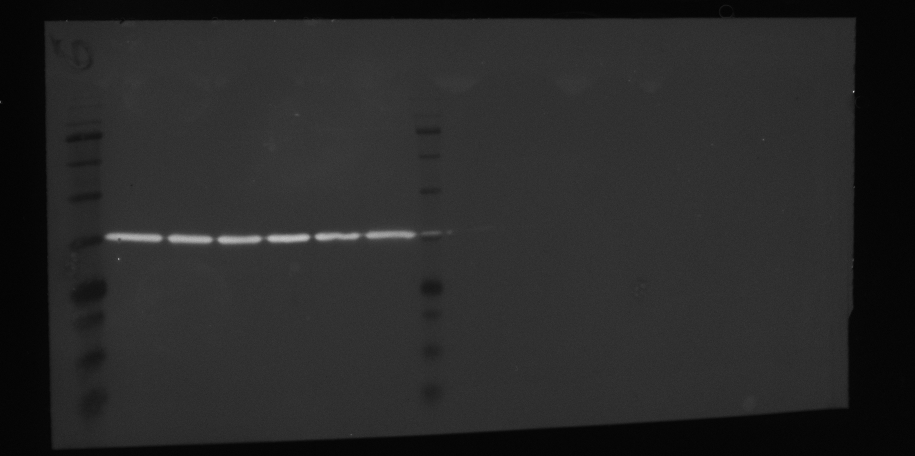

Supplement: Figure 4—source data 1. [file elife-102658-fig4-data1.zip › Figure 4-source data1/Figure 4F-4-source data1.tif]

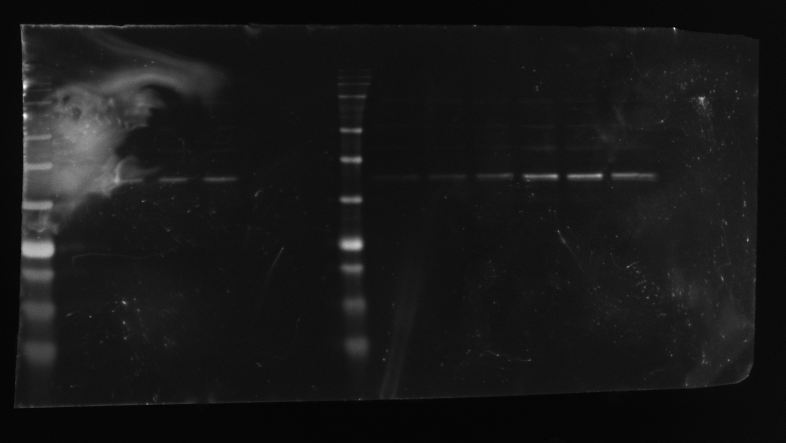

Supplement: Figure 4—source data 1. [file elife-102658-fig4-data1.zip › Figure 4-source data1/Figure 4A-2-source data1.tif]

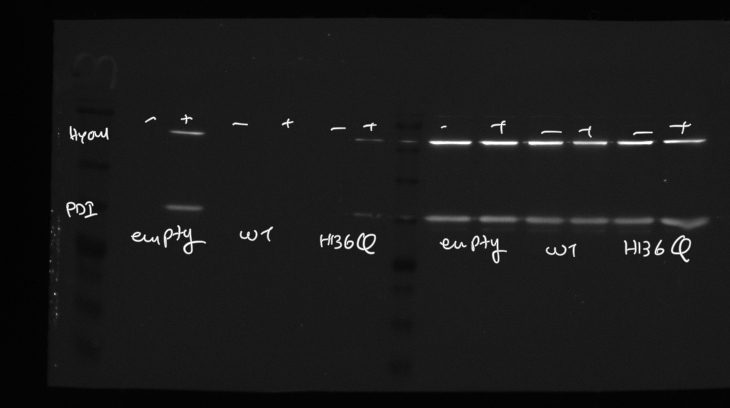

Supplement: Figure 4—source data 2. [file elife-102658-fig4-data2.zip › Figure 4-source data1/Figure 4F-3-source data1.tif]

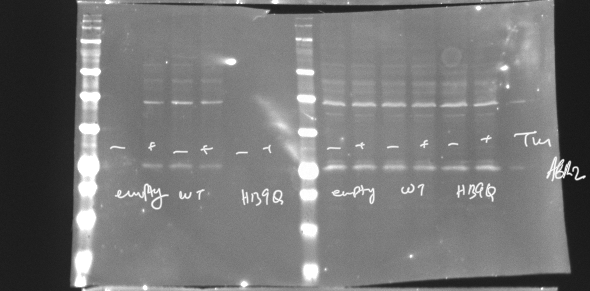

Supplement: Figure 4—source data 2. [file elife-102658-fig4-data2.zip › Figure 4-source data1/Figure 4A-1-source data1.tif]

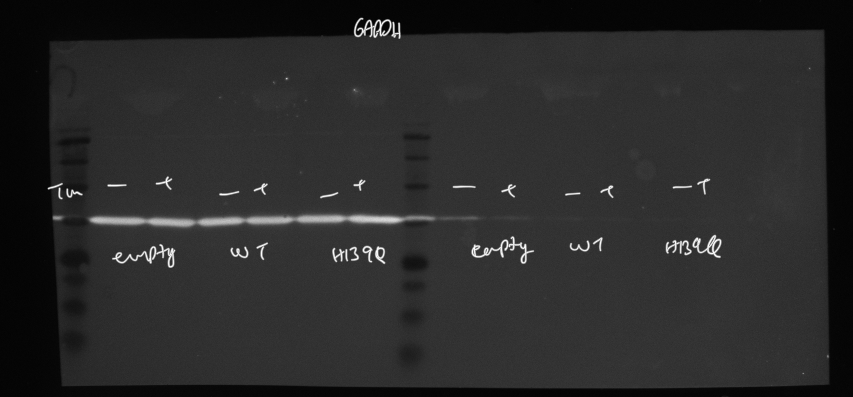

Supplement: Figure 4—source data 2. [file elife-102658-fig4-data2.zip › Figure 4-source data1/Figure 4A-4-source data1.tif]

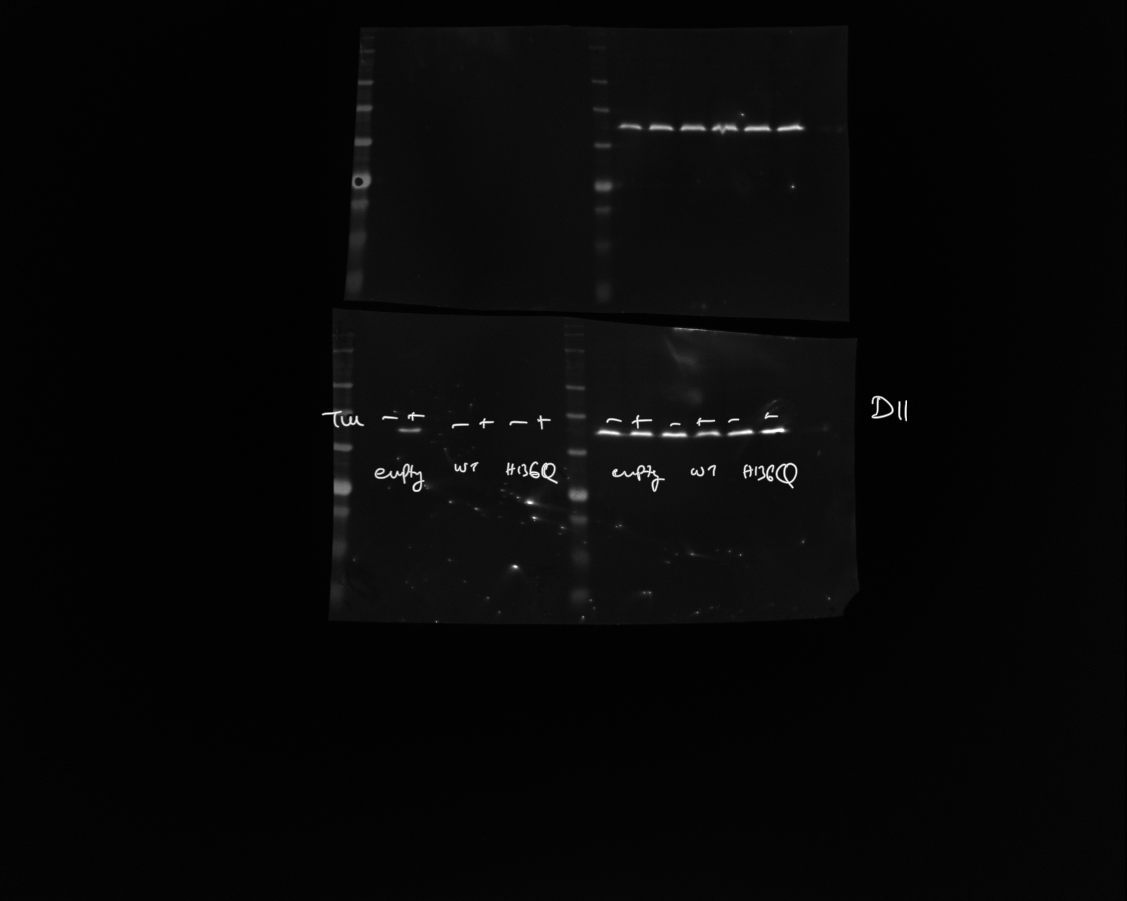

Supplement: Figure 4—source data 2. [file elife-102658-fig4-data2.zip › Figure 4-source data1/Figure 4F-2-source data1.tif]

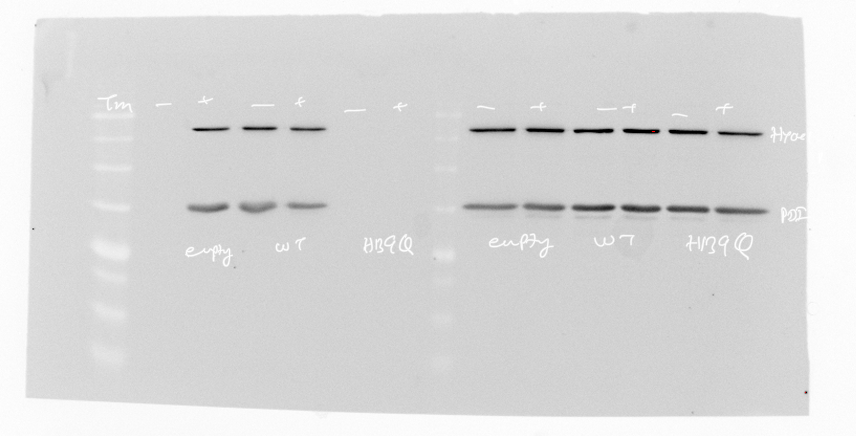

Supplement: Figure 4—source data 2. [file elife-102658-fig4-data2.zip › Figure 4-source data1/Figure 4A-3-source data1.tif]

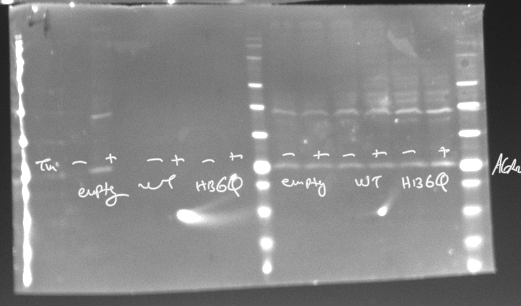

Supplement: Figure 4—source data 2. [file elife-102658-fig4-data2.zip › Figure 4-source data1/Figure 4F-1-source data1.tif]

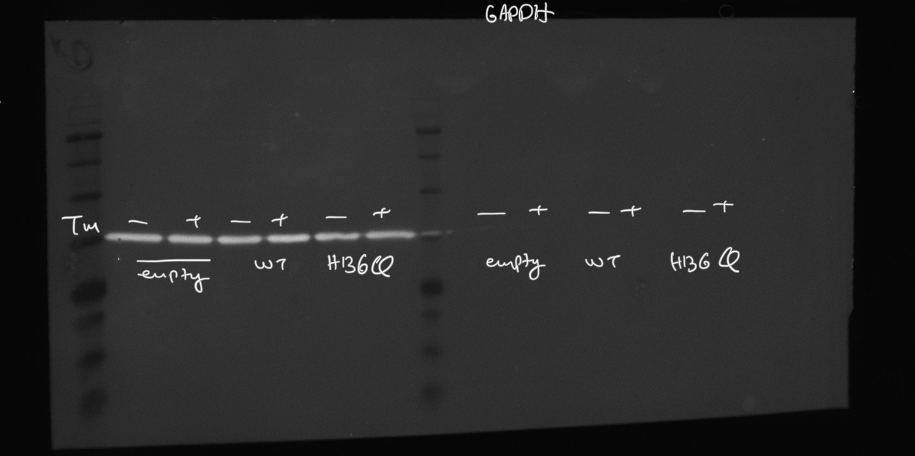

Supplement: Figure 4—source data 2. [file elife-102658-fig4-data2.zip › Figure 4-source data1/Figure 4F-4-source data1.tif]

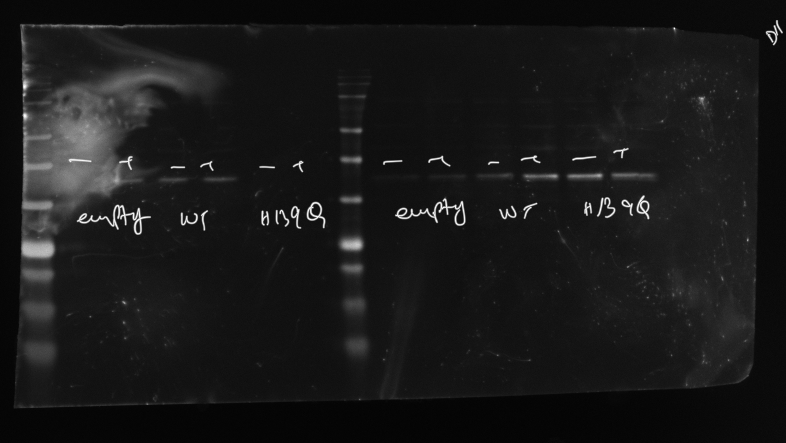

Supplement: Figure 4—source data 2. [file elife-102658-fig4-data2.zip › Figure 4-source data1/Figure 4A-2-source data1.tiff]

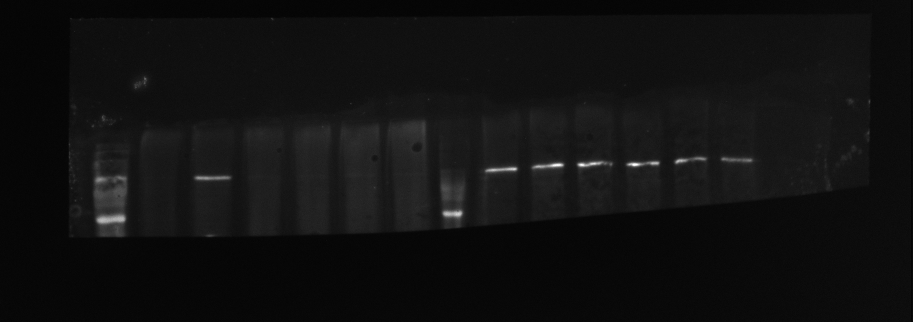

Supplement: Figure 4—figure supplement 1—source data 1. [file elife-102658-fig4-figsupp1-data1.zip › Figure 4-figure suplement 1-source data1/Figure 4-figure suplemment 1-B-5-source data1.tif]

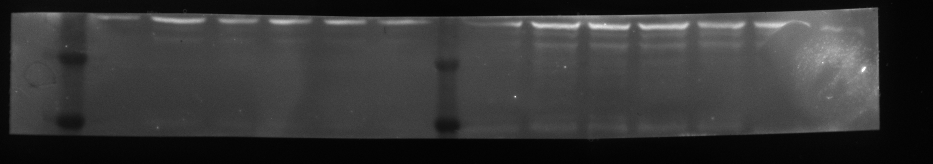

Supplement: Figure 4—figure supplement 1—source data 1. [file elife-102658-fig4-figsupp1-data1.zip › Figure 4-figure suplement 1-source data1/Figure 4-figure suplemment 1-C-2-source data1.tif]

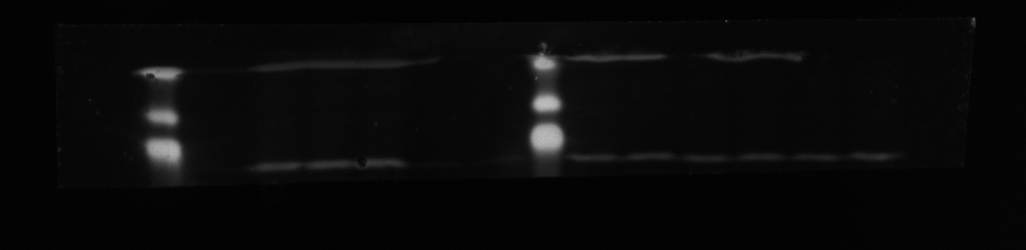

Supplement: Figure 4—figure supplement 1—source data 1. [file elife-102658-fig4-figsupp1-data1.zip › Figure 4-figure suplement 1-source data1/Figure 4-figure suplemment 1-A-1-source data1.tif]

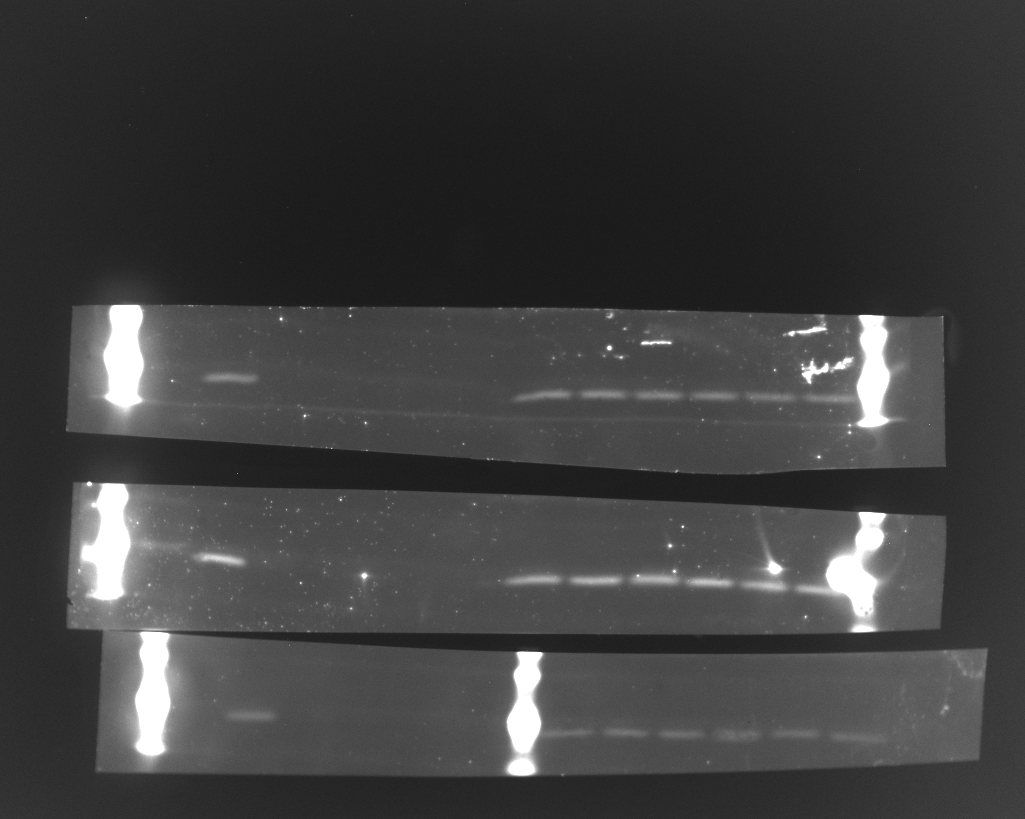

Supplement: Figure 4—figure supplement 1—source data 1. [file elife-102658-fig4-figsupp1-data1.zip › Figure 4-figure suplement 1-source data1/Figure 4-figure suplemment 1-B-1-source data1.tif]

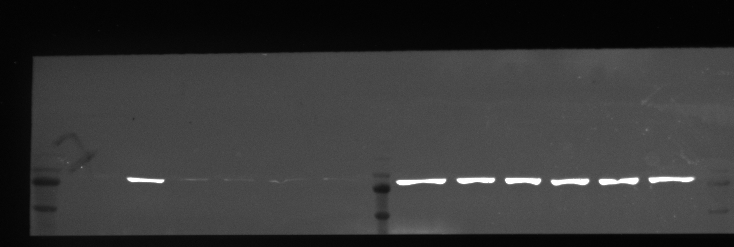

Supplement: Figure 4—figure supplement 1—source data 1. [file elife-102658-fig4-figsupp1-data1.zip › Figure 4-figure suplement 1-source data1/Figure 4-figure suplemment 1-B-4-source data1.tif]

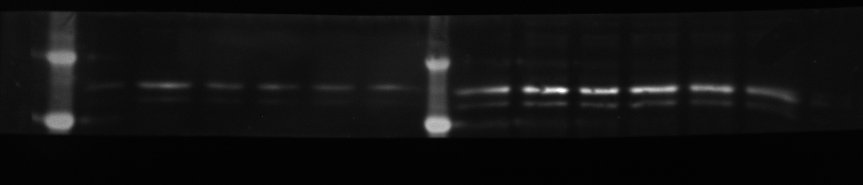

Supplement: Figure 4—figure supplement 1—source data 1. [file elife-102658-fig4-figsupp1-data1.zip › Figure 4-figure suplement 1-source data1/Figure 4-figure suplemment 1-C-3-source data1.tif]

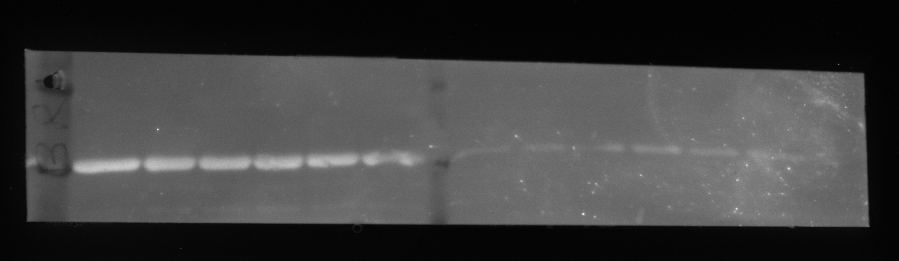

Supplement: Figure 4—figure supplement 1—source data 1. [file elife-102658-fig4-figsupp1-data1.zip › Figure 4-figure suplement 1-source data1/Figure 4-figure suplemment 1-A-4-source data1.tif]

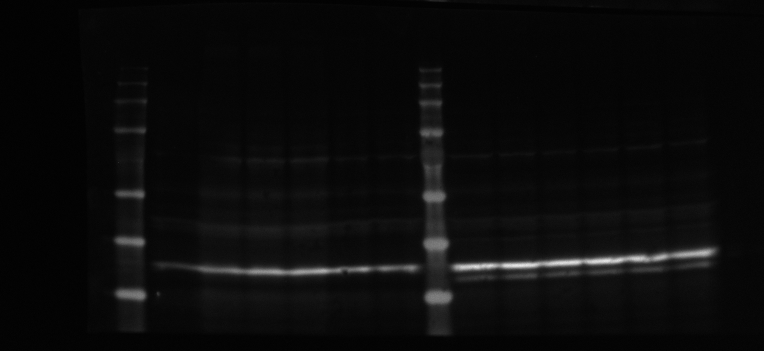

Supplement: Figure 4—figure supplement 1—source data 1. [file elife-102658-fig4-figsupp1-data1.zip › Figure 4-figure suplement 1-source data1/Figure 4-figure suplemment 1-A-3-source data1.tif]

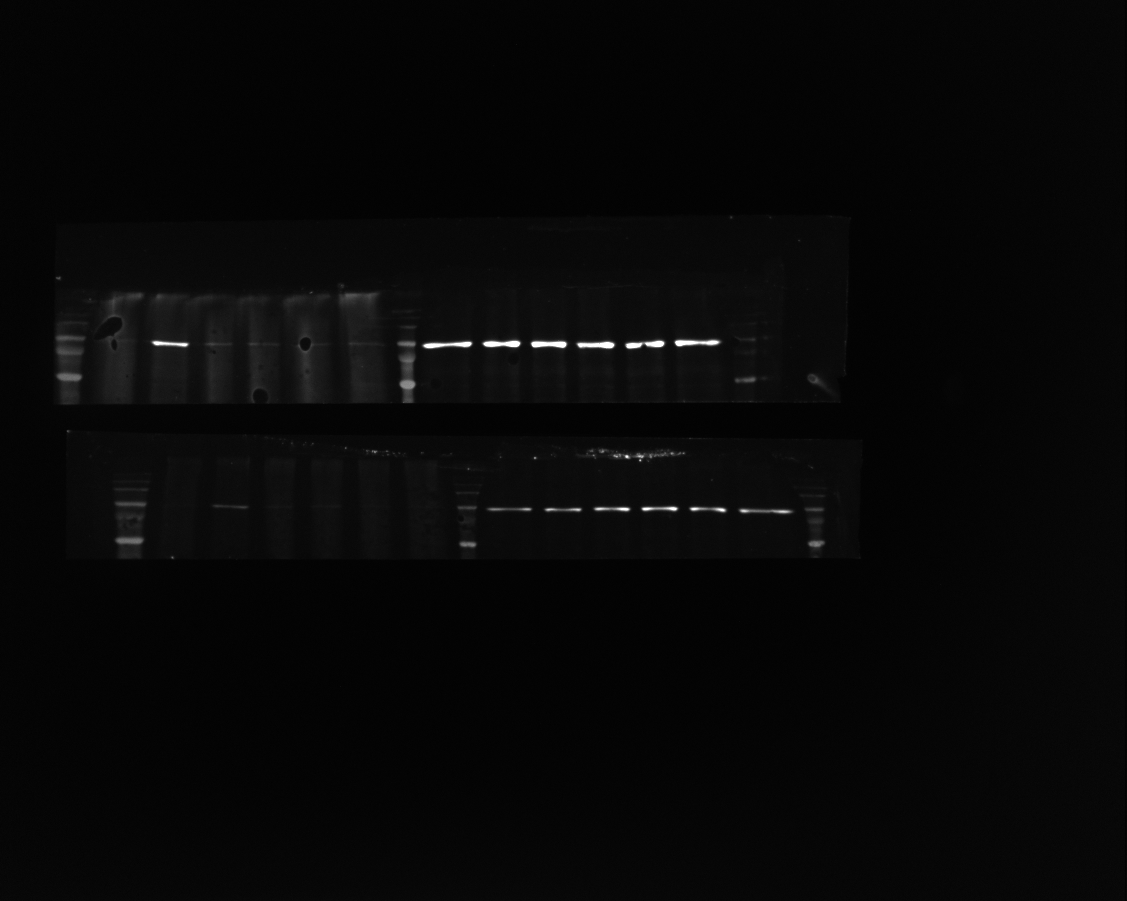

Supplement: Figure 4—figure supplement 1—source data 1. [file elife-102658-fig4-figsupp1-data1.zip › Figure 4-figure suplement 1-source data1/Figure 4-figure suplemment 1-B-3-source data1.tif]

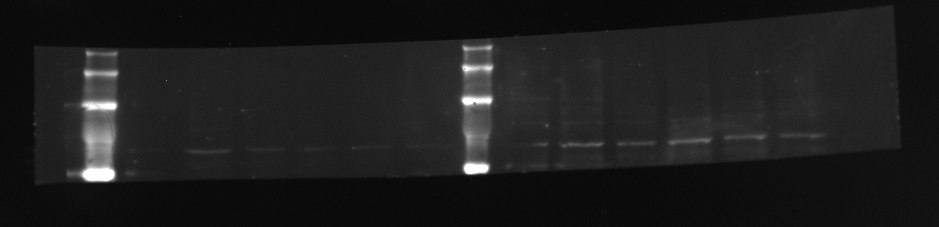

Supplement: Figure 4—figure supplement 1—source data 1. [file elife-102658-fig4-figsupp1-data1.zip › Figure 4-figure suplement 1-source data1/Figure 4-figure suplemment 1-C-4-source data1.tif]

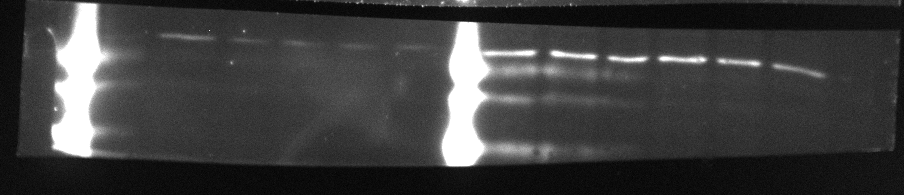

Supplement: Figure 4—figure supplement 1—source data 1. [file elife-102658-fig4-figsupp1-data1.zip › Figure 4-figure suplement 1-source data1/Figure 4-figure suplemment 1-C-1-source data1.tif]

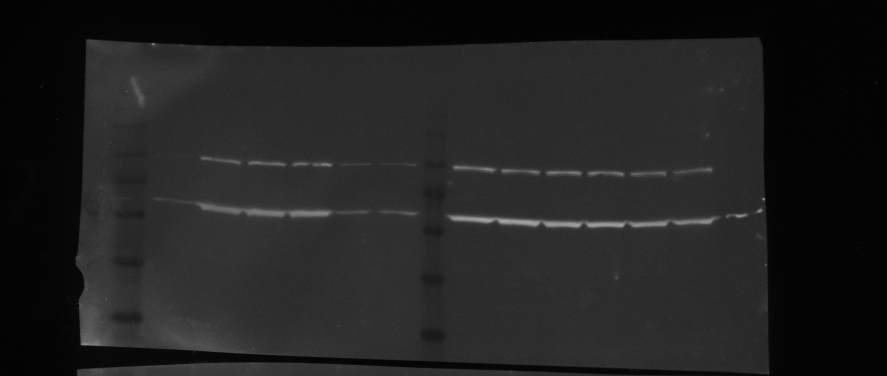

Supplement: Figure 4—figure supplement 1—source data 1. [file elife-102658-fig4-figsupp1-data1.zip › Figure 4-figure suplement 1-source data1/Figure 4-figure suplemment 1-A-2-source data1.tif]

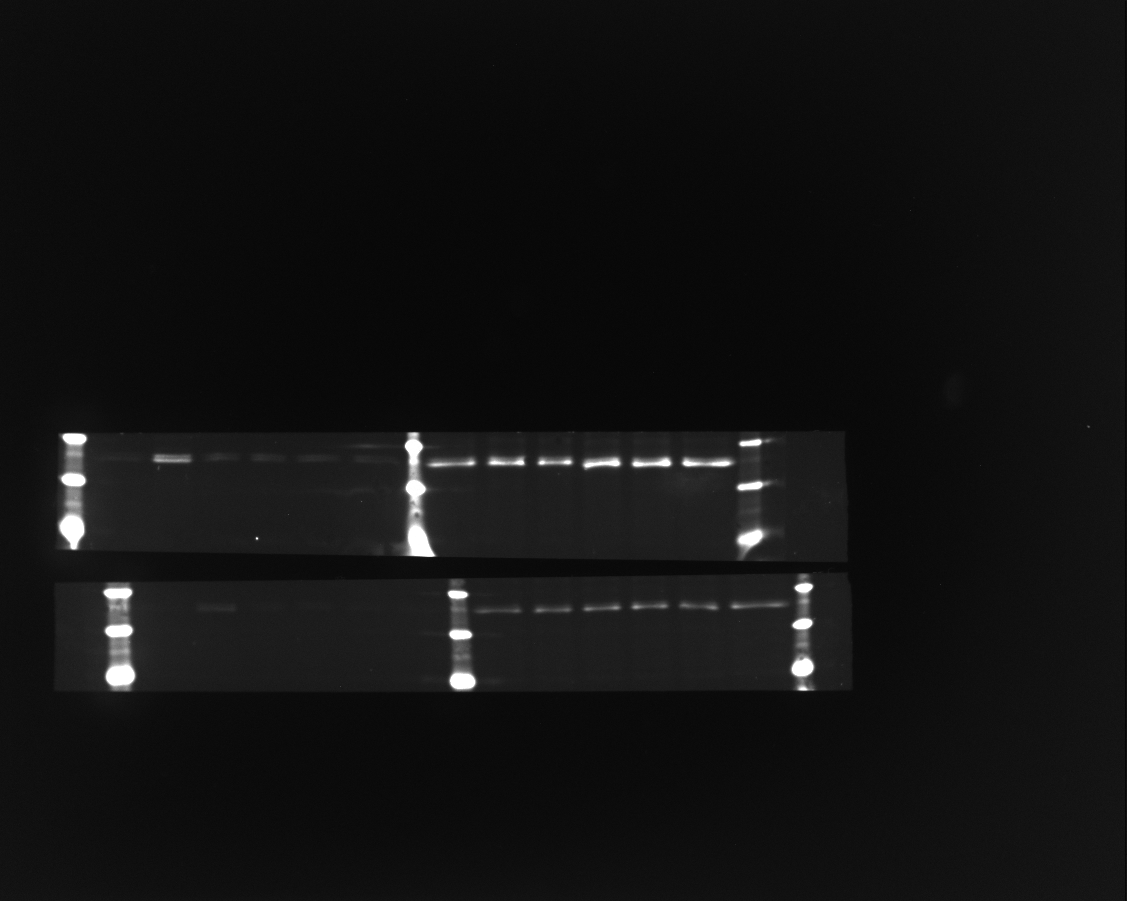

Supplement: Figure 4—figure supplement 1—source data 1. [file elife-102658-fig4-figsupp1-data1.zip › Figure 4-figure suplement 1-source data1/Figure 4-figure suplemment 1-B-2-source data1.tif]

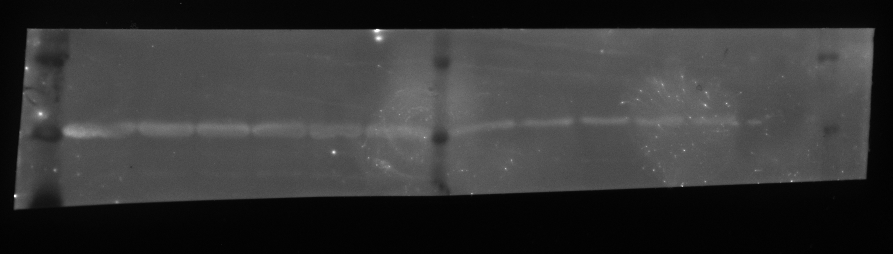

Supplement: Figure 4—figure supplement 1—source data 1. [file elife-102658-fig4-figsupp1-data1.zip › Figure 4-figure suplement 1-source data1/Figure 4-figure suplemment 1-C-5-source data1.tif]

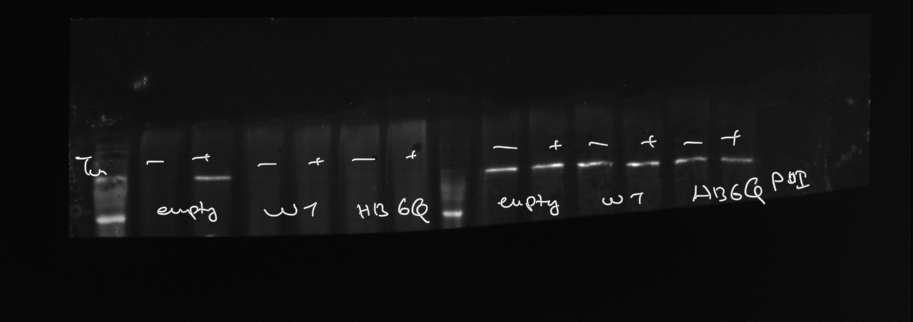

Supplement: Figure 4—figure supplement 1—source data 2. [file elife-102658-fig4-figsupp1-data2.zip › Figure 4-figure suplement 1-source data1/Figure 4-figure suplemment 1-B-5-source data1.tif]

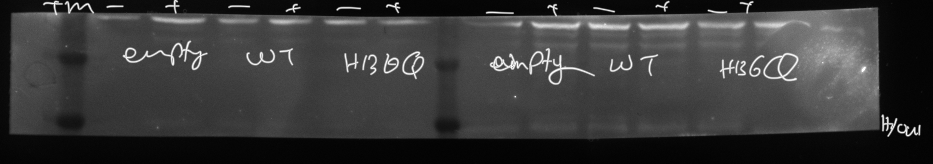

Supplement: Figure 4—figure supplement 1—source data 2. [file elife-102658-fig4-figsupp1-data2.zip › Figure 4-figure suplement 1-source data1/Figure 4-figure suplemment 1-C-2-source data1.tif]

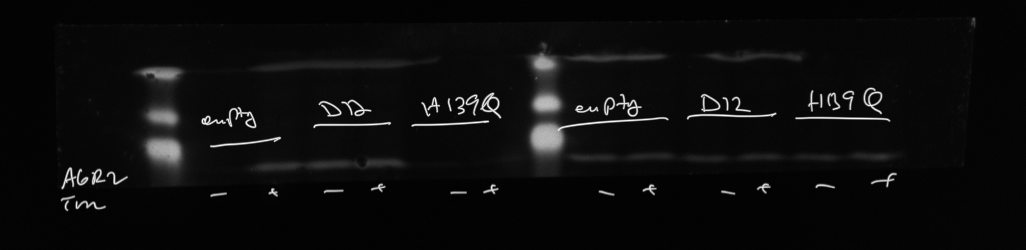

Supplement: Figure 4—figure supplement 1—source data 2. [file elife-102658-fig4-figsupp1-data2.zip › Figure 4-figure suplement 1-source data1/Figure 4-figure suplemment 1-A-1-source data1.tif]

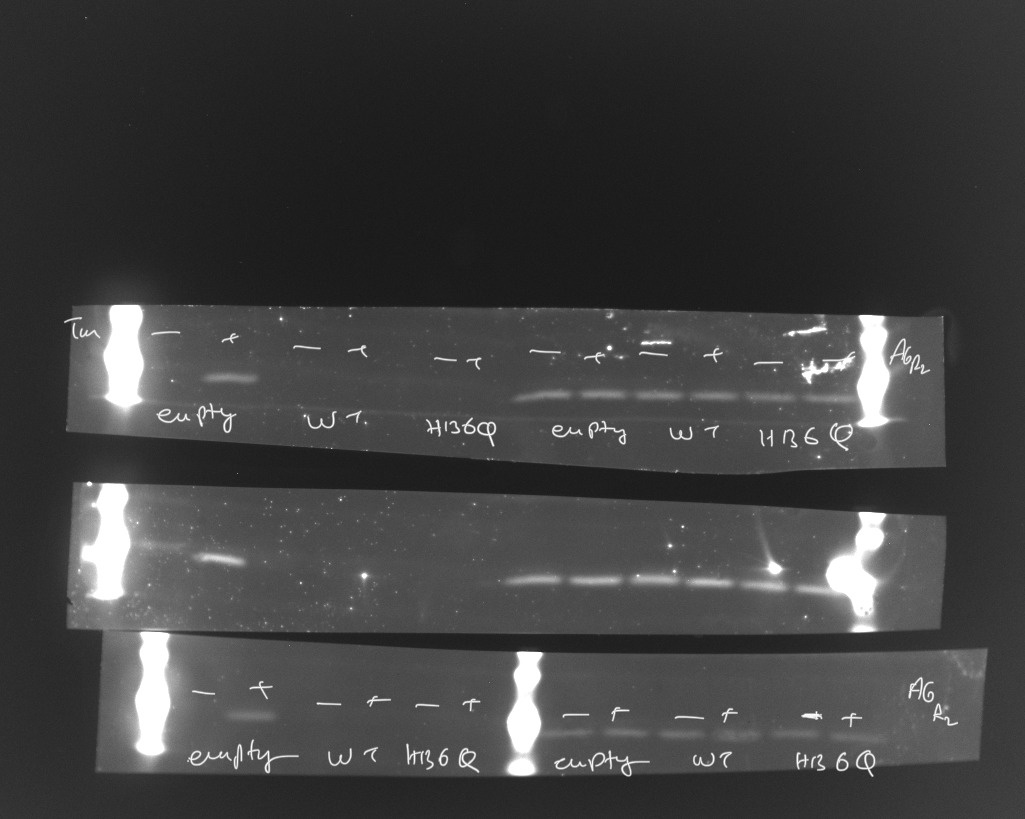

Supplement: Figure 4—figure supplement 1—source data 2. [file elife-102658-fig4-figsupp1-data2.zip › Figure 4-figure suplement 1-source data1/Figure 4-figure suplemment 1-B-1-source data1.tif]

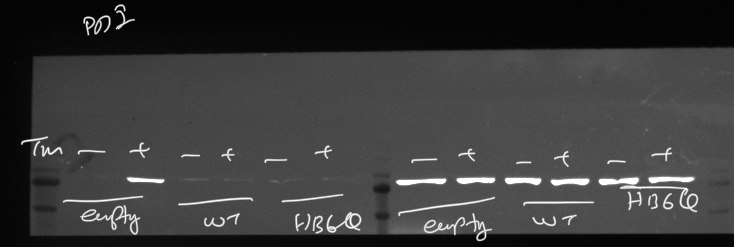

Supplement: Figure 4—figure supplement 1—source data 2. [file elife-102658-fig4-figsupp1-data2.zip › Figure 4-figure suplement 1-source data1/Figure 4-figure suplemment 1-B-4-source data1.tif]

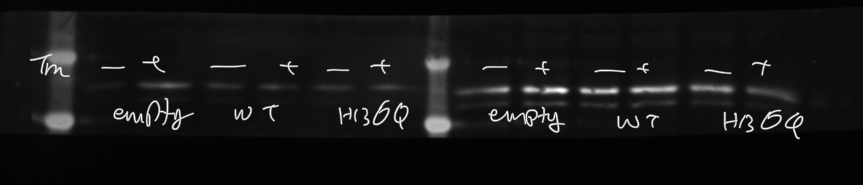

Supplement: Figure 4—figure supplement 1—source data 2. [file elife-102658-fig4-figsupp1-data2.zip › Figure 4-figure suplement 1-source data1/Figure 4-figure suplemment 1-C-3-source data1.tif]

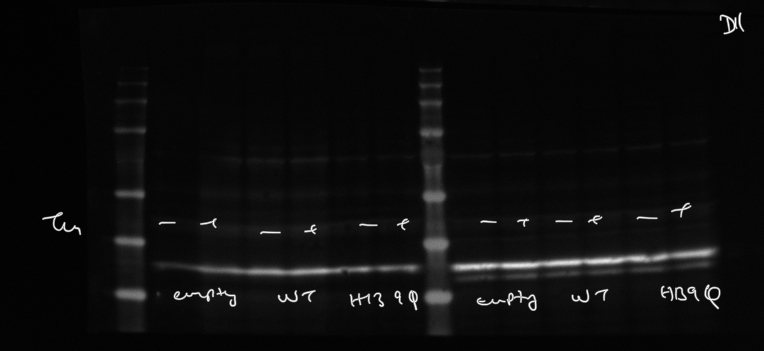

Supplement: Figure 4—figure supplement 1—source data 2. [file elife-102658-fig4-figsupp1-data2.zip › Figure 4-figure suplement 1-source data1/Figure 4-figure suplemment 1-A-3-source data1.tiff]

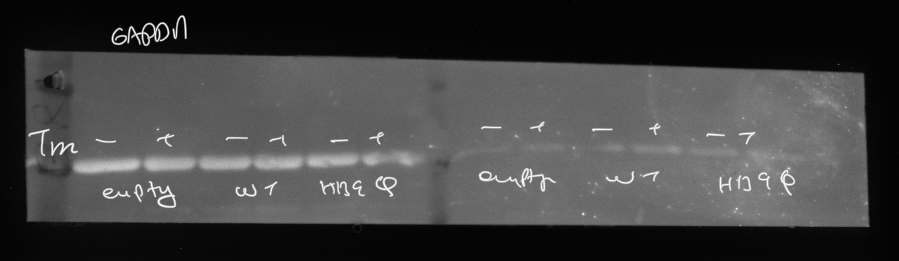

Supplement: Figure 4—figure supplement 1—source data 2. [file elife-102658-fig4-figsupp1-data2.zip › Figure 4-figure suplement 1-source data1/Figure 4-figure suplemment 1-A-4-source data1.tif]

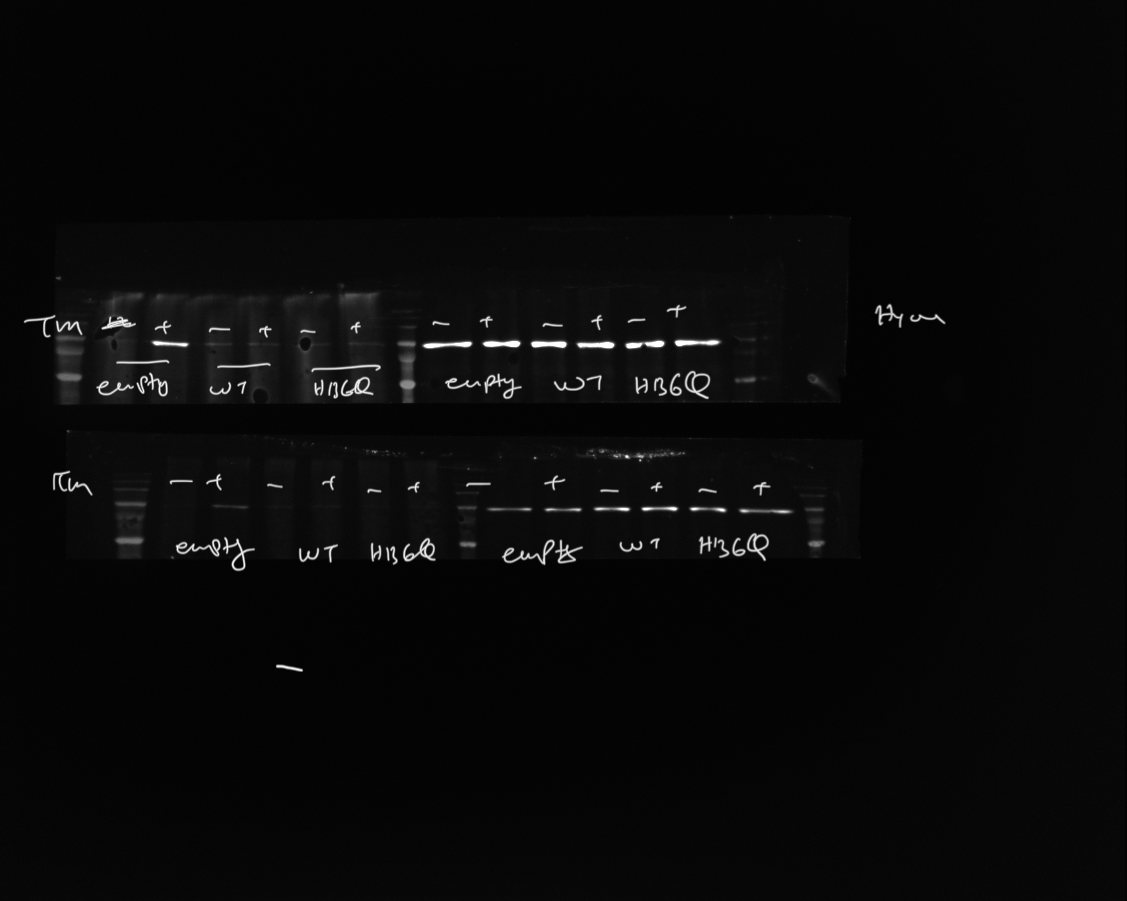

Supplement: Figure 4—figure supplement 1—source data 2. [file elife-102658-fig4-figsupp1-data2.zip › Figure 4-figure suplement 1-source data1/Figure 4-figure suplemment 1-B-3-source data1.tif]

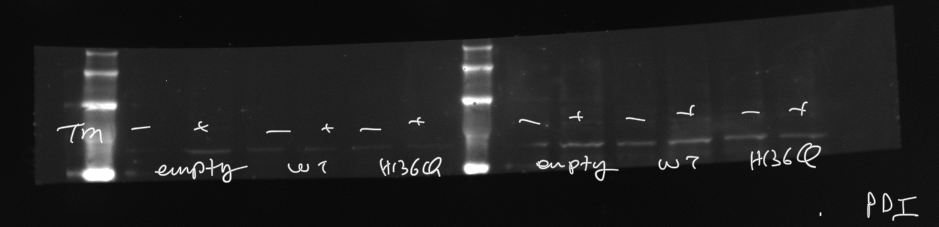

Supplement: Figure 4—figure supplement 1—source data 2. [file elife-102658-fig4-figsupp1-data2.zip › Figure 4-figure suplement 1-source data1/Figure 4-figure suplemment 1-C-4-source data1.tif]

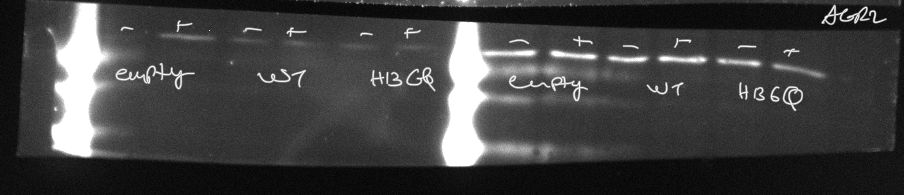

Supplement: Figure 4—figure supplement 1—source data 2. [file elife-102658-fig4-figsupp1-data2.zip › Figure 4-figure suplement 1-source data1/Figure 4-figure suplemment 1-C-1-source data1.tif]

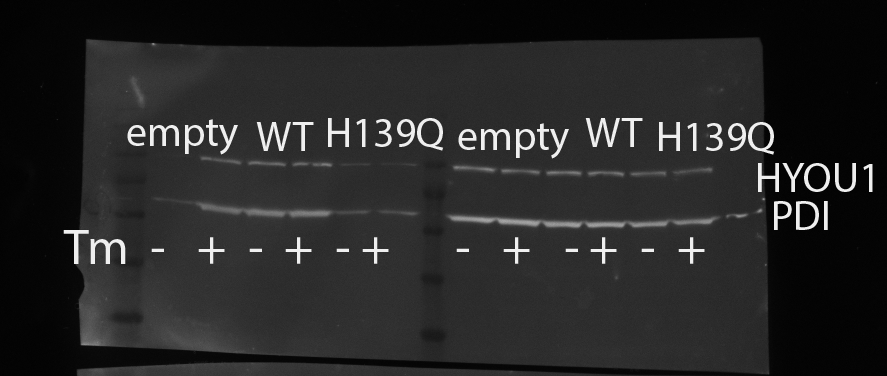

Supplement: Figure 4—figure supplement 1—source data 2. [file elife-102658-fig4-figsupp1-data2.zip › Figure 4-figure suplement 1-source data1/Figure 4-figure suplemment 1-A-2-source data1.tif]

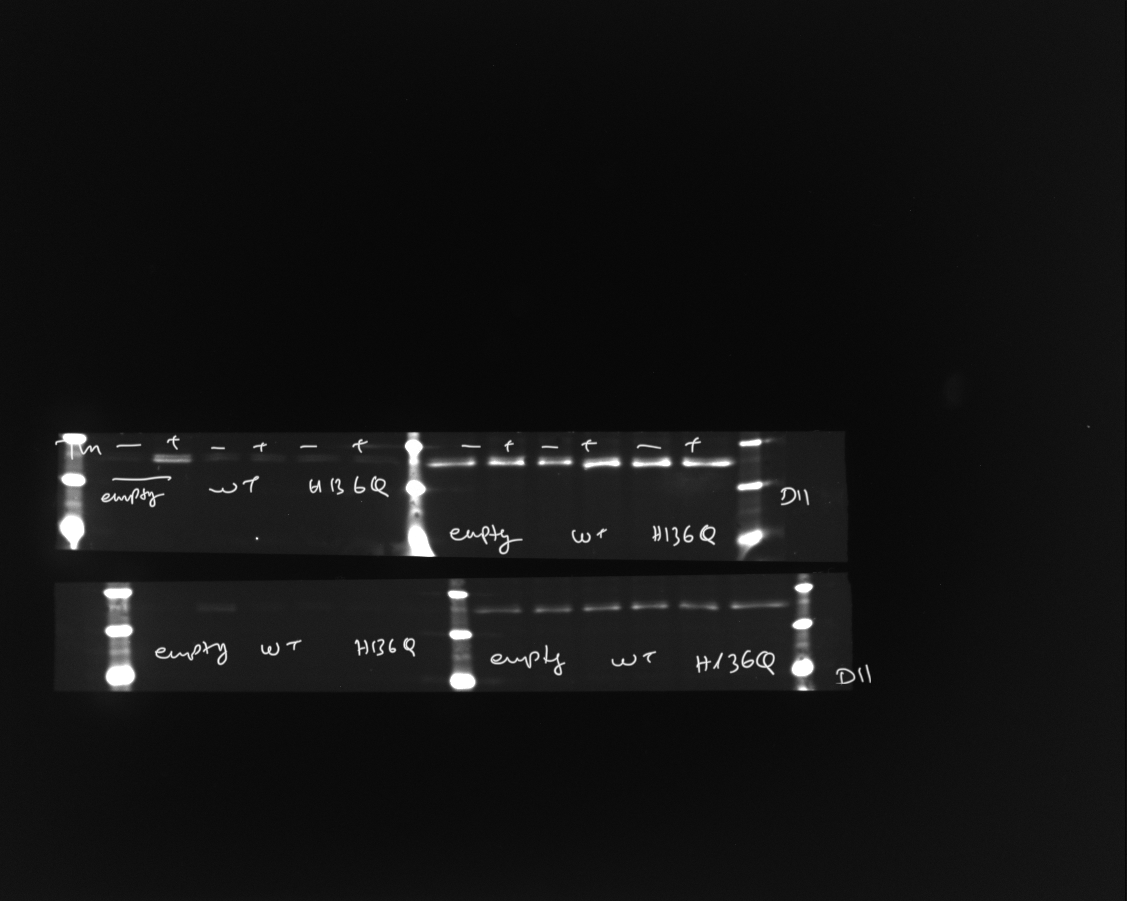

Supplement: Figure 4—figure supplement 1—source data 2. [file elife-102658-fig4-figsupp1-data2.zip › Figure 4-figure suplement 1-source data1/Figure 4-figure suplemment 1-B-2-source data1.tif]

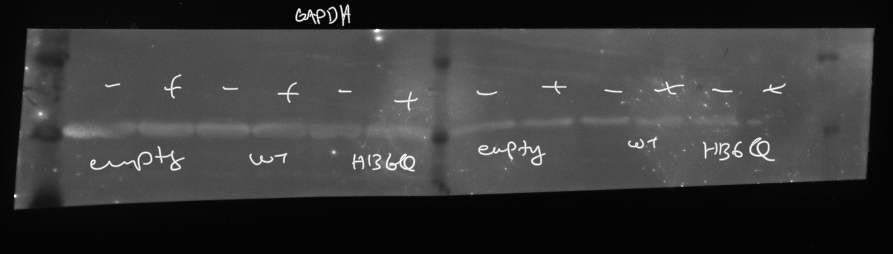

Supplement: Figure 4—figure supplement 1—source data 2. [file elife-102658-fig4-figsupp1-data2.zip › Figure 4-figure suplement 1-source data1/Figure 4-figure suplemment 1-C-5-source data1.tif]

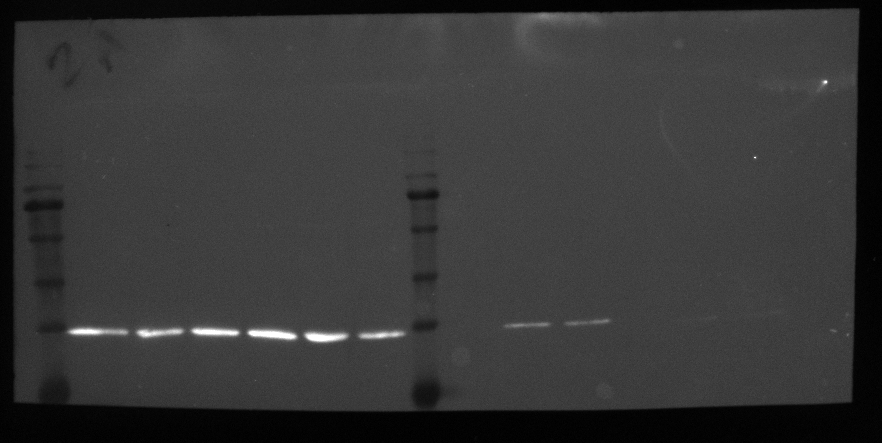

Supplement: Figure 5—source data 1. [file elife-102658-fig5-data1.zip › Figure 5-source data1/Figure 5D-4-source data1.tif]

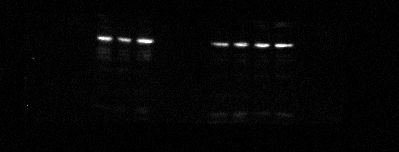

Supplement: Figure 5—source data 1. [file elife-102658-fig5-data1.zip › Figure 5-source data1/Figure 5B-1-source data1.tif]

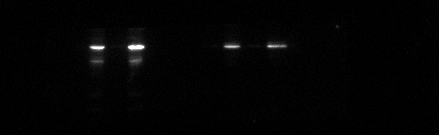

Supplement: Figure 5—source data 1. [file elife-102658-fig5-data1.zip › Figure 5-source data1/Figure 5A-1-source data1.tif]

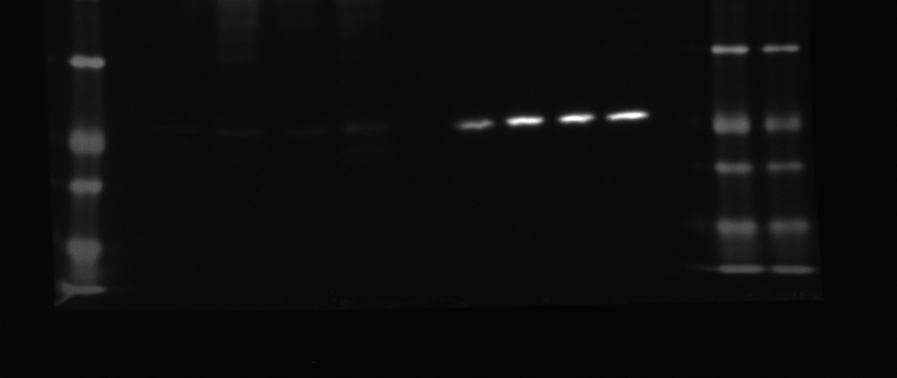

Supplement: Figure 5—source data 1. [file elife-102658-fig5-data1.zip › Figure 5-source data1/Figure 5C-2-source data1.tif]

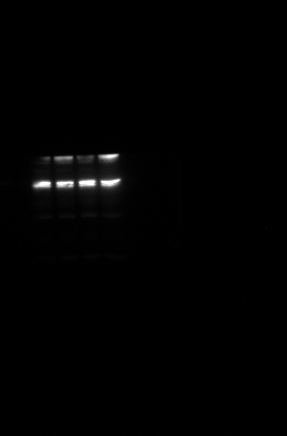

Supplement: Figure 5—source data 1. [file elife-102658-fig5-data1.zip › Figure 5-source data1/Figure 5B-5-source data1.tif]

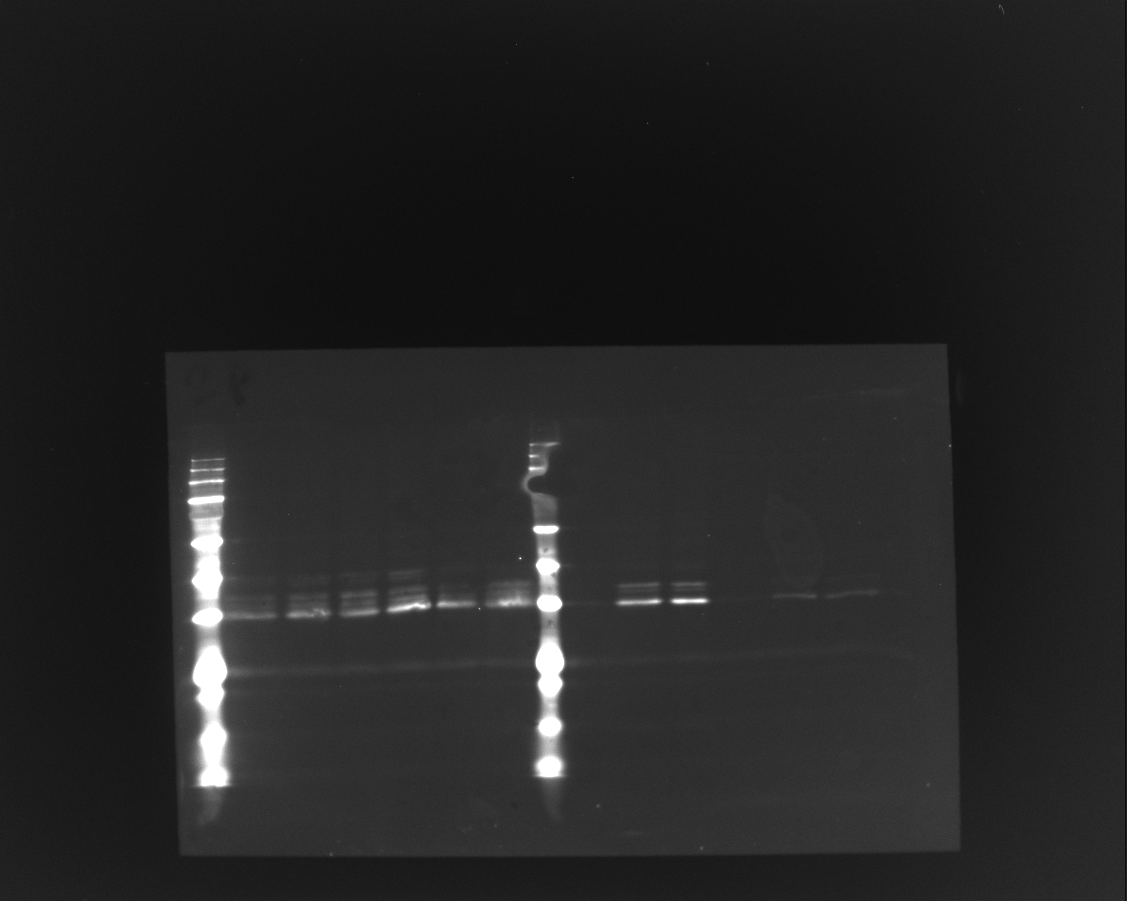

Supplement: Figure 5—source data 1. [file elife-102658-fig5-data1.zip › Figure 5-source data1/Figure 5D-5-source data1.tif]

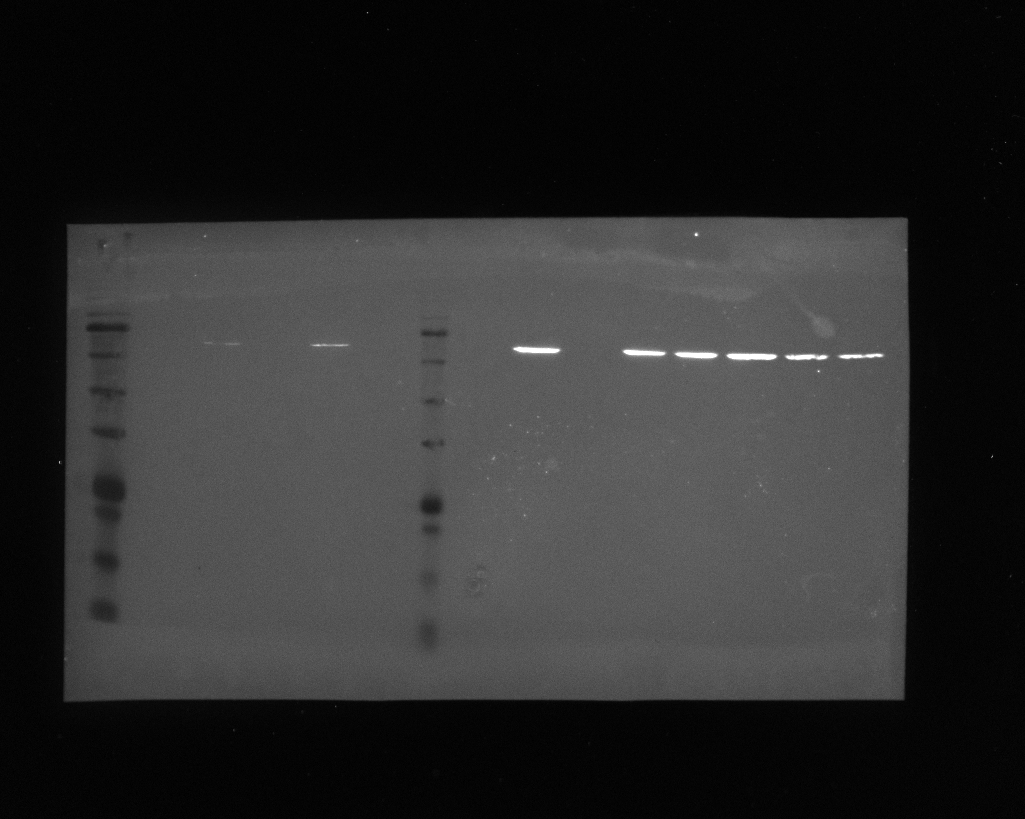

Supplement: Figure 5—source data 1. [file elife-102658-fig5-data1.zip › Figure 5-source data1/Figure 5A-4-source data1.tif]

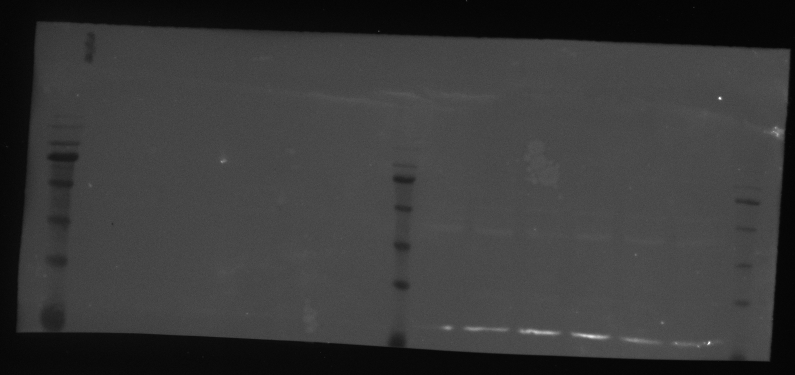

Supplement: Figure 5—source data 1. [file elife-102658-fig5-data1.zip › Figure 5-source data1/Figure 5D-1-source data.tif]

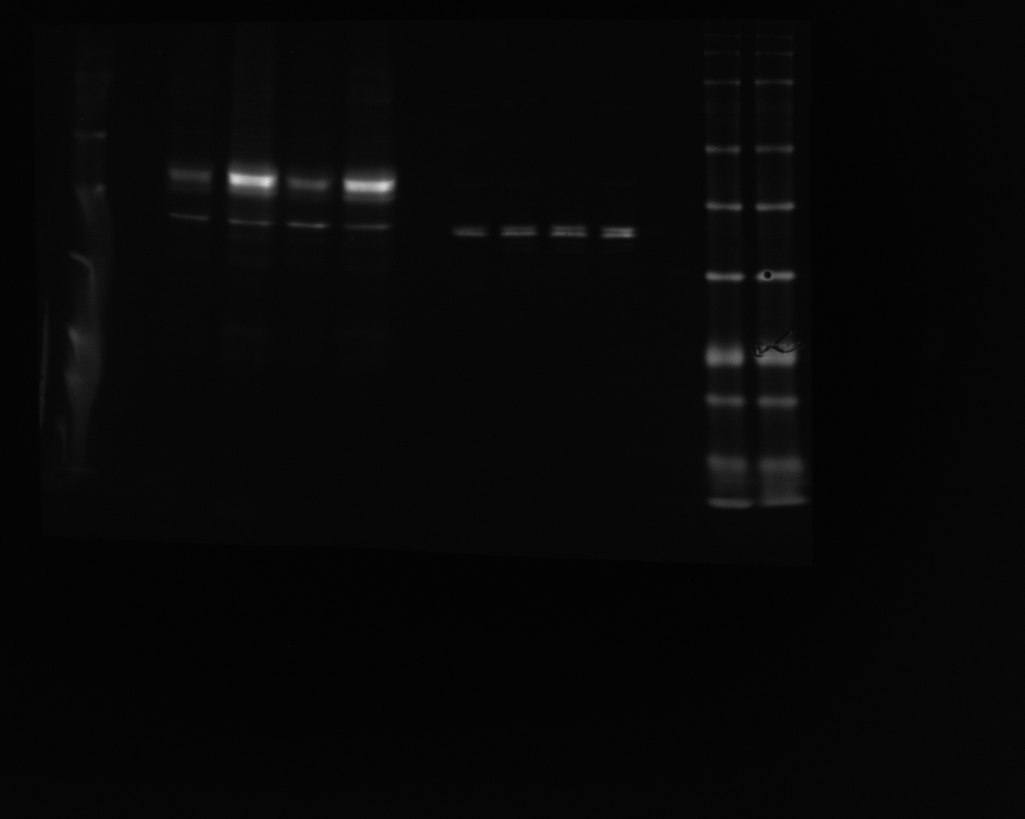

Supplement: Figure 5—source data 1. [file elife-102658-fig5-data1.zip › Figure 5-source data1/Figure 5C-3-source data1.tif]

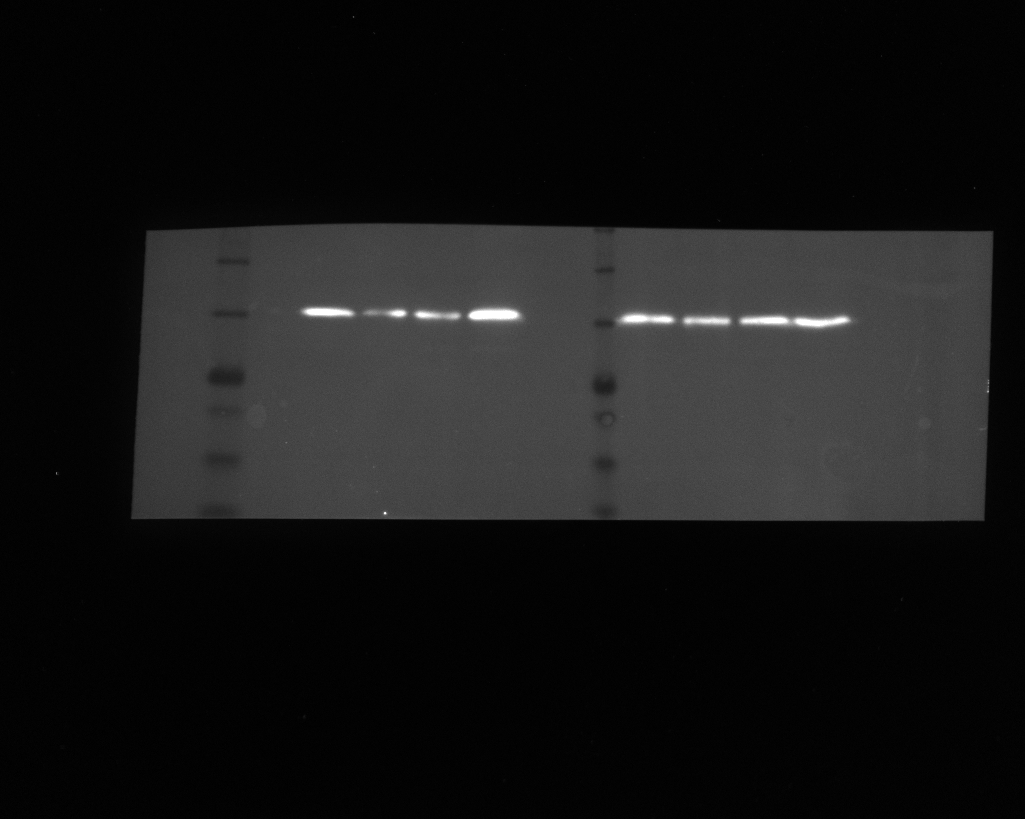

Supplement: Figure 5—source data 1. [file elife-102658-fig5-data1.zip › Figure 5-source data1/Figure 5B-4-source data1.tif]

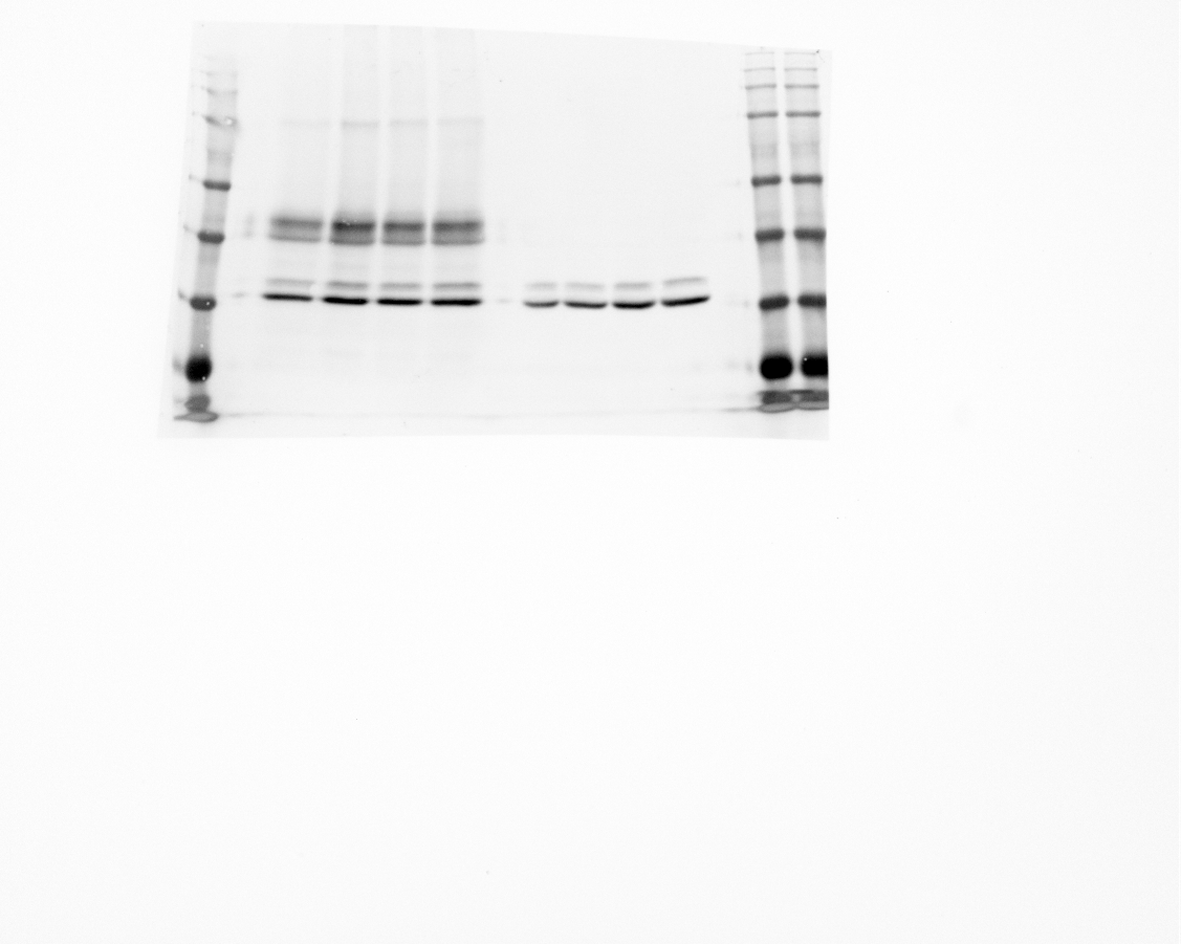

Supplement: Figure 5—source data 1. [file elife-102658-fig5-data1.zip › Figure 5-source data1/Figure 5C-4-source data1.tif]

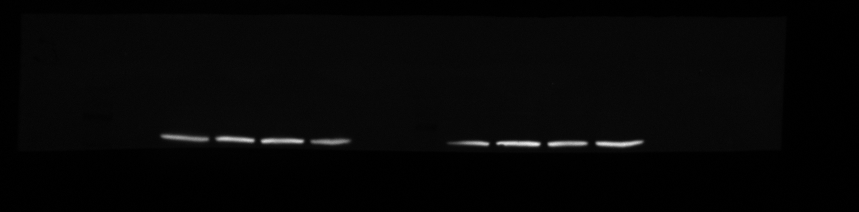

Supplement: Figure 5—source data 1. [file elife-102658-fig5-data1.zip › Figure 5-source data1/Figure 5B-3-source data1.tif]

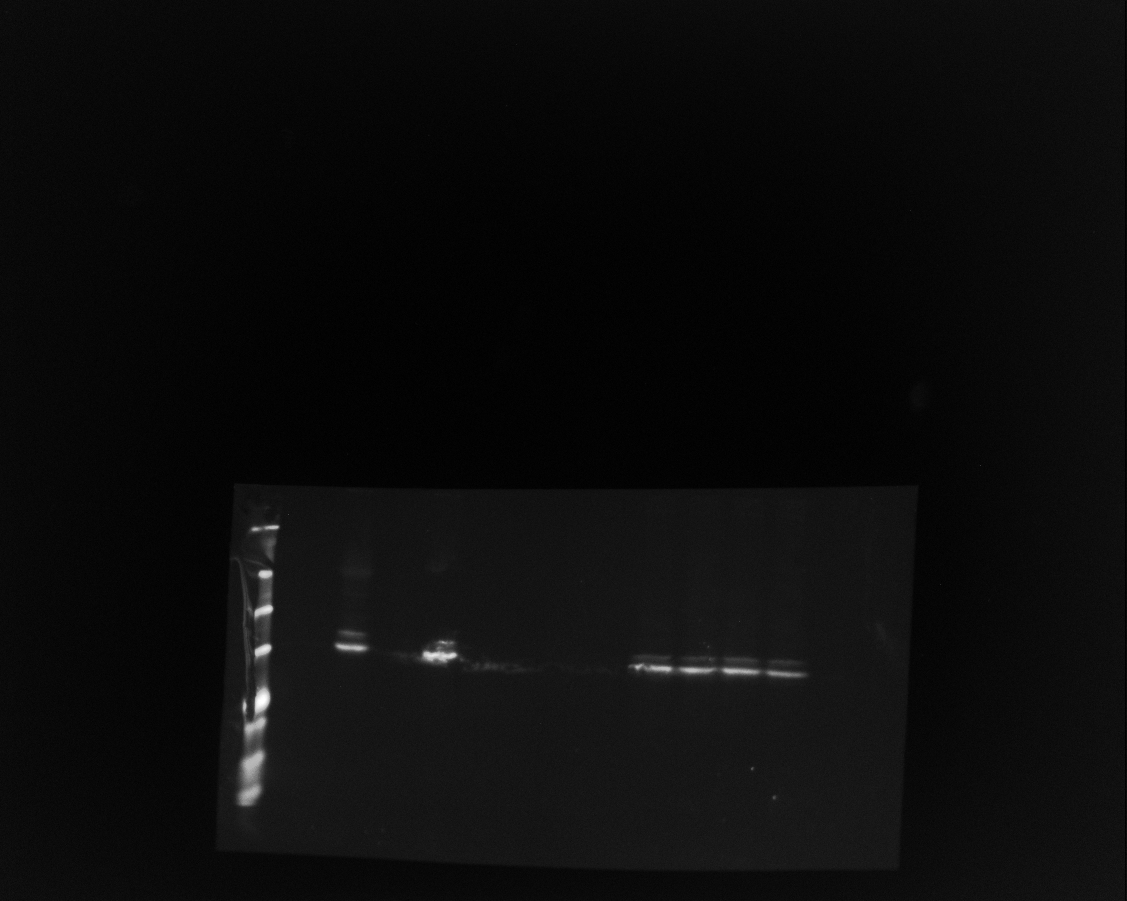

Supplement: Figure 5—source data 1. [file elife-102658-fig5-data1.zip › Figure 5-source data1/Figure 5A-3-source data1.tif]

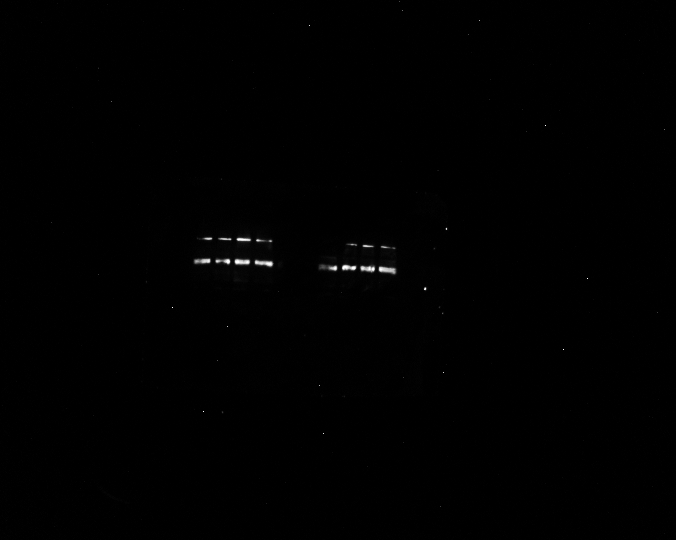

Supplement: Figure 5—source data 1. [file elife-102658-fig5-data1.zip › Figure 5-source data1/Figure 5B-2-source data1.tif]

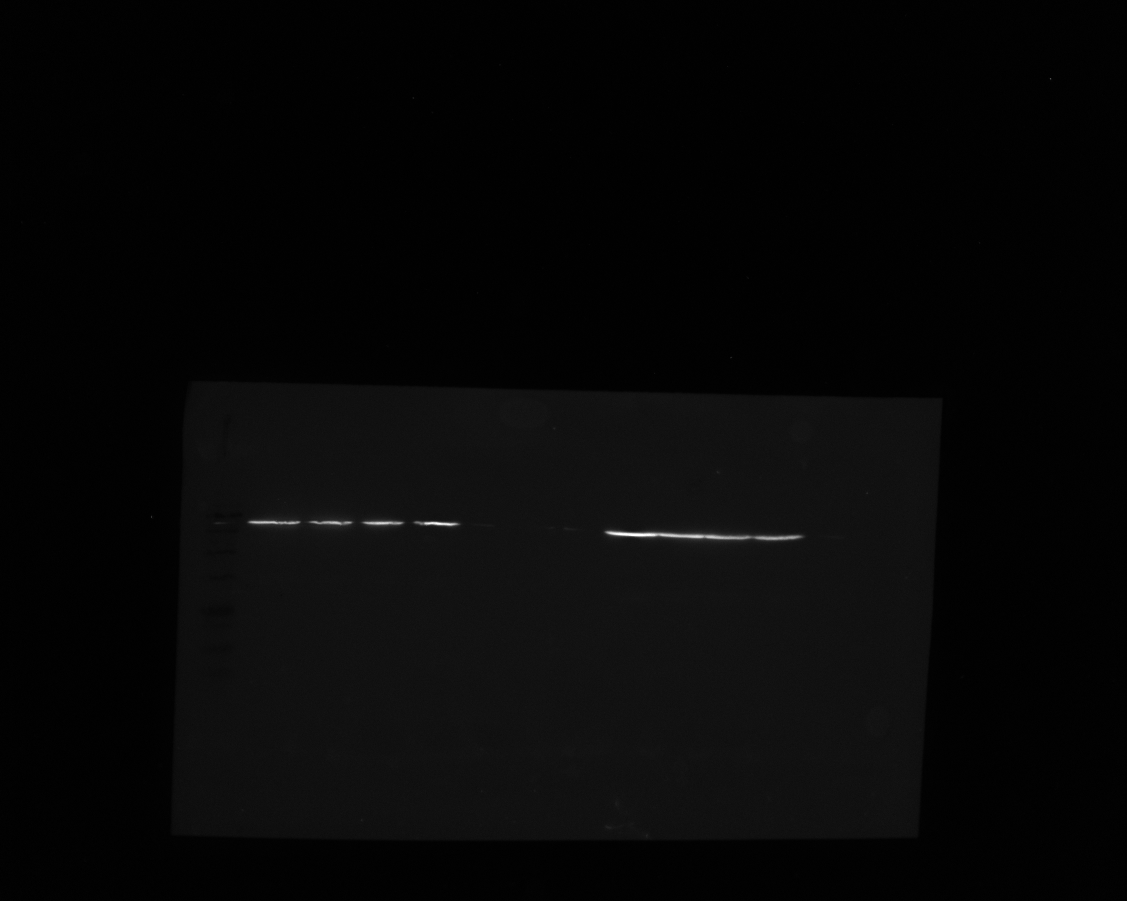

Supplement: Figure 5—source data 1. [file elife-102658-fig5-data1.zip › Figure 5-source data1/Figure 5A-5-source data.tif]

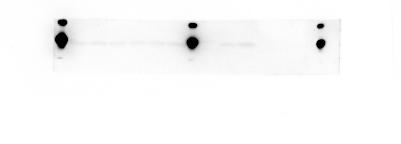

Supplement: Figure 5—source data 1. [file elife-102658-fig5-data1.zip › Figure 5-source data1/Figure 5D-3-source data1.tif]

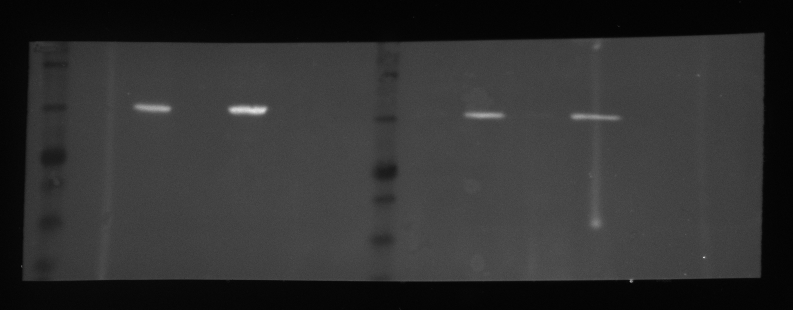

Supplement: Figure 5—source data 1. [file elife-102658-fig5-data1.zip › Figure 5-source data1/Figure 5A-2-source data1.tif]

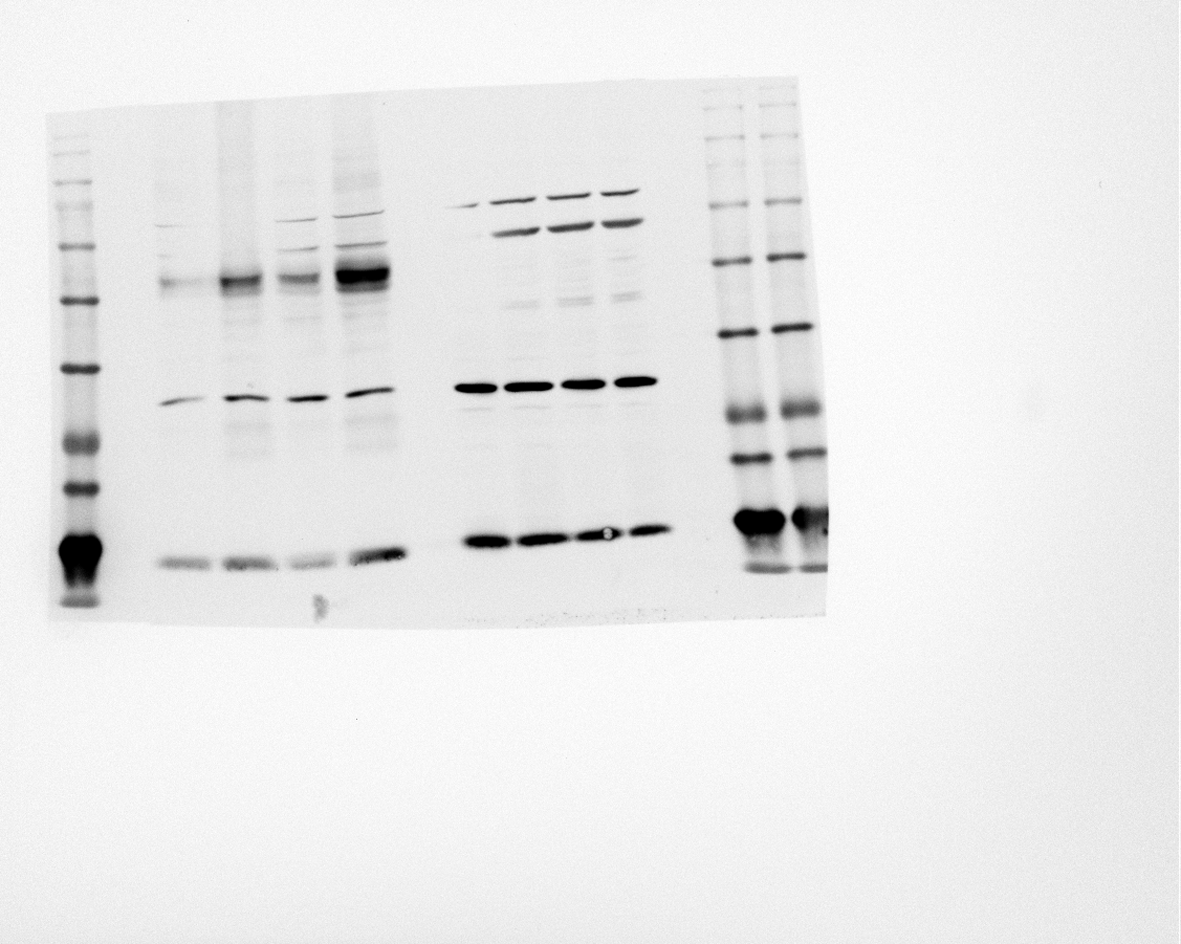

Supplement: Figure 5—source data 1. [file elife-102658-fig5-data1.zip › Figure 5-source data1/Figure 5C-1-source data1.tif]

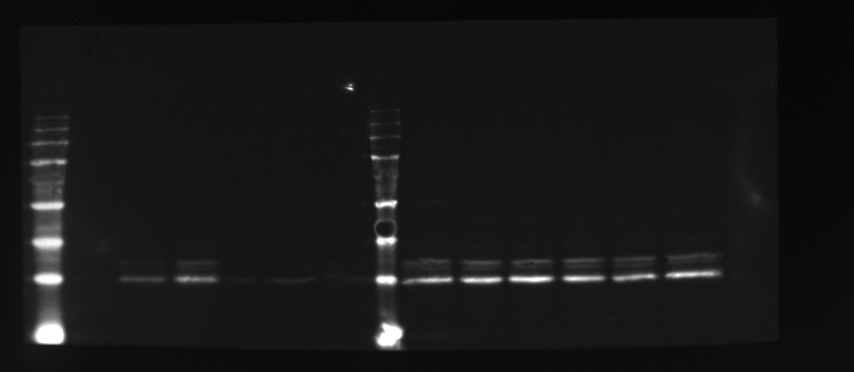

Supplement: Figure 5—source data 1. [file elife-102658-fig5-data1.zip › Figure 5-source data1/Figure 5D-2-source data.tif]

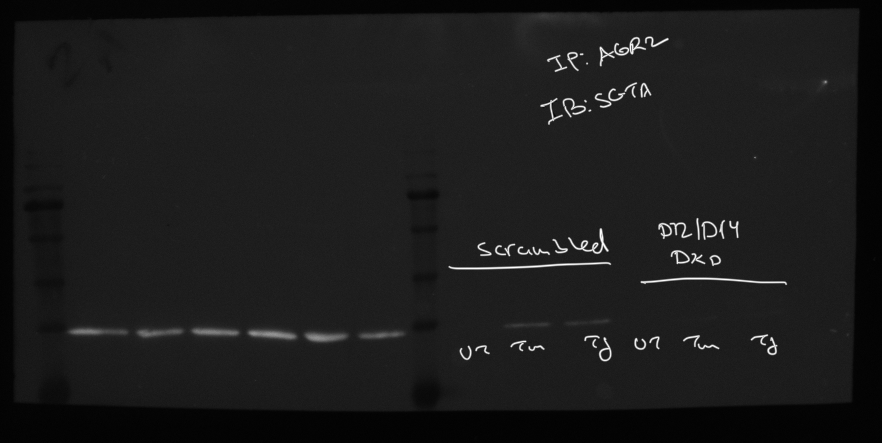

Supplement: Figure 5—source data 2. [file elife-102658-fig5-data2.zip › Figure 5-source data1/Figure 5D-4-source data1.tif]

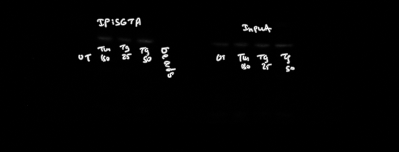

Supplement: Figure 5—source data 2. [file elife-102658-fig5-data2.zip › Figure 5-source data1/Figure 5B-1-source data1.tif]

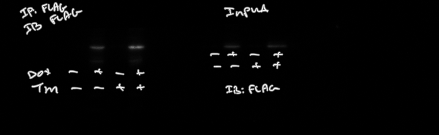

Supplement: Figure 5—source data 2. [file elife-102658-fig5-data2.zip › Figure 5-source data1/Figure 5A-1-source data1.tif]

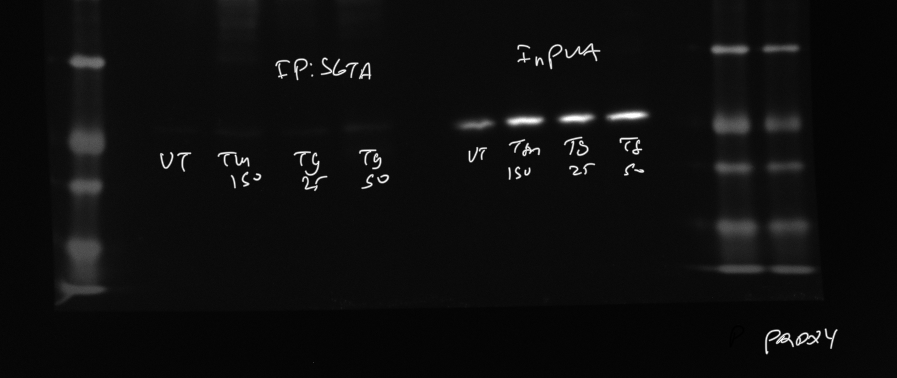

Supplement: Figure 5—source data 2. [file elife-102658-fig5-data2.zip › Figure 5-source data1/Figure 5C-2-source data1.tif]

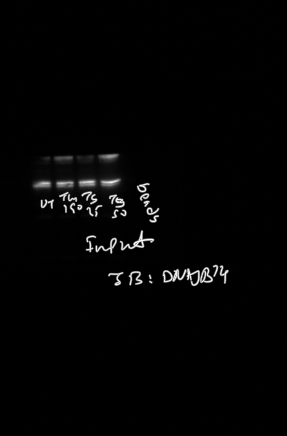

Supplement: Figure 5—source data 2. [file elife-102658-fig5-data2.zip › Figure 5-source data1/Figure 5B-5-source data1.tif]

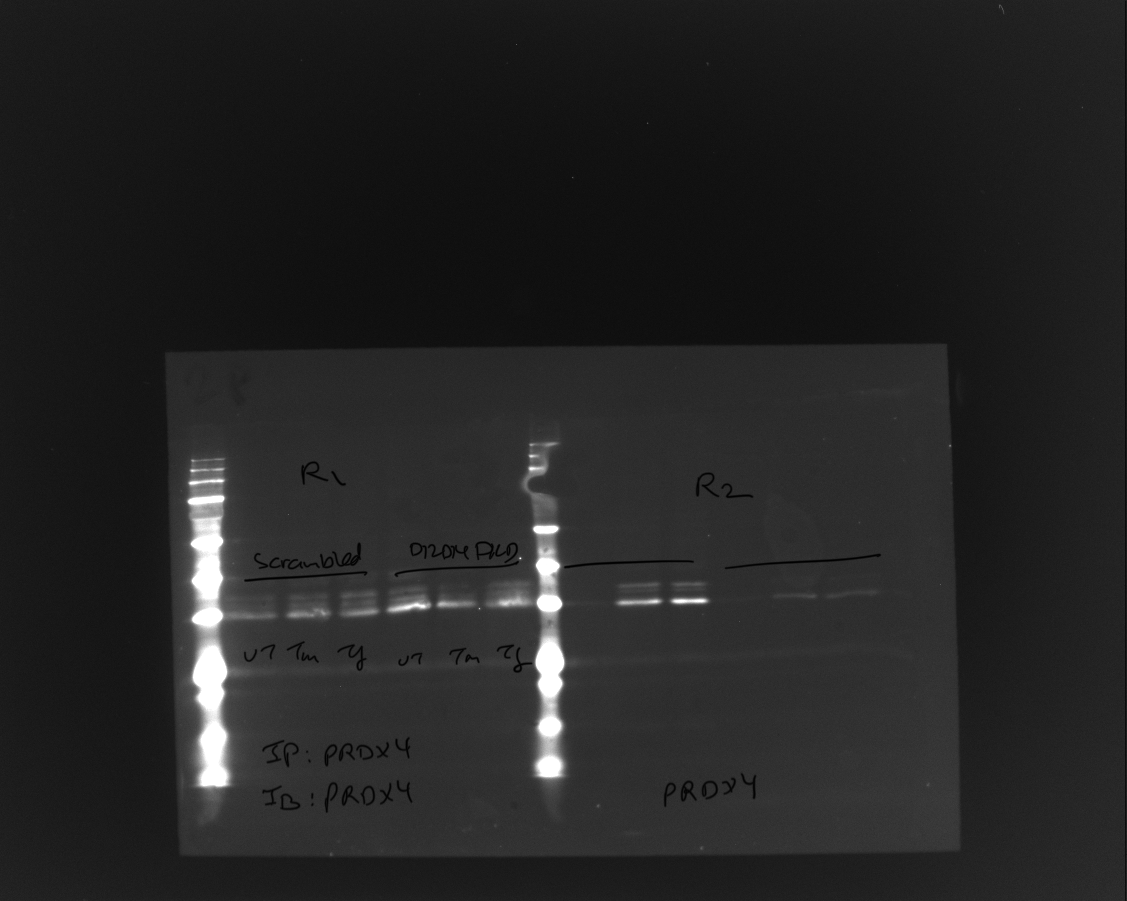

Supplement: Figure 5—source data 2. [file elife-102658-fig5-data2.zip › Figure 5-source data1/Figure 5D-5-source data1.tif]

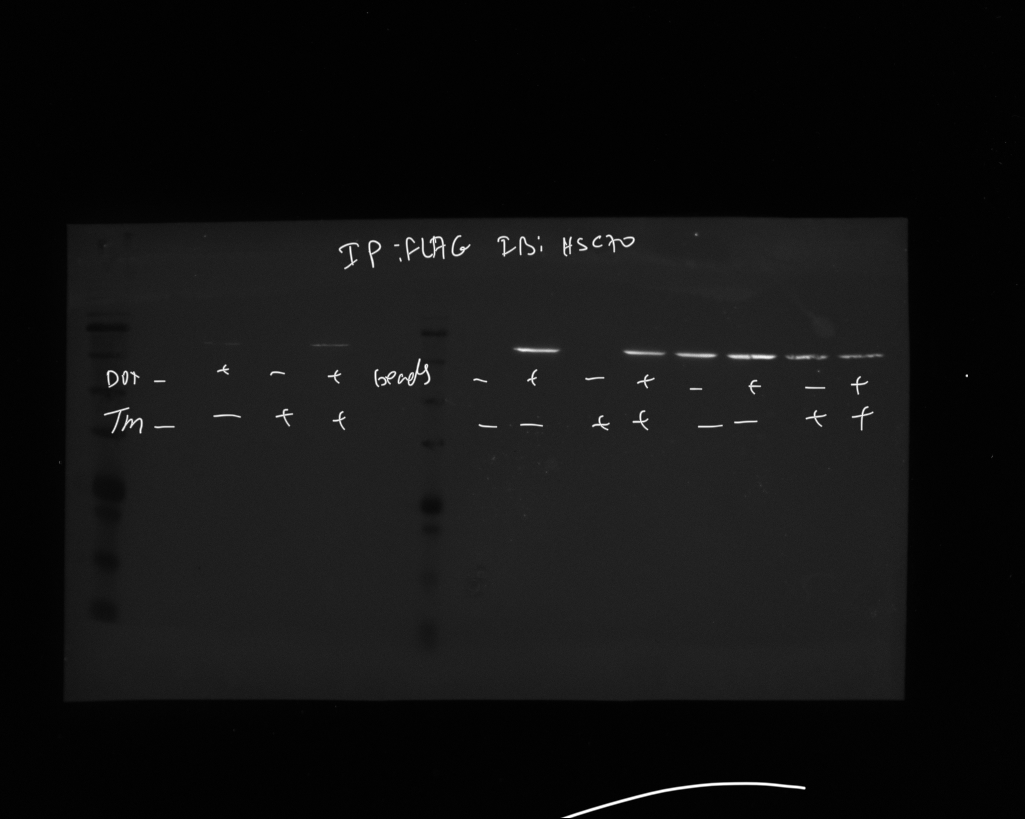

Supplement: Figure 5—source data 2. [file elife-102658-fig5-data2.zip › Figure 5-source data1/Figure 5A-4-source data1.tif]

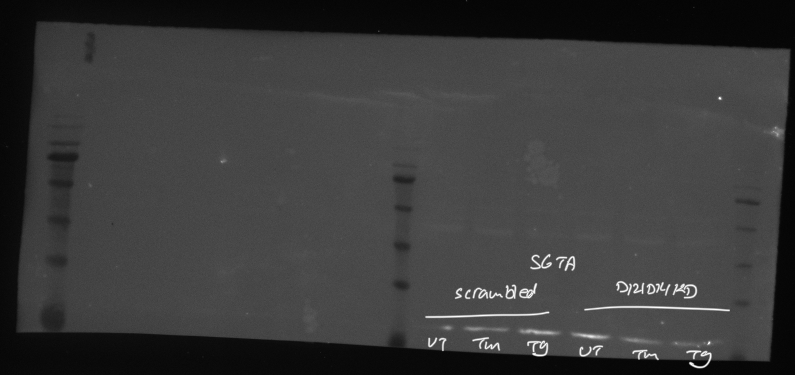

Supplement: Figure 5—source data 2. [file elife-102658-fig5-data2.zip › Figure 5-source data1/Figure 5D-1-source data.tif]

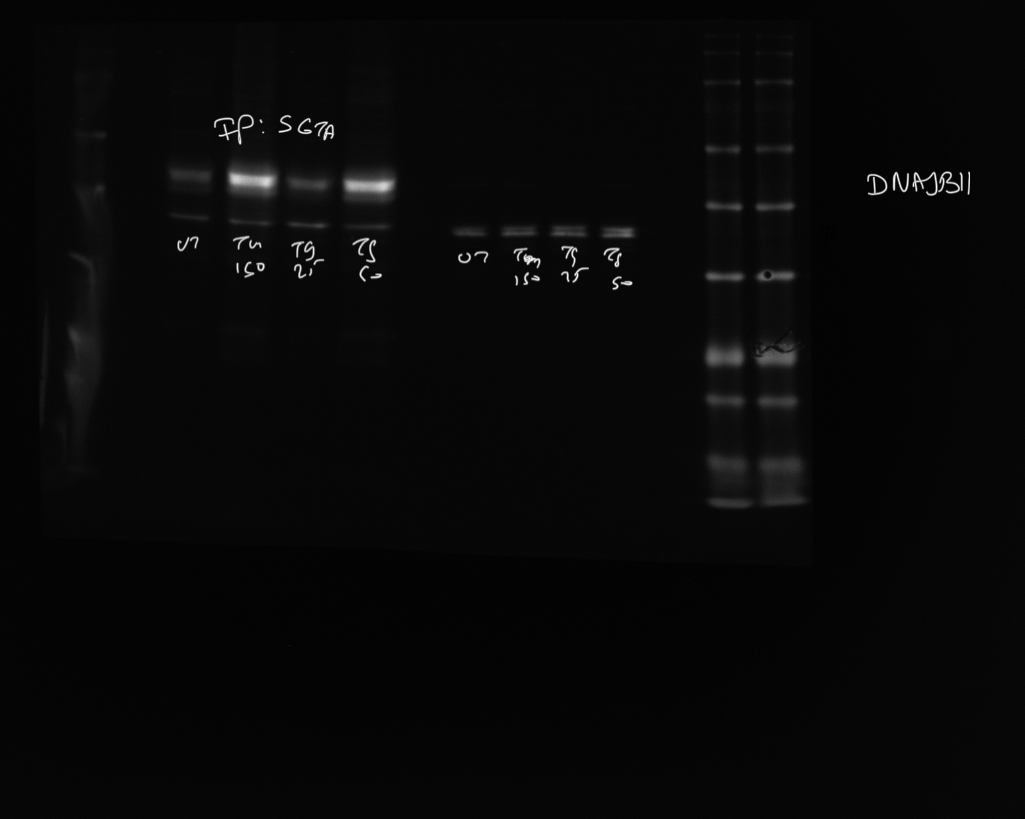

Supplement: Figure 5—source data 2. [file elife-102658-fig5-data2.zip › Figure 5-source data1/Figure 5C-3-source data1.tif]

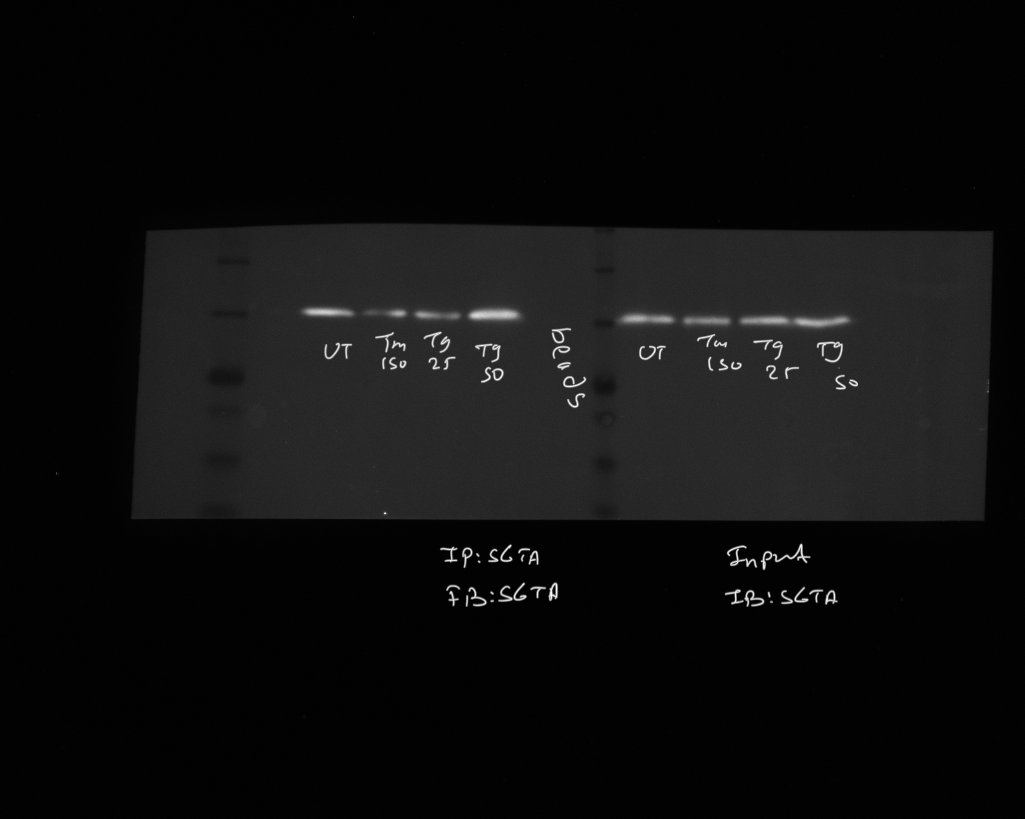

Supplement: Figure 5—source data 2. [file elife-102658-fig5-data2.zip › Figure 5-source data1/Figure 5B-4-source data1.tif]

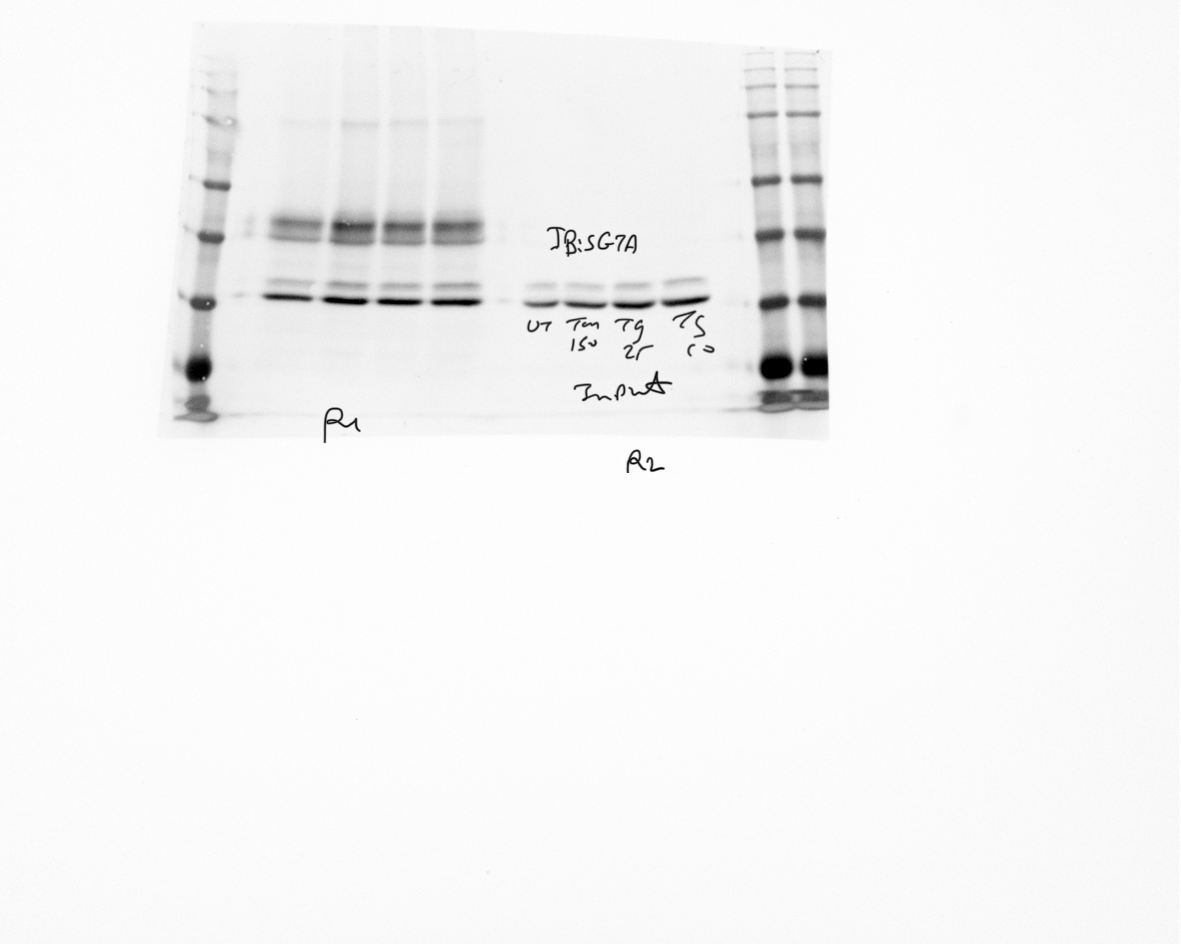

Supplement: Figure 5—source data 2. [file elife-102658-fig5-data2.zip › Figure 5-source data1/Figure 5C-4-source data1.tif]

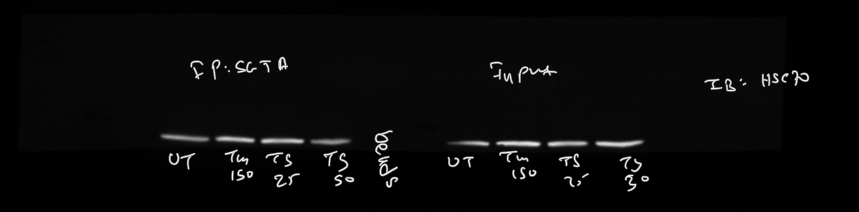

Supplement: Figure 5—source data 2. [file elife-102658-fig5-data2.zip › Figure 5-source data1/Figure 5B-3-source data1.tif]

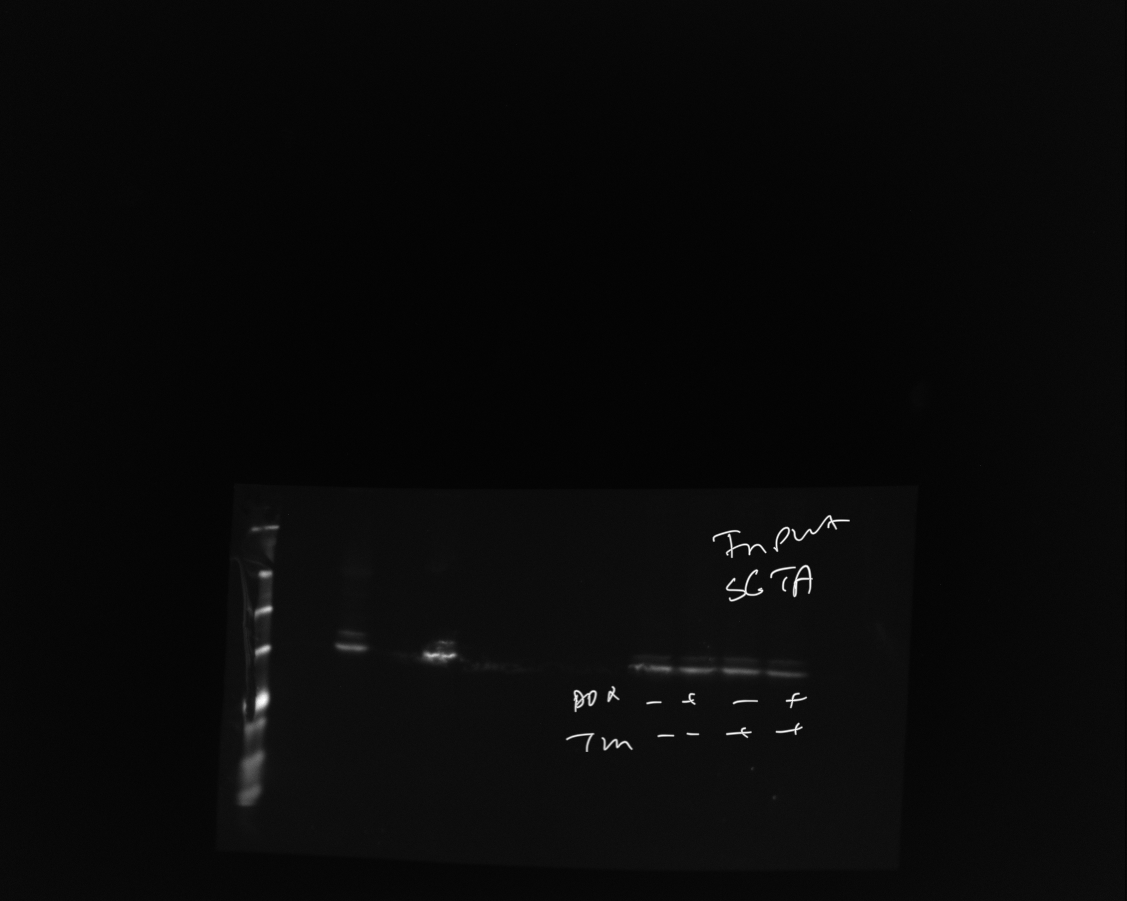

Supplement: Figure 5—source data 2. [file elife-102658-fig5-data2.zip › Figure 5-source data1/Figure 5A-3-source data1.tif]

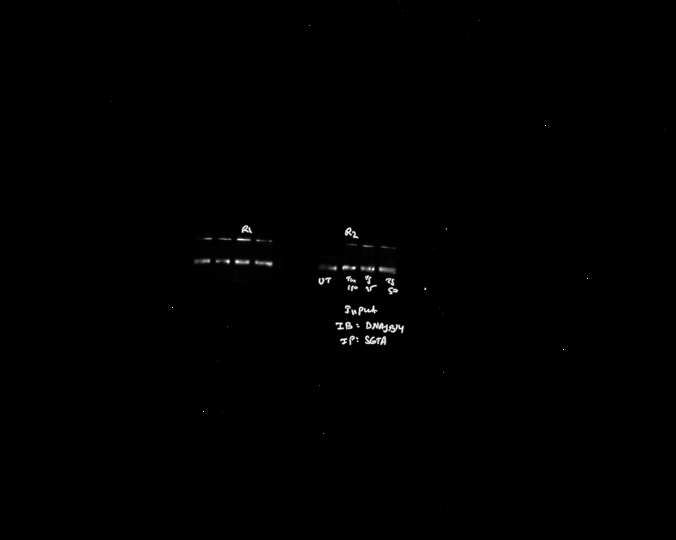

Supplement: Figure 5—source data 2. [file elife-102658-fig5-data2.zip › Figure 5-source data1/Figure 5B-2-source data1.tif]

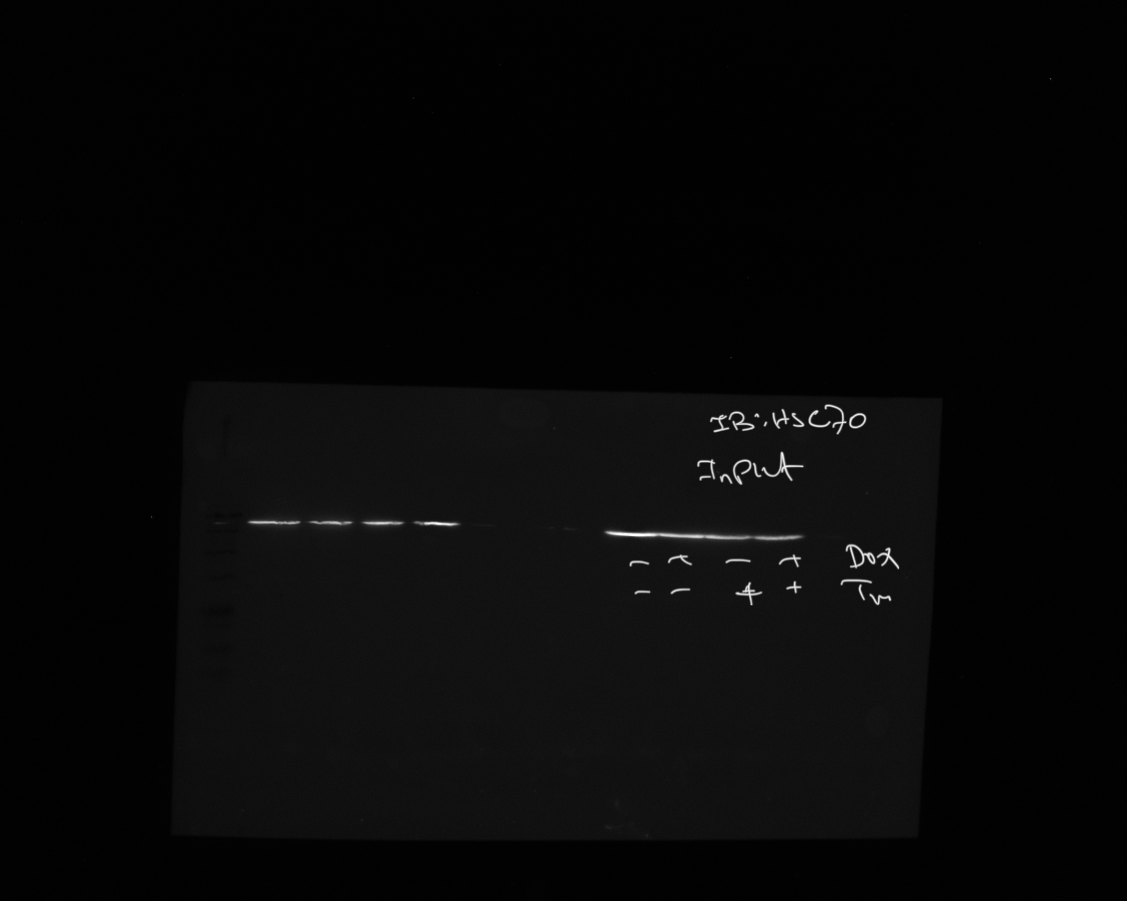

Supplement: Figure 5—source data 2. [file elife-102658-fig5-data2.zip › Figure 5-source data1/Figure 5A-5-source data.tif]

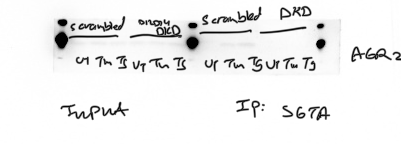

Supplement: Figure 5—source data 2. [file elife-102658-fig5-data2.zip › Figure 5-source data1/Figure 5D-3-source data1.tif]

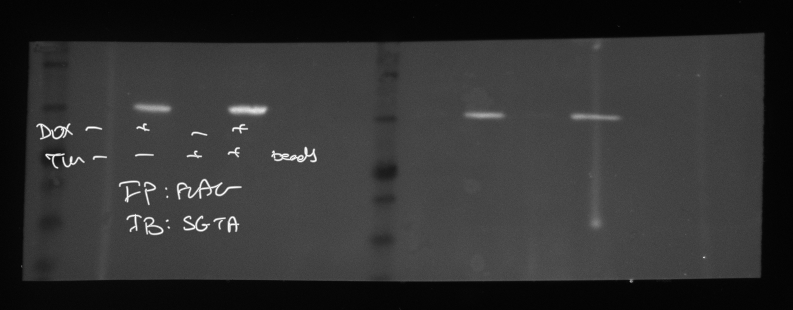

Supplement: Figure 5—source data 2. [file elife-102658-fig5-data2.zip › Figure 5-source data1/Figure 5A-2-source data1.tif]

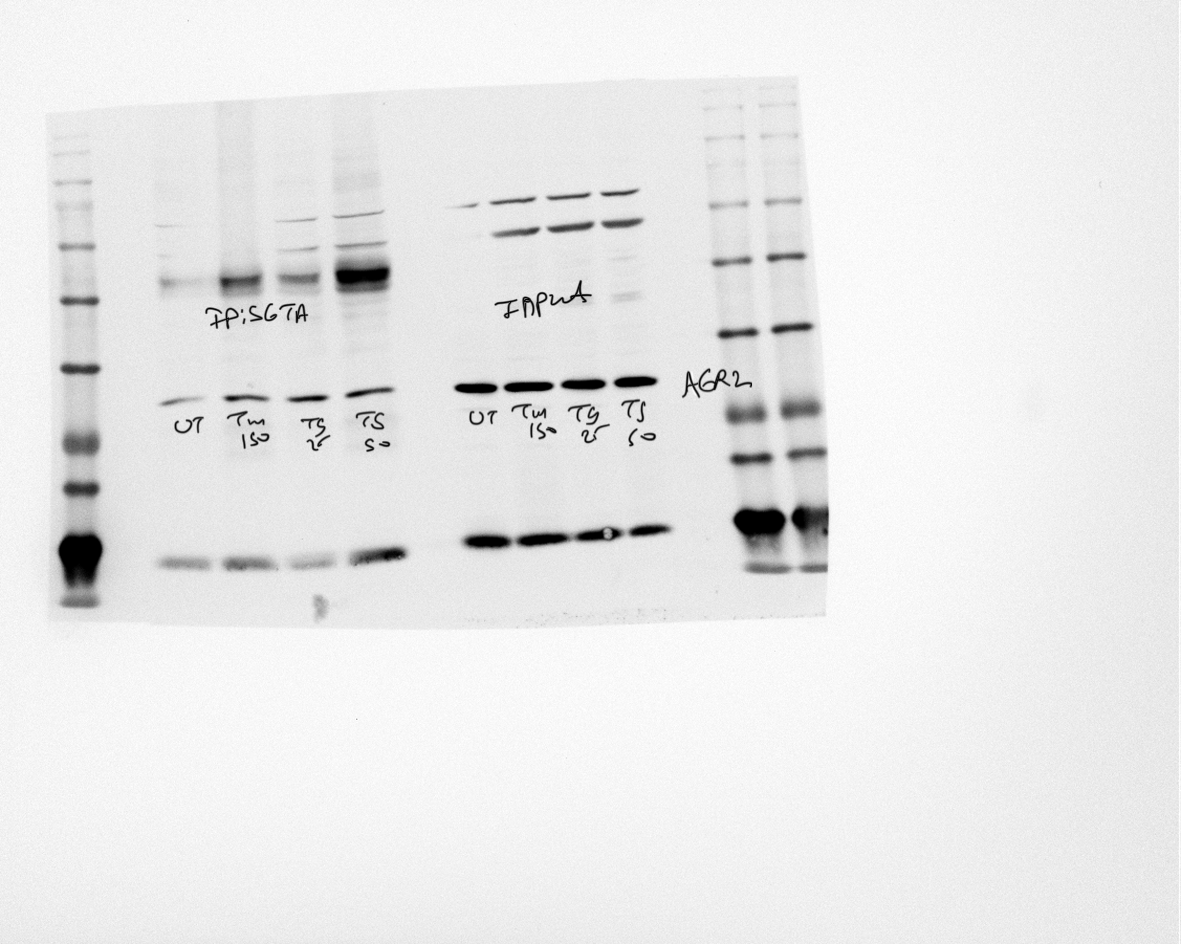

Supplement: Figure 5—source data 2. [file elife-102658-fig5-data2.zip › Figure 5-source data1/Figure 5C-1-source data1.tif]

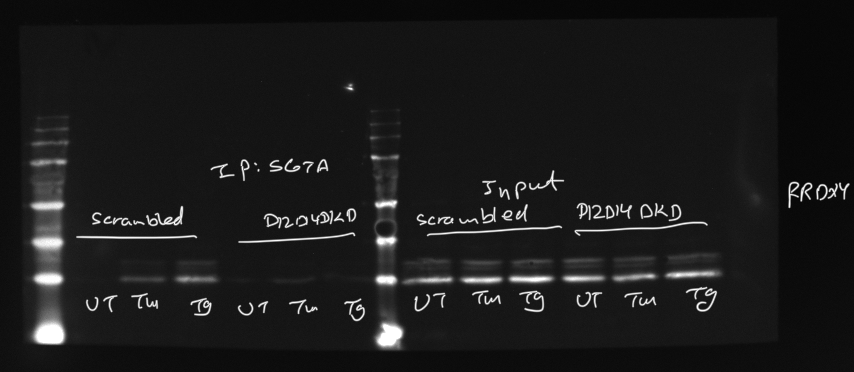

Supplement: Figure 5—source data 2. [file elife-102658-fig5-data2.zip › Figure 5-source data1/Figure 5D-2-source data.tif]

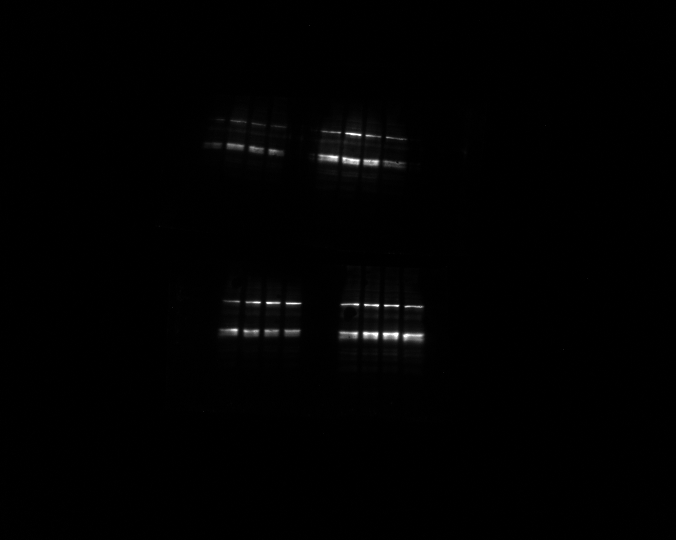

Supplement: Figure 5—figure supplement 1—source data 1. [file elife-102658-fig5-figsupp1-data1.zip › Figure 5-figure suplement 1-source data1/Figure 5-figure suplemment 1-D-6-source data1tif.tif]

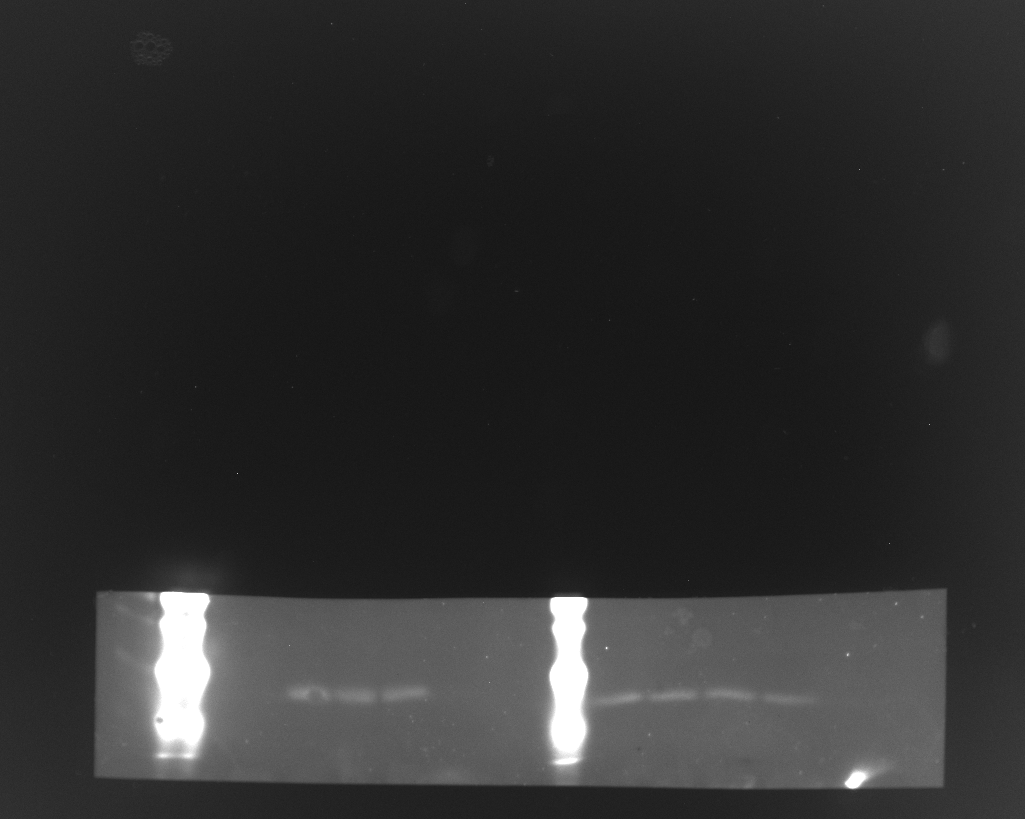

Supplement: Figure 5—figure supplement 1—source data 1. [file elife-102658-fig5-figsupp1-data1.zip › Figure 5-figure suplement 1-source data1/Figure 5-figure suplemment 1-E-1-source data1.tif]

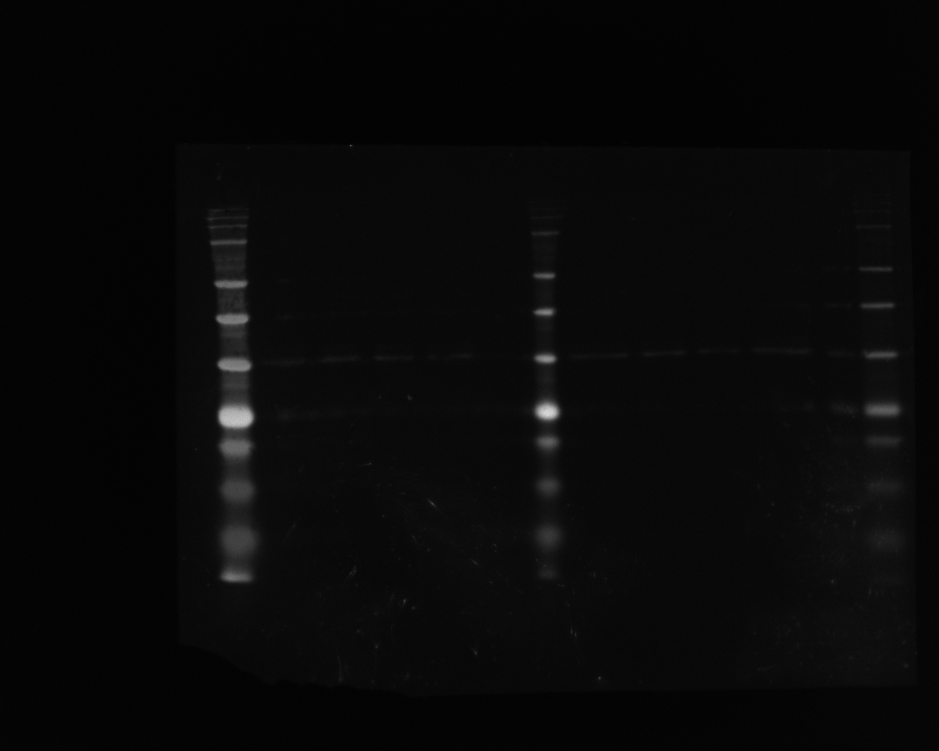

Supplement: Figure 5—figure supplement 1—source data 1. [file elife-102658-fig5-figsupp1-data1.zip › Figure 5-figure suplement 1-source data1/Figure 5-figure suplemment 1-C-4-source data1.tif]

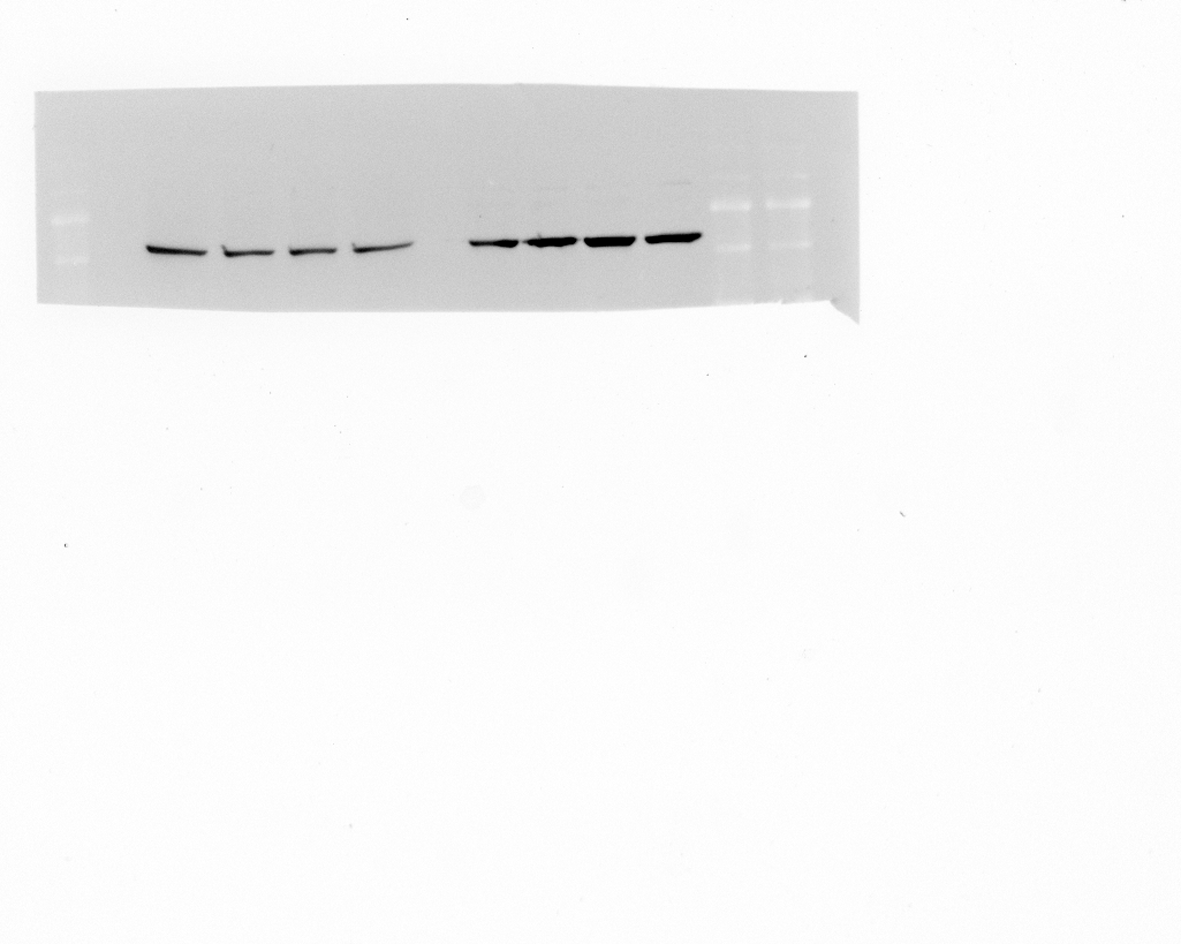

Supplement: Figure 5—figure supplement 1—source data 1. [file elife-102658-fig5-figsupp1-data1.zip › Figure 5-figure suplement 1-source data1/Figure 5-figure suplemment 1-B-3-source data1.tif]

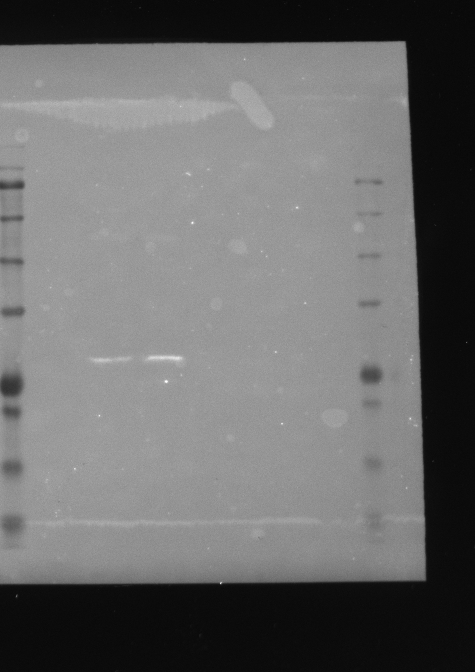

Supplement: Figure 5—figure supplement 1—source data 1. [file elife-102658-fig5-figsupp1-data1.zip › Figure 5-figure suplement 1-source data1/Figure 5-figure suplemment 1-F-1-source data1.tif]

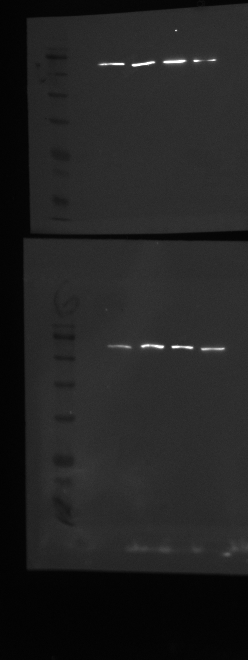

Supplement: Figure 5—figure supplement 1—source data 1. [file elife-102658-fig5-figsupp1-data1.zip › Figure 5-figure suplement 1-source data1/Figure 5-figure suplemment 1-D-2-source data1.tif]

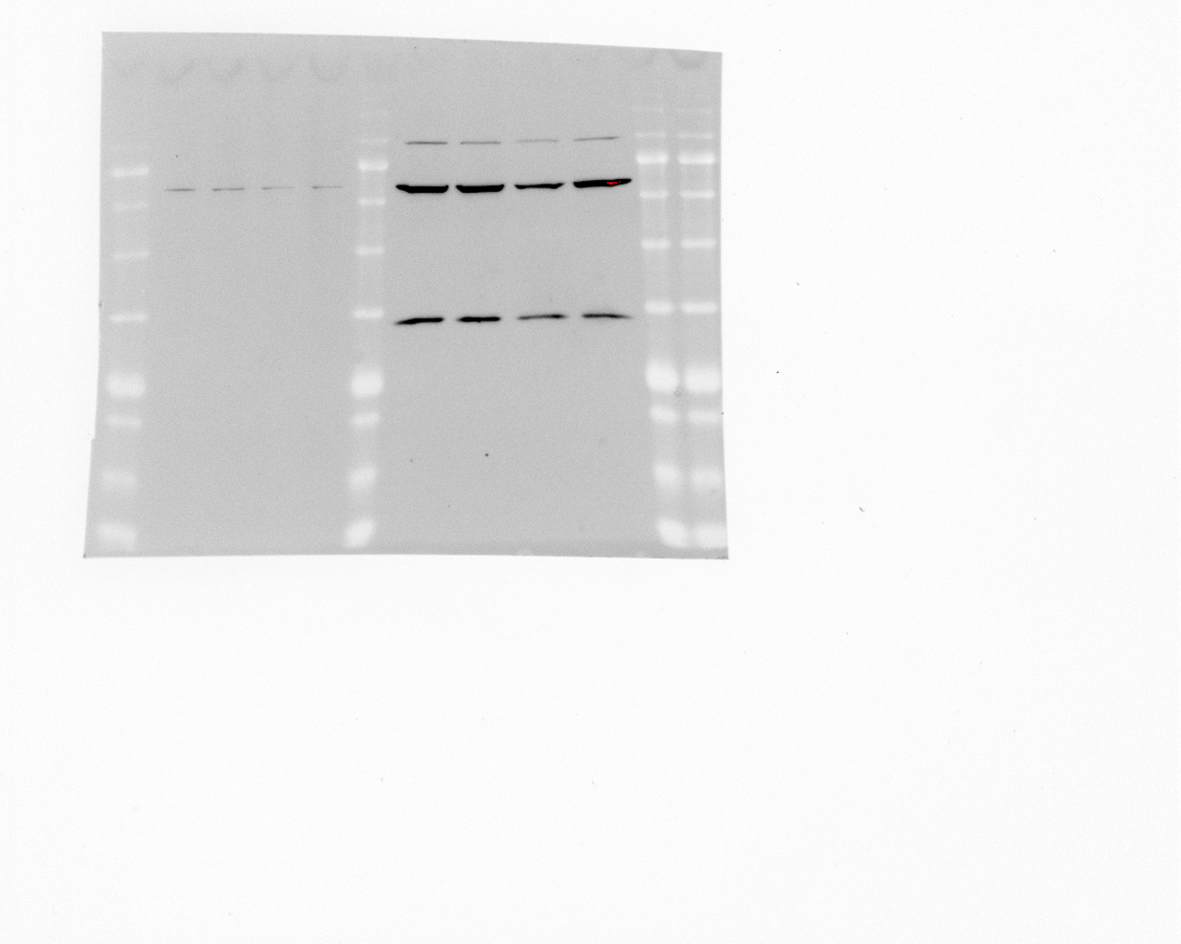

Supplement: Figure 5—figure supplement 1—source data 1. [file elife-102658-fig5-figsupp1-data1.zip › Figure 5-figure suplement 1-source data1/Figure 5-figure suplemment 1-A-3-source data1.tif]

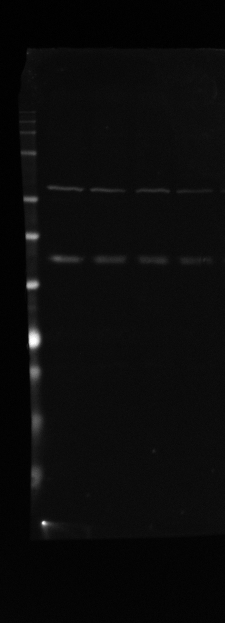

Supplement: Figure 5—figure supplement 1—source data 1. [file elife-102658-fig5-figsupp1-data1.zip › Figure 5-figure suplement 1-source data1/Figure 5-figure suplemment 1-C-5-source data1.tif]

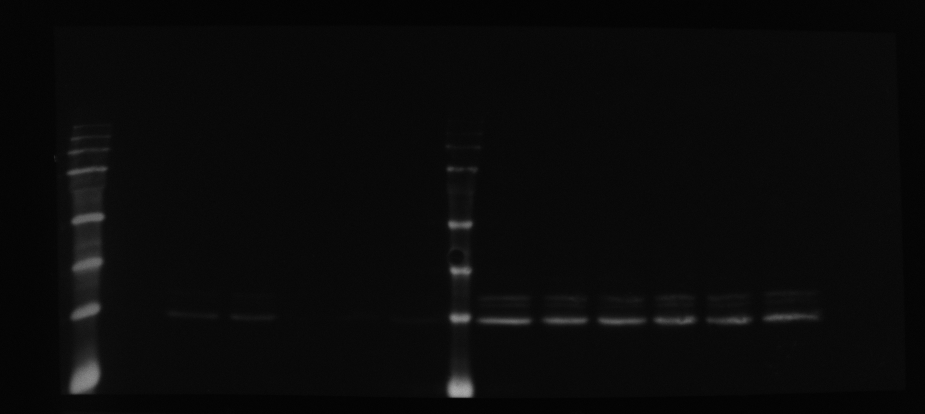

Supplement: Figure 5—figure supplement 1—source data 1. [file elife-102658-fig5-figsupp1-data1.zip › Figure 5-figure suplement 1-source data1/Figure 5-figure suplemment 1-F-4-source data1.tif]

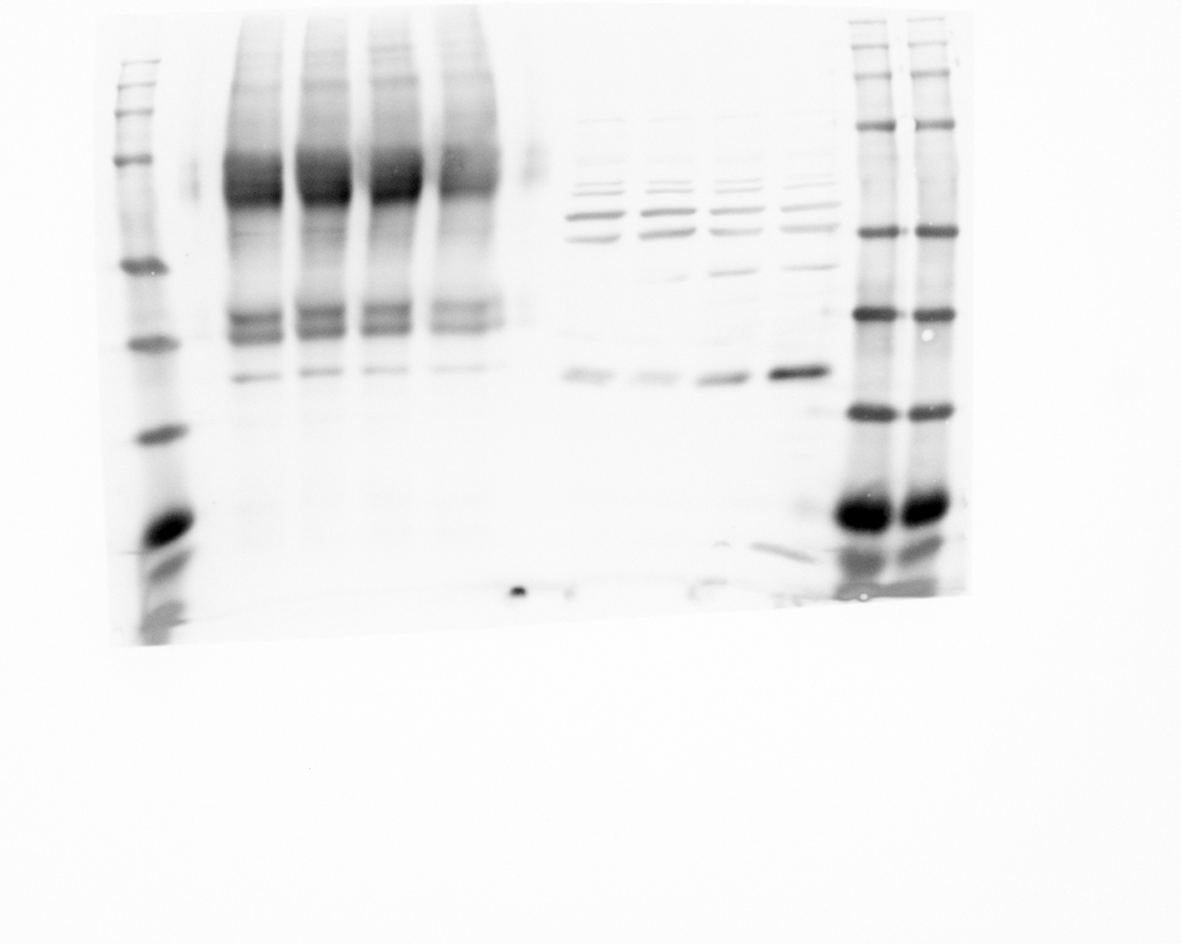

Supplement: Figure 5—figure supplement 1—source data 1. [file elife-102658-fig5-figsupp1-data1.zip › Figure 5-figure suplement 1-source data1/Figure 5-figure suplemment 1-B-2-source data1.tif]

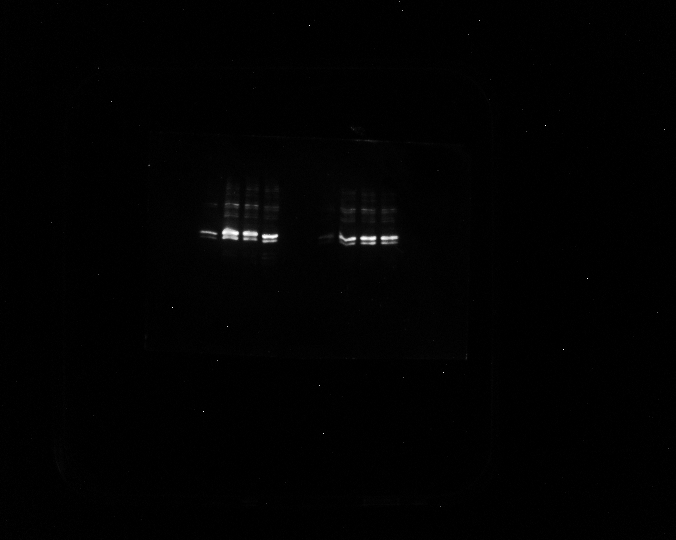

Supplement: Figure 5—figure supplement 1—source data 1. [file elife-102658-fig5-figsupp1-data1.zip › Figure 5-figure suplement 1-source data1/Figure 5-figure suplemment 1-D-3-source data1.tif]

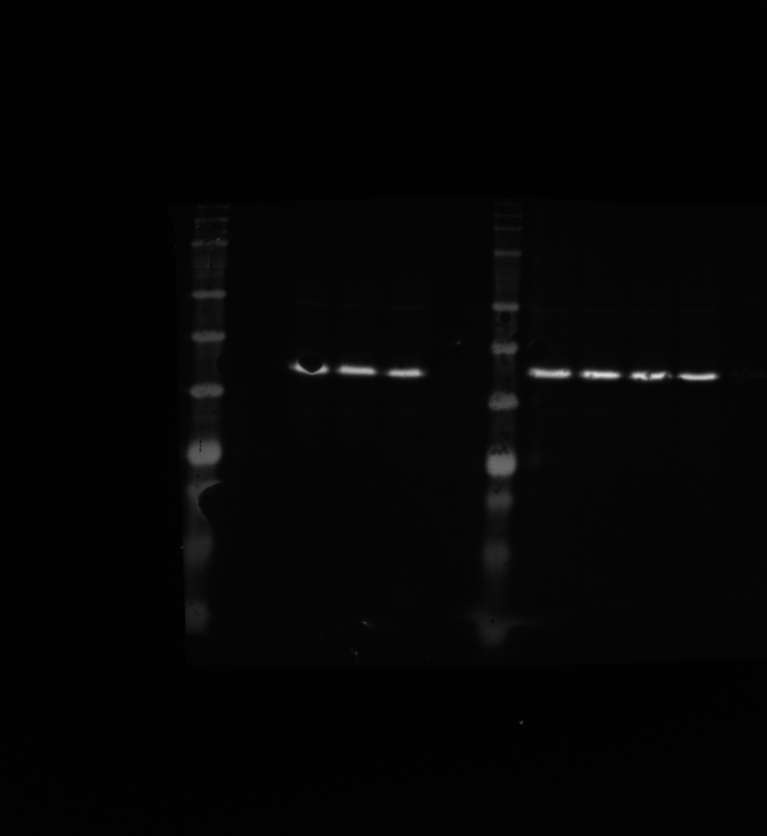

Supplement: Figure 5—figure supplement 1—source data 1. [file elife-102658-fig5-figsupp1-data1.zip › Figure 5-figure suplement 1-source data1/Figure 5-figure suplemment 1-C-1-source data1.tif]

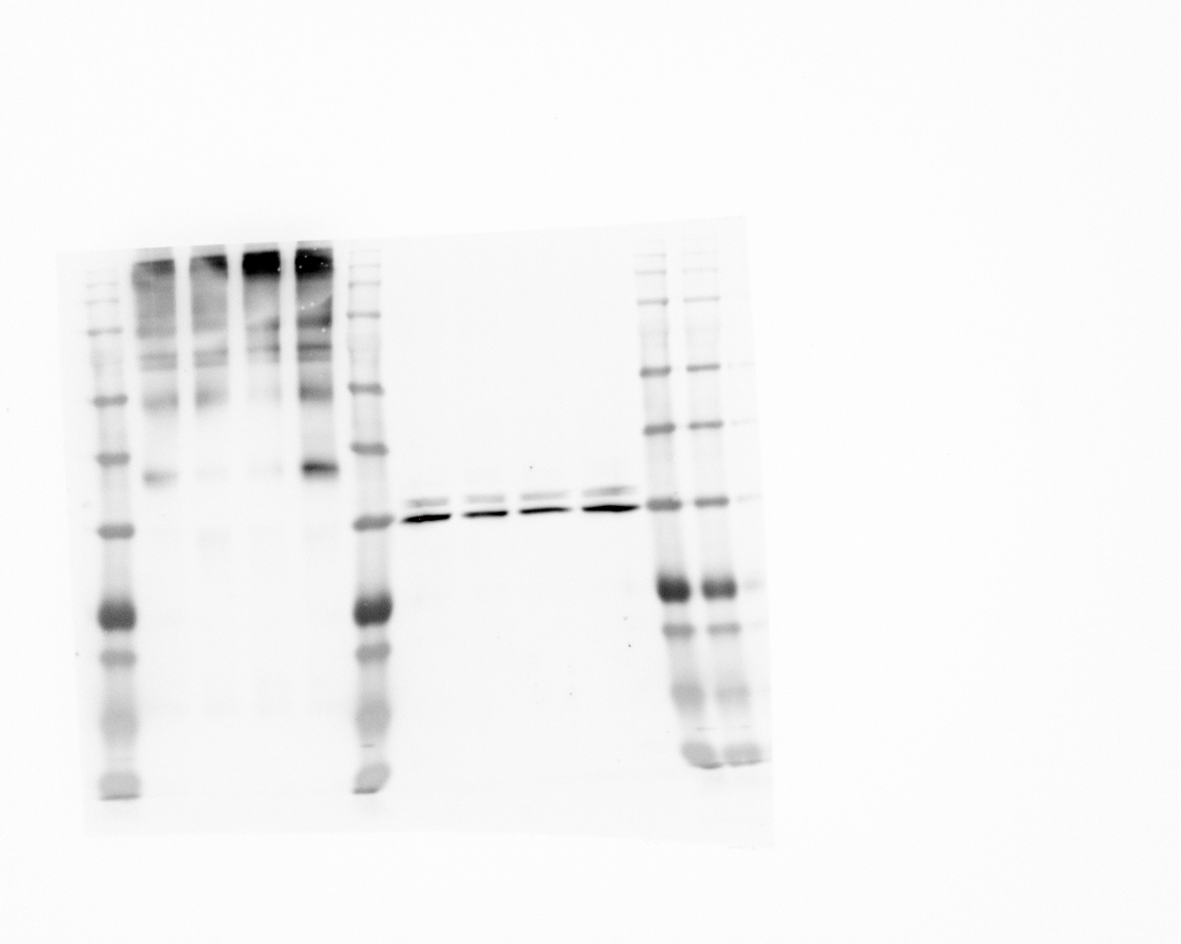

Supplement: Figure 5—figure supplement 1—source data 1. [file elife-102658-fig5-figsupp1-data1.zip › Figure 5-figure suplement 1-source data1/Figure 5-figure suplemment 1-A-2-source data1.tif]

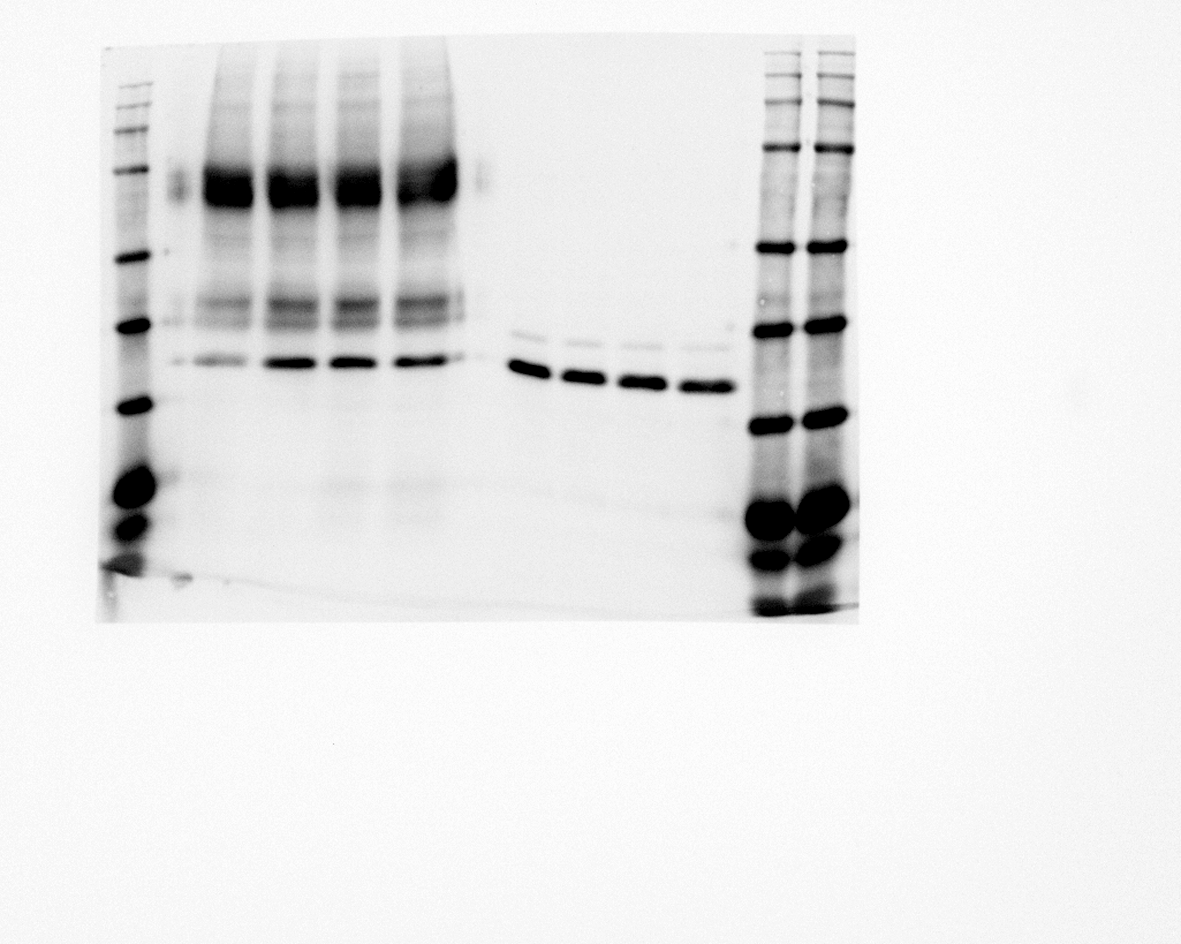

Supplement: Figure 5—figure supplement 1—source data 1. [file elife-102658-fig5-figsupp1-data1.zip › Figure 5-figure suplement 1-source data1/Figure 5-figure suplemment 1-B-1-source data1.tif]

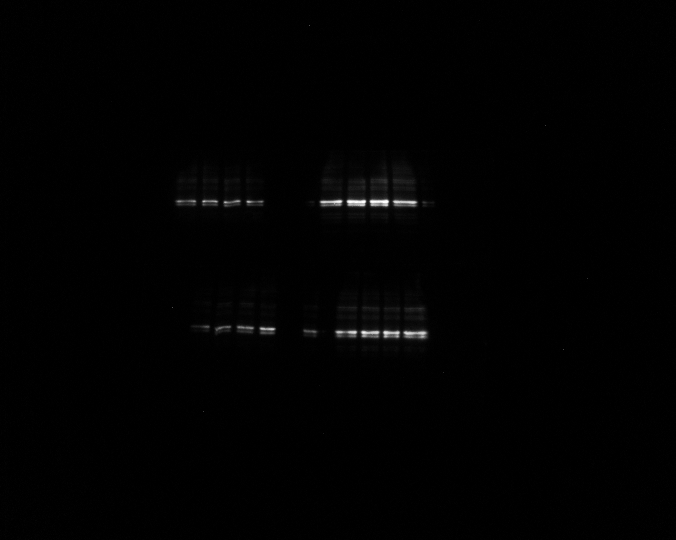

Supplement: Figure 5—figure supplement 1—source data 1. [file elife-102658-fig5-figsupp1-data1.zip › Figure 5-figure suplement 1-source data1/Figure 5-figure suplemment 1-D-4-source data1.tif]

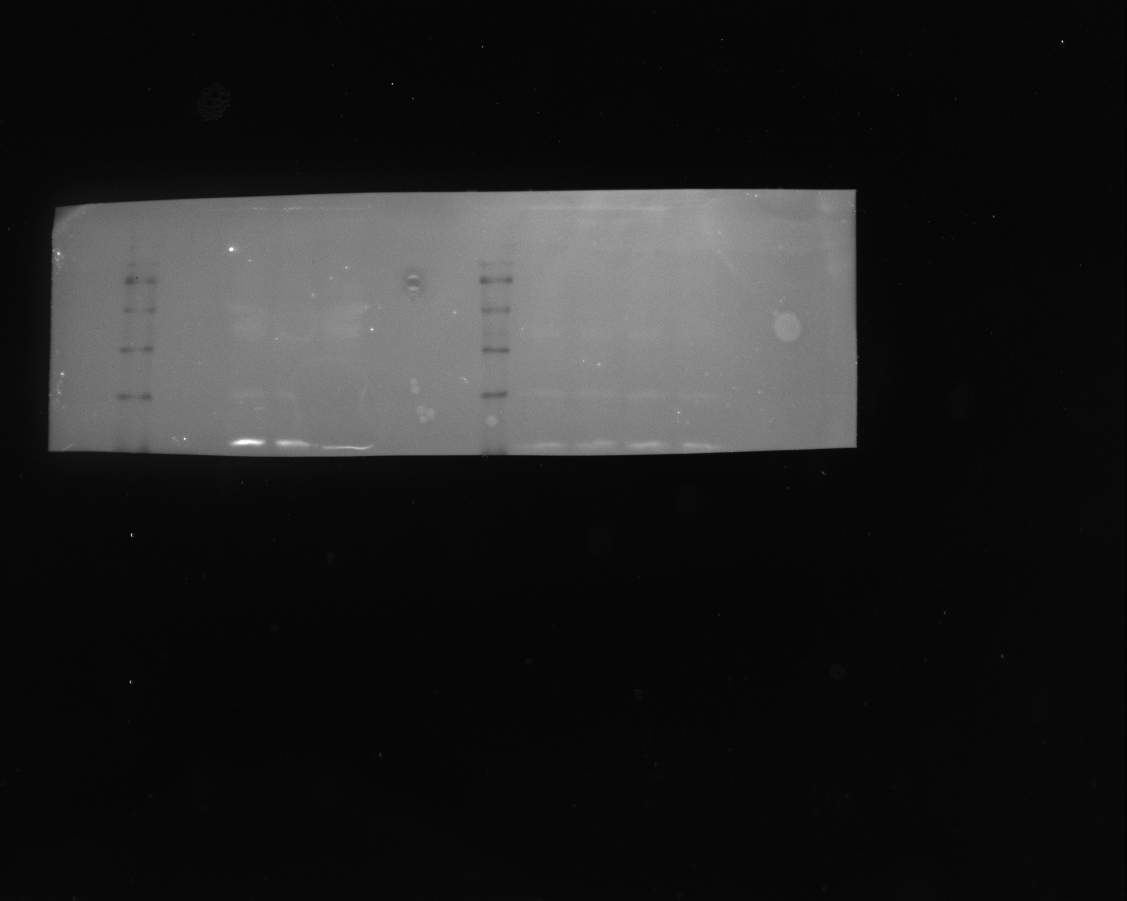

Supplement: Figure 5—figure supplement 1—source data 1. [file elife-102658-fig5-figsupp1-data1.zip › Figure 5-figure suplement 1-source data1/Figure 5-figure suplemment 1-E-3-source data1.tif]

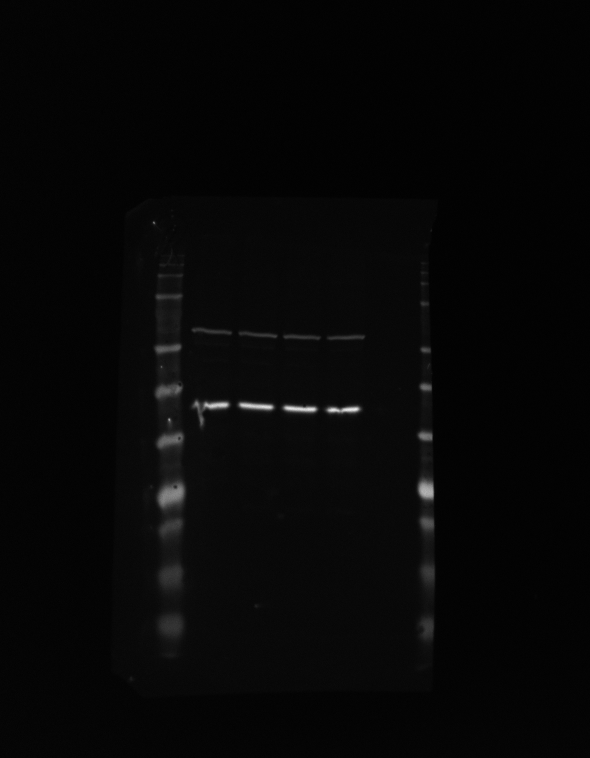

Supplement: Figure 5—figure supplement 1—source data 1. [file elife-102658-fig5-figsupp1-data1.zip › Figure 5-figure suplement 1-source data1/Figure 5-figure suplemment 1-C-2-source data1.tif]

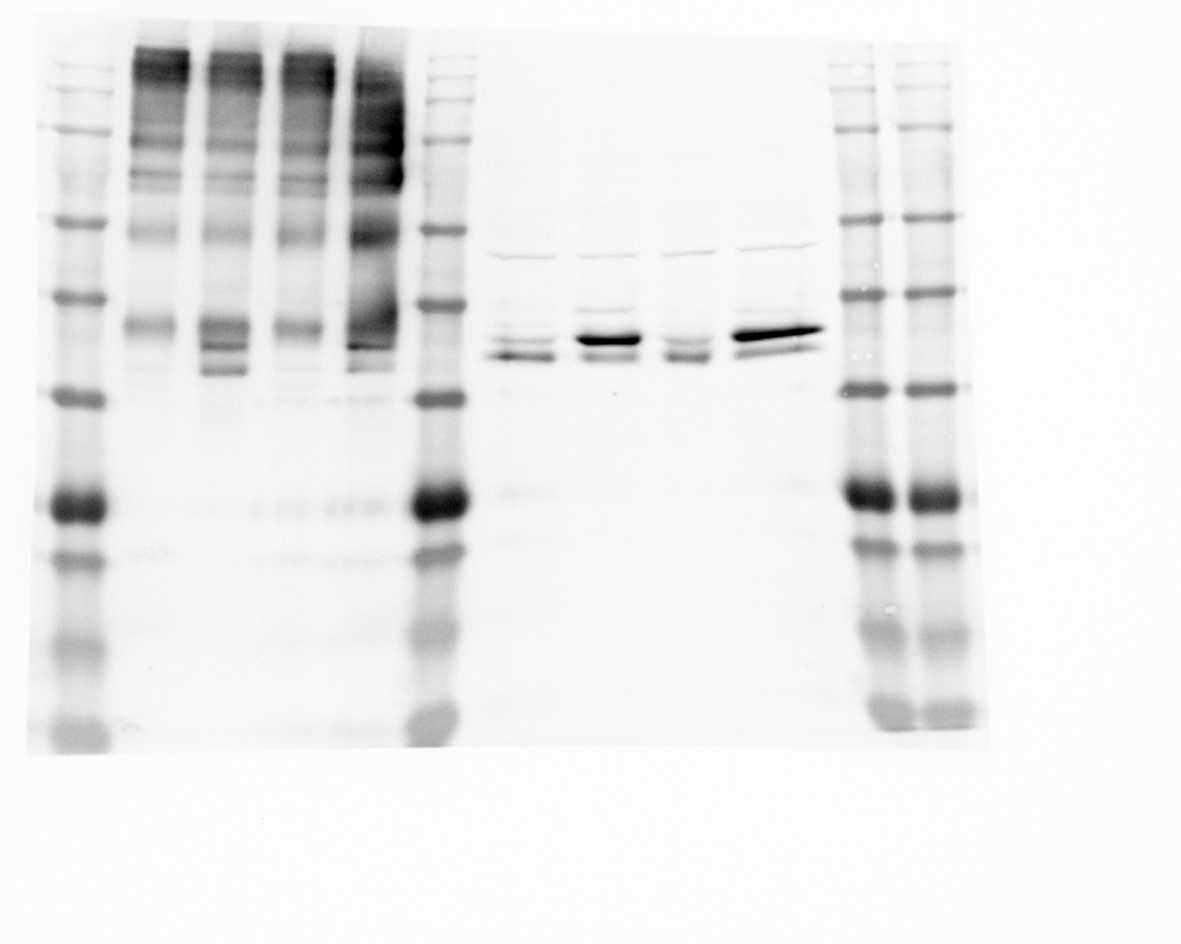

Supplement: Figure 5—figure supplement 1—source data 1. [file elife-102658-fig5-figsupp1-data1.zip › Figure 5-figure suplement 1-source data1/Figure 5-figure suplemment 1-A-1-source data1.tif]

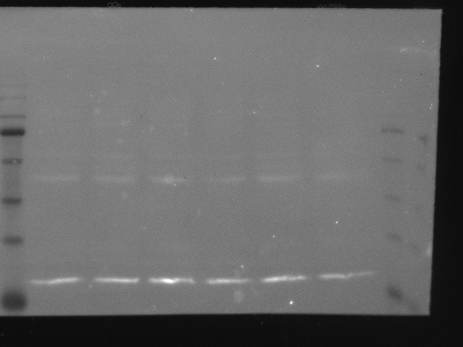

Supplement: Figure 5—figure supplement 1—source data 1. [file elife-102658-fig5-figsupp1-data1.zip › Figure 5-figure suplement 1-source data1/Figure 5-figure suplemment 1-F-3-source data1.tif]
