# Supplementary figures and images for: Cytosolic and endoplasmic reticulum chaperones inhibit wt-p53 to increase cancer cells' survival by refluxing ER-proteins to the cytosol (part 3 of 3)
Source: eLife. 2025 Apr 9;14:e102658. doi: 10.7554/eLife.102658 (PMC11981610; doi:10.7554/eLife.102658)

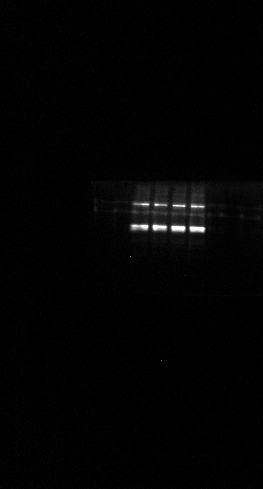

Supplement: Figure 5—figure supplement 1—source data 1. [file elife-102658-fig5-figsupp1-data1.zip › Figure 5-figure suplement 1-source data1/Figure 5-figure suplemment 1-D-5-source data1.tif]

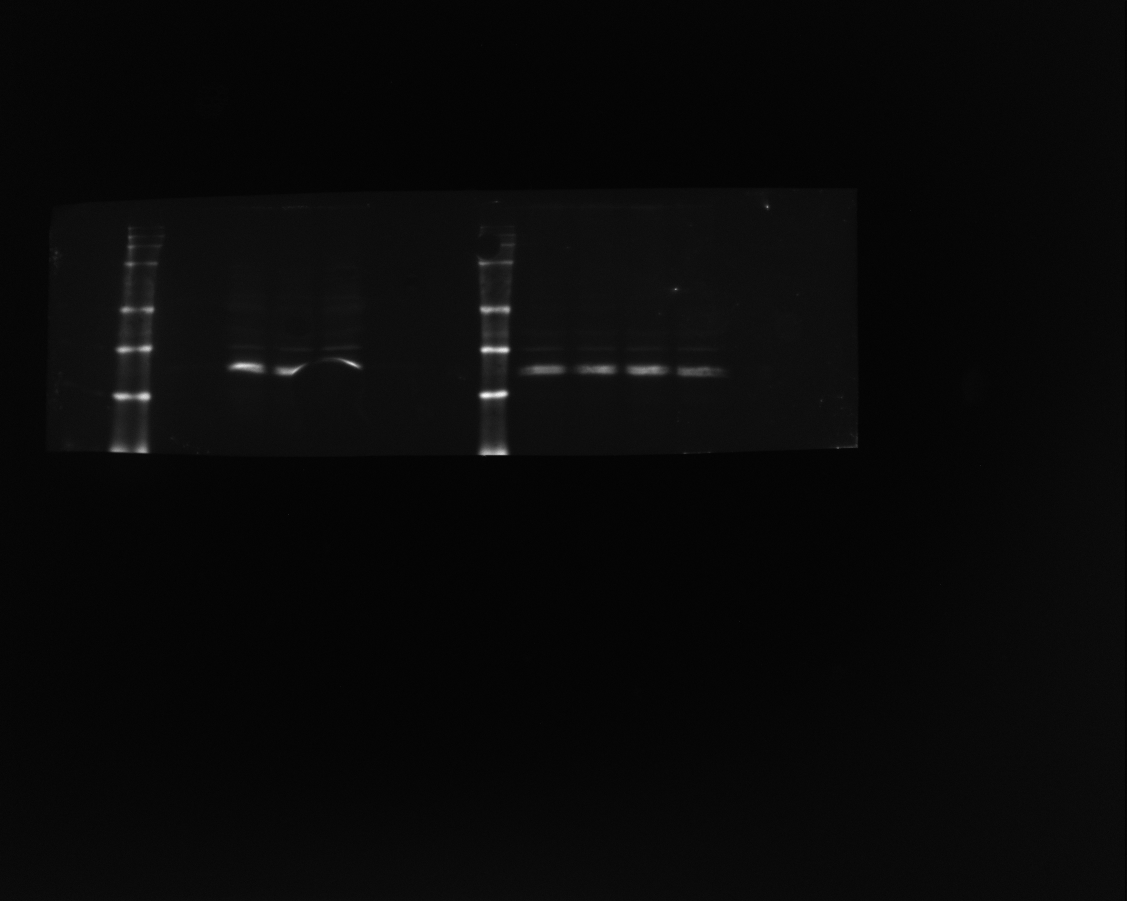

Supplement: Figure 5—figure supplement 1—source data 1. [file elife-102658-fig5-figsupp1-data1.zip › Figure 5-figure suplement 1-source data1/Figure 5-figure suplemment 1-E-2-source data1.tif]

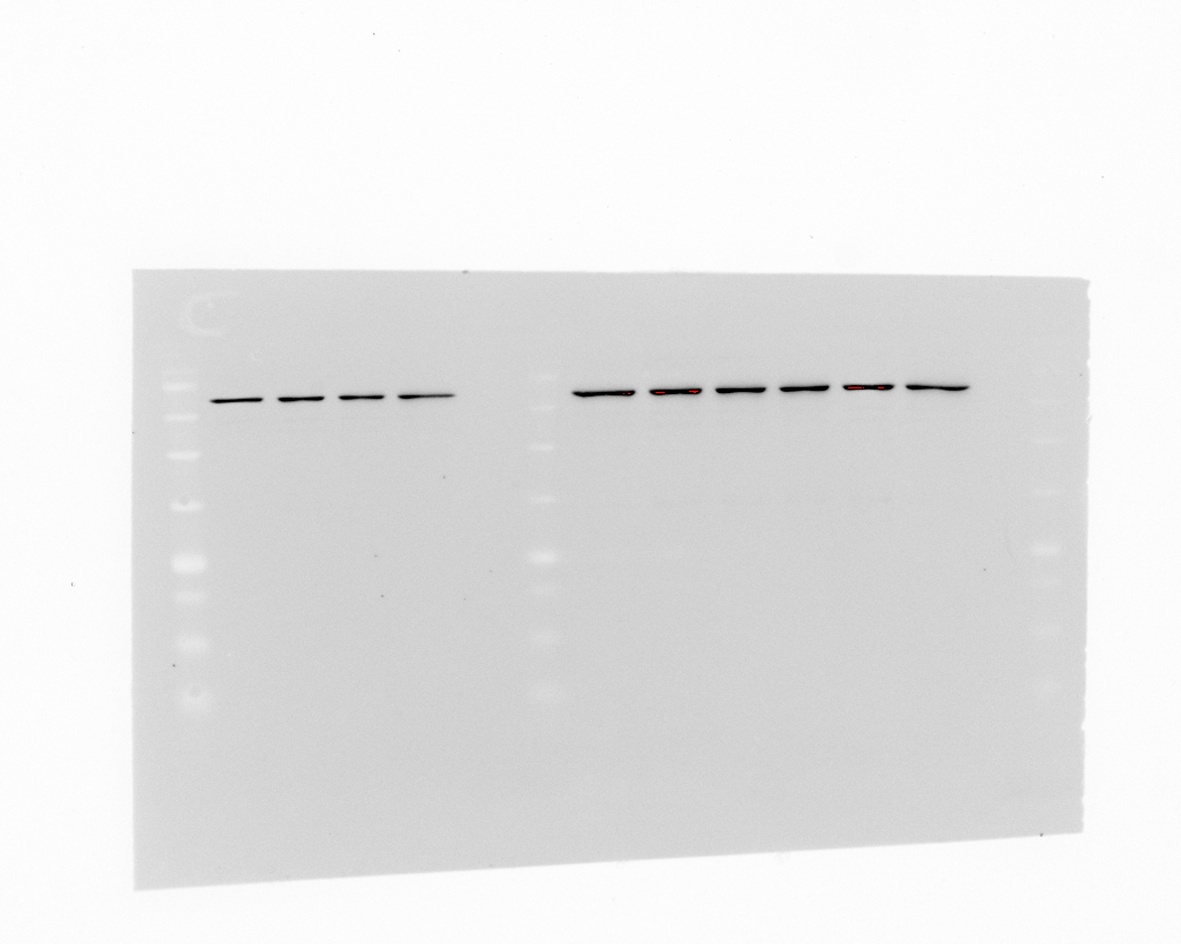

Supplement: Figure 5—figure supplement 1—source data 1. [file elife-102658-fig5-figsupp1-data1.zip › Figure 5-figure suplement 1-source data1/Figure 5-figure suplemment 1-C-6-source data1.jpg]

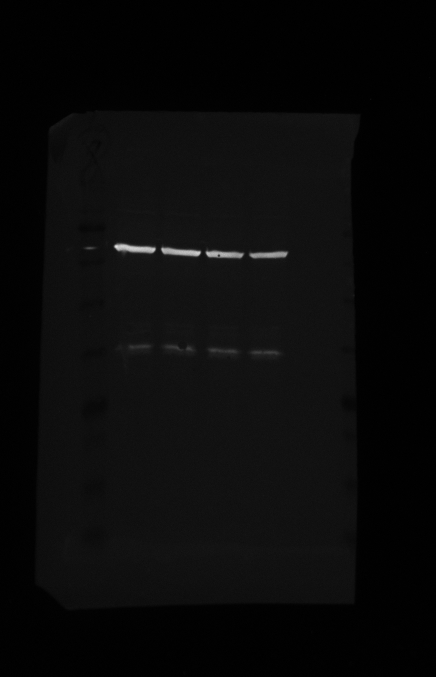

Supplement: Figure 5—figure supplement 1—source data 1. [file elife-102658-fig5-figsupp1-data1.zip › Figure 5-figure suplement 1-source data1/Figure 5-figure suplemment 1-C-3-source data1.tif]

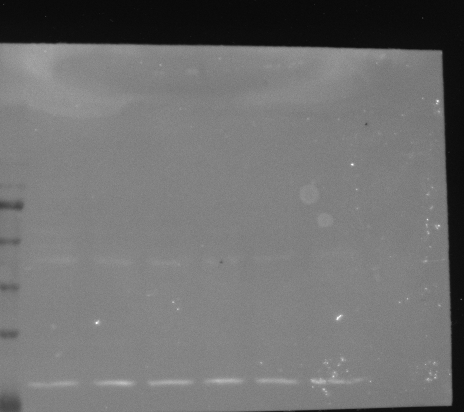

Supplement: Figure 5—figure supplement 1—source data 1. [file elife-102658-fig5-figsupp1-data1.zip › Figure 5-figure suplement 1-source data1/Figure 5-figure suplemment 1-F-2-source data1.tif]

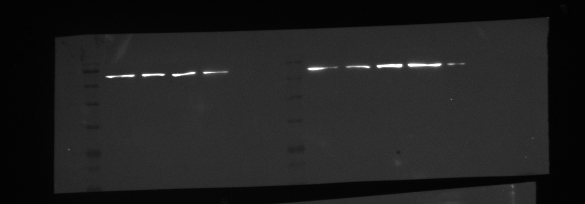

Supplement: Figure 5—figure supplement 1—source data 1. [file elife-102658-fig5-figsupp1-data1.zip › Figure 5-figure suplement 1-source data1/Figure 5-figure suplemment 1-D-1-source data1.tif]

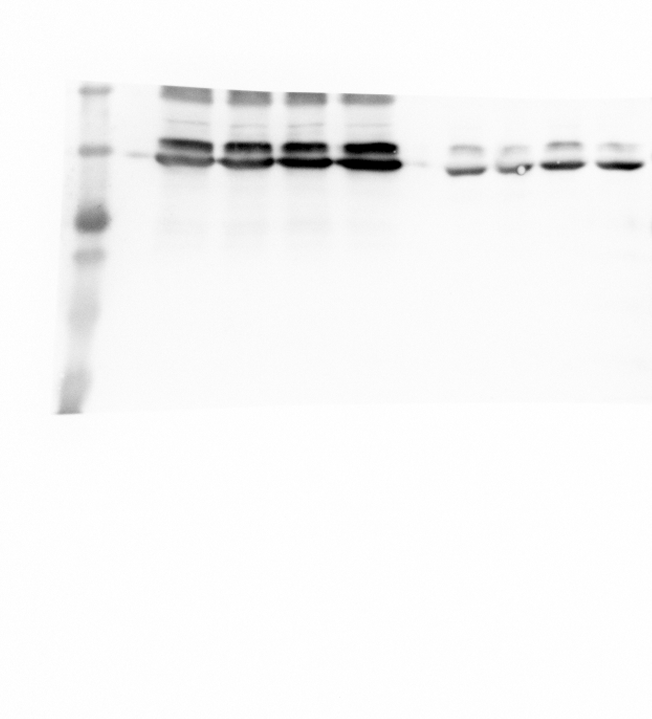

Supplement: Figure 5—figure supplement 1—source data 1. [file elife-102658-fig5-figsupp1-data1.zip › Figure 5-figure suplement 1-source data1/Figure 5-figure suplemment 1-B-4-source data1.tif]

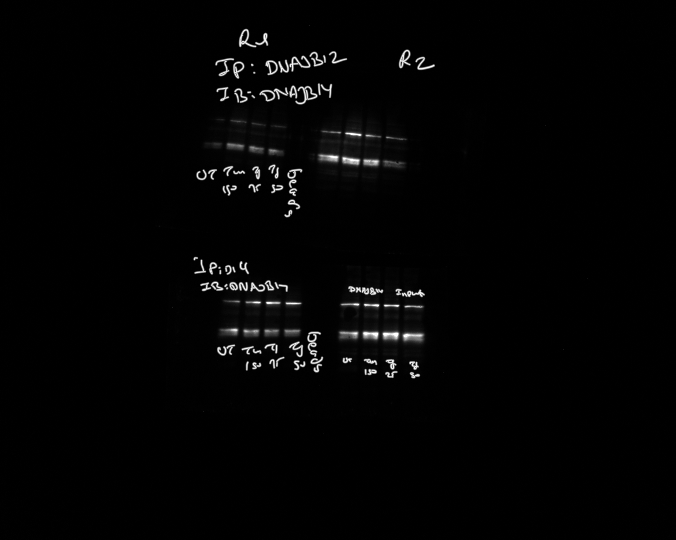

Supplement: Figure 5—figure supplement 1—source data 2. [file elife-102658-fig5-figsupp1-data2.zip › Figure 5-figure suplement 1-source data1/Figure 5-figure suplemment 1-D-6-source data1tif.tif]

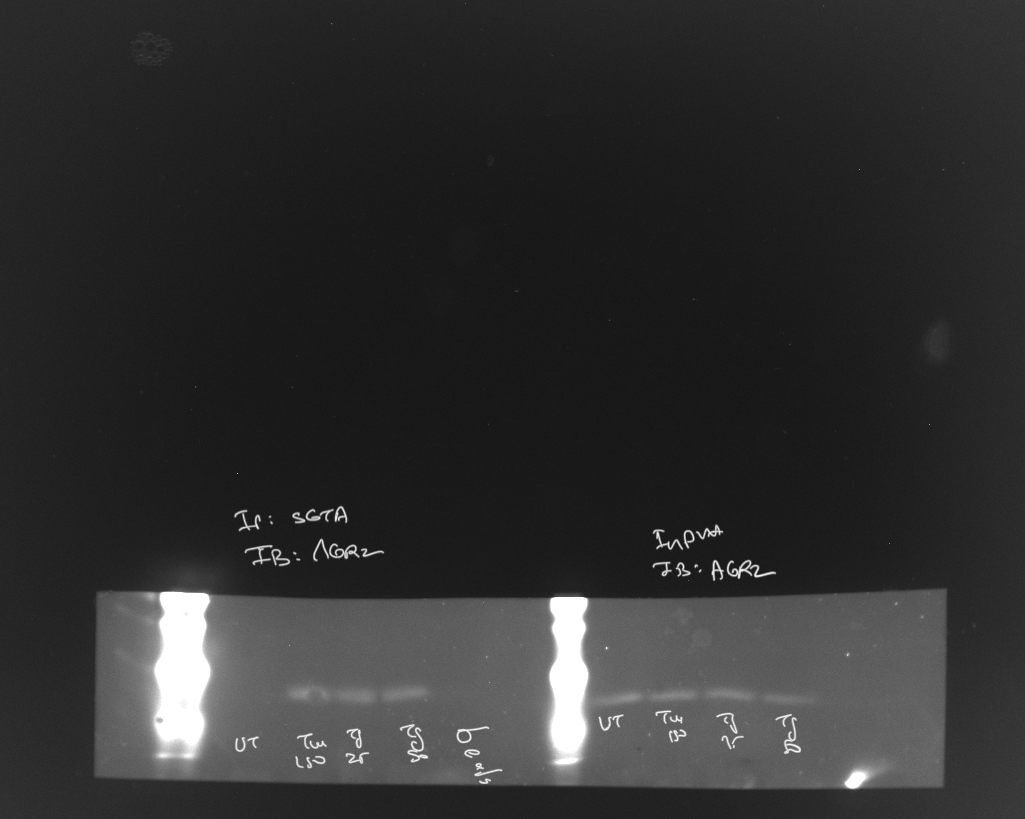

Supplement: Figure 5—figure supplement 1—source data 2. [file elife-102658-fig5-figsupp1-data2.zip › Figure 5-figure suplement 1-source data1/Figure 5-figure suplemment 1-E-1-source data1.tif]

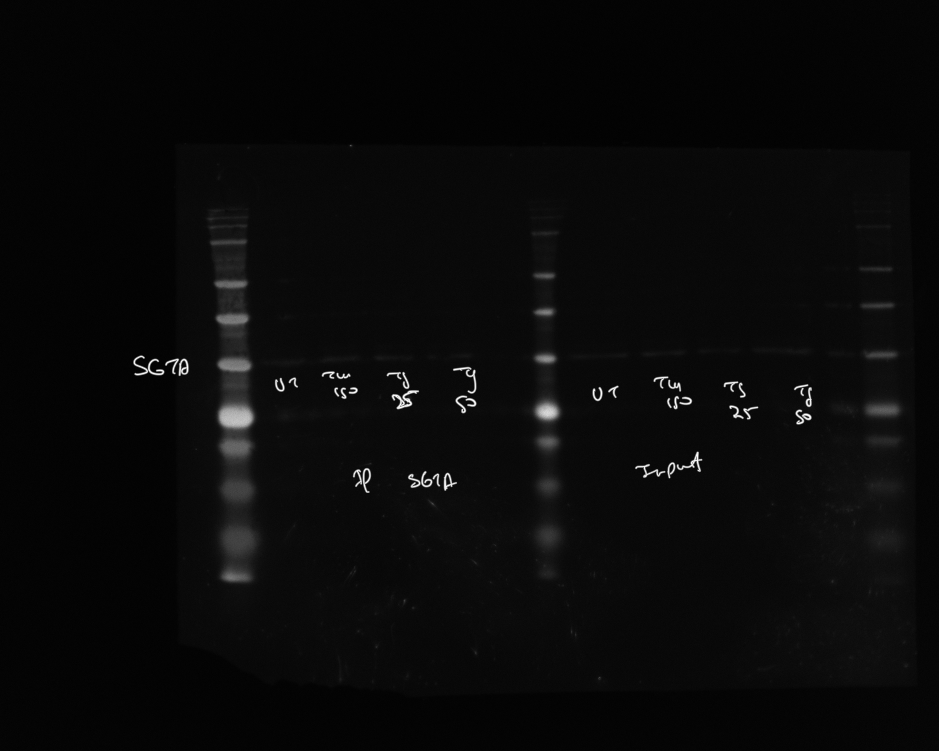

Supplement: Figure 5—figure supplement 1—source data 2. [file elife-102658-fig5-figsupp1-data2.zip › Figure 5-figure suplement 1-source data1/Figure 5-figure suplemment 1-C-4-source data1.tif]

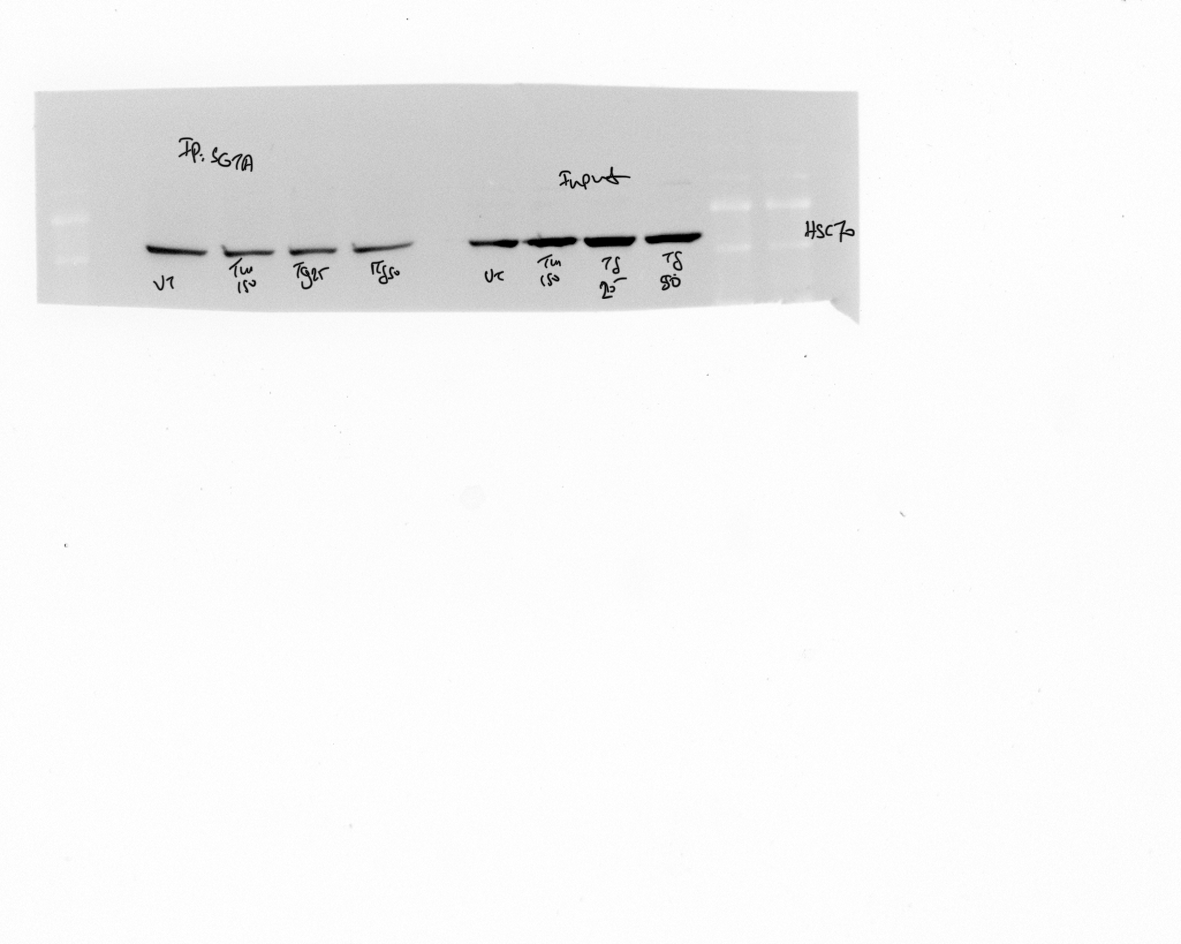

Supplement: Figure 5—figure supplement 1—source data 2. [file elife-102658-fig5-figsupp1-data2.zip › Figure 5-figure suplement 1-source data1/Figure 5-figure suplemment 1-B-3-source data1.tif]

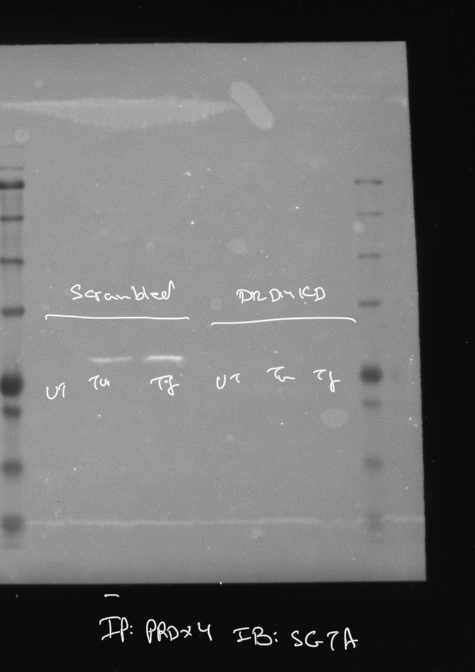

Supplement: Figure 5—figure supplement 1—source data 2. [file elife-102658-fig5-figsupp1-data2.zip › Figure 5-figure suplement 1-source data1/Figure 5-figure suplemment 1-F-1-source data1.tif]

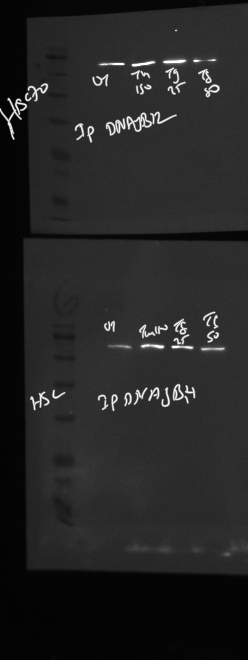

Supplement: Figure 5—figure supplement 1—source data 2. [file elife-102658-fig5-figsupp1-data2.zip › Figure 5-figure suplement 1-source data1/Figure 5-figure suplemment 1-D-2-source data1.tif]

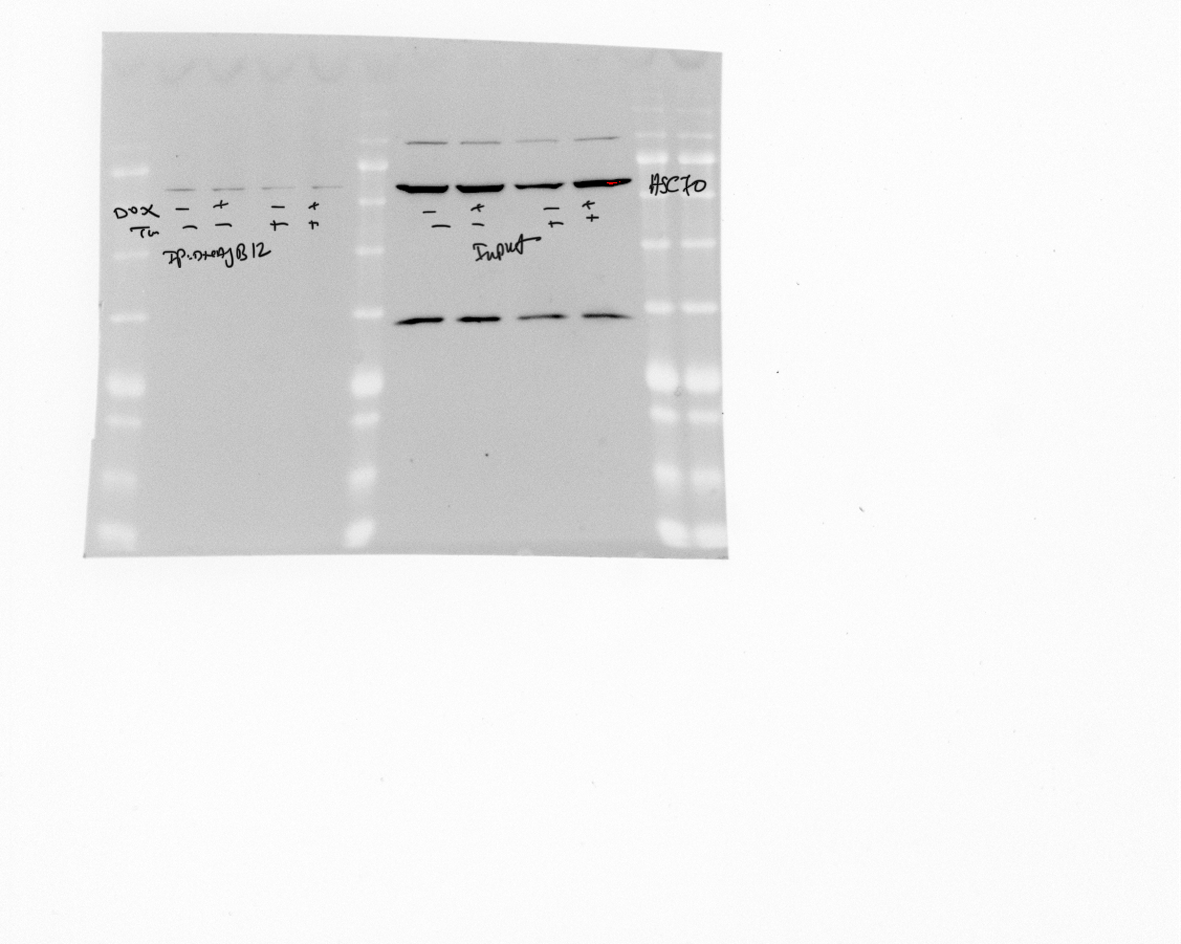

Supplement: Figure 5—figure supplement 1—source data 2. [file elife-102658-fig5-figsupp1-data2.zip › Figure 5-figure suplement 1-source data1/Figure 5-figure suplemment 1-A-3-source data1.tif]

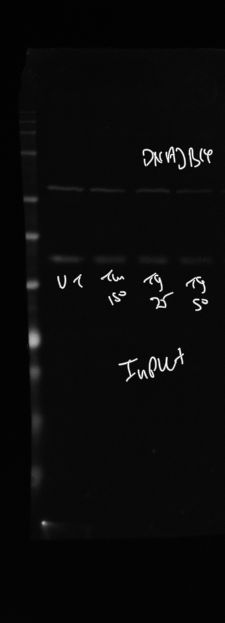

Supplement: Figure 5—figure supplement 1—source data 2. [file elife-102658-fig5-figsupp1-data2.zip › Figure 5-figure suplement 1-source data1/Figure 5-figure suplemment 1-C-5-source data1.tif]

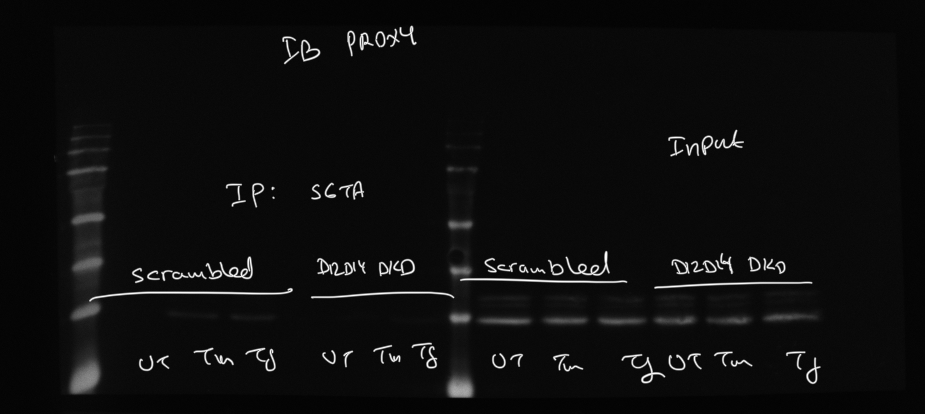

Supplement: Figure 5—figure supplement 1—source data 2. [file elife-102658-fig5-figsupp1-data2.zip › Figure 5-figure suplement 1-source data1/Figure 5-figure suplemment 1-F-4-source data1.tif]

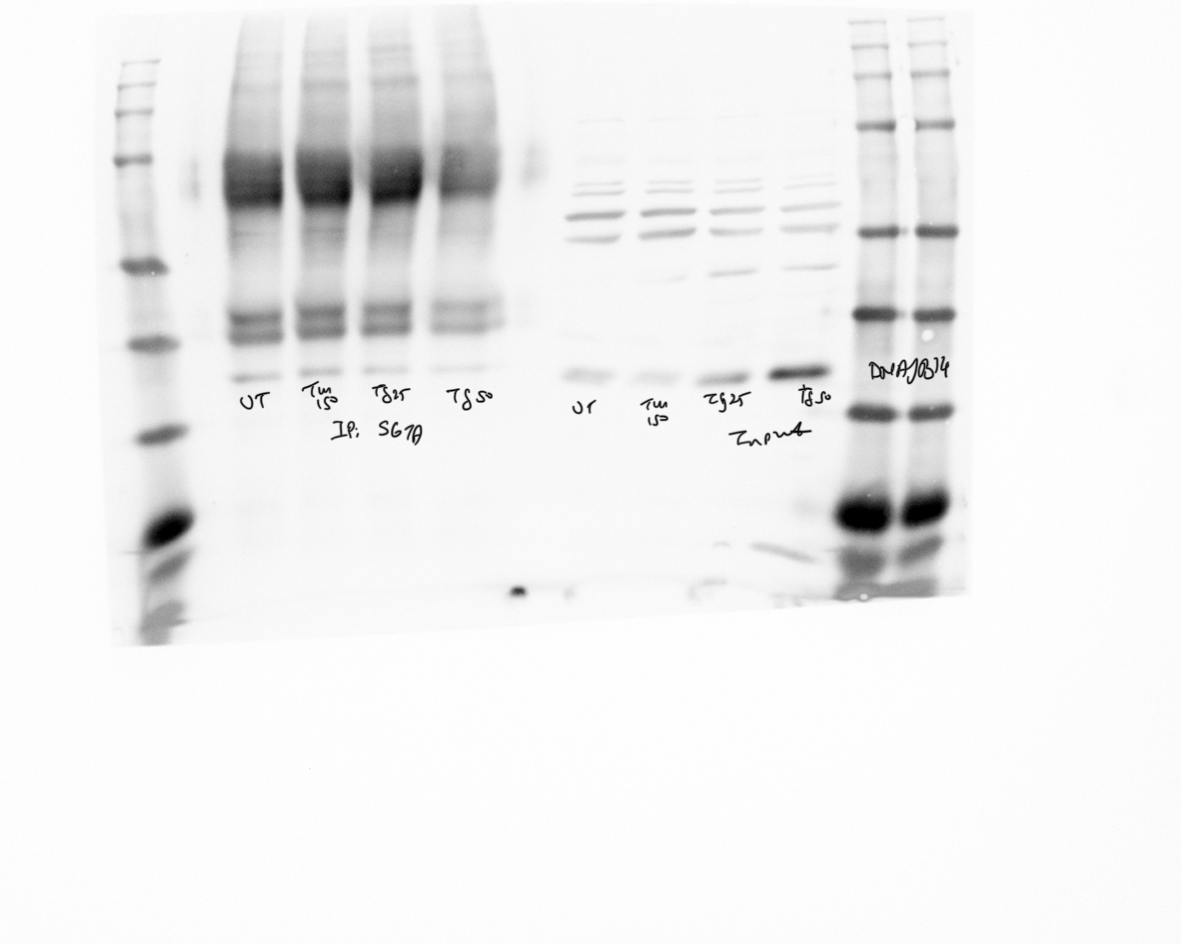

Supplement: Figure 5—figure supplement 1—source data 2. [file elife-102658-fig5-figsupp1-data2.zip › Figure 5-figure suplement 1-source data1/Figure 5-figure suplemment 1-B-2-source data1.tif]

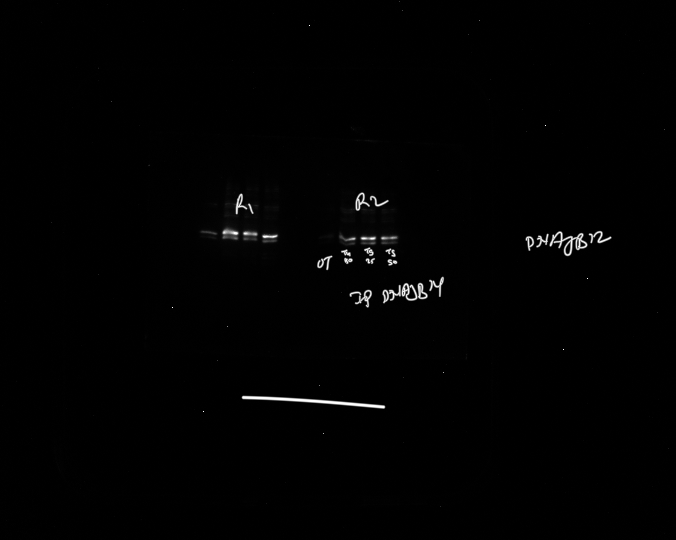

Supplement: Figure 5—figure supplement 1—source data 2. [file elife-102658-fig5-figsupp1-data2.zip › Figure 5-figure suplement 1-source data1/Figure 5-figure suplemment 1-D-3-source data1.tif]

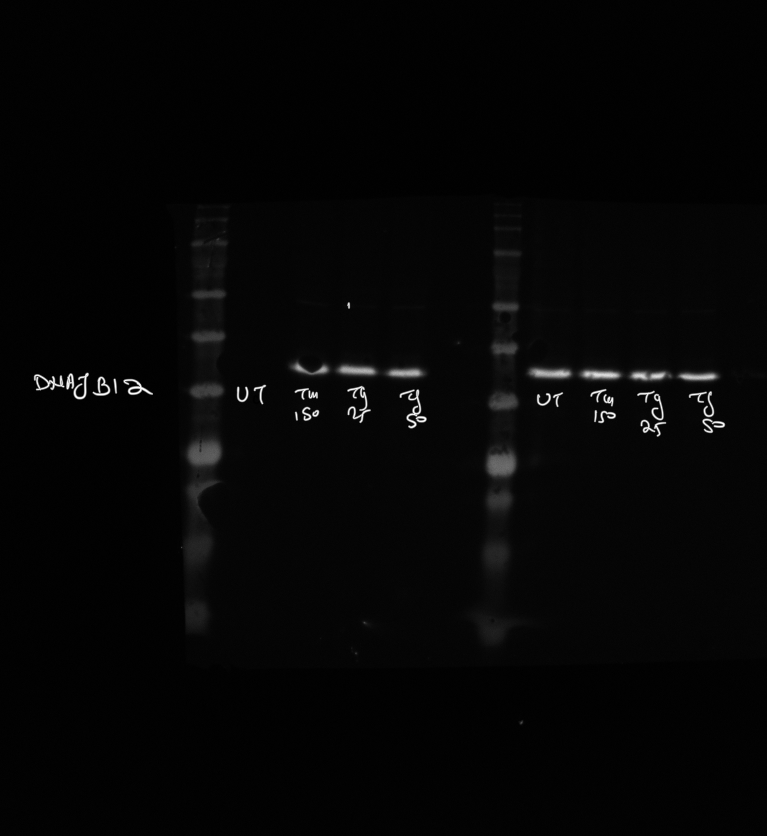

Supplement: Figure 5—figure supplement 1—source data 2. [file elife-102658-fig5-figsupp1-data2.zip › Figure 5-figure suplement 1-source data1/Figure 5-figure suplemment 1-C-1-source data1.tif]

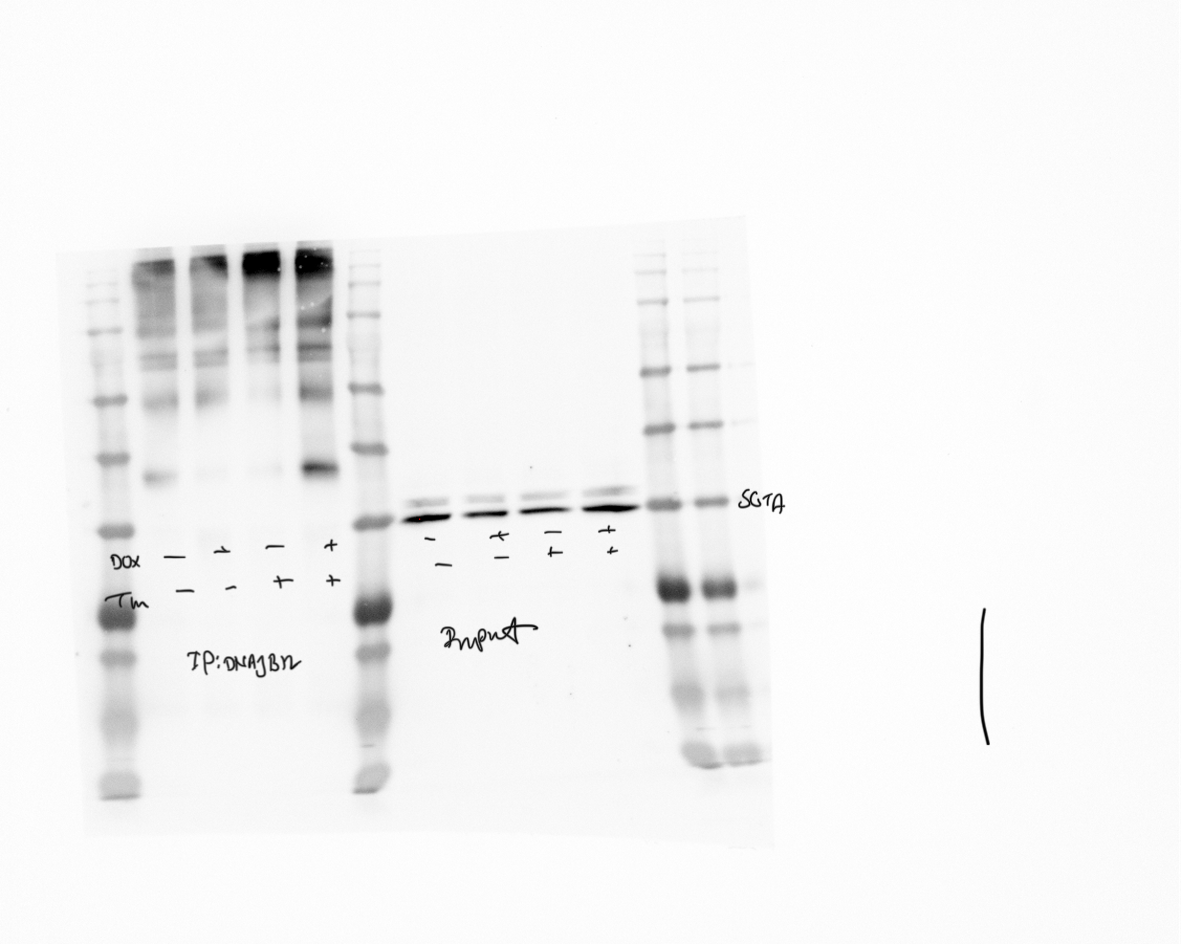

Supplement: Figure 5—figure supplement 1—source data 2. [file elife-102658-fig5-figsupp1-data2.zip › Figure 5-figure suplement 1-source data1/Figure 5-figure suplemment 1-A-2-source data1.tif]

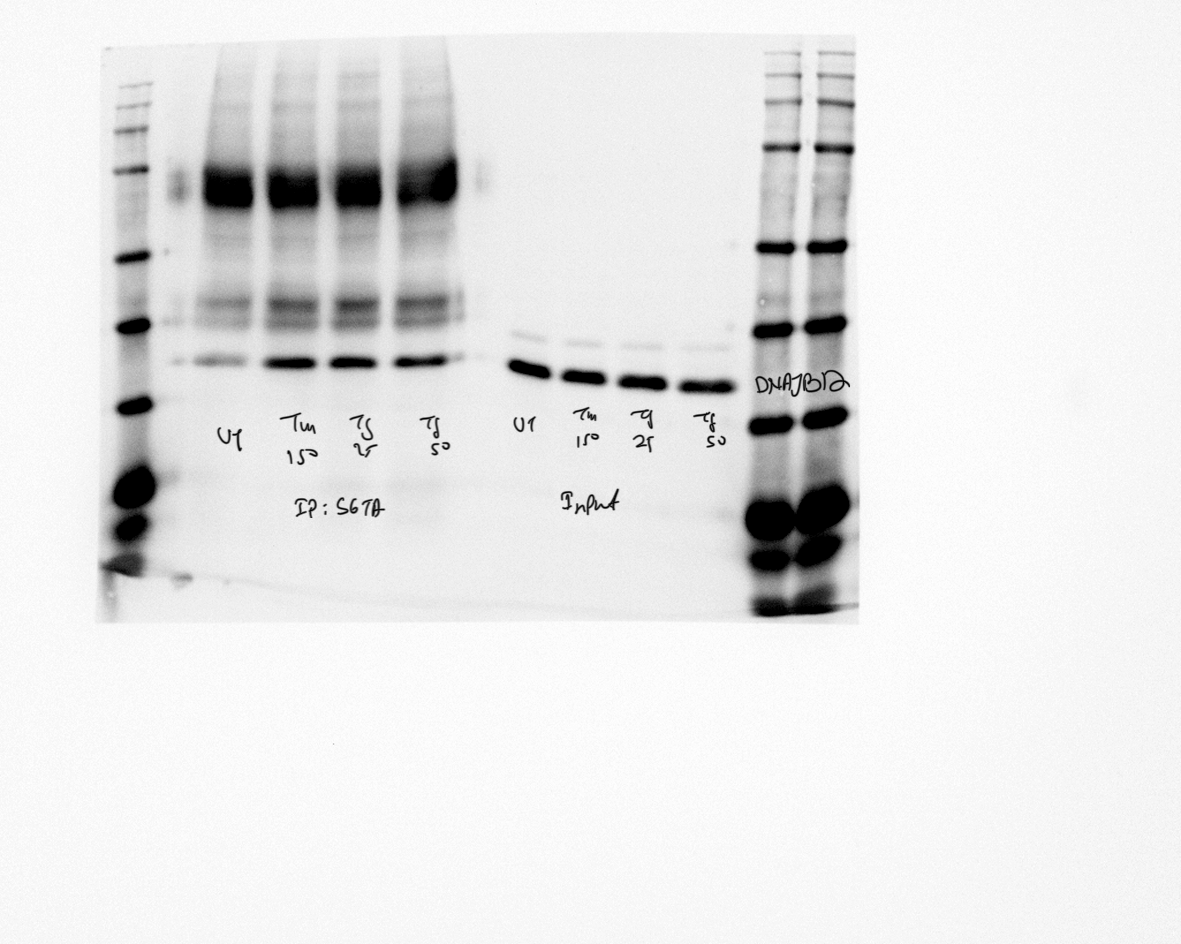

Supplement: Figure 5—figure supplement 1—source data 2. [file elife-102658-fig5-figsupp1-data2.zip › Figure 5-figure suplement 1-source data1/Figure 5-figure suplemment 1-B-1-source data1.tif]

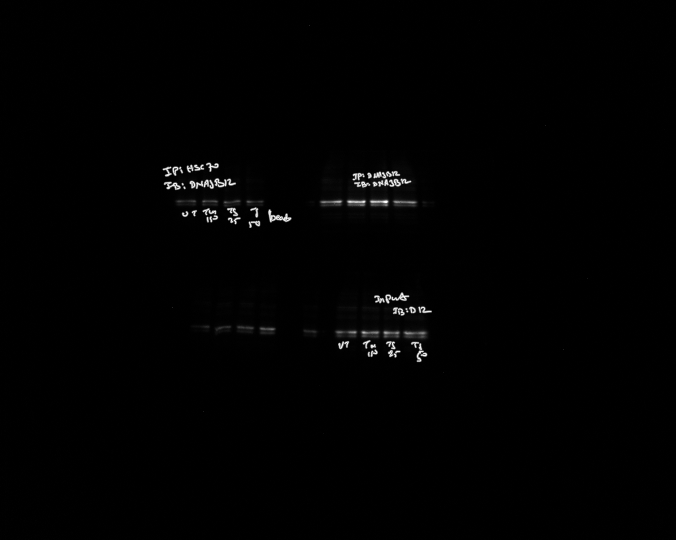

Supplement: Figure 5—figure supplement 1—source data 2. [file elife-102658-fig5-figsupp1-data2.zip › Figure 5-figure suplement 1-source data1/Figure 5-figure suplemment 1-D-4-source data1.tif]

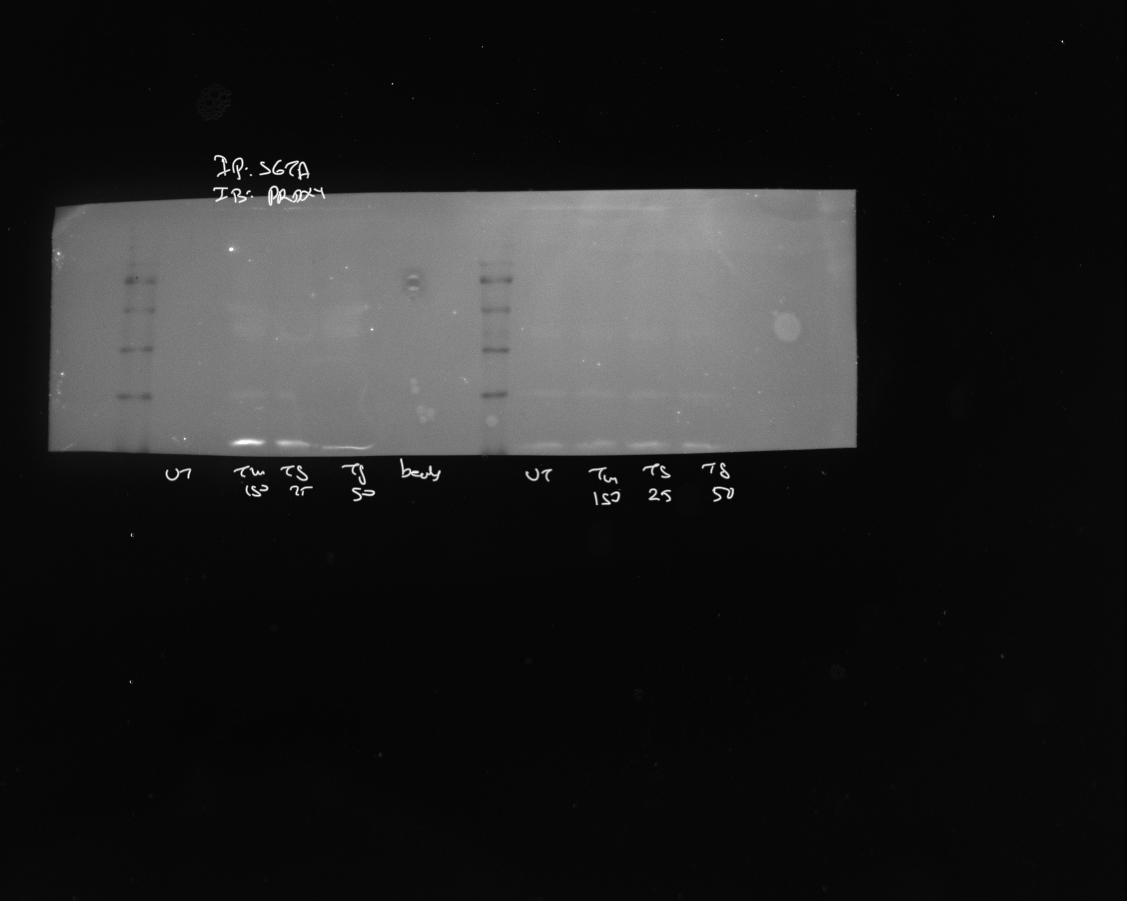

Supplement: Figure 5—figure supplement 1—source data 2. [file elife-102658-fig5-figsupp1-data2.zip › Figure 5-figure suplement 1-source data1/Figure 5-figure suplemment 1-E-3-source data1.tif]

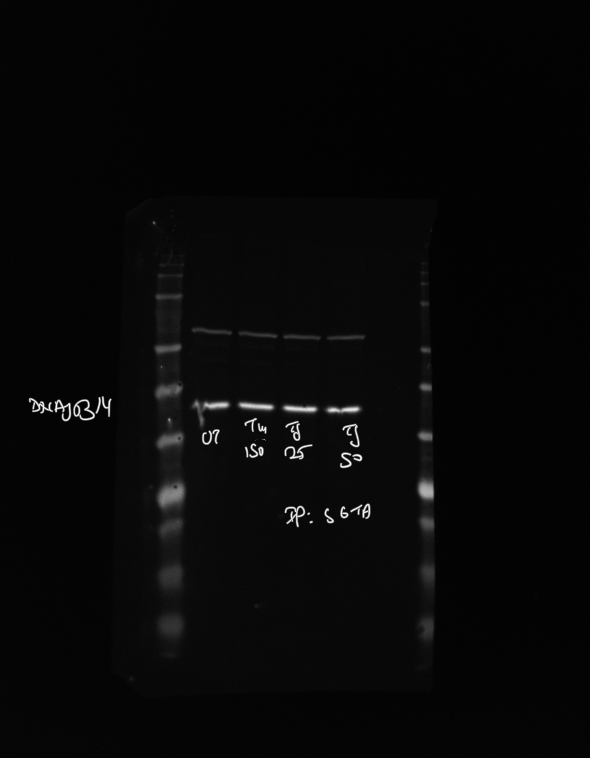

Supplement: Figure 5—figure supplement 1—source data 2. [file elife-102658-fig5-figsupp1-data2.zip › Figure 5-figure suplement 1-source data1/Figure 5-figure suplemment 1-C-2-source data1.tif]

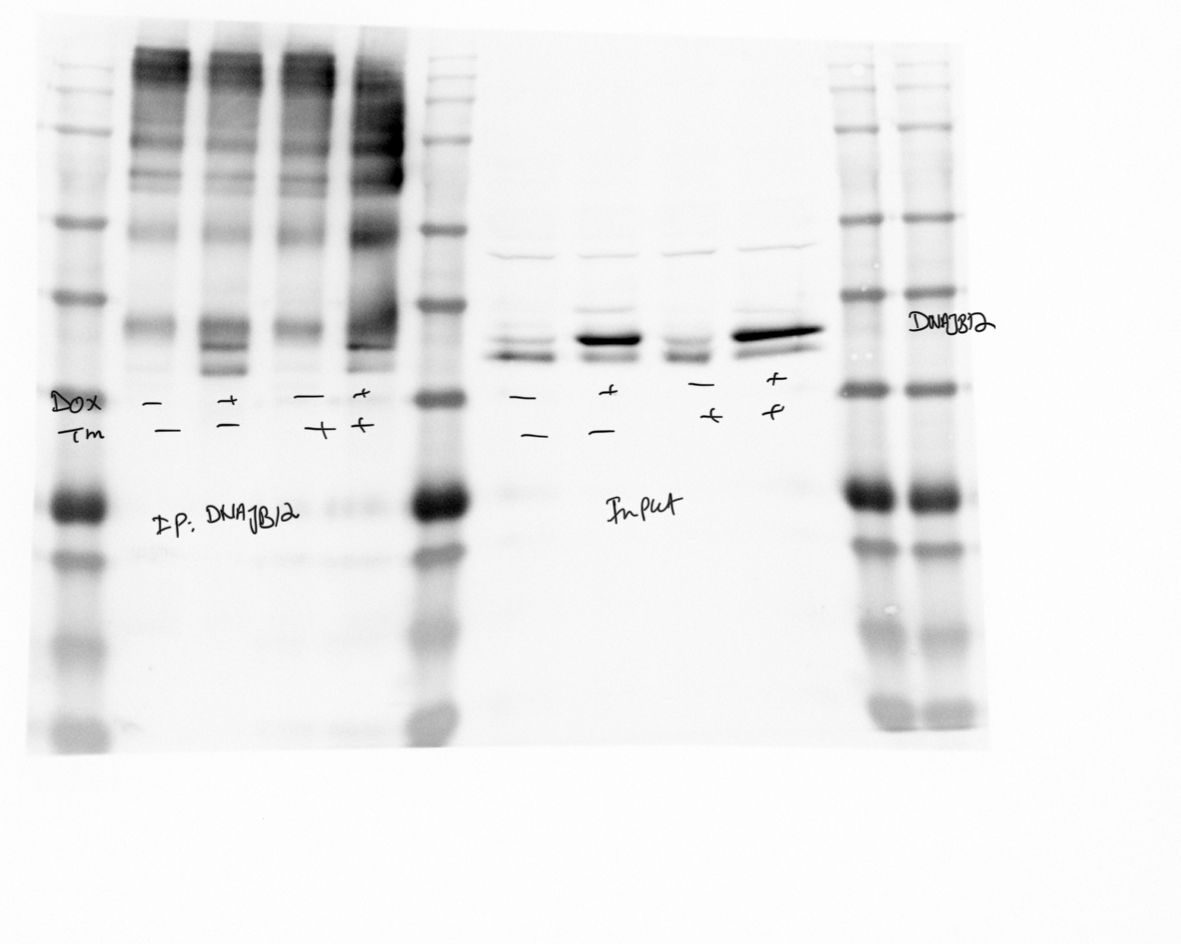

Supplement: Figure 5—figure supplement 1—source data 2. [file elife-102658-fig5-figsupp1-data2.zip › Figure 5-figure suplement 1-source data1/Figure 5-figure suplemment 1-A-1-source data1.tif]

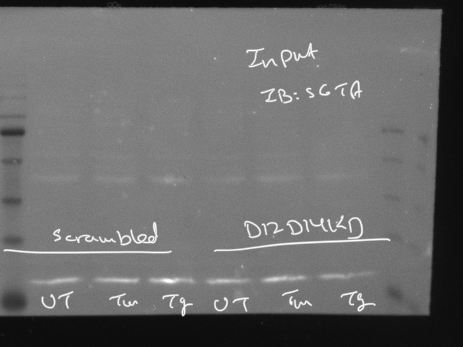

Supplement: Figure 5—figure supplement 1—source data 2. [file elife-102658-fig5-figsupp1-data2.zip › Figure 5-figure suplement 1-source data1/Figure 5-figure suplemment 1-F-3-source data1.tif]

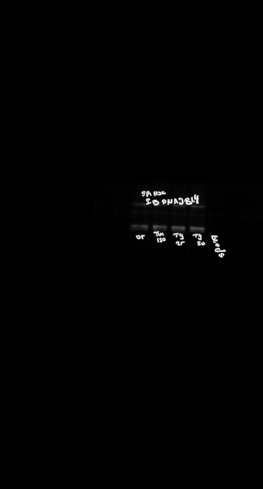

Supplement: Figure 5—figure supplement 1—source data 2. [file elife-102658-fig5-figsupp1-data2.zip › Figure 5-figure suplement 1-source data1/Figure 5-figure suplemment 1-D-5-source data1.tif]

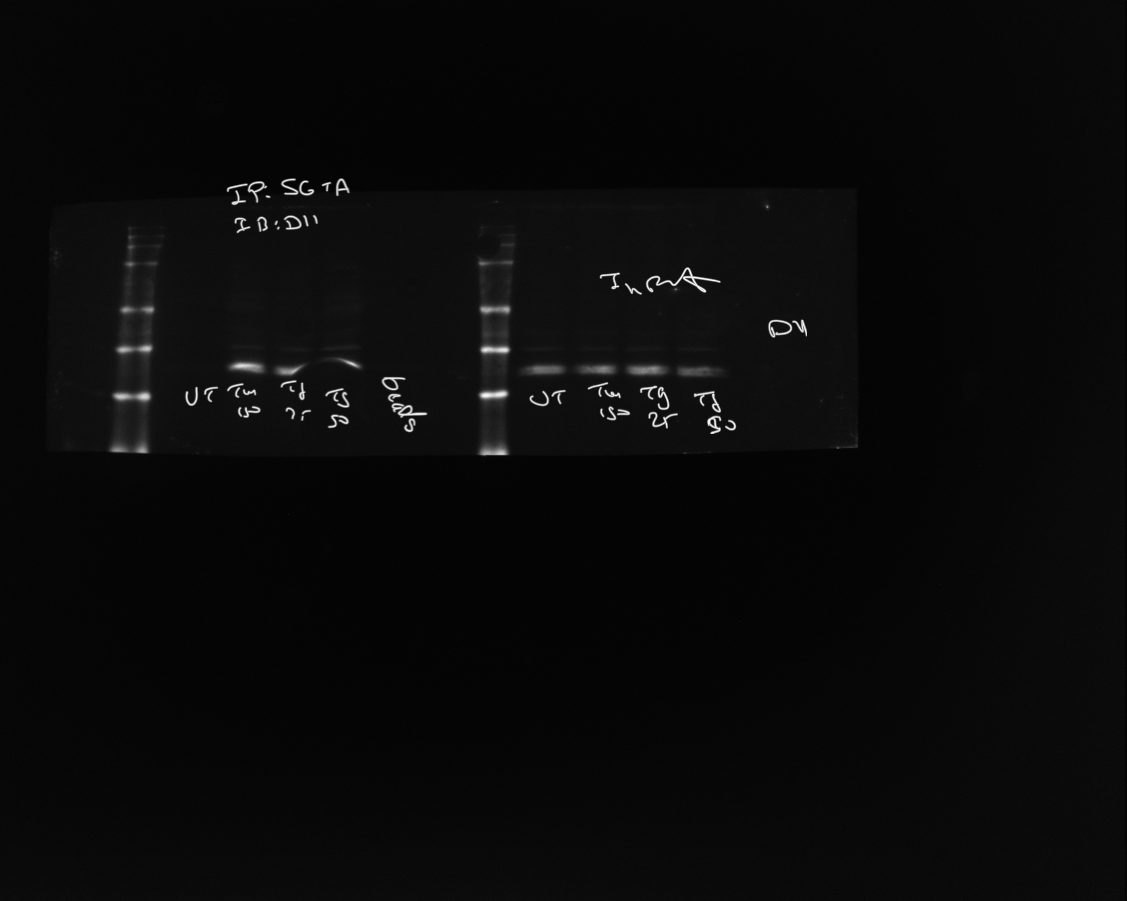

Supplement: Figure 5—figure supplement 1—source data 2. [file elife-102658-fig5-figsupp1-data2.zip › Figure 5-figure suplement 1-source data1/Figure 5-figure suplemment 1-E-2-source data1.tif]

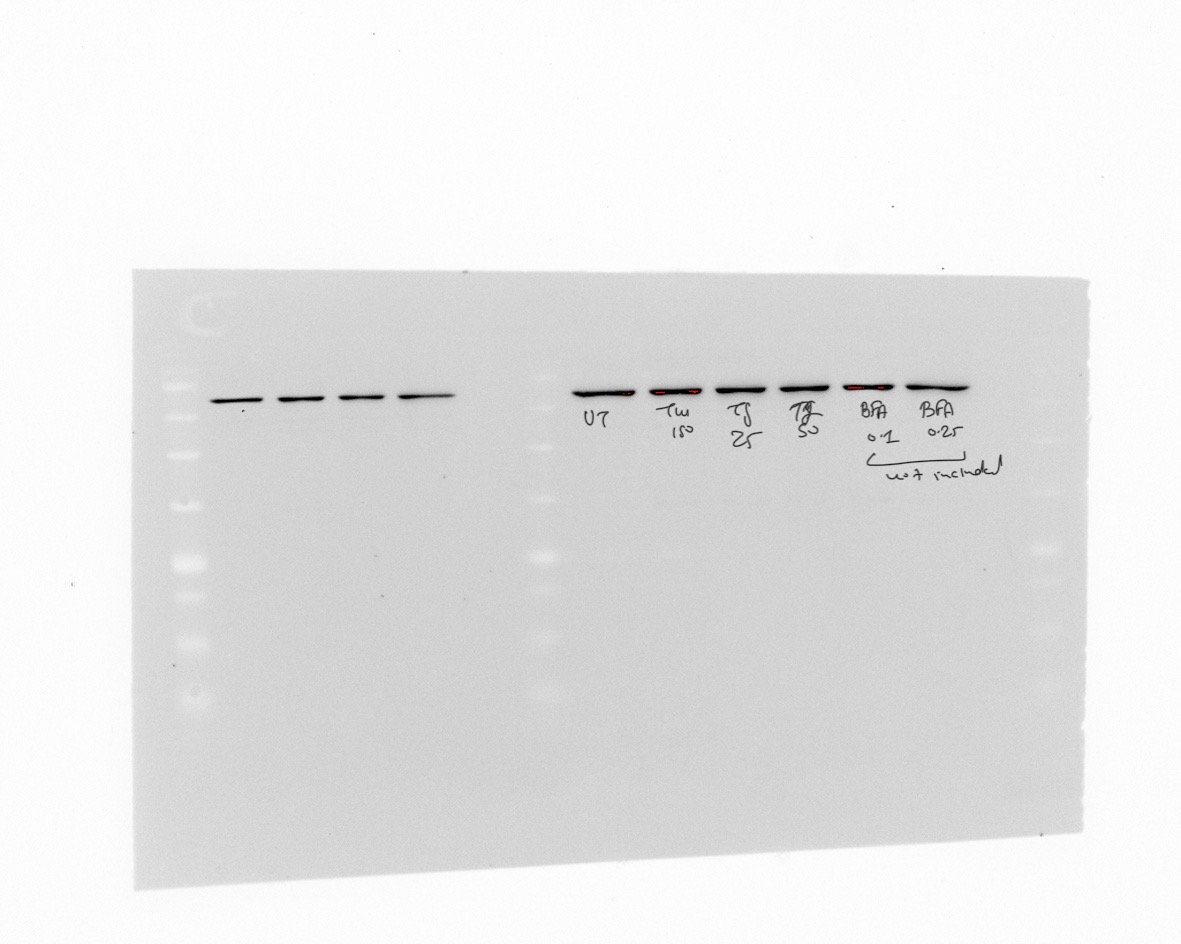

Supplement: Figure 5—figure supplement 1—source data 2. [file elife-102658-fig5-figsupp1-data2.zip › Figure 5-figure suplement 1-source data1/Figure 5-figure suplemment 1-C-6-source data1.jpg]

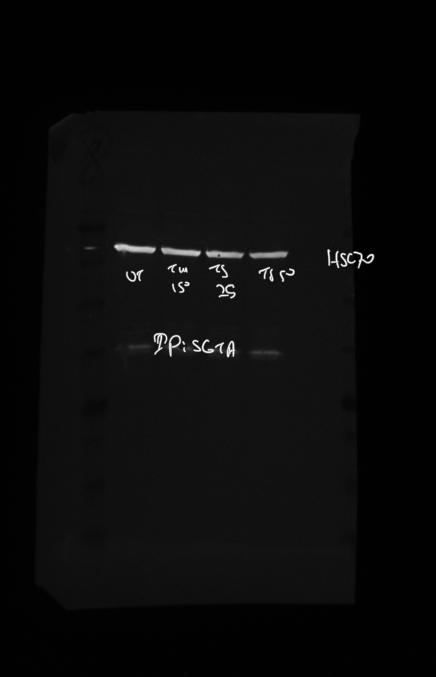

Supplement: Figure 5—figure supplement 1—source data 2. [file elife-102658-fig5-figsupp1-data2.zip › Figure 5-figure suplement 1-source data1/Figure 5-figure suplemment 1-C-3-source data1.tif]

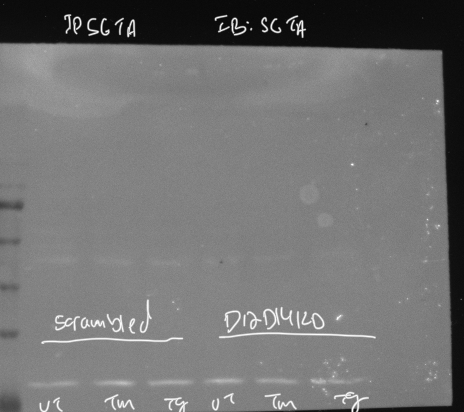

Supplement: Figure 5—figure supplement 1—source data 2. [file elife-102658-fig5-figsupp1-data2.zip › Figure 5-figure suplement 1-source data1/Figure 5-figure suplemment 1-F-2-source data1.tif]

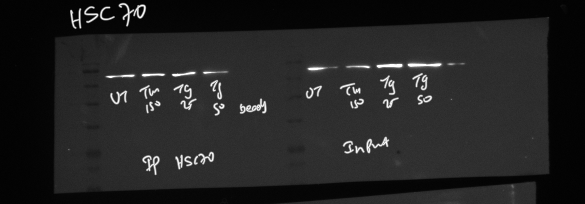

Supplement: Figure 5—figure supplement 1—source data 2. [file elife-102658-fig5-figsupp1-data2.zip › Figure 5-figure suplement 1-source data1/Figure 5-figure suplemment 1-D-1-source data1.tif]

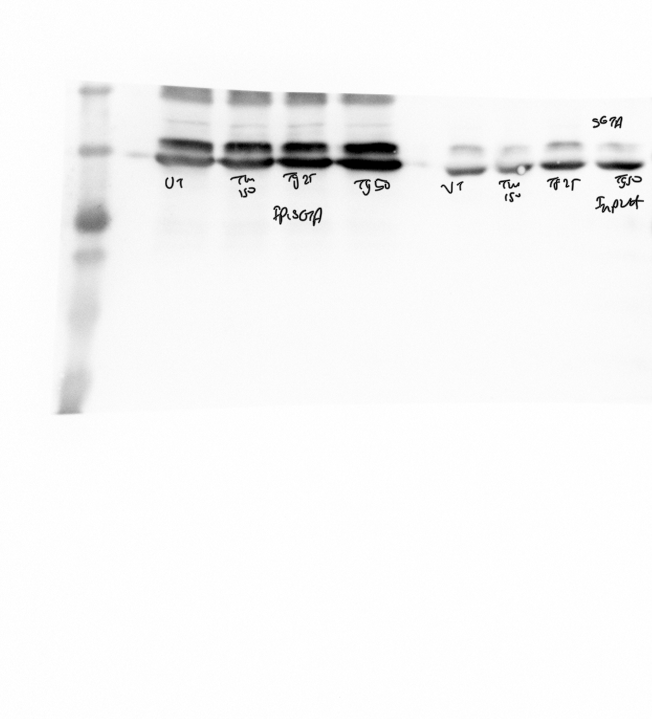

Supplement: Figure 5—figure supplement 1—source data 2. [file elife-102658-fig5-figsupp1-data2.zip › Figure 5-figure suplement 1-source data1/Figure 5-figure suplemment 1-B-4-source data1.tif]
