# Supplementary material for: Cytosolic and endoplasmic reticulum chaperones inhibit wt-p53 to increase cancer cells' survival by refluxing ER-proteins to the cytosol
Source: eLife. 2025 Apr 9;14:e102658. doi: 10.7554/eLife.102658 (PMC11981610; doi:10.7554/eLife.102658)
Supplement: Supplementary file 2. — (a) List of the different antibodies used in this study. (b) List of the commercially available siRNA used in this study. (c) List of oligos used for SGTA-CRISPRi from Gilbert et al., 2014. (d) List of oligos used for DNAJB12 and DNAJB14 cloning. [file elife-102658-supp2.docx]

**Supplement file 2a:**

| **Antibody (Host)** | **Company/Cat. No.** |
| --- | --- |
| HYOU1 (Rabbit) | Cell signalling #13452S |
| DNAJB12 (Rabbit) | Ptg/16780 |
| DNAJB14 (Rabbit) | Ptg/16501 |
| SGTA (Rabbit) | Ptg/11019 |
| SGTA (Mouse) | Ptg/ 60305-1-Ig |
| PDIA1 (Rabbit) | Ptg/11245-1-AP |
| PDIA1 (Mouse) | Ptg/66422-1-Ig |
| AGR2 (Rabbit) | Ptg/12275-1-AP |
| AGR2 (Mouse) | SantaCruz/sc-101211 |
| AGR2 (Rat) | Biolegend/943102 |
| DNAJB11 (Rabbit) | Ptg/15484-1-AP |
| PRDX4 (Rabbit) | Ptg/10703-1-AP |
| PRDX4 (Mouse) | Ptg/ 60286-1-Ig |
| pan-p53 (DO-1) (Mouse) | SantaCruz/sc-126 |
| p53 (Rabbit) | Ptg/ 10442-1-AP |
| p53 (Rabbit) | Ptg/60283 |
| phospho-p53 (Ser15) (Rabbit) | Ptg/28961 |
| phospho-p53 (Ser15) (Mouse) | Ptg/ 67826-1-Ig |
| p21 Waf1/Cip1 (12D1) (Rabbit) | Cell signaling #2947 |
| FLAG-DYKDDDDK (Mouse) | Ptg/66008-4 |
| FLAG-DYKDDDDK (Rabbit) | Cell signaling #14793 |
| GADPH (G-9) (Mouse) | SantaCruz/sc-365062 |
| HSP90 (4F10) (Mouse) | SantaCruz/sc- 69703 |
| HSC70 (Mouse) | SantaCruz/sc-7298 |
| Goat anti-Mouse IgG (H+L) Highly Cross-Adsorbed Secondary Antibody, Alexa Fluor™ Plus 488 | Invitrogen/ #A32723 |
| Goat anti-Rabbit IgG (H+L) Highly Cross-Adsorbed Secondary Antibody, Alexa Fluor™ Plus 488 | Invitrogen/ #A32731 |
| Goat anti-Rabbit IgG (H+L) Highly Cross-Adsorbed Secondary Antibody, Alexa Fluor™ Plus 647 | Invitrogen/ #A32733 |
| Goat anti-Mouse IgG (H+L) Highly Cross-Adsorbed Secondary Antibody, Alexa Fluor™ Plus 647 | Invitrogen/ #A32728 |
| Goat anti-Rabbit IgG (H+L) Highly Cross-Adsorbed Secondary Antibody, Alexa Fluor™ Plus 555 | Invitrogen/ #A32732 |
| Goat anti-Rat IgG (H+L) Cross-Adsorbed Secondary Antibody, Alexa Fluor™ 488 | Invitrogen/ #A-11006 |
| Goat anti-Rat IgG (H+L) Highly Cross-Adsorbed Secondary Antibody, Alexa Fluor™ Plus 647 | Invitrogen/ #A48265 |

**Supplement file 2b:**

| Scrambled sgRNA CRISPR/Cas9 All-in-One Lentivector | AmpR | ABM:K010 |
| --- | --- | --- |
| DNAJB12 sgRNA CRISPR/Cas9 All-in-One Lentivector set (Human#1) | AmpR | ABM:K0614305 |
|  |  |  |
| DNAJB12 sgRNA CRISPR/Cas9 All-in-One Lentivector set (Human#2) | AmpR | ABM:K0614305 |
|  |  |  |
| DNAJB12 sgRNA CRISPR/Cas9 All-in-One Lentivector set (Human#3) | AmpR | ABM:K0614305 |
|  |  |  |
| DNAJB14 sgRNA CRISPR/Cas9 All-in-One Lentivector set (Human#1) | AmpR | ABM:K0614505 |
|  |  |  |
| DNAJB14 sgRNA CRISPR/Cas9 All-in-One Lentivector set (Human#2) | AmpR | ABM:K0614505 |
|  |  |  |
| DNAJB14 sgRNA CRISPR/Cas9 All-in-One Lentivector set (Human#3) | AmpR | ABM:K0614505 |

**Supplement File 2c:**

SGTA-CRISPRi Oligos from Gilbert, L.A., et al., 2014 [1]

| SGTA-1,all,GACCGATCCCCGACCCACCGA | |
| --- | --- |
| SGTA-10,all,GGTCTGCGGCTCGGGGCCCA | |
| SGTA-2,all,GTCTGGGGTCTGCGGCTCG |  |
| SGTA-3,all,GAAGGAAGTGACGCAACGTAG | |
| SGTA-4,all,GGCGCCTTTCTTTTGCGC |  |
| SGTA-5,all,GCCAGGGTCACCGCGACCCGC | |
| SGTA-6,all,GCCTACTCACAGGACCCCGC |  |
| SGTA-7,all,GCGCAAGCGCAACCGTCGG |  |
| SGTA-8,all,GCACAGGCGCGTTAATGA |  |
| SGTA-9,all,GTAAGAGTTTGGGGATCGTG | |

**Supplement file 2d:**

| Oligo | Sequence |
| --- | --- |
| \| hDNAJB12_F_BamHI \| GGCGGAGGatccGTGGATGTCATCACTCCGCGCCCGGCTG \| \| --- \| --- \| \| hDNAJB12_R_NotI \| TGGTGTGGCGGCCgCAGGACTATCCATGCAGGGAGGCCTGCACCTCTG \| \| hDNAJB14-F-BamhI \| GGAGCAggatccAGCTATGGAGGGGAACAGGGATGAGGCTGAGAAAT \| \| hDNAJB14-R-NotI \| AAATTggcggcCgCAGTTCATCCTCCTTTATAAAGACTGGTAAGCC \| \| hDNAJB12_F_FLAG_BamhI \| GGCGGAGGatccGTGGATGGACTACAAAGACGATGACGACAAGTCATCACTCCGCGCCCGGCTGC \| \| hDNAJB14_F_HA_BamHI \| GGAGCAggatccAGCTATGTACCCATACGATGTTCCAGATTACGCTGAGGGGAACAGGGATGAGGCTGAG \| \| hDNAJB12-F_NheI \| CGGAGGgctagcGTGGATGTCATCACTCCGCGCCCGGCTG \| \| hDNAJB12-R_XbaI \| GGTGTGtctagaAGGACTATCCATGCAGGGAGGCCTGCACCTCTGA \| \| hDNAJB12_F_FLAG_NheI \| CGGAGGgctagcGTGGATGGACTACAAAGACGATGACGACAAGTCATCACTCCGCGCCCGGCTGCCC \| \| hDNAJB14-F_NheI \| GGAGCAgctagcAGCTATGGAGGGGAACAGGGATGAGGCTGAGAA \| \| hDNAJB14-R_XbaI \| AAATTgtctagaAGTTCATCCTCCTTTATAAAGACTGGTAA \| \| hDNAJB14_F_HA_NheI \| GGAGCAgctagcAGCTATGTACCCATACGATGTTCCAGATTACGCTGAGGGGAACAGGGATGAGGCTGAG \| | |

1. Gilbert, L.A., et al., *Genome-Scale CRISPR-Mediated Control of Gene Repression and Activation.* Cell, 2014. **159**(3): p. 647-61.
